# Multilocus Genotyping of Human *Giardia* Isolates Suggests Limited Zoonotic Transmission and Association between Assemblage B and Flatulence in Children

**DOI:** 10.1371/journal.pntd.0001262

**Published:** 2011-08-02

**Authors:** Marianne Lebbad, Ingvor Petersson, Lillemor Karlsson, Silvia Botero-Kleiven, Jan O. Andersson, Bo Svenungsson, Staffan G. Svärd

**Affiliations:** 1 Department of Diagnostics and Vaccinology, Swedish Institute for Communicable Disease Control, Solna, Sweden; 2 Microbiology and Tumor Biology Centre, Karolinska Institutet, Stockholm, Sweden; 3 Department of Communicable Disease Control and Prevention, Stockholm County Council, Stockholm, Sweden; 4 Department of Clinical Microbiology, Karolinska University Hospital, Stockholm, Sweden; 5 Department of Molecular Evolution, Evolutionary Biology Centre, Uppsala University, Uppsala, Sweden; 6 Department of Cell and Molecular Biology, Uppsala University, Uppsala, Sweden; New York University School of Medicine, United States of America

## Abstract

**Background:**

*Giardia intestinalis* is one of the most common diarrhea-related parasites in humans, where infection ranges from asymptomatic to acute or chronic disease. *G. intestinalis* consists of eight genetically distinct genotypes or assemblages, designated A–H, and assemblages A and B can infect humans. Giardiasis has been classified as a possible zoonotic disease but the role of animals in human disease transmission still needs to be proven. We tried to link different assemblages and sub-assemblages of *G. intestinalis* isolates from Swedish human patients to clinical symptoms and zoonotic transmission.

**Methodology/Principal Findings:**

Multilocus sequence-based genotyping of 207 human *Giardia* isolates using three gene loci: ß-giardin, glutamate dehydrogenase (*gdh*), and triose phosphate isomerase (*tpi*) was combined with assemblage-specific *tpi* PCRs. This analysis identified 73 patients infected with assemblage A, 128 with assemblage B, and six with mixed assemblages A+B. Multilocus genotypes (MLGs) were easily determined for the assemblage A isolates, and most patients with this genotype had apparently been infected through anthroponotic transmission. However, we also found evidence of limited zoonotic transmission of *Giardia* in Sweden, since a few domestic human infections involved the same assemblage A MLGs previously reported in Swedish cats and ruminants. Assemblage B was detected more frequently than assemblage A and it was also more common in patients with suspected treatment failure. However, a large genetic variability made determination of assemblage B MLGs problematic. Correlation between symptoms and assemblages was found only for flatulence, which was significantly more common in children less than six years of age infected with assemblage B.

**Conclusions/Significance:**

This study shows that certain assemblage A subtypes are potentially zoonotic and that flatulence is connected to assemblage B infections in young children. Determination of MLGs from assemblages A and B can be a valuable tool in outbreak situations and to help identify possible zoonotic transmission.

## Author Summary

*Giardia intestinalis* is a protozoan parasite found world-wide and it is a major cause of diarrhea in humans and other mammals. The genetic variability within *G. intestinalis* is high with eight distinct genotypes or assemblages (A-H). Here we performed sequence-based multilocus genotyping of around 200 human *Giardia* isolates. We found evidence of limited zoonotic transmission of certain A subtypes and an association between flatulence and assemblage B infection in children. This shows that it is important to investigate different assemblages and sub-assemblages of *G. intestinalis* in human infections in order to understand the clinical significance, zoonotic potential, sequence divergence, and transmission pathways of this parasite.

## Introduction

*Giardia intestinalis* (synonyms: *G. lamblia*, *G. duodenalis*) is a protozoan parasite that infects a wide array of vertebrates, including humans, pets, livestock, wildlife, and marine animals [1], [2]. *Giardia* has a global distribution and is one of the most common diarrhea-related parasites in humans, where infection ranges from asymptomatic to symptomatic, involving both acute and chronic disease. According to estimates, about 200 million people worldwide have symptoms of intestinal giardiasis, and 500 000 new cases occur annually [3]. Due to its impact on health, especially among children in developing countries, *Giardia* has been included in the Neglected Diseases Initiative of the World Health Organization (WHO) since 2004 [4].

*Giardia intestinalis* consists of eight morphologically identical but genetically distinct genotypes or assemblages, designated A–H [1], [5]. Assemblages A and B can infect humans and other mammals, whereas assemblages C–H appear to be host specific. Giardiasis has been classified as a possible zoonotic infection by the WHO since 1979 [6], and studies conducted in India and Thailand [7], [8] have suggested zoonotic transmission of *Giardia*; nonetheless, the role of animals in human disease transmission still needs to be proven [9]–[11]. Several investigations have also tried to link the severity of infection to a certain assemblage, but the results have been inconclusive [12]–[16]. Most of those studies relied on genotyping using only one or two genetic markers, such as the small subunit ribosomal RNA (*ssrRNA*), ß-giardin, glutamate dehydrogenase (*gdh*), or triose phosphate isomerase (*tpi*) gene. The information thus obtained has limited discriminatory power, and it has recently been suggested that a special multilocus genotyping approach should be used to compare multilocus genotypes (MLGs) from different sources [17]. Lately, the occurrence of mixed assemblage infections has also gained attention through research in which use of assemblage-specific *tpi* primers has allowed much more extensive detection of mixed assemblage A and B infections than is feasible when using more general primers [18], [19]. Accordingly, in-depth studies that focus on several genetic loci and use assemblage-specific primers are needed to clarify both the issue of zoonotic transmission and the correlation between assemblage and disease pattern. It might also be possible to use this combination of methods for source tracing in outbreak situations.

In Sweden, giardiasis has been a notifiable disease since 1989, and 1200–1500 cases are reported each year in a population of about nine million. Although most cases are imported, domestic transmission occurs as well. Recently, a study of *Giardia* genotypes in animals in Sweden was published [20], but molecular characterization of human *Giardia* isolates in this country has been limited to an outbreak involving three nursery schools, where the only subtype found was A3, as determined at the ß-giardin locus [21].

The purpose of the present study was to determine the genetic variability of *G. intestinalis* isolated from patients with infections acquired in Sweden and abroad. Multilocus genotyping was used as a tool to investigate *Giardia* with regard to the relationship between assemblages and symptoms, the zoonotic potential, sequence divergence, and possible transmission dynamics.

## Materials and Methods

### Sources of isolates and patient information

Fecal samples from 214 patients with *Giardia* infection diagnosed by light microscopy at Karolinska University Hospital, Stockholm, between May 2007 and April 2009 were referred to the Swedish Institute for Communicable Disease Control (SMI) in Solna. The majority of the study participants (including nine adopted children) had consulted a physician due to intestinal symptoms (n = 175). The remaining cases (n = 39) were detected in connection with health check-ups (adopted children n = 9, other subjects n = 10) or through source tracing (n = 20). In addition, 11 patients provided a second fecal sample that was microscopically positive after treatment. Besides routine parasitological examination of all samples, 179 of the fecal specimens were also cultured for bacterial enteropathogens by standard methods. Information regarding travel abroad within two weeks prior to onset of disease, symptoms, treatment, and possible routes of transmission was obtained by use of postal questionnaires or telephone interviews.

### Ethics statement

The study was approved by the Regional Ethics Committee of Karolinska Institutet, Stockholm, Sweden. Written consent for being part of the study was obtained from each patient and parents or primary caretakers signed for their children.

### Microscopy and DNA extraction

The *Giardia* cysts were studied after concentration by the formol/ethyl acetate technique, using direct immunofluorescent labeling with a monoclonal antibody (Agua-Glo, Waterborne Inc., New Orleans, LA, USA) and counterstaining with DAPI (4′6-diamidino-2-phenyl-indole). DNA was extracted directly from stools using a QIAamp DNA Mini Kit (Qiagen, Hilden, Germany) according to the manufacturer's recommendations. Before carrying out the extraction procedure, the cysts were disrupted in a Mini-BeadBeater (Biospec Products Inc., Bartlesville, OK, USA) [22]. For a limited number of samples (n = 13) showing no initial amplification, the cysts were isolated on a sucrose gradient and subjected to a renewed extraction [22].

### Molecular methods

All 225 samples were analyzed using a nested ß-giardin PCR [23] a semi-nested *gdh* PCR [24], and a nested *tpi* PCR [25], with the expected amplicons of 511, 432, and 530 bp, respectively. Using conditions described elsewhere, restriction fragment length polymorphism (RFLP) analysis was performed on aliquots of the products from all three PCR amplifications: ß-giardin [23] *gdh* [24], and *tpi* [26]. All samples were also analyzed using assemblage A- and B-specific *tpi* PCR primers [19], [27] with the expected amplicons of 373 and 400 bp, respectively. All amplicons generated in the ß-giardin PCR and the majority of amplicons from the *gdh* and *tpi* PCRs were sequenced in both directions. In addition, an alternative nested *gdh* PCR with subsequent sequencing as well as sequencing of the amplicons obtained from single ß-giardin PCR was performed on a limited number of assemblage A isolates [17],[28]. The sequences thus obtained were used to create a concatenated assemblage A tree. Chromatograms and sequences were examined using the BioEdit sequence analysis program (http://www.mbio.ncsu.edu/BioEdit/page2.html). The BLAST tool (http://www.ncbi.nlm.nih.gov/blast/) was used to compare nucleotide sequences with sequences in the GenBank database.

Representative nucleotide sequences without ambiguous positions have been deposited in GenBank under the following accession numbers: GQ329671-GQ329679, HM1407-HM140725, HM136880-HM136891, HM165208-HM165227, and JF773747-JF773759. In addition all ß-giardin, *gdh*, and *tpi* sequences from 120 assemblage B isolates, positive at all three loci, have been appended as Supplementary Files S1, S2, S3.

### Phylogenetic analysis

For phylogenetic analyses, MLGs with unambiguous sequences identified in the current investigation and in our previous study of animals in Sweden [20] were combined with reference sequences of representative isolates from GenBank (Supplementary Table S1). After removal of primer sequences, the ß-giardin, *gdh*, and *tpi* datasets for assemblage A consisted of 700 (product from single ß-giardin PCR), 694 (merged products from two *gdh* PCRs), and 490 nucleotides, respectively, and the corresponding datasets for assemblage B comprised 475, 393, and 490 nucleotides. RAxML version 7.0.4 [29] was used to perform maximum likelihood analyses with the GTR substitution model and among-site rate variation (GTRGAMMA), and the same approach was applied in bootstrap analyses with 1000 replicates.

### Statistical methods

Chi squared test or Fisher's exact test, as appropriate, were used to evaluate differences between characteristics of patients infected with different assemblages.

## Results

### Combined results of ß-giardin, gdh, and tpi PCR amplifications

Altogether, *Giardia* isolates from 207 of the 214 patients were successfully genotyped. PCR analyses yielded the expected amplicons for all three genes in 192 isolates, for two genes in nine isolates, and for one gene in six isolates. Seven isolates were negative in all three PCRs despite repeated trials. Six of these seven exhibited only a few cysts, which were all DAPI negative; the seventh sample did contain DAPI-positive cysts, but it had most likely been exposed to formalin before extraction. Of the 11 additional samples that were microscopically positive after treatment, 10 were positive for all three genes, whereas one sample was negative.

### PCR-RFLP and assemblage A- and B-specific tpi PCR

**Figure 1:**
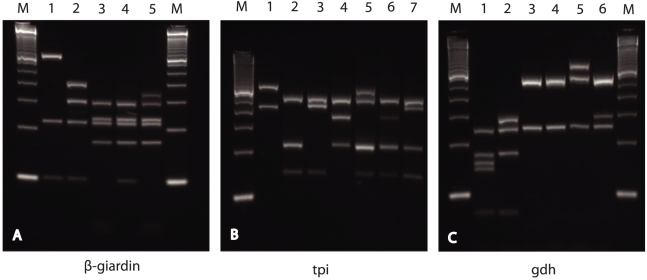
PCR-RFLP analysis of Giardia isolates. Panel A: Gelred (Biotium) stained 3.5% MetaPhor agarose gel (Cambrex) showing electrophoretic separation of nested β-giardin PCR products (511 bp) after digestion with HaeIII: lane 1, assemblage A (novel pattern Sweh166, 173, 178); lane 2, assemblage A (ordinary pattern); lane 3, assemblage B (ordinary pattern), lane 4, assemblage B (novel pattern Sweh198); lane 5, assemblage B (mixed pattern). Panel B: Electrophoretic separation of nested *tpi* PCR products (530 bp) after digestion with DdeI: lane 1, assemblage A; lane 2, assemblage B (ordinary pattern); lane 3, assemblage B (novel pattern Sweh121), lane 4, assemblage B (novel pattern Sweh154); lanes 5, 6 and 7, assemblage B (mixed patterns). Panel C: Electrophoretic separation of semi-nested *gdh* PCR products (432 bp) after digestion with NlaIV: lane 1, assemblage AII; lane 2, assemblage AI; lanes 3 and 4, assemblage B; lanes 5 and 6, assemblage B (mixed patterns). Molecular size markers (M) are 50-bp ladders (Invitrogen).

RFLP analysis was successful for all isolates amplified at the ß-giardin, *gdh*, and *tpi* loci (Table 1). Most of the isolates showed the expected RFLP patterns for assemblages A and B at all three genes. Patterns corresponding to mixed assemblage A and B infections were observed in three isolates (Table 2). Three other isolates (Sweh166, 173, and 178) that had a sub-assemblage AI pattern in the *gdh* RFLP displayed a novel assemblage A pattern at the ß-giardin gene (Fig. 1). This pattern has recently been observed in samples from ruminants and cats in Sweden (unpublished data). Considering assemblage B, one new pattern was observed at the ß-giardin gene (Sweh198), and two new patterns were found at the *tpi* gene (Sweh121 and Sweh154) (Fig. 1). Additional bands in the RFLPs of assemblage B were seen in all three genes: ß-giardin (n = 1), *gdh* (n = 11), and *tpi* (n = 9) (Fig. 1). Sequence analyses of these isolates revealed nucleotide substitutions or overlapping nucleotides (double peaks in chromatograms) at different restriction sites, which explains both the new patterns and the additional bands. The results obtained using the assemblage A- and B-specific *tpi* primers confirmed all findings of the RFLP analysis of the *tpi* gene and added three more mixed A+B infections that were not detected in any of the RFLPs (Tables 1 and 2). The additional findings were confirmed by sequencing the amplicons from the assemblage-specific *tpi* PCR. All 16 samples that were negative with the original *tpi* primers were also negative with the assemblage-specific primers. The combined results of all RFLPs and assemblage-specific *tpi* PCR demonstrated 73 patients with assemblage A, 128 with assemblage B, and six with mixed assemblages A+B (Tables 1 and 2).

**Table 1:** Distribution of assemblages among 207 isolates determined by PCR-RFLP and assemblage A- and B-specific PCR.

	Assemblages				
	A	B	A+B	Not amplified	Total positive
ß-giardin RFLP	74^a^	124^b^	2	14	200
*gdh* RFLP	73^c^	129	0	12	202
*tpi* RFLP	72	124^d^	2	16	198
*tpi* A+B PCR	70	122	6	16	198
Combined results	73	128	6	7	207

a Including three samples with a novel A pattern (Sweh166, 173, and 178).

b Including one sample with a novel B pattern (Sweh198).

c Including three samples with AI (Sweh166, 173, and 178) and 70 with AII patterns.

d Including two samples with novel B patterns (Sweh121 and 154).

**Table 2:** Molecular characterization of isolates from patients presenting with mixed assemblage A and B infections.

Isolate	ß-giardin^a^	*tpi* a	*gdh* a	*tpi* A+B PCR	Origin of infection	Co-infections	Patient information
Sweh068	B^b^	B^b^	B^b^	A^c^+B	Ethiopia	*Campylobacter*	Adoptive child
Sweh098	A3	AII	AII	A+B^c^	India	*Blastocystis*	Adult tourist
Sweh110	A+B	A+B	B^b^	A^c^+B	India	*Blastocystis*	Adult tourist
Sweh131	A2	A+B	AII	A+B^c^	China	None	Adoptive child
Sweh140	A3	AII	AII	A+B^c^	India	*Shigella* and *Campylobacter*	Adult tourist
Sweh207	A+B	B^b^	B^b^	A^c^+B	India	*Blastocystis*	Adult tourist

a Data based on RFLP and sequencing.

b Sequences containing overlapping nucleotides.

c Confirmed by sequencing.

### Sequencing and phylogenetic analyses

In total, 200 ß-giardin, 195 *gdh*, and 195 *tpi* amplicons were sequenced, including all the 192 isolates that were positive at all three genes.

### Assemblage A

Sequencing of 74 assemblage A isolates at the ß-giardin locus revealed 36 isolates with subtype A2, 30 with subtype A3, and five with a mixture of subtype A2 and A3, as demonstrated by overlapping nucleotides at subtype specific positions in the chromatograms. Furthermore, three assemblage A isolates that exhibited a new ß-giardin RFLP assemblage A pattern had sequences identical to GenBank acc. no. AY655702, previously reported from cats, ferrets and various ruminants [17], [20], [30]–[32]. At the *tpi* locus, these three isolates corresponded to GenBank acc. no. EU781027, a sequence identified in different animal species in Sweden [20]. However, at the *gdh* locus, the three sequences were either identical to sub-assemblage AI or to GenBank acc. no. EU769224, which was recently found in a fallow deer and a cat in Sweden [20]. In addition, one assemblage A isolate at the *tpi* locus had a sequence that corresponded to the isolate ISSGd85 (GenBank acc. no. EU041753) obtained from a human in Western Sahara [26]. All remaining assemblage A isolates that were sequenced at the *gdh* (n = 69) and *tpi* (n = 67) loci corresponded to sub-assemblage AII. Apart from the five isolates with mixed A2–A3 subtypes at the ß-giardin gene and one isolate with overlapping nucleotides at position 445 of the *tpi* gene, all other assemblage A isolates had sequences without ambiguous positions (Table 3).

**Table 3:** Characterization of 67 assemblage A isolates* based on sequencing data and assemblage-specific PCR.

No. of isolates (isolate code)	ß-giardin	*tpi*	*gdh*	*tpi* A+B	MLG
31	A2	AII	AII	A	AII-1
1 (Sweh037)	A2	AII^a^	AII	A	Mixed
1 (Sweh038)	A2	AII^b^	AII	A	AII novel
26	A3	AII	AII	A	AII-2
5	A2+A3^c^	AII	AII	A	Mixed AII-1+AII-2
2 (Sweh166, 178)	A^d^	A^e^	AI	A	A novel^g^
1 (Sweh173)	A^d^	A^e^	A^f^	A	A novel^h^

*Isolates with mixed assemblage A+B infections are not included.

a A/G at position 445.

b GenBank acc. no. EU041753 (isolate ISSGd85, human).

c C/T at position 460 and 468.

d GenBank acc. no. AY655702 (unnamed isolate, cattle).

e GenBank acc. no. EU781027 (isolate Swecat170, cat).

f GenBank acc. no. EU769224 (isolate Swecat202, cat).

g Equal to previous MLG from various Swedish animals (cats and ruminants).

h Equal to previous MLG from Swefd154 (fallow deer) and Swecat202 (cat).

### Multilocus genotyping of assemblage A isolates

**Figure 2:**
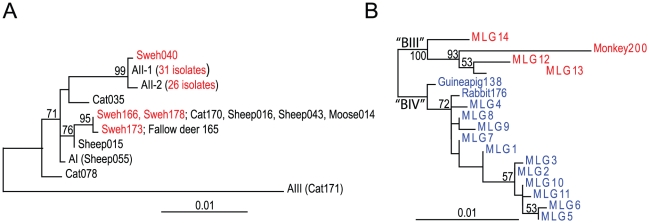
Nucleotide maximum likelihood trees based on concatenated datasets for ß-giardin, *gdh*, and *tpi* gene sequences. MLGs with unambiguous sequences identified in this study (Table 3 and 4) are combined with reference isolates and isolates from our previous MLG study of animals in Sweden [20] (Supplementary Table S1). (A) Phylogenetic tree based on 1884 aligned positions of assemblage A isolates. Isolates identified in the present study are indicated in red. (B) Phylogenetic tree based on 1358 aligned positions of 17 assemblage B MLGs identified in our present and our previous study [20]. BIII (red) and BIV (blue) isolates are assigned according to their clustering with reference isolates in phylogenetic trees of the individual genes (Fig. S1). Only bootstrap support values >50% are shown.

Five different assemblage A MLGs could be identified in 67 isolates that had single assemblage A infections and had been sequenced on all three genes (Table 3). Two of these, MLG AII-1 and MLG AII-2, were found most frequently, whereas the other three were seen in only a few cases. A phylogenetic analysis was performed on these five MLGs, along with two reference MLGs (AI and AIII) and three MLGs unique to our previously conducted genotyping of animals in Sweden [20] (Table 3 and Fig. 2A). Three of the MLGs branched with AII isolates, whereas the remaining two MLGs (from Sweh166, 178, and 173), which are shared with animals, could not be conclusively categorized as AI or AII (Fig. 2A).

### Assemblage B

A much more complex picture was found for assemblage B than for assemblage A. At the ß-giardin locus, 59 of 124 (48%) sequenced assemblage B isolates exhibited no double peaks at any position in the chromatograms, and subtypes could be determined. These 59 isolates corresponded to 10 previously reported and 10 new subtypes. The novel subtypes were represented by one isolate each, whereas the two previously reported subtypes B1-1 and B1-3 (GenBank acc. nos. EU637579 and EU881698; subtype names as suggested by Geurden and collegues [18]) were found in nine and 18 patients, respectively. At the *tpi* locus, 50 out of 122 (41%) sequenced assemblage B isolates had no double peaks in the chromatograms. Sixteen different subtypes were identified, seven of which had already been described and nine were novel. The most common subtype was found in 21 isolates and corresponded to BIV isolate Ad-19 (GenBank acc. no. AF069560). Of the 123 assemblage B isolates that were sequenced at the *gdh* locus, 44 (36%) displayed no double peaks in the chromatograms, whereas the remaining 79 exhibited from one to 14 positions with overlapping nucleotides. Twelve different subtypes were seen, five of them previously reported and seven new. Two subtypes were frequently seen and corresponded to two different BIV isolates, in 18 cases Ad-7 (GenBank acc. no. L40508) and in 12 cases gd-ber12 (GenBank acc. no. DQ923581). The remaining 10 subtypes were seen in one to four isolates each.

### Multilocus genotyping of Assemblage B isolates

Thirty-one of 120 assemblage B isolates that were successfully sequenced at all three loci and without overlapping nucleotides at any position were grouped into 14 different MLGs (Table 4). Phylogenetic analyses of individual genes (Supplementary Fig. S1) and concatenation of the three genes (Fig. 2B) indicated that MLGs 12, 13, 14 and the monkey isolate (Monkey200) branched together to the exclusion of the other human MLGs, the guinea pig (Guinea pig138) and the rabbit (Rabbit176) MLGs, with 100% bootstrap support (Fig. 2B). In the phylogenies of *gdh* and *tpi*, MLGs 12–14 and Monkey200 branched together with BIII isolates, whereas the other MLGs branched with BIV isolates, Guinea pig138 and Rabbit176 (Supplementary Fig. S1B and C). The separation into BIII and BIV was not clear in the ß-giardin phylogeny (Supplementary Fig. S1A), although the bootstrap support for the alternative topology was low. Table 4 shows that a few “subtypes” from each locus (B1-1, B1-3, and B1-5 at ß-giardin; Ad-19 and GS/M at *tpi*; Ad-7, gd-ber 12, and Vanc89/UBC/059 at *gdh*) were identified in many of the MLGs, but the grouping was not consistent, because they appeared in different combinations.

**Table 4:** Characterization of 31 assemblage B isolates based on the ß-giardin, *gdh*, and *tpi* gene sequences*.

Isolate	Isolate/Subtype/GenBank accession number			MLG	Intestinal symptoms	Origin of infection
	ß-giardin	*tpi*	*gdh*			
Sweh001Sweh021, 022^a^Sweh041Sweh051, 056, 057, 058^a^Sweh156Sweh159, 160^a^Sweh213Sweh217	B1-3EU881698	Ad-19/BIVAF069560	Ad-7/BIVL40508	1	NoYes, YesYesYes, No, No, NoYesYes, NoYesYes	SwitzerlandSweden, Malta^b^SwedenSwedenSpainSwedenSwedenUSA
Sweh059	B1-3	Ad-19/BIV	gd-ber12, DQ923581	2	Yes	Finland
Sweh144	B1-3	GS/M/BIV/L02116	gd-ber12	3	Yes	Sweden
Sweh158Sweh199, 200, 202^a^	B1-1EU637579	Novel BHM140723	Ad-7/BIV	4	YesYes, No, No	USASweden
Sweh168	B1-1	Ad-19/BIV	gd-ber12	5	Yes	Sweden
Sweh154	B1-1	M12/BIV EU834845	gd-ber12	6	Yes	Sweden
Sweh074	B1-1	Ad-19/BIV	Ad-7/BIV	7	Yes	Mauritius
Sweh179	B1-1	GS/M BIV	Vanc89/UBC/059AY178750	8	Yes	Nicaragua
Sweh033	B1-2EU881697	ST25DQ789114	Vanc89/UBC/059BIV	9	Yes	Canada
Sweh192	B1-5EU881700	Ad-19/BIV	gd-ber12	10	Yes	Sweden
Sweh047, 048, 049^a^	B1-5	GS/M/BIV	gd-ber12	11	Yes, Yes, No	Sweden
Sweh060	BG-Ber6 DQ090527	2434AY368165	Novel BHM136881	12	Yes	Sweden
Sweh136	ISSGF4/B4 AY072728	BAH12/BIII AF069561	Novel BHM136884	13	Yes	Germany
Sweh107	Novel B HM165222	Novel BHM140716	BAH12/BIII AF069059	14	Yes	India

*The sequences contained no ambiguous positions at any loci. Identical sequences are marked in the same color.

a Family cluster.

b Country of infection for index case.

### Allelic sequence divergence in Assemblage B

Overlapping nucleotides were frequently observed in assemblage B sequences and occurred at the ß-giardin gene in 52% of the isolates, at the *tpi* gene in 59%, and at the *gdh* gene in 64%. In Supplementary Tables S2A–C, the present ß-giardin, *tpi*, and *gdh* sequencing data from 120 assemblage B isolates are compared with corresponding data from reference BIII and BIV isolates. Double peaks occurred at 47 different positions in the sequenced 511-bp fragment of the ß-giardin gene, at 88 positions in the 530-bp fragment of the *tpi* gene, and at 59 positions in the 432-bp fragment of the *gdh* gene; note that only those positions where nucleotide substitutions or overlapping nucleotides occurred in five or more isolates are shown in the tables. Supplementary Tables S2A–C illustrate widespread occurrence of overlapping nucleotides in the positions that Wielinga and Thompson [33] have proposed to differentiate between sub-assemblage BIII and BIV. Supplementary Table S2A shows that 30% of the isolates (36/120) had C and T at position 354 of the ß-giardin gene, the only position suggested to differentiate between BIII and BIV in this locus [19]. Considering the *tpi* gene, the five positions 39, 91, 165, 168, and 210 have been proposed for differentiation [33], and in our study double peaks were frequently observed for all of these positions in the chromatograms (24%, 27%, 33%, 24%, and 12%, respectively; Supplemenatry Table S2B). At the *gdh* gene positions 309, 429, 447, 540, 561 and 612 have been suggested for sub-assemblage differentiation [33], but again we found overlapping nucleotides in all those positions (38%, 32%, 36%, 46%, 18%, and 41%, respectively; Supplementary Table S2C).

### Patient data

The ages of the patients ranged from 0 to 76 years (median 31.5, mean 30.4); 109 were female and 105 male. The distribution of assemblages in relation to gender among the 207 patients whose isolates were successfully genotyped was as follows: 36 females had assemblage A, 64 assemblage B, and four mixed assemblages A+B; among the males, 37 had assemblage A, 64 assemblage B, and two mixed assemblages A+B. The age distribution was as follows: 0–5 years (n = 40), 6–10 years (n = 19), 11–20 years (n = 9), and >20 years (n = 146). Clinical and epidemiological data were obtained from 181 patients in the form of survey responses. For the remaining 33 participants, designation of the country of origin of the infection and partial information concerning symptoms were acquired from the mandatory notification submitted by the clinician according to the Swedish Communicable Diseases Act.

### Origin of infection

Table 5 shows the distribution of assemblages in relation to country of infection. Fifty-three patients with symptoms of giardiasis did not report any travel outside Sweden within 14 days prior to the onset of their symptoms and were thus considered to represent domestic infections, and 13 asymptomatic patients were also regarded as domestic infections based on previous history of travel. The majority of the patients (61%) were infected outside Europe and more than half of those in various Asian countries. Assemblage B was the most prevalent assemblage from all parts of the world except Latin America, for which there was more equal distribution of assemblages A and B (Table 5).

**Table 5:** Probable area of origin of infection in 214 giardiasis patients presented in relation to assemblages.

	Sweden	Other European countries	Africa	Asia^a^	Latin America	North America	Not stated	Total
Assemblage A	22	5	11	22	12	0	1	73
Assemblage B	42	11	20	43	9	3	0	128
Assemblage A+B	0	0	1	5		0	0	6
PCR negative	2	0	3	1	1	0	0	7
All cases	66	16	35	71	22	3	1	214

a Thirty-eight infections originating from Asia were acquired in India, and six of those were assemblage A, 28 assemblage B, and four assemblage A+B.

### Microbiological investigation

Bacterial investigations performed on 179 samples revealed that 12 patients had co-infections with the following bacteria: *Campylobacter* (n = 7), *Shigella* (n = 2), *Salmonella* (n = 1), and both *Campylobacter* and *Shigella* (n = 2). Parasitological examination of all samples detected several additional parasites: *Enterobius vermicularis* (n = 3), *Trichuris trichiura* (n = 1), *Hymenolepis nana* (n = 1), *Cryptosporidium* spp. (n = 4), *Blastocystis* spp. (n = 31), *Entamoeba dispar* (determined by PCR) (n = 2), and other non-pathogenic amoebas (n = 26).

### Correlation between assemblages and symptoms

Table 6 presents clinical data from 145 symptomatic persons who responded to the survey and had no co-infections with other diarrhea-related enteropathogens. The only correlation found between symptoms and infection with specific assemblages was noted for flatulence, which was reported more frequently by patients infected with assemblage B (p = 0.006, Table 6). However, this correlation was no longer apparent when the analysis was restricted to patients over five years of age (p = 0.16, data not shown). Separate analyses of symptomatic children aged 0–5 years (n = 12) revealed six children that were infected with assemblage B, and they all suffered from flatulence; six had assemblage A infection, and none of them experienced flatulence. Thus, flatulence was significantly more common in children infected with assemblage B (p = 0.0022). Ten patients, two of whom were co-infected with *Campylobacter* and *Shigella*, were hospitalized due to diarrhea and treated with intravenous fluids. Seven of these subjects were infected with assemblage B and two with assemblage A, and one was infected with assemblages A+B. The two patients with assemblage A (Sweh166 and 178) were both infected in Sweden with a unique MLG hitherto found only in animals in this country (Table 3).

**Table 6:** Symptoms of 145 giardiasis patients in relation to assemblage (mixed infections with other enteropathogens excluded).*

	Patients infected with *Giardia intestinalis*		
Symptoms	Assemblage A (n = 51)	Assemblage B (n = 87)	All patients (n = 145^c^)
Diarrhea^a^	48/51 (94)	86/87 (99)	141/145 (97)
Bowel movements^a^			
<3/day	9/51 (18)	20/87 (23)	30/145 (21)
3–5/day	12/51 (24)	31/87 (36)	44/145 (30)
>5/day	27/51 (53)	35/87 (40)	67/145 (46)
Abdominal pain^a^	33/50 (66)	55/85 (65)	94/142 (66)
Bloody stools^a^	3/50 (6)	5/87 (6)	8/144 (6)
Vomiting^a^	17/50 (34)	29/87 (33)	47/144 (33)
Flatulence^b^	33/51 (65)	73/86 (85)	112/144 (78)
Fever >38°C^a^	12/50 (24)	19/85 (22)	32/142 (23)
Loss of weight^a^	33/50 (66)	65/85 (76)	103/142 (73)

NOTE: Response rates for the different items on the questionnaires varied from 98% to 100%.

*The data given represent number of findings/number of patients who answered the specific questions (%).

a No significant difference between the assemblage A and B patient groups.

b Significant difference between the assemblage A and assemblage B patient groups, p = 0.006.

c Includes four patients with assemblage A+B and three with negative PCR results.

### Family clusters

Eighteen family clusters involving a total of 46 individuals (2–5 per cluster) were identified. In 16 of these clusters, samples from two or more individuals were successfully genotyped. Six families were infected with assemblage A and eight with assemblage B, whereas in two families the members were infected with either assemblage A or B. Multilocus genotyping was successful in all six assemblage A (data not shown) and in five of the assemblage B-infected family clusters (Table 4). All members in each of these family clusters shared the same MLGs.

### Treatment

Information on treatment of giardiasis was available for 173 patients: 108 had been given metronidazole, 63 tinidazole, and two albendazole. Due to the study design, consistent follow-up of treatment was not done. Nevertheless, 11 patients (seven given metronidazole and four tinidazole) provided a second fecal sample containing *Giardia* parasites after treatment. All three genes were investigated in samples taken before and after treatment, which revealed two patients with assemblage A, (MLG AII-1 or MLG AII-2 respectively), eight with assemblage B, and one with a mixed assemblage A+B infection. The sample provided after treatment by the patient with mixed infection was positive only for assemblage B. Both patients with assemblage A had been infected in Sweden, whereas the patient with mixed assemblage A+B and six of the patients with assemblage B had been infected in India. The remaining assemblage B patients were infected in Ecuador and Gambia, respectively.

## Discussion

To the best of our knowledge, this is the first large-scale study of *Giardia* infections to use both sequence-based multilocus genotyping and assemblage-specific PCR to determine associations between symptoms and assemblages and to investigate transmission dynamics and possible zoonotic transmission. Most of the *Giardia* infections we studied (69%) were acquired abroad, although domestic infections were not infrequent (31%). Assemblage B was found in 128 patients (60%) and assemblage A in 73 (34%). Mixed assemblage A+B infections were identified in six patients (3%) while samples from seven patients (3%) could not be amplified in PCR. Assemblage B dominated in infections acquired in Sweden (64%) as well as in those originating from most other parts of the world, which agrees with previous reports from Europe, Latin America, Asia, and Australia [13], [22], [34]–[36], although studies from Brazil and Egypt have instead shown a predominance of assemblage A [37], [38]. It has been suggested that the higher parasite excretion in assemblage B-infections as detected by microscopy [39] or real-time PCR [13] could explain the predominance of this assemblage in certain areas. Clearly, further research is needed to explore this issue.

Several studies have reported correlations between assemblages and symptoms, but there has been a lack of concordance in the data obtained [12]–[16], [40]. We compared symptoms and assemblages in 138 patients with pure assemblage A or B infections, and the only apparent correlation was that flatulence was significantly more common in children aged 0–5 years who were infected with assemblage B parasites. *Blastocystis* was detected in samples from many (14%) of our patients, but the clinical significance of this organism is controversial, and the occurrence had no effect on the outcome of the analyses (data not shown). In 11 family clusters, the members shared the same *Giardia* MLGs, but, interestingly, only some of the individuals developed symptoms. This indicates that attention should be given to other factors than the genotypes of parasites such as the patients' immune status, parasite infective dose, age, nutritional status, underlying systemic diseases, and presence of co-infections.

Recent studies using either conventional PCR with assemblage-specific primers [18] or real time PCR [41] have shown a much larger degree of mixed-assemblage infections in humans than was previously reported, which further complicates the attribution of symptoms to infection with a specific assemblage. In our investigation, the combined results of RFLP, sequencing, and use of assemblage-specific primers yielded only six patients (3%) with mixed-assemblage infections, which contradicts the above-mentioned findings but agrees with another recent study that also employed assemblage-specific *tpi* primers [34]. These six patients were all infected outside Europe (one in Africa and five in Asia), in countries where *Giardia* transmission is much higher, and they were all symptomatic. However, five of them were co-infected with bacteria or other parasites, which underlines the difficulty of correlating *Giardia* genotypes with symptoms in cases of infection originating from endemic areas (Table 2).

Since 1979, the WHO has regarded giardiasis as a zoonotic disease, and many studies have suggested zoonotic transmission of *Giardia*, although this has never been proven conclusively. It has been proposed that multilocus genotyping is a powerful tool for investigating possible zoonotic transmission [11], [17], and we used this strategy in the present study of human isolates and in a previous investigation of Swedish animal isolates [20]. In the current study, it seemed that most patients with assemblage A infection had acquired their parasites through the anthroponotic route, since they harbored sub-assemblage AII, which is rarely found in animals [11]. However, three patients who were diagnosed with symptomatic giardiasis in November 2008 and came from the same area north of Stockholm carried two different assemblage A MLGs previously detected in cats and ruminants in Sweden (Table 3, Fig. 2A) [20]. Two of these patients did not report any particular contact with animals prior to infection, and, notably, they were the only patients infected with assemblage A that were hospitalized and treated with intravenous fluids during the study period. The third patient was apparently a moose and deer hunter who was responsible for dismembering the animal carcasses and whose hunting expedition took place shortly before he became symptomatic. None of these patients had traveled outside their home area prior to infection, thus domestic zoonotic transmission is strongly indicated.

Few assemblage B isolates have been found in animals in Sweden, and not many investigators have conducted multilocus genotyping of assemblage B in isolates from animals [19], [42], hence it is difficult to compare human and animal isolates in this context. Two studies of the ß-giardin locus in animal isolates have detected assemblage B subtypes identical to those that were most common in our study (i.e., B1-1 and B1-3) [43], [44]. However, findings concerning only one gene are not sufficient to compare different isolates, which stresses the need for multilocus genotyping of more B isolates, from both humans and animals.

Eighteen family clusters were identified during the study period. Six of these were connected with assemblage A, and the families were infected with MLG AII-1 or MLG AII-2, or a combination of both. It should be mentioned that the resolution of assemblage A was limited, since these two MLGs predominated throughout the study (Table 3). Markers with higher discriminatory power are needed to render multilocus genotyping useful in outbreaks involving *Giardia* assemblage A. In five out of eight clusters where assemblage B parasites were identified, patients connected with each other carried the same assemblage B MLGs (Table 4), which indicates that in some instances it might be possible to use assemblage B MLGs for source tracing in outbreak situations. However, the frequent occurrence of overlapping nucleotides in assemblage B sequences in one, two, or all three genes examined limited the value of MLGs from assemblage B, because these could be determined in only 31 isolates exhibiting sequences without heterogeneous positions in all three genes (Table 4). In these isolates, there was good concordance between sub-assemblages BIII and BIV at the *tpi* and *gdh* loci, but otherwise the separation of isolates into sub-assemblages BIII or BIV was greatly affected by overlap occurring at the specific nucleotide positions that can supposedly distinguish between these subgroups [33] (Supplementary Tables S2A–C). This implies that determining BIII and BIV is of limited value due to widespread occurrence of heterogeneous positions in the sequences. The polymorphism of assemblage B was also reflected in the RFLPs of this assemblage, which sometimes were difficult to interpret due to unusual patterns caused by the presence of alternate nucleotides at the restriction sites (Fig. 1). A high degree of polymorphism in assemblage B has also been observed in other studies [17], [22], [26], [45] and has been further investigated by cloning [46]–[48]. This feature has been attributed to mixed subtype infections or allelic sequence divergence, or a combination of both. In the present study, isolates for which all three genes produced double peaks in the chromatogram were strongly correlated with infections acquired outside Europe (p = <0.001), suggesting that mixed subtype infections are more common in areas with high prevalence of *Giardia*. Almost all assemblage A sequences, except five with mixed subtype A2 and A3 at the β-giardin locus, displayed unequivocal nucleotide sequences at all three loci. The level of allelic sequence divergence was found to be higher in a recent whole-genome sequencing of the assemblage B isolate GS [49] compared to that observed in assemblage A isolate WB [50], which agrees with the larger number of double peaks for assemblage B sequences.

There was good agreement between assignment of assemblages at all three loci, and no assemblage swapping (i.e., different assemblages at different loci in the same isolate) was detected in any of the isolates exhibiting single-assemblage infections at all three genes, as determined by PCR-RFLP and sequencing. Assemblage swapping has been reported by other investigators [9], [24], [36] and has been attributed to recombination between assemblages or mixed assemblage infection. We identified six mixed assemblage infections and these can potentially be recombinants or fusions between assemblage A and B parasites but a serial dilution of the templates removed the minor assemblage, arguing against recombinat isolates (data not shown). Intra-assemblage recombination is more likely to occur than inter-assemblage recombination, since assemblage A and B isolates are only 78% identical at the nucleotide level. However, intra-assemblage recombination is more difficult to detect but our data in Table 4 shows a mixed pattern of alleles, which can be the result of recombination between different assemblage B subtypes. Preliminary results from our group have revealed that overlapping nucleotides occur in discriminatory positions in the chromatograms of single *Giardia* cysts or trophozoites obtained from assemblage B isolates (data not shown). This demonstrates that there are different subtype-specific alleles in one cell, which further strengthens the suggestion that recombination occurs within assemblages.

Treatment failure was suspected in 11 patients (two with assemblage A, eight with assemblage B, and one with mixed assemblage infection) who showed persistence of parasites in a second fecal sample provided after treatment. Comparison of sequence data obtained before and after treatment was possible in 10 of those subjects. Notably, the patient with mixed assemblage A+B infection harbored only assemblage B parasites after treatment. The isolates from the eight patients with assemblage B infection had almost identical sequences before and after treatment, although the results varied slightly due to frequent double peaks in the chromatograms. None of these patients shared the exact same sequences at all loci, thus their *Giardia* isolates could not be attributed to any specific combination of assemblage B sequences. The present study was not designed to conduct systematic post-treatment follow-ups, and thus the 11 patients with suspected treatment failure were detected incidentally, and no additional data could be collected. Anecdotal reports concerning treatment failure in patients with assemblage B acquired in India have indicated that well-planned studies are needed to address this issue.

We performed multilocus genotyping of the three protein-coding genes ß-giardin, *tpi*, and *gdh* combined with analysis using assemblage-specific *tpi* primers, which at present might be the best choice for *Giardia* genotyping. However, it was difficult to compare our results with the findings of other investigations due to the lack of standardization of PCR primers and protocols, the extensive polymorphisms in assemblage B in the studied genes combined with inconsistent interpretation of sequence chromatograms, and no uniform nomenclature of subtypes and MLGs. Hopefully, the addition of new markers and thorough testing of a large number of reference strains and isolates from various sources using of the same loci and the same primer sets will aid the development of a more uniform, standardized, and informative approach to *Giardia* genotyping.

Most of the *Giardia* infections investigated here originated abroad, although the proportion of domestic infections was by no means insignificant. Even though most of our patients appeared to be infected by anthroponotic transmission, we found evidence that also zoonotic transmission of *Giardia* occurs in Sweden, since the same assemblage A MLGs previously detected in Swedish cats and ruminants were found in a few human domestic cases. The only correlation observed between symptoms and assemblages was that flatulence was more common in children 0–5 years of age infected with assemblage B. Mixed-assemblage infections were uncommon. Assemblage B was found more often than assemblage A in both travelers and domestic cases, and it was also more common in patients with suspected treatment failure. Determination of assemblage A and B MLGs proved to be a useful tool that can be used in outbreak situations or to demonstrate possible zoonotic potential. However, the extensive polymorphism seen in assemblage B hampered the determination of MLGs, because only isolates without ambiguous positions could be included.

## Supporting Information

Figure S1 Nucleotide maximum likelihood trees based on ß-giardin (A), *gdh* (B), and *tpi* (C) gene sequences. All unique unambiguous sequences from Table 4 are included together with reference isolates and isolates from our previous MLG study of animals in Sweden [20] (Supplementary Table S1). The color coding is the same as for the clustering into BIII (red) and BIV (blue) isolates shown in Figure 2. The trees are based on 475, 393, and 490 aligned positions, respectively. Only bootstrap support values >50 are shown.
(EPS)

File S1 β-giardin sequences from 120 isolates.
(DOC)

File S2 Gdh sequences from 120 isolates.
(DOC)

File S3 Tpi sequences from 120 isolates.
(DOC)

Table S1 (DOC)

Table S2 (DOC)
