# Supplementary material for: Multilocus Genotyping of Human Giardia Isolates Suggests Limited Zoonotic Transmission and Association between Assemblage B and Flatulence in Children
Source: PLoS Negl Trop Dis. 2011 Aug 2;5(8):e1262. doi: 10.1371/journal.pntd.0001262 (PMC3149019; doi:10.1371/journal.pntd.0001262)
Supplement: File S1 — β-giardin sequences from 120 isolates. (DOC) [file pntd.0001262.s002.doc]

Supplementary file S1. β-giardin sequences from 120 isolates

>Sweh001

AGGTCCGCCGCGTCGACGACGACACGCGTGTGAAGATGATCAAGGACGCCATCGCACACCTTGACAGACTCATCCAGACAGAGTCGAGGAAGCGCCAGGCCTCGTTCGAGGACATCCGCGAGGAAGTCAAGAAGTCTGCCGACAACATGTACCTGACGATCAAGGAGGAGATCGACACCATGGCCGCAAACTTCCGCAAGTCTCTTGCTGAGATGGGCGATACGCTCAACAACGTCGAGACGAACCTCCAGAACCAGATCGCCATCCACAACGACGCCATCGCAGCCCTTAGGAAGGAGGCCCTCAAGAGCCTGAACGACCTCGAGACAGGCATCGCCACGGAGAACGCCGAGAGGAAGAAGATGTACGACCAGCTCAACGAGAAAGTCGCAGAGGGCTTCGCCCGCATCTCCGCTGCCATCGAGAAGGAGACGATCGCCCGCGAGAGGGCCGTCAGCGCCGCCACGACAGAGGCCCTCACAAACACGAA

>Sweh005

AGGTCCGCCGCGTCGACGACGACACGCGTGTAAAGATGATCAAGGACGCCATCGCGCACCTCGACAGACTCATCCAGACGGAGTCGAGGAAGCGCCAGGCCTCGTTCGAGGACATYCGCGAGGAAGTCAAGAAGTCTGCCGACAACATGTACCTGACGATCAAGGAGGAGATCGACACCATGGCCGCAAACTTCCGCAAGTCTCTYGCTGAGATGGGCGACACGCTCAACAACGTCGAGACGAACCTCCAGAACCAGATCGCYATCCACAACGACGCCATCGCAGCCCTCAGGAAGGAGGCCCTCAAGAGCCTGAACGACCTCGAGACAGGCATCGCCACGGAGAACGCCGAGAGGAAGAAGATGTATGACCAGCTCAACGAGAAAGTCGCAGAGGGCTTCGCCCGCATCTCCGCTGCCATCGAGAAGGAGACGATCGCCCGCGAGAGGGCCGTCAGCGCCGCCACGACAGAGGCCCTCACAAACACGA

>Sweh006

AGGTCCGCCGCGTCGACGACGACACGCGTGTGAAGATGATCAAGGACGCCATCGCGCACCTYGACAGACTCATCCAGACRGAGTCGAGGAAGCGCCAGGCCTCGTTCGAGGACATCCGCGAGGARGTCAAGAAGTCTGCCGACAACATGTACCTGACGATCAAGGAGGAGATCGACACCATGGCCGCAAACTTCCGCAAGTCTCTCGCTGAGATGGGCGACACGCTCAACAACGTCGAGACGAACCTCCAGAACCAGATCGCCATCCACAACGACGCCATCGCAGCCCTCAGGAAGGAGGCCCTCAAGAGCCTGAACGACCTCGAGACAGGCATCGCCACGGAGAACGCCGAGAGGAAGAAGATGTATGACCAGCTCAACGAGAAAGTCGCAGAGGGCTTCGCCCGCATCTCCGCTGCCATCGAGAAGGAGACGATCGCCCGCGAGAGGGCCGTCAGCGCCGCCACGACAGAGGCCCTCACAAACAC

>Sweh007

AGGTCCGCCGCGTCGACGACGACACGCGTGTGAAGATGATCAAGGACGCCATCGCGCACCTCGACAGACTCATCCAGACGGAGTCGAGGAAGCGCCAGGCCTCGTTCGAGGACATCCGCGAGGAAGTCAAGAAGTCCGCCGACAACATGTACCTGACGATCAAGGAGGAGATCGACACCATGGCCGCAAACTTCCGCAAGTCTCTCGCTGAGATGGGCGACACGCTCAACAACGTCGAGACGAACCTCCAGAACCAGATCGCCATTCACAACGACGCCATCGCAGCCCTCAGGAAGGAGGCCCTCAAGAGCCTGAACGACCTCGAGACAGGCATCGCCACGGAGAACGCCGAGAGGAAGAAGATGTATGACCAGCTCAACGAGAAAGTCGCAGAGGGCTTCGCCCGCATCTCCGCTGCCATCGAGAAGGAGACGATCGCCCGCGAGAGGGCCGTCAGCGCCGCCACGACAGAGGCCCTCACAAACACGA-

>Sweh008

~GGTCCGCCGCGTCGACGACGACACGCGTGTGAAGATGATCAAGGACGCCATCGCGCACCTCGACAGACTCATCCAGACRGAGTCGAGGAAGCGCCAGGCCTCGTTCGAGGACATCCGCGAGGAAGTCAAGAAGTCTGCCGACAACATGTACCTGACGATCAAGGAGGAGATCGACACCATGGCCGCAAACTTCCGCAAGTCTCTCGCTGAGATGGGCGACACGCTCAACAACGTCGAGACGAACCTCCAGAACCAGATCGCCATCCACAACGACGCCaTCGCAGCCCTCAGGAAGGAGGCCCTCAAGAGCCTGAACGACCTCGAGACAGGCATCGCCACGGAGAACGCCGAGAGGAAGAAGATGTATGACCAGCTCAACGAGAAAGTCGCAGAGGGCTTCGCCCGCATCTCCGCTGCCATCGAGAAGGAGACGATCGCCCGCGAGAGGGCCGTCAGCGCCGCCACGACAGAGGCCCTCACAAACACGA

>Sweh009

AGGTCCGCCGCGTCGACGACGACACGCGTGTGAAGATGATCAAGGACGCCATCGCGCACCTYGACAGACTCATCCAGACAGAGTCGAGGAAGCGCCAGGCCTCGTTCGAGGACATCCGCGAGGAAGTCAAGAAGTCTGCCGACAACATGTACCTGACGATCAAGGAGGAGATCGACACCATGGCCGCAAACTTCCGCAAGTCTCTCGCTGAGATGGGCGACACGCTCAACAACGTCGAGACGAACCTCCAGAACCAGATCGCCATCCACAACGACGCCATCGCAGCCCTCAGGAAGGAGGCCCTCAAGAGCCTGAACGACCTYGAGACAGGCATCGCCACGGAGAACGCCGAGAGGAAGAAGATGTATGACCAGCTCAACGAGAAAGTCGCAGAGGGCTTCGCCCGCATCTCCGCYGCCATCGAGAAGGAGACGATCGCCCGCGAGAGGGCCGTYAGCGCCGCCACGACAGAGGCCCTCACAAACAC---

>Sweh010

-------CCGCGTCGACGACGACACGCGTGTGAAGATGATCAAGGACGCCATCGCGCACCTYGACAGACTCATCCAGACAGAGTCGAGGAAGCGCCAGGCCTCGTTCGAGGACATCCGCGAGGAAGTCAAGAAGTCTGCCGACAACATGTACCTGACGATCAAGGAGGAGATCGACACCATGGCCGCAAACTTCCGCAAGTCTCTCGCTGAGATGGGCGACACGCTCAACAACGTCGAGACGAACCTCCAGAACCAGATCGCCATCCACAACGACGCCATCGCAGCCCTCAGGAAGGAGGCCCTCAAGAGCCTGAACGACCTCGARACRGGCATCGCCACGGAGAACGCCGAGAGGAAGAAGATGTATGACCAGCTCAACGAGAAAGTCGCAGAGGGCTTCGCCCGCATCTCMGCTGCCATCGAGAAGGAGACGATCGCCCGCGAGAGGGCCGTCAGCGCCGCCACGACAGAGGCCCTCACA--------

>Sweh011

-------CCGCGTCGACGACGACACGCGTGTGAAGATGATCAAGGACGCCATCGCGCACCTCGACAGACTCATCCAGACAGAGTCGAGGAAGCGCCAGGCCTCGTTCGAGGACATCCGCGAGGAAGTCAAGAAGTCTGCCGACAACATGTACCTGACGATCAAGGAGGAGATCGACACCATGGCCGCAAACTTCCGCAAGTCTCTCGCTGAGATGGGCGACACGCTCAACAACGTCGAGACGAACCTCCAGAACCAGATCGCCATCCACAACGACGCCATCGCAGCCCTCAGGAAGGAGGCCCTCAAGAGCCTGAACGACCTCGARACRGGCATCGCCACGGAGAACGCCGAGAGGAAGAAGATGTATGACCAGCTCAACGAGAAAGTCGCAGAGGGCTTCGCCCGCATCTCMGCTGCCATCGAGAAGGAGACGATCGCCCGCGAGAGGGCCGTCAGCGCCGCCACGACAGAGGCCCTCACA--------

>Sweh013

--GTCCGCCGCGTCGACGACGACACGCGTGTGAAGATGATCAAGGACGCCATCGCGCACCTCGACAGACTCATCCAGACGGAGTCGAGGAAGCGCCAGGCCTCGTTCGAGGACATCCGCGAGGAAGTCAAGAAGTCTGCCGACAACATGTACCTGACGATCAAGGAGGAGATCGACACCATGGCCGCAAACTTCCGCAAGTCTCTCGCTGAGATGGGCGACACGCTCAACAACGTCGAGACGAACCTCCAGAACCAGGTCGCCATCCACAACGACGCCATCGCAGCCCTCAGGAAGGAGGCCCTCAAGAGCCTGAACGACCTCGAGACAGGCATCGCCACGGAGAACGCCGAGAGGAAGAAGATGTATGACCAGCTCAACGAGAAAGTCGCAGAGGGCTTCGCCCGCATCTCCGCTGCCATCGAGAAGGAGACGATCGCCCGCGAGAGGGCCGTCAGCGCCGCCACGACAGAGGCCCTCACAAACACGA-

>Sweh014

AGGTCCGCCGCGTCGACGACGACACGCGTGTGAAGATGATCAAGGACGCCATCGCGCACCTCGACAGACTCATCCAGACAGAGTCGAGGAAGCGCCAGGCCTCGTTCGAGGACATCCGCGAGGARGTCAAGAAGTCTGCCGACAACATGTACCTGACGATCAAGGAGGAGATCGACACCATGGCCGCAAACTTCCGCAAGTCTCTCGCTGAGATGGGCGACACGCTCAACAACGTCGAGACGAACCTCCAGAACCAGATCGCCATCCACAACGACGCCATCGCAGCCCTCAGGAAGGAGGCCCTCAAGAGCCTGAACGACCTCGAGACAGGCATCGCCACGGAGAACGCCGAGAGGAAGAAGATGTATGACCAGCTCAACGAGAAAGTCGCAGAGGGCTTCGCCCGCATCTCCGCTGCCATCGAGAAGGAGACGATCGCYCGCGAGAGGGCCGTCAGCGCCGCCACGACAGAGGCCCTCACAAACACGA

>Sweh015

AGGTCCGCCGCGTCGACGACGACACGCGTGTGAAGATGATCAAGGACGCCATCGCGCACCTCGACAGACTCATCCAGACAGAGTCGAGGAAGCGCCAGGCCTCGTTCGAGGACATCCGCGAGGAAGTCAAGAAGTCTGCCGACAACATGTACCTGACGATCAAGGAGGAGATCGACACCATGGCCGCAAACTTCCGCAAGTCTCTYGCTGAGATGGGCGACACGCTCAACAACGTCGAGACGAACCTCCAGAACCAGATCGCCATCCACAACGACGCCATCGCAGCCCTCAGGAAGGAGGCCCTCAAGAGCCTGAACGACCTCGAGACAGGCATCGCCACGGAGAACGCCGAGAGGAAGAAGATGTATGACCAGCTCAACGAGAAAGTCGCAGAGGGCTTCGCCCGCATCTCCGCTGCCATCGAGAAGGAGACGATCGCCCGCGAGAGGGCCGTCAGCGCCGCCACGACAGAGGCCCTCACAAACACGAA

>Sweh019

AGGTCCGCCGCGTCGACGACGACACGCGTGTGAAGATGATCAAGGACGCCATCGCGCACCTYGACAGACTCATCCAGACAGAGTCGAGGAAGCGCCAGGCCTCGTTCGAGGACATCCGCGAGGAAGTCAAGAAGTCTGCCGACAACATGTACCTGACGATCAAGGAGGAGATCGACACCATGGCCGCAAACTTCCGCAAGTCTCTYGCTGAGATGGGCGACACGCTCAACAACGTCGAGACGAACCTCCAGAACCAGATCGCCATCCACAACGACGCCATCGCAGCCCTCAGGAAGGAGGCCCTCAAGAGCCTGAACGACCTCGAGACAGGCATCGCCACGGAGAACGCCGAGAGGAAGAAGATGTATGACCAGCTCAACGAGAAAGTCGCAGAGGGCTTCGCCCGCATCTCCGCTGCCATCGAGAAGGAGACGATCGCCCGCGAGAGGGCCGTCAGCGCCGCCACGACAGAGGCCCTCACAAACACGA

>Sweh021

-GGTCCGCCGCGTCGACGACGACACGCGTGTGAAGATGATCAAGGACGCCATCGCACACCTTGACAGACTCATCCAGACAGAGTCGAGGAAGCGCCAGGCCTCGTTCGAGGACATCCGCGAGGAAGTCAAGAAGTCTGCCGACAACATGTACCTGACGATCAAGGAGGAGATCGACACCATGGCCGCAAACTTCCGCAAGTCTCTTGCTGAGATGGGCGATACGCTCAACAACGTCGAGACGAACCTCCAGAACCAGATCGCCATCCACAACGACGCCATCGCAGCCCTTAGGAAGGAGGCCCTCAAGAGCCTGAACGACCTCGAGACAGGCATCGCCACGGAGAACGCCGAGAGGAAGAAGATGTACGACCAGCTCAACGAGAAAGTCGCAGAGGGCTTCGCCCGCATCTCCGCTGCCATCGAGAAGGAGACGATCGCCCGCGAGAGGGCCGTCAGCGCCGCCACGACAGAGGCCCTCACAAACACGAA

>Sweh022

AGGTCCGCCGCGTCGACGACGACACGCGTGTGAAGATGATCAAGGACGCCATCGCACACCTTGACAGACTCATCCAGACAGAGTCGAGGAAGCGCCAGGCCTCGTTCGAGGACATCCGCGAGGAAGTCAAGAAGTCTGCCGACAACATGTACCTGACGATCAAGGAGGAGATCGACACCATGGCCGCAAACTTCCGCAAGTCTCTTGCTGAGATGGGCGATACGCTCAACAACGTCGAGACGAACCTCCAGAACCAGATCGCCATCCACAACGACGCCATCGCAGCCCTTAGGAAGGAGGCCCTCAAGAGCCTGAACGACCTCGAGACAGGCATCGCCACGGAGAACGCCGAGAGGAAGAAGATGTACGACCAGCTCAACGAGAAAGTCGCAGAGGGCTTCGCCCGCATCTCCGCTGCCATCGAGAAGGAGACGATCGCCCGCGAGAGGGCCGTCAGCGCCGCCACGACAGAGGCCCTCACAAACACGAA

>Sweh023

-GGTCCGCCGCGTCGACGACGACACGCGTGTGAAGATGATCAAGGACGCCATCGCGCACCTCGACAGACTCATCCAGACGGAGTCGAGGAAGCGCCAGGCCTCGTTCGAGGACATCCGCGAGGAAGTCAAGAAGTCTGCCGACAACATGTACCTGACGATCAAGGAGGAGATCGACACCATGGCCGCAAACTTCCGCAAGTCTCTTGCTGAGATGGGCGACACGCTCAACAACGTCGAGACGAACCTCCAGAACCAGATCGCCATCCACAACGACGCCATCGCAGCCCTCAGGAAGGAGGCCCTCAAGAGCCTGAACGACCTCGAGACAGGCATCGCCACGGAGAACGCCGAGAGGAAGAAGATGTATGACCAGCTCAACGAGAAAGTCGCAGAGGGCTTCGCCCGCATCTCCGCCGCCATCGAGAAGGAGACGATCGCCCGCGAGAGGGCCGTCAGCGCTGCCACGACAGAGGCCCTCACAAACACG--

>Sweh025

~GGTCCGCCGCGTCGACGACGACACGCGTGTGAAGATGATCAAGGACGCCATYGCGCACCTYGACAGACTCATCCAGACAGAGTCGAGGAAGCGCCAGGCCTCGTTCGAGGACATCCGCGAGGARGTCAAGAAGTCTGCCGACAACATGTACCTGACGATCAAGGAGGAGATCGACACCATGGCCGCAAACTTCCGCAAGTCTCTTGCTGAGATGGGCGACACGCTCAACAACGTCGAGACGAACCTCCAGAACCAGATCGCCATCCACAACGACGCCATCGCAGCCCTCAGGAAGGAGGCCCTCAAGAGCCTGAACGACCTCGAGACAGGCATCGCCACGGAGAACGCCGAGAGGAAGAAGATGTATGACCAGCTCAACGAGAAAGTCGCAGAGGGCTTCGCCCGCATCTCCGCTGCCATCGAGAAGGAGACGATCGCCCGCGAGAGGGCCGTCAGCGCCGCCACGACAGAGGCCCTYACAAACACGAA

>Sweh027

AGGTCCGCCGCGTCGACGACGACACGCGTGTGAAGATGATCAAGGACGCCATCGCGCACCTCGACAGACTCATCCAGACAGAGTCGAGGAAGCGCCAGGCCTCGTTCGAGGACATCCGCGAGGAAGTCAAGAAGTCTGCCGACAACATGTACCTGACGATCAAGGAGGAGATCGACACCATGGCCGCAAACTTCCGCAAGTCTCTCGCTGAGATGGGCGACACGCTCAACAACGTCGAGACGAACCTCCAGAACCAGATCGCCATCCACAACGACGCCATCGCAGCCCTCAGGAAGGAGGCCCTCAAGAGCCTGAACGACCTCGAGACAGGSATCGCCACGGARAACGCCGAGAGGAAGAAGATGTATGACCAGCTCAACGAGAAAGTCGCAGAGGGCTTCGCCCGCATCTCCGCTGCCATCGAGAAGGAGACGATCGCCCGCGAGAGGGCCGTCAGCGCCGCCACGACAGAGGCCCTCACAAACACGAA

>Sweh028

AGGTCCGCCGCGTCGACGACGACACGCGTGTGAAGATGATCAAGGACGCCATCGCGCACCTYGACAGACTCATCCAGACAGAGTCGAGGAAGCGCCAGGCCTCGTTCGAGGACATCCGCGAGGARGTCAAGAAGTCTGCCGACAACATGTACCTGACGATCAAGGAGGAGATCGACACCATGGCCGCAAACTTCCGCAAGTCTCTYGCTGAGATGGGCGACACGCTCAACAACGTCGAGACGAACCTCCAGAACCAGATCGCCRTCCACAACGACGCCATCGCAGCCCTCAGGAAGGAGGCCCTCAAGAGCCTGAACGACCTCGAGACAGGCATCGCCACGGAGAACGCCGAGAGGAAGAAGATGTATGACCAGCTCAACGAGAAAGTCGCAGAGGGCTTCGCCCGCATCTCCGCTGCCATCGAGAAGGAGACGATCGCCCGCGAGAGGGCCGTCAGCGCCGCCACGACAGAGGCCCTCACAAACACGAA

>Sweh033

---TCCGCCGCGTCGACGACGACACGCGTGTGAAGATGATCAAGGACGCCATCGCGCACCTTGACAGACTCATCCAGACAGAGTCGAGGAAGCGCCAGGCCTCGTTCGAGGACATCCGCGAGGAAGTCAAGAAGTCTGCCGACAACATGTACCTGACGATCAAGGAGGAGATCGACACCATGGCCGCAAACTTCCGCAAGTCTCTTGCTGAGATGGGCGACACGCTCAACAACGTCGAGACGAACCTCCAGAACCAGATCGCCATCCACAACGACGCCATCGCAGCCCTCAGGAAGGAGGCCCTCAAGAGCCTGAACGACCTCGAGACAGGCATCGCCACGGAGAACGCCGAGAGGAAGAAGATGTATGACCAGCTCAACGAGAAAGTCGCAGAGGGCTTCGCCCGCATCTCCGCTGCCATCGAGAAGGAGACGATCGCCCGCGAGAGGGCCGTCAGCGCCGCCACGACAGAGGCCCTCACAAACACGAA

>Sweh034

~~~~~~~~~~~~~~~~~~~~~~~~~~~~~~~~~AGATGATCaAGGACGCCATCGCGCACCTTGACAGACTCATCCAGACAGAGTCGAGGAAGCGCCAGGCCTCGTTCGAGGACATCCGCGAGGAAGTCAAGAAGTCTGCCGACAACATGTACCTGACGATCAAGGAGGAGATCGACACCATGGCCGCAAACTTCCGCAAGTCTCTYGCTGAGATGGGCGACACGCTCAACAACGTCGAGACGAACCTCCAGAACCAGATCGCCATCCACAACGACGCCATCGCAGCCCTCAGGAAGGAGGCCCTCAAGAGCCTGAACGACCTCGAGACAGGCATCGCCACGGAGAACGCCGAGAGGAAGAAGATGTATGACCAGCTCAACGAGAAAGTCGCAGAGGGCTTCGCCCGCATCTCCGCTGCCATCGAGAAGGAGACGATCGCCCGCGAGAGGGCCGTCAGCGCCGCCACGACAGAGGCCCTCACAAACACGAA

>Sweh035

AGGTCCGCCGCGTCGACGACGACACGCGTGTGAAGATGATCAAGGACGCCATCGCGCACCTCGACAGACTCATCCAGACAGAGTCGAGGAAGCGCCAGGCCTCGTTCGAGGACATCCGCGAGGAAGTCAAGAAGTCTGCCGACAACATGTACCTGACGATCAAGGAGGAGATCGACACCATGGCCGCAAACTTCCGCAAGTCTCTYGCTGAGATGGGCGACACGCTCAACAACGTCGAGACGAACCTCCAGAACCAGATCGCCATCCACAACGACGCCATCGCAGCCCTCAGGAAGGAGGCCCTCAAGAGCCTGAACGACCTYGAGACAGGCATCGCCACGGAGAACGCCGAGAGGAAGAAGATGTATGACCAGCTCAACGAGAAAGTCGCAGAGGGCTTCGCCCGCATCTCCGCTGCCATCGAGAAGGAGACGATCGCCCGCGAGAGGGCCGTCAGCGCCGCCACGACAGAGGCCCTCACAAACACGAA

>Sweh039

-----CGCCGCGTCGACGACGACACGCGTGTGAAGATGATCAAGGACGCCATCGCGCACCTCGACAGACTCATCCAGACGGAGTCGAGGAAGCGCCAGGCCTCGTTCGAGGACATCCGCGAGGAAGTCAAGAAGTCTGCCGACAACATGTACCTGACGATCAAGGAGGAGATCGACACCATGGCCGCAAACTTCCGCAAGTCTCTTGCTGAGATGGGCGACACGCTCAACAACGTCGAGACGAACCTCCAGAACCAGATCGCCATCCACAACGACGCCATCGCAGCCCTCAGGAAGGAGGCCCTCAAGAGCCTGAAYGACCTCGAGACAGGCATCGCCACGGAGAACGCCGAGAGGAAGAAGATGTATGACCAGCTCAACGAGAAAGTCGCAGAGGGCTTCGCCCGCATCTCCGCCGCCATCGAGAAGGAGACGATCGCCCGCGAGAGGGCCGTCAGCGCTGCCACGACAGAGGCCCTCACAAACACGAA

>Sweh041

AGGTCCGCCGCGTCGACGACGACACGCGTGTGAAGATGATCAAGGACGCCATCGCACACCTTGACAGACTCATCCAGACAGAGTCGAGGAAGCGCCAGGCCTCGTTCGAGGACATCCGCGAGGAAGTCAAGAAGTCTGCCGACAACATGTACCTGACGATCAAGGAGGAGATCGACACCATGGCCGCAAACTTCCGCAAGTCTCTTGCTGAGATGGGCGATACGCTCAACAACGTCGAGACGAACCTCCAGAACCAGATCGCCATCCACAACGACGCCATCGCAGCCCTTAGGAAGGAGGCCCTCAAGAGCCTGAACGACCTCGAGACAGGCATCGCCACGGAGAACGCCGAGAGGAAGAAGATGTACGACCAGCTCAACGAGAAAGTCGCAGAGGGCTTCGCCCGCATCTCCGCTGCCATCGAGAAGGAGACGATCGCCCGCGAGAGGGCCGTCAGCGCCGCCACGACAGAGGCCCTCACAAACACGAA

>Sweh042

AGGTCCGCCGCGTCGACGACGACACGCGTGTGAAGATGATCAAGGACGCCATCGCGCACCTTGACAGACTCATCCAGACAGAGTCGAGGAAGCGCCAGGCCTCGTTCGAGGACATCCGCGAGGAAGTCAAGAAGTCTGCCGACAACATGTACCTGACGATCAAGGAGGAGATCGACACCATGGCCGCAAACTTCCGCAAGTCTCTTGCTGAGATGGGCGACACGCTCAACAACGTCGAGACGAACCTCCAGAACCAGATCGCCATCCACAACGACGCCATCGCAGCCCTTAGGAAGGAGGCCCTCAAGAGCCTGAACGACCTCGAGACAGGCATCGCCACGGAGAACGCCGAGAGGAAGAAGATGTATGACCAGCTCAACGAGAAAGTCGCAGAGGGCTTCGCCCGCATCTCCGCTGCCATCGAGAAGGAGACGATCGCCCGCGAGAGGGCCGTCAGCGCCGCCACGACAGAGGCCCTCACAAACACGAA

>Sweh043

---TCCGCCGCGTCGACGACGACACGCGTGTGAAGATGATCAAGGACGCCATCGCGCACCTCGACAGACTCATCCAGACAGAGTCGAGGAAGCGCCAGGCCTCGTTCGAGGACATCCGCGAGGAAGTCAAGAAGTCTGCCGACAACATGTACCTGACGATCAAGGAGGAGATTGACACCATGGCCGCAAACTTCCGCAAGTCTCTTGCTGAGATGGGCGACACGCTCAACAACGTCGAGACGAACCTCCAGAACCAGATCGCCATCCACAACGACGCCATCGCAGCCCTCAGGAAGGAGGCCCTCAAGAGCCTGAACGACCTCGAGACAGGCATCGCCACGGAGAACGCCGAGAGGAAGAAGATGTATGACCAGCTCAACGAGAAAGTCGCAGAGGGCTTCGCCCGCATCTCCGCTGCCATCGAGAAGGAGACGATCGCCCGCGAGAGGGCCGTCAGCGCCGCCACGACAGAGGCCCTCACAAACACGAA

>Sweh044

----CCGCCGCGTCGACGACGACACGCGTGTGAAGATGATCAAGGACGCCATCGCGCACCTCGACAGACTCATCCAGACAGAGTCGAGGAAGCGCCAGGCCTCGTTCGAGGACATCCGCGAGGAAGTCAAGAAGTCYGCCGACAACATGTACCTGACGATCAAGGAGGAGATCGACACCATGGCCGCAAACTTCCGCAAGTCTCTTGCTGAGATGGGCGACACGCTCAACAACGTCGAGACGAACCTCCAGAACCAGATCGCCATCCACAACGACGCCATCGCAGCCCTCAGGAAGGAGGCCCTCAAGAGCCTGAACGACCTCGAGACAGGCATCGCCACGGAGAACGCCGAGAGGAAGAAGATGTATGACCAGCTCAACGAGAAAGTCGCAGAGGGCTTCGCCCGCATCTCCGCTGCCATCGAGAAGGAGACGATCGCCCGCGAGAGGGCCGTCAGCGCCGCCACGACAGAGGCCCTCACAAACACGAA

>Sweh045

-GGTCCGCCGCGTCGACGACGACACGCGTGTGAAGATGATCAAGGACGCCATCGCGCACCTCGACAGACTCATCCAGACRGAGTCGAGGAAGCGCCAGGCCTCGTTCGAGGACATCCGCGAGGAAGTCAAGAAGTCTGCCGACAACATGTACCTGACGATCAAGGAGGAGATCGACACCATGGCCGCAAACTTCCGCAAGTCTCTYGCTGAGATGGGCGACACGCTCAACAACGTCGAGACGAACCTCCAGAACCAGATCGCCATCCACAACGACGCCATCGCAGCCCTCAGGAAGGAGGCCCTCAAGAGCCTGAACGACCTCGAGACAGGCATCGCCACGGAGAACGCCGAGAGGAAGAAGATGTATGACCAGCTCAACGAGAAAGTCGCAGAGGGCTTCGCCCGCATCTCCGCYGCCATCGAGAAGGAGACGATCGCCCGCGAGAGGGCCGTCAGCGCYGCCACGACAGAGGCCCTCACAAACACGAA

>Sweh047

AGGTCCGCCGCGTCGACGACGACACGCGTGTGAAGATGATCAAGGACGCCATCGCGCACCTTGACAGACTCATCCAGACAGAGTCGAGGAAGCGCCAGGCCTCGTTCGAGGACATCCGCGAGGAAGTCAAGAAGTCTGCCGACAACATGTACCTGACGATCAAGGAGGAGATCGACACCATGGCCGCAAACTTCCGCAAGTCTCTTGCTGAGATGGGCGATACGCTCAACAACGTCGAGACGAACCTCCAGAACCAGATCGCCATCCACAACGACGCCATCGCAGCCCTTAGGAAGGAGGCCCTCAAGAGCCTGAACGACCTCGAGACAGGCATCGCCACGGAGAACGCCGAGAGGAAGAAGATGTACGACCAGCTCAACGAGAAAGTCGCAGAGGGCTTCGCCCGCATCTCCGCTGCCATCGAGAAGGAGACGATCGCCCGCGAGAGGGCCGTCAGCGCCGCCACGACAGAGGCCCTCACAAACACGAA

>Sweh048

----CCGCCGCGTCGACGACGACACGCGTGTGAAGATGATCAAGGACGCCATCGCGCACCTTGACAGACTCATCCAGACAGAGTCGAGGAAGCGCCAGGCCTCGTTCGAGGACATCCGCGAGGAAGTCAAGAAGTCTGCCGACAACATGTACCTGACGATCAAGGAGGAGATCGACACCATGGCCGCAAACTTCCGCAAGTCTCTTGCTGAGATGGGCGATACGCTCAACAACGTCGAGACGAACCTCCAGAACCAGATCGCCATCCACAACGACGCCATCGCAGCCCTTAGGAAGGAGGCCCTCAAGAGCCTGAACGACCTCGAGACAGGCATCGCCACGGAGAACGCCGAGAGGAAGAAGATGTACGACCAGCTCAACGAGAAAGTCGCAGAGGGCTTCGCCCGCATCTCCGCTGCCATCGAGAAGGAGACGATCGCCCGCGAGAGGGCCGTCAGCGCCGCCACGACAGAGGCCCTCACAAACACGAA

>Sweh049

AGGTCCGCCGCGTCGACGACGACACGCGTGTGAAGATGATCAAGGACGCCATCGCGCACCTTGACAGACTCATCCAGACAGAGTCGAGGAAGCGCCAGGCCTCGTTCGAGGACATCCGCGAGGAAGTCAAGAAGTCTGCCGACAACATGTACCTGACGATCAAGGAGGAGATCGACACCATGGCCGCAAACTTCCGCAAGTCTCTTGCTGAGATGGGCGATACGCTCAACAACGTCGAGACGAACCTCCAGAACCAGATCGCCATCCACAACGACGCCATCGCAGCCCTTAGGAAGGAGGCCCTCAAGAGCCTGAACGACCTCGAGACAGGCATCGCCACGGAGAACGCCGAGAGGAAGAAGATGTACGACCAGCTCAACGAGAAAGTCGCAGAGGGCTTCGCCCGCATCTCCGCTGCCATCGAGAAGGAGACGATCGCCCGCGAGAGGGCCGTCAGCGCCGCCACGACAGAGGCCCTCACAAACACGAA

>Sweh051

--GTCCGCCGCGTCGACGACGACACGCGTGTGAAGATGATCAAGGACGCCATCGCACACCTTGACAGACTCATCCAGACAGAGTCGAGGAAGCGCCAGGCCTCGTTCGAGGACATCCGCGAGGAAGTCAAGAAGTCTGCCGACAACATGTACCTGACGATCAAGGAGGAGATCGACACCATGGCCGCAAACTTCCGCAAGTCTCTTGCTGAGATGGGCGATACGCTCAACAACGTCGAGACGAACCTCCAGAACCAGATCGCCATCCACAACGACGCCATCGCAGCCCTTAGGAAGGAGGCCCTCAAGAGCCTGAACGACCTCGAGACAGGCATCGCCACGGAGAACGCCGAGAGGAAGAAGATGTACGACCAGCTCAACGAGAAAGTCGCAGAGGGCTTCGCCCGCATCTCCGCTGCCATCGAGAAGGAGACGATCGCCCGCGAGAGGGCCGTCAGCGCCGCCACGACAGAGGCCCTCACAAACACGAA

>Sweh056

AGGTCCGCCGCGTCGACGACGACACGCGTGTGAAGATGATCAAGGACGCCATCGCACACCTTGACAGACTCATCCAGACAGAGTCGAGGAAGCGCCAGGCCTCGTTCGAGGACATCCGCGAGGAAGTCAAGAAGTCTGCCGACAACATGTACCTGACGATCAAGGAGGAGATCGACACCATGGCCGCAAACTTCCGCAAGTCTCTTGCTGAGATGGGCGATACGCTCAACAACGTCGAGACGAACCTCCAGAACCAGATCGCCATCCACAACGACGCCATCGCAGCCCTTAGGAAGGAGGCCCTCAAGAGCCTGAACGACCTCGAGACAGGCATCGCCACGGAGAACGCCGAGAGGAAGAAGATGTACGACCAGCTCAACGAGAAAGTCGCAGAGGGCTTCGCCCGCATCTCCGCTGCCATCGAGAAGGAGACGATCGCCCGCGAGAGGGCCGTCAGCGCCGCCACGACAGAGGCCCTCACAAACACGAA

>Sweh057

AGGTCCGCCGCGTCGACGACGACACGCGTGTGAAGATGATCAAGGACGCCATCGCACACCTTGACAGACTCATCCAGACAGAGTCGAGGAAGCGCCAGGCCTCGTTCGAGGACATCCGCGAGGAAGTCAAGAAGTCTGCCGACAACATGTACCTGACGATCAAGGAGGAGATCGACACCATGGCCGCAAACTTCCGCAAGTCTCTTGCTGAGATGGGCGATACGCTCAACAACGTCGAGACGAACCTCCAGAACCAGATCGCCATCCACAACGACGCCATCGCAGCCCTTAGGAAGGAGGCCCTCAAGAGCCTGAACGACCTCGAGACAGGCATCGCCACGGAGAACGCCGAGAGGAAGAAGATGTACGACCAGCTCAACGAGAAAGTCGCAGAGGGCTTCGCCCGCATCTCCGCTGCCATCGAGAAGGAGACGATCGCCCGCGAGAGGGCCGTCAGCGCCGCCACGACAGAGGCCCTCACAAACACGAA

>Sweh058

--GTCCGCCGCGTCGACGACGACACGCGTGTGAAGATGATCAAGGACGCCATCGCACACCTTGACAGACTCATCCAGACAGAGTCGAGGAAGCGCCAGGCCTCGTTCGAGGACATCCGCGAGGAAGTCAAGAAGTCTGCCGACAACATGTACCTGACGATCAAGGAGGAGATCGACACCATGGCCGCAAACTTCCGCAAGTCTCTTGCTGAGATGGGCGATACGCTCAACAACGTCGAGACGAACCTCCAGAACCAGATCGCCATCCACAACGACGCCATCGCAGCCCTTAGGAAGGAGGCCCTCAAGAGCCTGAACGACCTCGAGACAGGCATCGCCACGGAGAACGCCGAGAGGAAGAAGATGTACGACCAGCTCAACGAGAAAGTCGCAGAGGGCTTCGCCCGCATCTCCGCTGCCATCGAGAAGGAGACGATCGCCCGCGAGAGGGCCGTCAGCGCCGCCACGACAGAGGCCCTCACAAACACGAA

>Sweh059

AGGTCCGCCGCGTCGACGACGACACGCGTGTGAAGATGATCAAGGACGCCATCGCACACCTTGACAGACTCATCCAGACAGAGTCGAGGAAGCGCCAGGCCTCGTTCGAGGACATCCGCGAGGAAGTCAAGAAGTCTGCCGACAACATGTACCTGACGATCAAGGAGGAGATCGACACCATGGCCGCAAACTTCCGCAAGTCTCTTGCTGAGATGGGCGATACGCTCAACAACGTCGAGACGAACCTCCAGAACCAGATCGCCATCCACAACGACGCCATCGCAGCCCTTAGGAAGGAGGCCCTCAAGAGCCTGAACGACCTCGAGACAGGCATCGCCACGGAGAACGCCGAGAGGAAGAAGATGTACGACCAGCTCAACGAGAAAGTCGCAGAGGGCTTCGCCCGCATCTCCGCTGCCATCGAGAAGGAGACGATCGCCCGCGAGAGGGCCGTCAGCGCCGCCACGACAGAGGCCCTCACAAACACGAA

>Sweh060

AGGTCCGCCGCGTCGACGACGACACGCGTGTGAAGATGATCAAGGACGCCATCGCGCACCTCGACAGACTCATCCAGACGGAGTCGAGGAAGCGCCAGGCCTCGTTCGAGGACATCCGCGAGGAAGTCAAGAAGTCTGCCGACAACATGTACCTGACGATCAAGGAGGAGATCGACACCATGGCCGCAAACTTCCGCAAGTCTCTTGCTGAGATGGGCGACACGCTCAACAACGTCGAGACGAACCTCCAGAACCAGATCGCCATCCACAACGACGCCATCGCAGCCCTCAGGAAGGAGGCCCTCAAGAGCCTGAACGACCTCGAGACAGGCATCGCCACGGAGAACGCCGAGAGGAAGAAGATGTATGACCAGCTCAACGAGAAAGTCGCAGAGGGCTTCGCCCGCATCTCCGCTGCCATCGAGAAGGAGACGATCGCCCGCGAGAGGGCCGTCAGCGCCGCCACGACAGAGGCCCTCACAAACACGAA

>Sweh062

AGGTCCGCCGCGTCGACGACGACACGCGTGTGAAGATGATCAAGGACGCCATCGCGCACCTCGACAGACTCATCCAGACAGAGTCGAGGAAGCGCCAGGCCTCGTTCGAGGACATCCGCGAGGAAGTCAAGAAGTCTGCCGACAACATGTACCTGACGATCAAGGAGGAGATCGACACCATGGCCGCAAACTTCCGCAAGTCTCTTGCTGAGATGGGCGACACGCTCAACAACGTCGAGACGAACCTCCAGAACCAGATCGCCATCCACAACGACGCCATCGCAGCCCTCAGGAAGGAGGCCCTCAAGAGCCTGAACGACCTCGAGACAGGCATCGCCACGGAGAACGCCGAGAGGAAGAAGATGTATGACCAGCTCAACGAGAAAGTCGCAGAGGGCTTCGCCCGCATCTCCGCTGCCATCGAGAAGGAGACGATCGCCCGCGAGAGGGCCGTCAGCGCCGCCACGACAGAGGCCCTCACAAACACGAA

>Sweh064

AGGTCCGCCGCGTCGACGACGACACGCGTGTGAAGATGATCAAGGACGCCATCGCGCACCTCGACAGACTCATCCAGACAGAGTCGAGGAAGCGCCAGGCCTCGTTCGAGGACATCCGCGAGGARGTCAAGAAGTCTGCCGACAACATGTACCTGACGATCAAGGAGGAGATCGACACCATGGCCGCAAACTTCCGCAAGTCTCTTGCTGAGATGGGCGACACGCTCAACAACGTCGAGACGAACCTCCAGAACCAGATCGCCATCCACAACGACGCCATCGCAGCCCTCAGGAAGGAGGCCCTCAAGAGCCTGAACGACCTCGAGACAGGCATCGCCACGGAGAACGCCGAGAGGAAGAAGATGTATGACCAGCTCAACGAGAAAGTCGCAGAGGGCTTCGCCCGCATCTCCGCTGCCATCGAGAAGGAGACGATCGYCCGCGAGAGGGCCGTCAGCGCCGCCACGACAGAGGCCCTCACAAACACG

>Sweh066

-GGTCCGCCGCGTCGACGACGACACGCGTGTGAAGATGATCAAGGACGCCATCGCGCACCTCGACAGACTCATCCAGACAGAGTCGAGGAAGCGCCAGGCCTCGTTCGAGGACATCCGCGAGGAAGTCAAGAAGTCTGCCGACAACATGTACCTGACRATCAAGGAGGAGATCGACACCATGGCCGCAAACTTCCGCAAGTCTCTYGCTGAGATGGGCGACACGCTCAACAACGTCGAGACGAACCTCCAGAACCAGATCGCCATCCACAACGACGCCATCGCAGCCCTCAGGAAGGAGGCCCTCAAGAGCCTGAACGACCTCGAGACAGGCATCGCCACGGARAACGCCGAGAGGAAGAAGATGTATGACCAGCTCAACGAGAAAGTCGCAGAGGGCTTCGCCCGCATCTCCGCTGCCATCGAGAAGGAGACGATCGCCCGCGAGAGGGCCGTCAGCGCCGCCACGACAGAGGCCCTCACAAACACGAA

>Sweh067

-GGTCCGCCGCGTCGACGACGACACGCGTGTGAAGATGATCAAGGACGCCATCGCACACCTTGACAGACTCATCCAGACAGAGTCGAGGAAGCGCCAGGCCTCGTTCGAGGACATCCGCGAGGAAGTCAAGAAGTCTGCCGACAACATGTACCTGACGATCAAGGAGGAGATCGACACCATGGCCGCAAACTTCCGCAAGTCTCTTGCTGAGATGGGCGATACGCTCAACAACGTCGAGACGAACCTCCAGAACCAGATCGCCATCCACAACGACGCCATCGCAGCCCTTAGGAAGGAGGCCCTCAAGAGCCTGAACGACCTCGAGACAGGCATCGCCACGGAGAACGCCGAGAGGAAGAAGATGTACGACCAGCTCAACGAGAAAGTCGCAGAGGGCTTCGCCCGCATCTCCGCTGCCATCGAGAAGGAGACGATCGCCCGCGAGAGGGCCGTCAGCGCCGCCACGACAGAGGCCCTCACAAACACGAA

>Sweh068

AGGTCCGCCGCGTCGACGACGACACGCGTGTGAAGATGATCAAGGACGCCATCGCGCACCTYGACAGACTCATCCAGACRGAGTCGAGGAAGCGCCAGGCCTCGTTCGAGGACATCCGCGAGGARGTCAAGAAGTCTGCCGACAACATGTACCTGACGATCAAGGAGGAGATCGACACCATGGCCGCAAACTTCCGCAAGTCTCTYGCTGAGATGGGCGACACGCTCAACAACGTCGAGACGAACCTCCAGAACCAGATCGCCATCCACAACGACGCCATCGCAGCCCTCAGGAAGGAGGCCCTCAAGAGCCTGAACGACCTCGAGACAGGCATCGCCACGGAGAACGCCGAGAGGAAGAAGATGTATGACCAGCTCAACGAGAAAGTCGCAGAGGGCTTCGCCCGCATCTCCGCTGCCATCGAGAAGGAGACGATCGCYCGCGAGAGGGCCGTCAGCGCCGCCACGACAGAGGCCCTCACAAACACGAA

>Sweh069

~~~TCCGCCGCGTCGACGACGACACGCGTGTGAAGATGATCAAGGACGCCATCGCGCACCTYGACAGACTCATCCAGACRGAGTCGAGGAAGCGCCAGGCCTCGTTCGAGGACATCCGCGAGGAAGTCAAGAAGTCTGCCGACAACATGTACCTGACGATCAAGGAGGAGATCGACACCATGGCCGCAAACTTCCGCAAGTCTCTYGCTGAGATGGGCGACACGCTCAACAACGTCGAGACGAACCTCCAGAACCAGATCGCCATCCACAACGACGCCATCGCAGCCCTCAGGAARGAGGCCCTCAAGAGCCTGAACGACCTCGAGACAGGCATCGCCACGGAGAACGCCGAGAGGAAGAAGATGTATGACCAGCTCAACGAGAAAGTCGCAGAGGGCTTCGCCCGCATCTCCGCYGCCATCGAGAAGGAGACGATCGCYCGCGAGAGGGCCGTCAGCGCYGCCACGACAGAGGCCCTCACAAACACGAA

>Sweh074

AGGTCCGCCGCGTCGACGACGACACGCGTGTGAAGATGATCAAGGACGCCATCGCGCACCTTGACAGACTCATCCAGACAGAGTCGAGGAAGCGCCAGGCCTCGTTCGAGGACATCCGCGAGGAAGTCAAGAAGTCTGCCGACAACATGTACCTGACGATCAAGGAGGAGATCGACACCATGGCCGCAAACTTCCGCAAGTCTCTTGCTGAGATGGGCGACACGCTCAACAACGTCGAGACGAACCTCCAGAACCAGATCGCCATCCACAACGACGCCATCGCAGCCCTTAGGAAGGAGGCCCTCAAGAGCCTGAACGACCTCGAGACAGGCATCGCCACGGAGAACGCCGAGAGGAAGAAGATGTATGACCAGCTCAACGAGAAAGTCGCAGAGGGCTTCGCCCGCATCTCCGCTGCCATCGAGAAGGAGACGATCGCCCGCGAGAGGGCCGTCAGCGCCGCCACGACAGAGGCCCTCACAAACACGAA

>Sweh075

-------CCGCGTCGACGACGACACGCGTGTGAAGATGATCAAGGACGCCATCGCGCACCTCGACAGACTCATCCAGACAGAGTCGAGGAAGCGCCAGGCCTCGTTCGAGGACATCCGCGAGGAAGTCAAGAAGTCTGCCGACAACATGTACCTGACGATCAAGGAGGAGATCGACACCATGGCCGCAAACTTCCGCAAGTCTCTYGCTGAGATGGGCGACACGCTCAACAACGTCGAGACGAACCTCCAGAACCAGATCGCCATCCACAACGACGCCATCGCAGCCCTCAGGAAGGAGGCCCTCAAGAGCCTGAACGACCTCGAGACAGGCATCGCCACGGAGAACGCCGAGAGGAAGAAGATGTATGACCAGCTCAACGAGAAAGTCGCAGAGGGCTTCGCCCGCATCTCMGCTGCCATCGAGAAGGAGACGATCGCCCGCGAGAGGGCCGTCAGCGCCGCCACGACAGAGGCCCTCACAAACACGAA

>Sweh076

AGGTCCGCCGCGTCGACGACGACACGCGTGTGAAGATGATCaAGGACGCCATYGCGCACCTCGACAGACTCATCCAGACAGAGTCGAGGAAGCGCCAGGCCTCGTTCGAGGACATCCGCGAGGAAGTCAAGAAGTCTGCCGACAACATGTACCTGACGATCAAGGAGGAGATCGACACCATGGCCGCAAACTTCCGCAAGTCTCTCGCTGAGATGGGCGACACGCTCAACAACGTCGAGACGAACCTCCAGAACCAGATCGCCATCCACAACGACGCCATCGCAGCCCTCAGGAAGGAGGCCCTCAAGAGCCTGAACGACCTCGAGACAGGCATCGCCACGGAGAACGCCGAGAGGAAGAAGATGTATGACCAGCTCAACGAGAAAGTCGCAGAGGGCTTCGCCCGCATCTCCGCTGCCATCGAGAAGGAGACGATCGCCCGCGAGAGGGCCGTCAGCGCCGCCACGACAGAGGCCCTCACAAACACGAA

>Sweh079

~~~TCCGCCGCGTCGACGACGACACGCGTGTGAAGATGATCAAGGACGCCATCGCGCACCTCGACAGACTCATCCAGACRGAGTCGAGGAAGCGCCAGGCCTCGTTCGAGGACATYCGCGAGGAAGTCAAGAAGTCTGCCGACAACATGTACCTGACGATCAAGGAGGAGATCGACACCATGGCCGCAAACTTCCGCAAGTCTCTyGCTGAGATGGGCGACACGCTCAACAACGTCGAGACGAACCTCCAGAACCAGATCGCCATCCACAACGACGCCATCGCAGCCCTCAGGAAGGAGGCCCTCAAGAGCCTGAACGACCTCGAGACAGGCATCGCCACGGAGAACGCCGAGAGGAAGAAGATGTATGACCAGCTCAACGAGAAAGTCGCAGAGGGCTTCGCCCGCATCTCCGCYGCCATCGAGAAGGAGACGATCGCCCGCGAGAGGGCCGTCAGCGCYGCCACGACAGAGGCCCTCACAAACACGAA

>Sweh081

AGGTCCGCCGCGTCGACGACGACACGCGTGTGAAGATGATCAAGGACGCCATCGCGCACCTTGACAGACTCATCCAGACAGAGTCGAGGAAGCGCCAGGCCTCGTTCGAGGACATCCGCGAGGAAGTCAAGAAGTCTGCCGACAACATGTACCTGACGATCAAGGAGGAGATCGACACCATGGCCGCAAACTTCCGCAAGTCTCTCGCTGAGATGGGCGACACGCTCAACAACGTCGAGACGAACCTCCAGAACCAGATCGCCATCCACAACGACGCCATCGCAGCCCTCAGGAAGGAGGCCCTCAAGAGCCTGAACGACCTCGAGACAGGCATCGCCACGGAGAACGCCGAGAGGAAGAAGATGTATGACCAGCTCAACGAGAAAGTCGCAGAGGGCTTCGCCCGCATCTCCGCTGCCATCGAGAAGGAGACGATCGCCCGCGAGAGGGCCGTCAGCGCCGCCACGACAGAGGCCCTCACAAACACGAA

>Sweh082

----CCGCCGCGTCGACGACGACACGCGTGTGAAGATGATCAAGGACGCCATCGCGCACCTCGACAGACTCATCCAGACAGAGTCGAGGAAGCGCCAGGCCTCGTTCGAGGACATCCGCGAGGAAGTCAAGAAGTCTGCCGACAACATGTACCTGACGATCAAGGAGGAGATCGACACCATGGCCGCAAACTTCCGCAAGTCTCTTGCTGAGATGGGCGAYACGCTCAACAACGTCGAGACGAACCTCCAGAACCAGATCGCCATCCACAACGACGCCATCGCAGCCCTCAGGAAGGAGGCCCTCAAGAGCCTGAACGACCTCGAGACAGGCATCGCCACGGAGAACGCCGAGAGGAAGAAGATGTATGACCAGCTCAACGAGAAAGTCGCAGAGGGCTTCGCCCGCATCTCCGCTGCCATCGAGAAGGAGACGATCGCCCGCGAGAGGGCCGTCAGCGCCGCCACGACAGAGGCCCTCACAAACACGAA

>Sweh083

-GGTCCGCCGCGTCGACGACGACACGCGTGTGAAGATGATCAAGGACGCCATCGCGCACCTTGACAGACTCATCCAGACAGAGTCGAGGAAGCGCCAGGCCTCGTTCGAGGACATCCGCGAGGAAGTCAAGAAGTCTGCCGACAACATGTACCTGACGATCAAGGAGGAGATCGACACCATGGCCGCAAACTTCCGCAAGTCTCTTGCTGAGATGGGCGATACGCTCAACAACGTCGAGACGAACCTCCAGAACCAGATCGCCATCCACAACGACGCCATCGCAGCCCTTAGGAAGGAGGCCCTCAAGAGCCTGAACGACCTCGAGACAGGCATCGCCACGGAGAACGCCGAGAGGAAGAAGATGTACGACCAGCTCAACGAGAAAGTCGCAGAGGGCTTCGCCCGCATCTCCGCTGCCATCGAGAAGGAGACGATCGCCCGCGAGAGGGCCGTCAGCGCCGCCACGACAGAGGCCCTCACAAACACGAA

>Sweh084

-GGTCCGCCGCGTCGACGACGACACGCGTGTGAAGATGATCAAGGACGCCATCGCGCACCTTGACAGACTCATCCAGACAGAGTCGAGGAAGCGCCAGGCCTCGTTCGAGGACATCCGCGAGGAAGTCAAGAAGTCTGCCGACAACATGTACCTGACGATCAAGGAGGAGATCGACACCATGGCCGCAAACTTCCGCAAGTCTCTTGCTGAGATGGGCGACACGCTCAACAACGTCGAGACGAACCTCCAGAACCAGATCGCCATCCACAACGACGCCATCGCAGCCCTCAGGAAGGAGGCCCTCAAGAGCCTGAACGATCTCGAGACAGGCATCGCCACGGAGAATGCCGAGAGGAAGAAGATGTATGACCAGCTCAACGAGAAAGTCGCAGAGGGCTTCGCCCGCATCTCCGCTGCCATCGAGAAGGAGACGATCGCCCGCGAGAGGGCCGTCAGCGCCGCCACGACAGAGGCCCTCACAAACAC---

>Sweh086

AGGTCCGCCGCGTCGACGACGACACGCGTGTGAAGATGATCAAGGACGCCATCGCGCACCTYGACAGACTCATCCAGACRGAGTCGAGGAAGCGCCAGGCCTCGTTCGAGGACATCCGCGAGGAAGTCAAGAAGTCTGCCGACAACATGTACCTGACGATCAAGGAGGAGATCGACACCATGGCCGCAAACTTCCGCAAGTCTCTTGCTGAGATGGGCGACACGCTCAACAACGTCGAGACGAACCTCCAGAACCAGATCGCCATCCACAACGACGCCATCGCAGCCCTCAGGAAGGAGGCCCTCAAGAGCCTGAACGAYCTCGAGACAGGCATCGCCACGGAGAACGCCGAGAGGAAGAAGATGTATGACCAGCTCAACGAGAAAGTCGCAGAGGGCTTCGCCCGCATCTCCGCYGCCATCGAGAAGGAGACGATCGCYCGCGAGAGRGCCGTCAGCGCYGCCACGACAGAGGCCCTCACAAACACGAA

>Sweh088

AGGTCCGCCGCGTCGACGACGACACGCGTGTGAAGATGATCAAGGACGCCATCGCGCACCTCGACAGACTCATCCAGACAGAGTCGAGGAAGCGCCAGGCCTCGTTCGAGGACATCCGCGAGGAAGTCAAGAAGTCTGCCGACAACATGTACCTGACGATCAAGGAGGAGATCGACACCATGGCCGCAAACTTCCGCAAGTCTCTCGCTGAGATGGGCGACACGCTCAACAACGTCGAGACGAACCTCCAGAACCAGATCGCCATCCACAACGATGCCATCGCAGCCCTCAGGAAGGAGGCCCTCAAGAGCCTGAACGACCTCGAGACAGGCATCGCCACGGAGAACGCCGAGAGGAAGAAGATGTATGACCAGCTCAACGAGAAAGTCGCAGAGGGCTTCGCCCGCATCTCCGCTGCCATCGAGAAGGAGACGATCGCCCGCGAGAGGGCCGTCAGCGCCGCCACGACAGAGGCCCTCACAAACACGA-

>Sweh089

---TCCGCCGCGTCGACGACGACACGCGTGTGAAGATGATCAAGGACGCCATCGCGCACCTCGACAGACTCATCCAGACAGAGTCGAGGAAGCGCCAGGCCTCGTTCGAGGACATCCGCGAGGAAGTCAAGAAGTCTGCCGACAACATGTACCTGACGATCAAGGAGGAGATCGACACCATGGCCGCAAACTTCCGCAAGTCTCTYGCTGAGATGGGCGACACGCTCAACAACGTCGAGACGAACCTCCAGAACCAGATCGCCATCCACAACGACGCCATCGCAGCCCTCAGGAAGGAGGCCCTCAAGAGCCTGAACGACCTCGAGACAGGCATCGCCACGGAGAACGCCGAGAGGAAGAAGATGTATGACCAGCTCAACGAGAAAGTCGCAGAGGGCTTCGCCCGCATCTCCGCTGCCATCGAGAAGGAGACGATCGCCCGCGAGAGGGCCGTCAGCGCCGCCACGACAGAGGCCCTCACAAACACGAA

>Sweh090

AGGTCCGCCGCGTCGACGACGACACGCGTGTGAAGATGATCAAGGACGCCATCGCGCACCTYGACAGACTCATCCAGACAGAGTCGAGGAAGCGCCAGGCCTCGTTCGAGGACATCCGCGAGGARGTCAAGAAGTCYGCCGACAACATGTACCTGACGATYAAGGAGGAGATCGACACCATGGCCGCAAACTTCCGCAAGTCTCTYGCTGAGATGGGCGACACGCTCAACAACGTCGAGACGAACCTCCAGAACCAGATCGCCATCCACAACGACGCCATCGCAGCCCTCAGGAAGGAGGCCCTCAAGAGCCTGAACGACCTCGAGACAGGCATCGCCACGGAGAAYGCCGAGAGGAAGAAGATGTATGACCAGCTCAACGAGAAAGTCGCAGAGGGCTTCGCCCGCATCTCCGCTGCCATCGAGAAGGAGACGATCGCCCGCGAGAGGGCCGTCAGCGCCGCCACGACAGAGGCCCTCACAAACACGA

>Sweh091

AGGTCCGCCGCGTCGACGACGACACGCGTGTGAAGATGATCAAGGACGCCATCGCGCACCTYGACAGACTCATCCAGACAGAGTCGAGGAAGCGCCAGGCCTCGTTCGAGGACATCCGCGAGGAAGTCAAGAAGTCTGCCGACAACATGTACCTGACGATCAAGGAGGAGATCGACACCATGGCCGCAAACTTCCGCAAGTCTCTYGCTGAGATGGGCGACACGCTCAACAACGTCGAGACGAAYCTCCAGAACCAGATCGCCATCCACAACGACGCCATCGCAGCCCTCAGGAAGGAGGCCCTCAAGAGCCTGAACGACCTCGAGACAGGCATCGCCACGGAGAACGCCGAGAGGAAGAAGATGTATGACCAGCTCAACGAGAAAGTCGCAGAGGGCTTCGCYCGCATCTCCGCTGCCATCGAGAAGGAGACGATCGCCCGCGAGAGGGCCGTCAGCGCCGCCACGACAGAGGCCCTCACAAACACGAA

>Sweh092

AGGTCCGCCGCGTCGACGACGACACGCGTGTGAAGATGATCAAGGACGCCATCGCGCACCTYGACAGACTCATCCAGACAGAGTCGAGGAAGCGCCAGGCCTCGTTCGAGGACATCCGCGAGGAAGTCAAGAAGTCTGCCGACAACATGTACCTGACGATCAAGGAGGAGATCGACACCATGGCCGCAAACTTCCGCAAGTCTCTTGCTGAGATGGGCGACACGCTCAACAACGTCGAGACGAACCTCCAGAACCAGATCGCCATCCACAACGACGCCATCGCAGCCCTCAGGAAGGAGGCCCTCAAGAGCCTGAACGACCTCGAGACAGGCATCGCCACGGAGAAYGCCGAGAGGAAGAAGATGTATGACCAGCTCAACGAGAAAGTCGCAGAGGGCTTCGCCCGCATCTCCGCTGCCATCGAGAAGGAGACGATCGCCCGCGAGAGGGCCGTCAGCGCCGCCACGACAGAGGCCCTCACAAACACGAA

>Sweh093

AGGTCCGCCGCGTCGACGACGACACGCGTGTGAAGATGATCAAGGACGCCATCGCGCACCTYGACAGACTCATCCAGACAGAGTCGAGGAAGCGCCAGGCCTCGTTCGAGGACATCCGCGAGGAAGTCAAGAAGTCTGCCGACAACATGTACCTGACGATCAAGGAGGAGATCGACACCATGGCCGCAAACTTCCGCAAGTCTCTYGCTGAGATGGGCGACACGCTCAACAACGTCGAGACGAACCTCCAGAACCAGATCGCCATCCACAACGACGCCATCGCAGCCCTCAGGAAGGAGGCCCTCAAGAGCCTGAACGACCTCGAGACAGGCATCGCCACGGAGAACGCCGAGAGGAAGAAGATGTATGACCAGCTCAACGAGAAAGTCGCAGAGGGCTTCGCCCGCATCTCCGCYGCCATCGAGAAGGAGACGATCGCCCGCGAGAGGGCCGTCAGCGCCGCCACGACAGAGGCCCTCACAAACACGAA

>Sweh094

AGGTCCGCCGCGTCGACGACGACACGCGTGTGAAGATGATCAAGGACGCCATCGCGCACCTCGACAGACTCATCCAGACGGAGTCGAGGAAGCGCCAGGCCTCGTTCGAGGACATCCGCGAGGAAGTCAAGAAGTCTGCCGACAACATGTACCTGACGATCAAGGAGGAGATCGACACCATGGCCGCAAACTTCCGCAAGTCTCTTGCTGAGATGGGCGACACGCTCAACAACGTCGAGACGAACCTCCAGAACCAGATCGCCATCCACAACGACGCCATCGCAGCCCTCAGGAAGGAGGCCCTCAAGAGCCTGAACGACCTCGAGACAGGCATCGCCACGGAGAACGCCGAGAGGAAGAAGATGTATGACCAGCTCAACGAGAAAGTCGCAGAGGGCTTCGCCCGCATCTCCGCCGCCATCGAGAAGGAGACGATCGCCCGCGAGAGGGCCGTCAGCGCTGCCACGACAGAGGCCCTCACAAACACGAA

>Sweh095

AGGTCCGCCGCGTCGACGACGACACGCGTGTGAAGATGATCAAGGACGCCATCGCGCACCTCGACAGACTCATCCAGACAGAGTCGAGGAAGCGCCAGGCCTCGTTCGAGGACATCCGCGAGGAAGTCAAGAAGTCTGCCGACAACATGTACCTGACGATCAAGGAGGAGATCGACACCATGGCCGCAAACTTCCGCAAGTCTCTCGCTGAGATGGGCGACACGCTCAACAACGTCGAGACGAACCTCCAGAACCAGATCGCCATCCACAACGACGCCATCGCAGCCCTCAGGAAGGAGGCCCTCAAGAGCCTGAACGACCTCGAGACAGGCATCGCCACGGAGAACGCCGAGAGGAAGAAGATGTATGACCAGCTCAACGAGAAAGTCGCAGAGGGCTTCGCCCGCATCTCCGCTGCCATCGAGAAGGAGACGATCGCCCGCGAGAGGGCCGTCAGCGCCGCCACGACAGAGGCCCTCACAAACACGAA

>Sweh102

AGGTCCGCCGCGTCGACGACGACACGCGTGTGAAGATGATCAAGGACGCCATCGCGCACCTCGACAGACTCATCCAGACAGAGTCGAGGAAGCGCCAGGCCTCGTTCGAGGACATCCGCGAGGAAGTCAAGAAGTCTGCCGACAACATGTACCTGACGATCAAGGAGGAGATCGACACCATGGCCGCAAACTTCCGCAAGTCTCTCGCTGAGATGGGCGACACGCTCAACAACGTCGAGACGAACCTCCAGAACCARATCGCCATCCACAACGACGCCATCGCAGCCCTCAGGAAGGAGGCCCTCAAGAGCCTGAACGACCTCGAGACAGGCATCGCCACGGAGAACGCCGAGAGGAAGAAGATGTATGACCAGCTCAACGAGAAAGTCGCAGAGGGCTTCGCCCGCATCTCCGCTGCCATCGAGAAGGAGACGATCGCCCGCGAGAGGGCCGTCAGCGCCGCCACGACAGAGGCCCTCACAAACACGAA

>Sweh103

AGGTCCGCCGCGTCGACGACGACACGCGTGTGAAGATGATCAAGGACGCCATCGCGCACCTCGACAGACTCATCCAGACAGAGTCGAGGAAGCGCCAGGCCTCGTTCGAGGACATCCGCGAGGAAGTCAAGAAGTCTGCCGACAACATGTACCTGACGATCAAGGAGGAGATCGACACCATGGCCGCAAACTTCCGCAAGTCTCTCGCTGAGATGGGCGACACGCTCAACAACGTCGAGAYGAACCTCCAGAACCAGATCGCCATCCACAACGACGCCATCGCAGCCCTCAGGAAGGAGGCCCTCAAGAGCCTGAACGACCTCGAGACAGGCATCGCCACGGAGAACGCCGAGAGGAAGAAGATGTATGACCAGCTCAACGAGAAAGTCGCAGAGGGCTTCGCCCGCATCTCCGCTGCCATCGAGAAGGAGACGATCGCCCGCGAGAGGGCCGTCAGCGCCGCCACGACAGAGGCCCTCACAAACACGAA

>Sweh105

AGGTCCGCCGCGTCGACGACGACACGCGTGTGAAGATGATCAAGGACGCCATCGCGCACCTYGACAGACTCATCCAGACAGAGTCGAGGAAGCGCCAGGCCTCGTTCGAGGACATCCGCGAGGAAGTCAAGAAGTCTGCCGACAACATGTACCTGACGATCAAGGAGGAGATYGACACCATGGCCGCAAACTTCCGCAAGTCTCTCGCTGAGATGGGCGACACGCTCAACAACGTCGAGACGAACCTCCAGAACCAGATCGCYATCCACAACGACGCCATCGCAGCCCTCAGGAAGGAGGCCCTCAAGAGCCTGAACGACCTCGAGACAGGCATCGCCACGGAGAACGCCGAGAGGAAGAAGATGTATGACCAGCTCAACGAGAAAGTCGCAGAGGGCTTCGCCCGCATCTCCGCTGCCATCGAGAAGGAGACGATCGCCCGCGAGAGGGCCGTCAGCGCCGCCACGACAGAGGCCCTCACAAACACGAA

>Sweh106

AGGTCCGCCGCGTCGACGACGACACGCGTGTGAAGATGATCAAGGACGCCATCGCGCACCTCGACAGACTCATCCAGACAGAGTCGAGGAAGCGCCAGGCCTCGTTCGAGGACATCCGCGAGGAAGTCAAGAAGTCTGCCGACAACATGTACCTGACGATCAAGGAGGAGATCGACACCATGGCCGCAAACTTCCGCAAGTCTCTTGCTGAGATGGGCGACACGCTCAACAACGTCGAGACGAACCTCCAGAACCAGATCGCCATCCACAACGACGCCATCGCAGCCCTCAGGAAGGAGGCCCTCAAGAGCCTGAACGACCTCGAGACAGGCATCGCCACGGAGAACGCCGAGAGGAAGAAGATGTATGACCAGCTCAACGAGAAAGTCGCAGAGGGCTTCGCCCGCATCTCCGCTGCCATCGAGAAGGAGACGATCGCCCGCGAGAGGGCCGTCAGCGCCGCCACGACAGAGGCCCTCACAAACACGAA

>Sweh107

---TCCGCCGCGTCGACGACGACACGCGTGTGAAGATGATCAAGGACGCCATCGCGCACCTTGACAGACTCATCCAGACAGAGTCGAGGAAGCGCCAGGCCTCGTTCGAGGACATCCGCGAGGAAGTCAAGAAGTCTGCCGACAACATGTACCTGACGATCAAGGAGGAGATCGACACCATGGCCGCAAACTTCCGCAAGTCTCTTGCTGAGATGGGCGACACGCTCAACAACGTCGAGACGAACCTCCAGAACCAGATCGCCATCCACAACGACGCCATCGCAGCCCTCAGGAAGGAGGCCCTCAAGAGCCTGAACGACCTCGAGACAGGCATCGCCACGGAGAACGCCGAGAGGAAGAAGATGTATGACCAGCTCAACGAGAAAGTCGCAGAGGGCTTCGCCCGCATCTCCGCTGCCATCGAGAAGGAGACGATCGCTCGCGAGAGGGCCGTCAGCGCCGCCACGACAGAGGCCCTCACAAACACG--

>Sweh111

AGGTCCGCCGCGTCGACGACGACACGCGTGTGAAGATGATCAAGGACGCCATCGCGCACCTCGACAGACTCATCCAGACGGAGTCGAGGAAGCGCCAGGCCTCGTTCGAGGACATCCGCGAGGAAGTCAAGAAGTCTGCCGACAACATGTACCTGACGATCAAGGAGGAGATCGACACCATGGCCGCAAACTTCCGCAAGTCTCTYGCTGAGATGGGCGACACGCTCAACAACGTCGAGACGAACCTCCAGAACCAGATCGCCATCCACAACGACGCCATCGCAGCCCTCAGGAAGGAGGCCCTCAAGAGCCTGAACGACCTCGAGACAGGCATCGCCACGGAGAACGCCGAGAGGAAGAAGATGTATGACCAGCTCAACGAGAAAGTCGCAGAGGGCTTCGCCCGCATCTCCGCYGCCATCGAGAAGGAGACGATCGCCCGCGAGAGGGCCGTCAGCGCCGCCACGACAGAGGCCCTCACAAACACGAA

>Sweh112

AGGTCCGCCGCGTCGACGACGACACGCGTGTGAAGATGATCAAGGACGCCATCGCGCACCTTGACAGACTCATCCAGACAGAGTCGAGGAAGCGCCAGGCCTCGTTCGAGGACATCCGCGAGGAAGTCAAGAAGTCTGCCGACAACATGTACCTGACGATCAAGGAGGAGATCGACACCATGGCCGCAAACTTCCGCAAGTCTCTTGCTGAGATGGGCGACACGCTCAACAACGTCGAGACGAACCTCCAGAACCAGATCGCCATCCACAACGACGCCATCGCAGCCCTCAGGAAGGAGGCCCTCAAGAGCCTGAACGACCTCGAGACAGGCATCGCCACGGAGAACGCCGAGAGGAAGAAGATGTATGACCAGCTCAACGAGAAAGTCGCAGAGGGCTTCGCCCGCATCTCCGCTGCCATCGAGAAGGAGACGATCGCCCGCGAGAGGGCCGTCAGCGCCGCCACGACAGAGGCCCTCACAAACACGAA

>Sweh113

AGGTCCGCCGCGTCGACGACGACACGCGTGTGAAGATGATCAAGGAYGCCATCGCGCACCTCGACAGACTCATCCAGACRGAGTCGAGGAAGCGCCAGGCCTCGTTCGAGGACATCCGCGAGGAAGTCAAGAAGTCTGCCGACAACATGTACCTGACGATCAAGGAGGAGATCGACACCATGGCCGCAAACTTCCGCAAGTCTCTCGCTGAGATGGGCGACACGCTCAACAACGTCGAGACGAACCTCCAGAACCAGATCGCCATCCACAACGACGCCATCGCAGCCCTCAGGAAGGAGGCCCTCAAGAGCCTGAACGACCTCGAGACAGGCATCGCCACGGAGAACGCCGAGAGGAAGAAGATGTATGACCAGCTCAACGAGAAAGTCGCAGAGGGCTTCGCCCGCATCTCCGCTGCCATCGAGAAGGAGACGATCGCCCGCGAGAGGGCCGTCAGCGCCGCCACGACAGAGGCCCTCACAAACACGAA

>Sweh115

AGGTCCGCCGCGTCGACGACGACACGCGTGTGAAGATGATCAAGGACGCCATCGCGCACCTTGACAGACTCATCCAGACAGAGTCGAGGAAGCGCCAGGCCTCGTTCGAGGACATCCGCGAGGAAGTCAAGAAGTCTGCCGACAACATGTACCTGACGATCAAGGAGGAGATCGACACCATGGCCGCAAACTTCCGCAAGTCTCTYGCTGAGATGGGCGACACRCTCAACAACGTCGAGACGAACCTCCAGAACCAGATCGCCATCCACAACGACGCCATCGCAGCCCTCAGGAAGGAGGCCCTCAAGAGCCTGAACGACCTCGAGACAGGCATCGCCACGGAGAACGCCGAGAGGAAGAAGATGTATGACCAGCTCAACGAGAAAGTCGCAGAGGGCTTCGCCCGCATCTCCGCTGCCATCGAGAAGGAGACGATCGCCCGCGAGAGGGCCGTCAGCGCCGCCACGACAGAGGCCCTCACAAACACGAA

>Sweh116

AGGTCCGCCGCGTCGACGACGACACGCGTGTGAAGATGATCAAGGACGCCATCGCGCACCTCGACAGACTCATCCAGACAGAGTCGAGGAAGCGCCAGGCCTCGTTCGAGGACATCCGCGAGGAAGTCAAGAAGTCTGCCGACAACATGTACCTGACGATCAAGGAGGAGATCGACACCATGGCCGCAAACTTCCGCAAGTCTCTCGCTGAGATGGGCGACACGCTCAACAACGTCGAGACGAACCTCCAGAACCAGATCGCCATCCACAACGACGCCATCGCAGCCCTCAGGAAGGAGGCCCTCAAGAGCCTGAACGACCTCGAGACAGGCATCGCCACGGAGAACGCCGAGAGGAAGAAGATGTATGACCAGCTCAACGAGAAAGTCGCAGAGGGCTTCGCCCGCATCTCCGCTGCCATCGAGAAGGAGACGATCGCCCGCGAGAGGGCCGTCAGCGCCGCCACGACAGAGGCCCTCACAAACACGAA

>Sweh117

----CCGCCGCGTCGACGACGACACGCGTGTGAAGATGATCAAGGACGCCATCGCGCACCTCGACAGACTCATCCAGACGGAGTCGAGGAAGCGCCAGGCCTCGTTCGAGGACATCCGCGAGGAAGTCAAGAAGTCTGCCGACAACATGTACCTGACGATCAAGGAGGAGATCGACACCATGGCCGCAAACTTCCGCAAGTCTCTCGCTGAGATGGGCGACACGCTCAACAACGTCGAGACGAACCTCCAGAACCAGATCGCCATCCACAACGACGCCATCGCAGCCCTCAGGAAGGAGGCCCTCAAGAGCCTGAACGACCTCGAGACAGGCATCGCCACGGAGAACGCCGAGAGGAAGAAGATGTATGACCAGCTCAACGAGAAAGTCGCAGAGGGCTTCGCCCGCATCTCCGCTGCCATCGAGAAGGAGACGATCGCCCGCGAGAGGGCCGTCAGCGCCGCCACGACAGAGGCCCTCACAAACACGAA

>Sweh118

AGGTCCGCCGCGTCGACGACGACACGCGTGTGAAGATGATCAAGGACGCCATCGCGCACCTCGACAGACTCATCCAGACAGAGTCGAGGAAGCGCCAGGCCTCGTTCGAGGACATCCGCGAGGAAGTCAAGAAGTCTGCCGACAACATGTACCTGACGATCAAGGAGGAGATCGACACCATGGCCGCAAACTTCCGCAAGTCTCTYGCTGAGATGGGCGACACGCTCAACAACGTCGAGACGAACCTCCAGAACCAGATCGCCATCCACAACGACGCCATCGCAGCCCTCAGGAAGGAGGCCCTCAAGAGCCTGAACGACCTCGARACRGGCATCGCYACGGAGAACGCCGAGAGGAAGAAGATGTATGACCAGCTCAACGAGAAAGTCGCAGAGGGCTTCGCCCGCATCTCCGCYGCCATCGAGAAGGAGACGATCGCCCGCGAGAGGGCCGTCAGCGCCGCCACGACAGAGGCCCTCACAAACACGAA

>Sweh121

-GGTCCGCCGCGTCGACGACGACACGCGTGTGAAGATGATCAAGGACGCCATCGCGCACCTCGACAGACTCATCCAGACAGAGTCGAGGAAGCGCCAGGCCTCGTTCGAGGACATCCGCGAGGAAGTCAAGAAGTCTGCCGACAACATGTACCTGACGATCAAGGAGGAGATCGACACCATGGCCGCAAACTTCCGCAAGTCTCTCGCTGAGATGGGCGACACGCTCAACAACGTCGAGACGAACCTCCAGAACCAGATCGCCATCCACAACGACGCCATCGCAGCCCTCAGGAAGGAGGCCCTCAAGAGCCTGAACGACCTCGAGACAGGCATCGCCACGGAGAACGCCGAGAGGAAGAAGATGTATGACCAGCTCAACGAGAAAGTCGCAGAGGGCTTCGCCCGCATCTCCGCTGCCATCGAGAAGGAGACGATCGCCCGCGAGAGGGCCGTCAGCGCCGCCACGACGGAGGCCCTCACAAACACGAA

>Sweh123

~~~TCCGCCGCGTCGACGACGACACGCGTGTGAAGATGATCAAGGACGCCATCGCGCACCTYGACAGACTCATCCAGACRGAGTCGAGGAAGCGCCAGGCCTCGTTCGAGGACATCCGCGAGGAAGTCAAGAAGTCYGCCGACAACATGTACCTGACGATCAAGGAGGAGATCGACACCATGGCCGCAAACTTCCGCAAGTCTCTYGCTGAGATGGGCGACACGCTCAACAACGTCGAGACGAACCTCCAGAACCAGATCGCCATCCACAACGACGCCaTCGCAGCCCTCAGGAAGGAGGCCCTCAAGAGCCTGAACGACCTCGAGACAGGCATCGCCACGGAGAACGCCGAGAGGAAGAAGATGTATGACCAGCTCAACGAGAAAGTCGCAGAGGGCTTCGCCCGCATCTCCGCYGCCATCGAGAAGGAGACGATCGCCCGCGAGAGGGCCGTCAGCGCYGCCACGACAGAGGCCCTCACAAACACGAA

>Sweh124

AGGTCCGCCGCGTCGACGACGACACGCGTGTGAAGATGATCAAGGACGCCATCGCGCACCTCGACAGACTCATCCAGACAGAGTCGAGGAAGCGCCAGGCCTCGTTCGAGGACATCCGCGAGGAAGTCAAGAAGTCTGCCGACAACATGTACCTGACGATCAAGGAGGAGATCGACACCATGGCCGCAAACTTCCGCAAGTCTCTCGCTGAGATGGGCGACACGCTCAACAACGTCGAGACGAACCTCCAGAACCAGATCGCCATCCACAACGACGCCATCGCAGCCCTCAGGAAGGAGGCCCTCAAGAGCCTGAACGACCTCGAGACAGGCATCGCCACGGAGAACGCCGAGAGGAAGAAGATGTATGACCAGCTCAACGAGAAAGTCGCAGAGGGCTTCGCCCGCATCTCCGCTGCCATCGAGAAGGAGACGATCGCCCGCGAGAGGGCCGTCAGCGCCGCCACGACAGAGGCCCTCACAAACACGAA

>Sweh126

AGGTCCGCCGCGTCGACGACGACACGCGTGTGAAGATGATCAAGGACGCCATCGCGCACCTCGACAGACTCATCCAGACAGAGTCGAGGAAGCGCCAGGCCTCGTTCGAGGACATCCGCGAGGAAGTCAAGAAGTCTGCCGAYAACATGTACCTGACGATCAAGGAGGAGATCGACACCATGGCCGCAAACTTCCGCAAGTCTCTCGCTGAGATGGGCGACACGCTCAACAACGTCGAGACGAACCTCCAGAACCAGATCGCCATCCACAACGACGCCATCGCAGCCCTCAGGAAGGAGGCCCTCAAGAGCCTGAACGACCTCGAGACAGGCATCGCCACGGAGAACGCCGAGAGGAAGAAGATGTATGACCAGCTCAACGAGAAAGTCGCAGAGGGCTTCGCCCGCATCTCCGCTGCCATCGAGAAGGAGACGATCGCCCGCGAGAGGGCCGTCAGCGCCGCCACGACAGAGGCCCTCACAAACACGAA

>Sweh127

AGGTCCGCCGCGTCGACGACGACACGCGTGTGAAGATGATCAAGGACGCCATCGCGCACCTCGACAGACTCATCCAGACGGAGTCGAGGAAGCGCCAGGCCTCGTTCGAGGACATCCGCGAGGAAGTCAAGAAGTCTGCCGACAACATGTACCTGACGATCAAGGAGGAGATCGACACCATGGCCGCAAACTTCCGCAAGTCTCTYGCTGAGATGGGCGACACGCTCAACAACGTCGAGACGAACCTCCAGAACCAGATCGCCATCCACAACGACGCCATCGCAGCCCTCAGGAAGGAGGCCCTCAAGAGCCTGAACGACCTCGAGACAGGCATCGCCACGGAGAACGCCGAGAGGAAGAAGATGTATGACCAGCTCAACGAGAAAGTCGCAGAGGGCTTCGCCCGCATCTCCGCYGCCATCGAGAAGGAGACGATCGCCCGCGAGAGGGCCGTCAGCGCCGCCACGACAGAGGCCCTCACAAACACGAA

>Sweh128

~GGTCCGCCGCGTCGACGACGACACGCGTGTGAAGATGATCAAGGACGCCATCGCGCACCTYGACAGACTCATCCAGACAGAGTCGAGGAAGCGCCAGGCCTCGTTCGAGGACATCCGCGAGGAAGTCAAGAAGTCTGCCGACAACATGTACCTGACGATCAAGGAGGAGATCGACACCATGGCCGCAAACTTCCGCAAGTCTCTYGCTGAGATGGGCGACACGCTCAACAACGTCGAGACGAACCTCCAGAACCAGATCGCCATCCACAACGACGCCATCGCAGCCCTCAGGAAGGAGGCCCTCAAGAGCCTGAACGACCTCGAGACAGGCATCGCCACGGAGAACGCCGAGAGGAAGAAGATGTATGACCAGCTCAACGAGAAAGTCGCAGAGGGCTTCGCCCGCATCTCCGCTGCCATCGAGAAGGAGACGATCGCCCGCGAGAGGGCCGTCAGCGCCGCCACGACAGAGGCCCTCACAAACACGAA

>Sweh136

-GGTCCGCCGCGTCGACGACGACACGCGTGTGAAGATGATCAAGGACGCCATCGCGCACCTCGACAGACTCATCCAGACGGAGTCGAGGAAGCGCCAGGCCTCGTTCGAGGACATCCGCGAGGAAGTCAAGAAGTCTGCCGACAACATGTACCTGACGATCAAGGAGGAGATCGACACCATGGCCGCAAACTTCCGCAAGTCTCTTGCTGAGATGGGCGACACGCTCAACAACGTCGAGACGAACCTCCAGAACCAGATCGCCATCCACAACGACGCCATCGCAGCCCTCAGGAAGGAGGCCCTCAAGAGCCTGAACGACCTCGAGACAGGCATCGCCACGGAGAACGCCGAGAGGAAGAAGATGTATGACCAGCTCAACGAGAAAGTCGCAGAGGGCTTCGCCCGCATCTCCGCCGCCATCGAGAAGGAGACGATCGCCCGCGAGAGGGCCGTCAGCGCTGCCACGACAGAGGCCCTCACAAACACGAA

>Sweh137

-GGTCCGCCGCGTCGACGACGACACGCGTGTGAAGATGATCAAGGACGCCATCGCGCACCTCGACAGACTCATCCAGACRGAGTCGAGGAAGCGCCAGGCCTCGTTCGAGGACATCCGCGAGGAAGTCAAGAAGTCTGCCGACAACATGTACCTGACGATCAAGGAGGAGATCGACACCATGGCCGCAAACTTCCGCAAGTCTCTTGCTGAGATGGGCGACACGCTCAACAACGTCGAGACGAAYCTCCAGAACCAGATCGCCATCCACAACGACGCCATCGCAGCCCTCAGGAAGGAGGCCCTCAAGAGCCTGAACGACCTCGAGACAGGCATCGCCACGGAGAACGCCGAGAGGAAGAAGATGTATGACCAGCTCAACGAGAAAGTCGCAGAGGGCTTCGCCCGCATCTCCGCTGCCATCGAGAAGGAGACGATCGCCCGCGAGAGGGCCGTCAGCGCCGCCACGACAGAGGCCCTCACAAACACGAA

>Sweh141

-GGTCCGCCGCGTCGACGACGACACGCGTGTGAAGATGATCAAGGACGCCATCGCGCACCTYGACAGACTCATCCAGACRGAGTCGAGGAAGCGCCAGGCCTCGTTCGAGGACATCCGCGAGGAAGTCAAGAAGTCYGCCGACAACATGTACCTGACGATCAAGGAGGAGATCGACACCATGGCCGCAAACTTCCGCAAGTCTCTYGCTGAGATGGGCGACACGCTCAACAACGTCGAGACGAACCTCCAGAACCAGATCGCCATCCACAACGACGCCATCGCAGCCCTCAGGAAGGAGGCCCTCAAGAGCCTGAACGACCTCGARACRGGCATCGCCACGGAGAACGCCGAGAGGAAGAAGATGTATGACCAGCTCAACGAGAAAGTCGCAGAGGGCTTCGCCCGCATCTCCGCTGCCATCGAGAAGGAGACGATCGCCCGCGAGAGGGCCGTCAGCGCCGCCACGACAGAGGCCCTCACAAACACGAA

>Sweh142

--GTCCGCCGCGTCGACGACGACACGCGTGTGAAGATGATCAAGGACGCCATCGCGCACCTCGACAGACTCATCCAGACAGAGTCGAGGAAGCGCCAGGCCTCGTTCGAGGACATCCGCGAGGARGTCAAGAAGTCTGCCGACAACATGTACCTGACGATCAAGGAGGAGATCGACACCATGGCCGCAAACTTCCGCAAGTCTCTCGCTGAGATGGGCGACACGCTCAACAACGTCGAGACGAACCTCCAGAACCAGATCGCCATCCACAACGACGCCATCGCAGCCCTCAGGAAGGAGGCCCTCAAGAGCCTGAACGAYCTCGAGACAGGCATCGCCACGGAGAACGCCGAGAGGAAGAAGATGTATGACCAGCTCAACGAGAAAGTCGCAGAGGGCTTCGCCCGCATCTCCGCTGCCATCGAGAAGGAGACGATCGCCCGCGAGAGGGCCGTCAGCGCCGCCACGACAGAGGCCCTCACAAACACGAA

>Sweh143

--GTCCGCCGCGTCGACGACGACACGCGTGTGAAGATGATCAAGGACGCCATCGCGCACCTCGACAGACTCATCCAGACAGAGTCGAGGAAGCGCCAGGCCTCGTTCGAGGACATCCGCGAGGARGTCAAGAAGTCTGCCGACAACATGTACCTGACGATCAAGGAGGAGATCGACACCATGGCCGCAAACTTCCGCAAGTCTCTCGCTGAGATGGGCGACACGCTCAAYAACGTCGAGACGAACCTCCAGAACCAGATCGCCATCCACAACGACGCCATCGCAGCCCTCAGGAAGGAGGCCCTCAAGAGCCTGAACGACCTCGAGACAGGCATCGCCACGGAGAACGCCGAGAGGAAGAAGATGTATGACCAGCTCAACGAGAAAGTCGCAGAGGGCTTCGCCCGCATCTCCGCTGCCATCGAGAAGGAGACGATCGCCCGCGAGAGGGCCGTCAGCGCCGCCACGACAGAGGCCCTCACAAACACGAA

>Sweh144

AGGTCCGCCGCGTCGACGACGACACGCGTGTGAAGATGATCAAGGACGCCATCGCACACCTTGACAGACTCATCCAGACAGAGTCGAGGAAGCGCCAGGCCTCGTTCGAGGACATCCGCGAGGAAGTCAAGAAGTCTGCCGACAACATGTACCTGACGATCAAGGAGGAGATCGACACCATGGCCGCAAACTTCCGCAAGTCTCTTGCTGAGATGGGCGATACGCTCAACAACGTCGAGACGAACCTCCAGAACCAGATCGCCATCCACAACGACGCCATCGCAGCCCTTAGGAAGGAGGCCCTCAAGAGCCTGAACGACCTCGAGACAGGCATCGCCACGGAGAACGCCGAGAGGAAGAAGATGTACGACCAGCTCAACGAGAAAGTCGCAGAGGGCTTCGCCCGCATCTCCGCTGCCATCGAGAAGGAGACGATCGCCCGCGAGAGGGCCGTCAGCGCCGCCACGACAGAGGCCCTCACAAACACGAA

>Sweh148

AGGTCCGCCGCGTCGACGACGACACGCGTGTGAAGATGATCAAGGACGCCATCGCGCACCTyGACAGACTCATCCAGACAGAGTCGAGGAAGCGCCAGGCCTCGTTCGAGGACATCCGCGAGGAAGTCAAGAAGTCTGCCGACAACATGTACCTGACGATCAAGGAGGAGATCGACACCATGGCCGCAAACTTCCGCAAGTCTCTCGCTGAGATGGGCGACACGCTCAACAACGTCGAGACGAACCTCCAGAACCAGATCGCCATCCACAACGACGCCATCGCAGCCCTCAGGAAGGAGGCCCTCAAGAGCCTGAACGACCTCGAGACAGGCATCGCCACGGAGAACGCCGAGAGGAAGAAGATGTATGACCAGCTCAACGAGAAAGTCGCAGAGGGCTTCGCCCGCATCTCCGCTGCCATCGAGAAGGAGACGATCGCCCGCGAGAGGGCCGTCAGCGCCGCCACGACAGAGGCCCTCACAAACACGAA

>Sweh149

AGGTCCGCCGCGTCGACGACGACACGCGTGTGAAGATGATCAAGGACGCCATCGCRCACCTYGACAGACTCATCCAGACAGAGTCGAGGAAGCGCCAGGCCTCGTTCGAGGACATCCGCGAGGAAGTCAAGAAGTCTGCCGACAACATGTACCTGACGATCAAGGAGGAGATCGACACCATGGCCGCAAACTTCCGCAAGTCTCTYGCTGAGATGGGCGACACGCTCAACAACGTCGAGACGAACCTCCAGAACCAGATCGCCATCCACAACGACGCCATCGCAGCCCTCAGGAAGGAGGCCCTCAAGAGCCTGAACGACCTCGAGACAGGCATCGCCACGGAGAACGCCGAGAGGAAGAAGATGTATGACCAGCTCAACGAGAAAGTCGCAGAGGGCTTCGCCCGCATCTCCGCTGCCATCGAGAAGGAGACGATCGCCCGCGAGAGGGCCGTCAGCGCCGCCACGACAGAGGCCCTCACAAACACGAA

>Sweh151

~~~~~~GCCGCGTCGACGACGACACGCGTGTGAAGATGATCAAGGACGCCATCGCGCACCTYGACAGACTCATCCAGACAGAGTCGAGGAAGCGCCAGGCCTCGTTCGAGGACATCCGCGAGGAAGTCAAGAAGTCYGCCGACAACATGTACCTGACGATCAAGGAGGAGATCGACACCATGGCCGCAAACTTCCGCAAGTCTCTYGCTGAGATGGGCGACACGCTCAACAACGTCGAGACGAACCTCCAGAACCAGATCGCCATCCACAACGACGCCATCGCAGCCCTCAGGAAGGAGGCCCTCAAGAGCCTGAACGACCTCGAGACAGGCATCGCCACGGAGAACGCCGAGAGGAAGAAGATGTATGACCAGCTCAACGAGAAAGTCGCAGAGGGCTTCGCCCGCATCTCCGCTGCCATCGAGAAGGAGACGATCGCCCGCGAGAGGGCCGTCAGCGCCGCCACGACAGAGGCCCTCACAAACACGAA

>Sweh154

AGGTCCGCCGCGTCGACGACGACACGCGTGTGAAGATGATCAAGGACGCCATCGCGCACCTTGACAGACTCATCCAGACAGAGTCGAGGAAGCGCCAGGCCTCGTTCGAGGACATCCGCGAGGAAGTCAAGAAGTCTGCCGACAACATGTACCTGACGATCAAGGAGGAGATCGACACCATGGCCGCAAACTTCCGCAAGTCTCTTGCTGAGATGGGCGACACGCTCAACAACGTCGAGACGAACCTCCAGAACCAGATCGCCATCCACAACGACGCCATCGCAGCCCTTAGGAAGGAGGCCCTCAAGAGCCTGAACGACCTCGAGACAGGCATCGCCACGGAGAACGCCGAGAGGAAGAAGATGTATGACCAGCTCAACGAGAAAGTCGCAGAGGGCTTCGCCCGCATCTCCGCTGCCATCGAGAAGGAGACGATCGCCCGCGAGAGGGCCGTCAGCGCCGCCACGACAGAGGCCCTCACAAA

>Sweh156

-GGTCCGCCGCGTCGACGACGACACGCGTGTGAAGATGATCAAGGACGCCATCGCACACCTTGACAGACTCATCCAGACAGAGTCGAGGAAGCGCCAGGCCTCGTTCGAGGACATCCGCGAGGAAGTCAAGAAGTCTGCCGACAACATGTACCTGACGATCAAGGAGGAGATCGACACCATGGCCGCAAACTTCCGCAAGTCTCTTGCTGAGATGGGCGATACGCTCAACAACGTCGAGACGAACCTCCAGAACCAGATCGCCATCCACAACGACGCCATCGCAGCCCTTAGGAAGGAGGCCCTCAAGAGCCTGAACGACCTCGAGACAGGCATCGCCACGGAGAACGCCGAGAGGAAGAAGATGTACGACCAGCTCAACGAGAAAGTCGCAGAGGGCTTCGCCCGCATCTCCGCTGCCATCGAGAAGGAGACGATCGCCCGCGAGAGGGCCGTCAGCGCCGCCACGACAGAGGCCCTCACAAACACGAA

>Sweh156

~GGTCCGCCGCGTCGACGACGACACGCGTGTGAAGATGATCAAGGACGCCATCGCACACCTTGACAGACTCATCCAGACAGAGTCGAGGAAGCGCCAGGCCTCGTTCGAGGACATCCGCGAGGAAGTCAAGAAGTCTGCCGACAACATGTACCTGACGATCAAGGAGGAGATCGACACCATGGCCGCAAACTTCCGCAAGTCTCTTGCTGAGATGGGCGATACGCTCAACAACGTCGAGACGAACCTCCAGAACCAGATCGCCATCCACAACGACGCCATCGCAGCCCTTAGGAAGGAGGCCCTCAAGAGCCTGAACGACCTCGAGACAGGCATCGCCACGGAGAACGCCGAGAGGAAGAAGATGTACGACCAGCTCAACGAGAAAGTCGCAGAGGGCTTCGCCCGCATCTCCGCTGCCATCGAGAAGGAGACGATCGCCCGCGAGAGGGCCGTCAGCGCCGCCACGACAGAGGCCCTCACAAACACGAA

>Sweh158

AGGTCCGCCGCGTCGACGACGACACGCGTGTGAAGATGATCAAGGACGCCATCGCGCACCTTGACAGACTCATCCAGACAGAGTCGAGGAAGCGCCAGGCCTCGTTCGAGGACATCCGCGAGGAAGTCAAGAAGTCTGCCGACAACATGTACCTGACGATCAAGGAGGAGATCGACACCATGGCCGCAAACTTCCGCAAGTCTCTTGCTGAGATGGGCGACACGCTCAACAACGTCGAGACGAACCTCCAGAACCAGATCGCCATCCACAACGACGCCATCGCAGCCCTTAGGAAGGAGGCCCTCAAGAGCCTGAACGACCTCGAGACAGGCATCGCCACGGAGAACGCCGAGAGGAAGAAGATGTATGACCAGCTCAACGAGAAAGTCGCAGAGGGCTTCGCCCGCATCTCCGCTGCCATCGAGAAGGAGACGATCGCCCGCGAGAGGGCCGTCAGCGCCGCCACGACAGAGGCCCTCACAAAC-----

>Sweh159

AGGTCCGCCGCGTCGACGACGACACGCGTGTGAAGATGATCAAGGACGCCATCGCACACCTTGACAGACTCATCCAGACAGAGTCGAGGAAGCGCCAGGCCTCGTTCGAGGACATCCGCGAGGAAGTCAAGAAGTCTGCCGACAACATGTACCTGACGATCAAGGAGGAGATCGACACCATGGCCGCAAACTTCCGCAAGTCTCTTGCTGAGATGGGCGATACGCTCAACAACGTCGAGACGAACCTCCAGAACCAGATCGCCATCCACAACGACGCCATCGCAGCCCTTAGGAAGGAGGCCCTCAAGAGCCTGAACGACCTCGAGACAGGCATCGCCACGGAGAACGCCGAGAGGAAGAAGATGTACGACCAGCTCAACGAGAAAGTCGCAGAGGGCTTCGCCCGCATCTCCGCTGCCATCGAGAAGGAGACGATCGCCCGCGAGAGGGCCGTCAGCGCCGCCACGACAGAGGCCCT------------

>Sweh159

AGGTCCGCCGCGTCGACGACGACACGCGTGTGAAGATGATCAAGGACGCCATCGCACACCTTGACAGACTCATCCAGACAGAGTCGAGGAAGCGCCAGGCCTCGTTCGAGGACATCCGCGAGGAAGTCAAGAAGTCTGCCGACAACATGTACCTGACGATCAAGGAGGAGATCGACACCATGGCCGCAAACTTCCGCAAGTCTCTTGCTGAGATGGGCGATACGCTCAACAACGTCGAGACGAACCTCCAGAACCAGATCGCCATCCACAACGACGCCATCGCAGCCCTTAGGAAGGAGGCCCTCAAGAGCCTGAACGACCTCGAGACAGGCATCGCCACGGAGAACGCCGAGAGGAAGAAGATGTACGACCAGCTCAACGAGAAAGTCGCAGAGGGCTTCGCCCGCATCTCCGCTGCCATCGAGAAGGAGACGATCGCCCGCGAGAGGGCCGTCAGCGCCGCCACGACAGAGGCCCT------------

>Sweh160

AGGTCCGCCGCGTCGACGACGACACGCGTGTGAAGATGATCAAGGACGCCATCGCACACCTTGACAGACTCATCCAGACAGAGTCGAGGAAGCGCCAGGCCTCGTTCGAGGACATCCGCGAGGAAGTCAAGAAGTCTGCCGACAACATGTACCTGACGATCAAGGAGGAGATCGACACCATGGCCGCAAACTTCCGCAAGTCTCTTGCTGAGATGGGCGATACGCTCAACAACGTCGAGACGAACCTCCAGAACCAGATCGCCATCCACAACGACGCCATCGCAGCCCTTAGGAAGGAGGCCCTCAAGAGCCTGAACGACCTCGAGACAGGCATCGCCACGGAGAACGCCGAGAGGAAGAAGATGTACGACCAGCTCAACGAGAAAGTCGCAGAGGGCTTCGCCCGCATCTCCGCTGCCATCGAGAAGGAGACGATCGCCCGCGAGAGGGCCGTCAGCGCCGCCACGACAGAGGCCCTCACAAACACGAA

>Sweh161

AGGTCCGCCGCGTCGACGACGACACGCGTGTGAAGATGATCAAGGACGCCATCGCGCACCTCGACAGACTCATCCAGACAGAGTCGAGGAAGCGCCAGGCCTCGTTCGAGGACATCCGCGAGGAAGTCAAGAAGTCTGCCGACAACATGTACCTGACGATCAAGGAGGAGATCGACACCATGGCCGCAAACTTCCGCAAGTCTCTYGCTGAGATGGGCGACACGCTCAACAACGTCGAGACGAAYCTCCAGAACCAGATCGCCATCCACAACGACGCCATCGCAGCCCTCAGGAAGGAGGCCCTCAAGAGCCTGAACGACCTCGAGACAGGCATCGCCACGGAGAACGCCGAGAGGAAGAAGATGTATGACCAGCTCAACGAGAAAGTCGCAGAGGGCTTCGCCCGCATCTCCGCTGCCATCGAGAAGGAGACGATCGCCCGCGAGAGGGCCGTCAGCGCCGCCACGACAGAGGCCCTCACAAACACGA-

>Sweh163

AGGTCCGCCGCGTCGACGACGACACGCGTGTGAAGATGATCAAGGACGCCATCGCGCACCTCGACAGACTCATCCAGACAGAGTCGAGGAAGCGCCAGGCCTCGTTCGAGGACATCCGCGAGGAAGTCAAGAAGTCYGCCGACAACATGTACCTGACGATCAAGGAGGAGATCGACACCATGGCCGCAAACTTCCGCAAGTCTCTYGCTGAGATGGGCGACACGCTCAACAACGTCGAGACGAACCTCCAGAACCAGATCGCCATCCACAACGACGCCATCGCAGCCCTCAGGAAGGAGGCCCTCAAGAGCCTGAACGACCTCGAGACAGGCATCGCCACGGAGAACGCCGAGAGGAAGAAGATGTATGACCAGCTCAACGAGAAAGTCGCAGAGGGCTTCGCCCGCATCTCCGCTGCCATCGAGAAGGAGACGATCGCCCGCGAGAGGGCCGTCAGCGCCGCCACGACAGAGGCCCTCACAAACACGA-

>Sweh167

AGGTCCGCCGCGTCGACGACGACACGCGTGTGAAGATGATCAAGGACGCCATCGCGCACCTTGACAGACTCATCCAGACAGAGTCGAGGAAGCGCCAGGCCTCGTTCGAGGACATCCGCGAGGAAGTCAAGAAGTCTGCCGACAACATGTACCTGACGATCAAGGAGGAGATCGACACCATGGCCGCAAACTTCCGCAAGTCTCTTGCTGAGATGGGCGACACGCTCARCAACGTCGAGACGAACCTCCAGAACCAGATCGCCATCCACAACGACGCCATCGCAGCCCTTAGGAAGGAGGCCCTCAAGAGCCTGAACGACCTCGAGACRGGCATCGCCACGGAGAACGCCGAGAGGAAGAAGATGTATGACCAGCTCAACGAGAAAGTCGCAGAGGGCTTCGCCCGCATCTCCGCTGCCATCGAGAAGGAGACGATCGCCCGCGAGAGGGCCGTCAGCGCCGCCACGACAGAGGCCCTCACAAACACGAA

>SWeh168

AGGTCCGCCGCGTCGACGACGACACGCGTGTGAAGATGATCAAGGACGCCATCGCGCACCTTGACAGACTCATCCAGACAGAGTCGAGGAAGCGCCAGGCCTCGTTCGAGGACATCCGCGAGGAAGTCAAGAAGTCTGCCGACAACATGTACCTGACGATCAAGGAGGAGATCGACACCATGGCCGCAAACTTCCGCAAGTCTCTTGCTGAGATGGGCGACACGCTCAACAACGTCGAGACGAACCTCCAGAACCAGATCGCCATCCACAACGACGCCATCGCAGCCCTTAGGAAGGAGGCCCTCAAGAGCCTGAACGACCTCGAGACAGGCATCGCCACGGAGAACGCCGAGAGGAAGAAGATGTATGACCAGCTCAACGAGAAAGTCGCAGAGGGCTTCGCCCGCATCTCCGCTGCCATCGAGAAGGAGACGATCGCCCGCGAGAGGGCCGTCAGCGCCGCCACGACAGAGGCCCTCACAAACACGAA

>Sweh169

AGGTCCGCCGCGTCGACGACGACACGCGTGTGAAGATGATCAAGGACGCCATCGCGCACCTCGACAGACTCATCCAGACAGAGTCGAGGAAGCGCCAGGCCTCGTTCGAGGACATCCGCGAGGAAGTCAAGAAGTCYGCCGACAACATGTACCTGACGATCAAGGAGGAGATCGACACCATGGCCGCAAACTTCCGCAAGTCTCTCGCTGAGATGGGCGACACGCTCAACAACGTCGAGACGAACCTCCAGAACCAGATCGCCATCCACAACGACGCCATCGCAGCCCTCAGGAAGGAGGCCCTCAAGAGCCTGAACGACCTCGAGACAGGCATCGCCACGGAGAACGCCGAGAGGAAGAAGATGTATGACCAGCTCAACGAGAAAGTCGCAGAGGGCTTCGCCCGCATCTCCGCTGCCATCGAGAAGGAGACGATCGCCCGCGAGAGGGCCGTCAGCGCCGCCACGACAGAGGCCCTCACAAACACGAA

>Sweh170

AGGTCCGCCGCGTCGACGACGACACGCGTGTGAAGATGATCAAGGAYGCCATCGCGCACCTYGACAGACTCATCCAGACRGAGTCGAGGAAGCGCCAGGCCTCGTTCGAGGACATCCGCGAGGAAGTCAAGAAGTCTGCCGACAACATGTACCTGACGATCAAGGAGGAGATCGACACCATGGCCGCAAACTTCCGCAAGTCTCTYGCTGAGATGGGCGACACGCTCAACAACGTCGAGACGAACCTCCAGAACCAGATCGCCATCCACAACGACGCCATCGCRGCCCTCAGGAAGGAGGCCCTCAAGAGCCTGAACGACCTCGAGACAGGCATCGCCACGGAGAACGCCGAGAGGAAGAAGATGTATGACCAGCTCAACGAGAAAGTCGCAGAGGGCTTCGCCCGCATCTCCGCYGCCATCGAGAAGGAGACGATCGCCCGCGAGAGGGCCGTCAGCGCYGCCACGACAGAGGCCCTCACAAACACGAA

>Sweh179

AGGTCCGCCGCGTCGACGACGACACGCGTGTGAAGATGATCAAGGACGCCATCGCGCACCTTGACAGACTCATCCAGACAGAGTCGAGGAAGCGCCAGGCCTCGTTCGAGGACATCCGCGAGGAAGTCAAGAAGTCTGCCGACAACATGTACCTGACGATCAAGGAGGAGATCGACACCATGGCCGCAAACTTCCGCAAGTCTCTTGCTGAGATGGGCGACACGCTCAACAACGTCGAGACGAACCTCCAGAACCAGATCGCCATCCACAACGACGCCATCGCAGCCCTTAGGAAGGAGGCCCTCAAGAGCCTGAACGACCTCGAGACAGGCATCGCCACGGAGAACGCCGAGAGGAAGAAGATGTATGACCAGCTCAACGAGAAAGTCGCAGAGGGCTTCGCCCGCATCTCCGCTGCCATCGAGAAGGAGACGATCGCCCGCGAGAGGGCCGTCAGCGCCGCCACGACAGAGGCCCTCACAAACACGAA

>Sweh184

AGGTCCGCCGCGTCGACGACGACACGCGTGTGAAGATGATCAAGGACGCCATCGCGCACCTCGACAGACTCATCCAGACRGAGTCGAGGAAGCGCCAGGCCTCGTTCGAGGACATCCGCGAGGAAGTCAAGAAGTCTGCCGACAACATGTACCTGACGATCAAGGAGGAGATCGACACCATGGCCGCAAACTTCCGCAAGTCTCTYGCTGAGATGGGCGACACGCTCAACAACGTCGAGACGAACCTCCAGAACCAGATCGCCATCCACAACGACGCCATCGCAGCCCTCAGGAAGGAGGCCCTCAAGAGCCTGAACGACCTCGAGACAGGYATCGCCACGGAGAACGCCGAGAGGAAGAAGATGTATGACCAGCTCAACGAGAAAGTCGCAGAGGGCTTCGCCCGCATCTCCGCTGCCATCGAGAAGGAGACGATCGCCCGCGAGAGGGCCGTCAGCGCCGCCACGACAGAGGCCCTCACAAACACGAA

>Sweh186

AGGTCCGCCGCGTCGACGACGACACGCGTGTGAAGATGATCAAGGACGCCATCGCGCACCTTGACAGACTCATCCAGACAGAGTCGAGGAAGCGCCAGGCCTCGTTCGAGGACATCCGCGAGGAAGTCAAGAAGTCTGCCGACAACATGTACCTGACGATCAAGGAGGAGATCGAYACCATGGCCGCAAACTTCCGCAAGTCTCTCGCTGAGATGGGCGACACGCTCAACAACGTCGAGACGAACCTCCAGAACCAGATCGCCATCCACAACGACGCCATCGCAGCCCTCAGGAAGGAGGCCCTCAAGAGCCTGAACGACCTCGAGACAGGCATCGCCACGGAGAACGCCGAGAGGAAGAAGATGTATGACCAGCTCAACGAGAAAGTCGCAGAGGGCTTCGCCCGCATCTCCGCTGCCATCGAGAAGGAGACGATCGCCCGCGAGAGGGCCGTCAGCGCCGCCACGACAGAGGCCCTCACAAACACGAA

>Sweh188

AGGTCCGCCGCGTCGACGACGACACGCGTGTGAAGATGATCAAGGACGCCATCGCGCACCTTGACAGACTCATCCAGACAGAGTCGAGGAAGCGCCAGGCCTCGTTCGAGGACATCCGCGAGGAAGTCAAGAAGTCTGCCGACAACATGTACCTGACGATCAAGGAGGAGATCGATACCATGGCCGCAAACTTCCGCAAGTCTCTCGCTGAGATGGGCGACACGCTCAACAACGTCGAGACGAACCTCCAGAACCAGATCGCCATCCACAACGACGCCATCGCAGCCCTCAGGAAGGAGGCCCTCAAGAGCCTGAACGACCTCGAGACAGGCATCGCCACGGAGAACGCCGAGAGGAAGAAGATGTATGACCAGCTCAACGAGAAAGTCGCAGAGGGCTTCGCCCGCATCTCCGCTGCCATCGAGAAGGAGACGATCGCCCGCGAGAGGGCCGTCAGCGCCGCCACGACAGAGGCCCTCACAAACACGA-

>Sweh189

AGGTCCGCCGCGTCGACGACGACACGCGTGTGAAGATGATCAAGGACGCCATCGCGCACCTCGACAGACTCATCCAGACAGAGTCGAGGAAGCGCCAGGCCTCGTTCGAGGACATCCGCGAGGAAGTCAAGAAGTCTGCCGACAACATGTACCTGACGATCAAGGAGGAGATCGACACCATGGCCGCAAACTTCCGCAAGTCTCTYGCTGAGATGGGCGACACGCTCAACAACGTCGAGACGAACCTCCAGAACCAGATCGCYATCCACAACGACGCCATCGCAGCCCTCAGGAAGGAGGCCCTCAAGAGCCTGAACGACCTCGAGACAGGCATCGCCACGGAGAACGCCGAGAGGAAGAAGATGTATGACCAGCTCAACGAGAAAGTCGCAGAGGGCTTCGCCCGCATCTCCGCYGCCATCGAGAAGGAGACGATCGCCCGCGAGAGGGCCGTCAGCGCYGCCACGACAGAGGCCCTCACAAACACGAA

>Sweh191

AGGTCCGCCGCGTCGACGACGACACGCGTGTGAAGATGATCAAGGACGCCATCGCACACCTTGACAGACTCATCCAGACAGAGTCGAGGAAGCGCCAGGCCTCGTTCGAGGACATCCGCGAGGAAGTCAAGAAGTCTGCCGACAACATGTACCTGACGATCAAGGAGGAGATCGACACCATGGCCGCAAACTTCCGCAAGTCTCTTGCTGAGATGGGCGATACGCTCAACAACGTCGAGACGAACCTCCAGAACCAGATCGCCATCCACAACGACGCCATCGCAGCCCTTAGGAAGGAGGCCCTCAAGAGCCTGAACGACCTCGAGACAGGCATCGCCACGGAGAACGCCGAGAGGAAGAAGATGTACGACCAGCTCAACGAGAAAGTCGCAGAGGGCTTCGCCCGCATCTCCGCTGCCATCGAGAAGGAGACGATCGCCCGCGAGAGGGCCGTCAGCGCCGCCACGACAGAGGCCCTCACAAACACGAA

>Sweh192

AGGTCCGCCGCGTCGACGACGACACGCGTGTGAAGATGATCAAGGACGCCATCGCGCACCTTGACAGACTCATCCAGACAGAGTCGAGGAAGCGCCAGGCCTCGTTCGAGGACATCCGCGAGGAAGTCAAGAAGTCTGCCGACAACATGTACCTGACGATCAAGGAGGAGATCGACACCATGGCCGCAAACTTCCGCAAGTCTCTTGCTGAGATGGGCGATACGCTCAACAACGTCGAGACGAACCTCCAGAACCAGATCGCCATCCACAACGACGCCATCGCAGCCCTTAGGAAGGAGGCCCTCAAGAGCCTGAACGACCTCGAGACAGGCATCGCCACGGAGAACGCCGAGAGGAAGAAGATGTACGACCAGCTCAACGAGAAAGTCGCAGAGGGCTTCGCCCGCATCTCCGCTGCCATCGAGAAGGAGACGATCGCCCGCGAGAGGGCCGTCAGCGCCGCCACGACAGAGGCCCTCACAAACACGA-

>Sweh193

AGGTCCGCCGCGTCGACGACGACACGCGTGTGAAGATGATCAAGGACGCCATCGCGCACCTCGACAGACTCATCCAGACRGAGTCGAGGAAGCGCCAGGCCTCGTTCGAGGACATCCGCGAGGARGTCAAGAAGTCTGCCGACAACATGTACCTGACGATCAAGGAGGAGATCGACACCATGGCCGCAAACTTCCGCAAGTCTCTYGCTGAGATGGGCGACACGCTCAACAACGTCGAGACGAACCTCCAGAACCAGATCGCCATCCACAACGACGCCATCGCAGCCCTCAGGAAGGAGGCCCTCAAGAGCCTGAACGACCTCGAGACAGGCATCGCCACGGAGAACGCCGAGAGGAAGAAGATGTATGACCAGCTCAACGAGAAAGTCGCAGAGGGCTTCGCCCGCATCTCCGCTGCCATCGAGAAGGAGACGATCGCCCGCGAGAGGGCCGTCAGCGCCGCCACGACAGAGGCCCTCACAAACACGAA

>Sweh195

-GGTCCGCCGCGTCGACGACGACACGCGTGTGAAGATGATCAAGGACGCCATCGCGCACCTYGACAGACTCATCCAGACAGAGTCGAGGAAGCGCCAGGCCTCGTTCGAGGACATCCGCGAGGAAGTCAAGAAGTCTGCCGACAACATGTACCTGACGATCAAGGAGGAGATCGACACCATGGCCGCAAACTTCCGCAAGTCTCTYGCTGAGATGGGCGACACGCTCAACAACGTCGAGACGAACCTCCAGAACCAGATCGCCATCCACAACGACGCCATCGCAGCCCTCAGGAAGGAGGCCCTCAAGAGCCTGAACGACCTCGAGACAGGCATCGCCACGGAGAACGCCGAGAGGAAGAAGATGTATGACCAGCTCAACGAGAAAGTCGCAGAGGGCTTCGCCCGCATCTCCGCTGCCATCGAGAAGGAGACGATCGCCCGCGAGAGGGCCGTCAGCGCCGCCACGACAGAGGCCCTCACAAACACGAA

>Sweh196

--------------GACGACGACACGCGTGTGAAGATGATCAAGGACGCCATCGCGCACCTYGACAGACTCATCCAGACAGAGTCGAGGAAGCGCCAGGCCTCGTTCGAGGACATCCGCGAGGAAGTCAAGAAGTCTGCCGACAACATGTACCTGACGATCAAGGAGGAGATCGACACCATGGCCGCAAACTTCCGCAAGTCTCTCGCTGAGATGGGCGACACGCTCAACAACGTCGAGACGAACCTCCAGAACCAGATCGCCATCCACAACGACGCCATCGCAGCCCTCAGGAAGGAGGCCCTCAAGAGCCTGAACGACCTCGAGACAGGCATCGCCACGGAGAACGCCGAGAGGAAGAAGATGTATGACCAGCTCAACGAGAAAGTCGCAGAGGGCTTCGCCCGCATCTCCGCTGCCATCGAGAAGGAGACGATCGCCCGCGAGAGGGCCGTCAGCGCCGCCACGACAGAGGCCCTCACAAACACGAA

>Sweh197

AGGTCCGCCGCGTCGACGACGACACGCGTGTGAAGATGATCAAGGACGCCATCGCGCACCTCGACAGACTCATCCAGACAGAGTCGAGGAAGCGCCAGGCCTCRTTCGAGGACATCCGCGAGGAAGTCAAGAAGTCTGCCGACAACATGTACCTGACGATCAAGGAGGAGATCGACACCATGGCCGCAAACTTCCGCAAGTCTCTYGCTGAGATGGGCGACACGCTCAACAACGTCGAGACGAACCTCCAGAACCAGATCGCCATCCACAACGACGCCATCGCAGCCCTCAGGAAGGAGGCCCTCAAGAGCCTGAACGACCTCGAGACAGGCATCGCCACGGAGAACGCCGAGAGGAAGAAGATGTATGACCAGCTCAACGAGAAAGTCGCAGAGGGCTTCGCCCGCATCTCCGCTGCCATCGAGAAGGAGACGATCGCCCGCGAGAGGGCCGTCAGCGCCGCCACGACAGAGGCCCTCACAAACAC---

>Sweh198

AGGTCCGCCGCGTCGACGACGACACGCGTGTGAAGATGATCAAGGACGCCATCGCGCACCTCGACAGACTCATCCAGACGGAGTCGAGGAAGCGCCAGGCCTCGTTCGAGGACATTCGCGAGGAAGTCAAGAAGTCTGCCGACAACATGTACCTGACGATCAAGGAGGAGATCGACACCATGGCCGCAAACTTCCGCAAGTCTCTTGCTGAGATGGGCGACACGCTCAACAACGTCGAGACGAACCTCCAGAACCAGATCGCCATCCACAACGACGCCATCGCAGCCCTCAGGAAGGAGGCCCTCAAGAGCCTGAACGACCTCGAGACAGGCATCGCCACGGAGAACGCCGAGAGGAAGAAGATGTATGACCAGCTCAACGAGAAAGTCGCAGAGGGCTTCGCCCGCATCTCCGCCGCCATCGAGAAGGAGACGATCGCCCGCGAGAGGGCCGTCAGCGCTGCCACGACAGAGGCTCTCACAAACACGAA

>Sweh199

-GGTCCGCCGCGTCGACGACGACACGCGTGTGAAGATGATCAAGGACGCCATCGCGCACCTTGACAGACTCATCCAGACAGAGTCGAGGAAGCGCCAGGCCTCGTTCGAGGACATCCGCGAGGAAGTCAAGAAGTCTGCCGACAACATGTACCTGACGATCAAGGAGGAGATCGACACCATGGCCGCAAACTTCCGCAAGTCTCTTGCTGAGATGGGCGACACGCTCAACAACGTCGAGACGAACCTCCAGAACCAGATCGCCATCCACAACGACGCCATCGCAGCCCTTAGGAAGGAGGCCCTCAAGAGCCTGAACGACCTCGAGACAGGCATCGCCACGGAGAACGCCGAGAGGAAGAAGATGTATGACCAGCTCAACGAGAAAGTCGCAGAGGGCTTCGCCCGCATCTCCGCTGCCATCGAGAAGGAGACGATCGCCCGCGAGAGGGCCGTCAGCGCCGCCACGACAGAGGCCCTCACAAACACGAA

>Sweh200

AGGTCCGCCGCGTCGACGACGACACGCGTGTGAAGATGATCAAGGACGCCATCGCGCACCTTGACAGACTCATCCAGACAGAGTCGAGGAAGCGCCAGGCCTCGTTCGAGGACATCCGCGAGGAAGTCAAGAAGTCTGCCGACAACATGTACCTGACGATCAAGGAGGAGATCGACACCATGGCCGCAAACTTCCGCAAGTCTCTTGCTGAGATGGGCGACACGCTCAACAACGTCGAGACGAACCTCCAGAACCAGATCGCCATCCACAACGACGCCATCGCAGCCCTTAGGAAGGAGGCCCTCAAGAGCCTGAACGACCTCGAGACAGGCATCGCCACGGAGAACGCCGAGAGGAAGAAGATGTATGACCAGCTCAACGAGAAAGTCGCAGAGGGCTTCGCCCGCATCTCCGCTGCCATCGAGAAGGAGACGATCGCCCGCGAGAGGGCCGTCAGCGCCGCCACGACAGAGGCCCTCACAAACAC---

>Sweh202

-GGTCCGCCGCGTCGACGACGACACGCGTGTGAAGATGATCAAGGACGCCATCGCGCACCTTGACAGACTCATCCAGACAGAGTCGAGGAAGCGCCAGGCCTCGTTCGAGGACATCCGCGAGGAAGTCAAGAAGTCTGCCGACAACATGTACCTGACGATCAAGGAGGAGATCGACACCATGGCCGCAAACTTCCGCAAGTCTCTTGCTGAGATGGGCGACACGCTCAACAACGTCGAGACGAACCTCCAGAACCAGATCGCCATCCACAACGACGCCATCGCAGCCCTTAGGAAGGAGGCCCTCAAGAGCCTGAACGACCTCGAGACAGGCATCGCCACGGAGAACGCCGAGAGGAAGAAGATGTATGACCAGCTCAACGAGAAAGTCGCAGAGGGCTTCGCCCGCATCTCCGCTGCCATCGAGAAGGAGACGATCGCCCGCGAGAG------------------------------------------

>Sweh203

AGGTCCGCCGCGTCGACGACGACACGCGTGTGAAGATGATCAAGGACGCCATCGCGCACCTCGACAGACTCATCCAGACRGAGTCGAGGAAGCGCCAGGCCTCGTTCGAGGACATCCGCGAGGAAGTCAAGAAGTCTGCCGACAACATGTACCTGACGATCAAGGAGGAGATCGACACCATGGCCGCAAACTTCCGCAAGTCTCTTGCTGAGATGGGCGACACGCTCAACAACGTCGAGACGAACCTCCAGAACCAGATCGCCATCCACAACGACGCCATCGCAGCCCTCAGGAAGGAGGCCCTCAAGAGCCTGAACGACCTCGAGACAGGCATCGCCACGGAGAACGCCGAGAGGAAGAAGATGTATGACCAGCTCAACGAGAAAGTCGCAGAGGGCTTCGCCCGCATCTCCGCYGCCATCGAGAAGGAGACGATCGCCCGCGAGAGGGCCGTCAGCGCYGCCACGACAGAGGCCCTCACAAACACGAA

>Sweh206

AGGTCCGCCGCGTCGACGACGACACGCGTGTGAAGATGATCAAGGACGCCATCGCGCACCTCGACAGACTCATCCAGACAGAGTCGAGGAAGCGCCAGGCCTCGTTCGAGGACATCCGCGAGGAAGTCAAGAAGTCTGCCGACAACATGTACCTGACGATCAAGGAGGAGATCGACACCATGGCCGCAAACTTCCGCAAGTCTCTYGCTGAGATGGGCGACACGCTCAACAACGTCGAGACGAAYCTCCAGAACCAGATCGCCATCCACAACGACGCCATCGCAGCCCTCAGGAAGGAGGCCCTCAAGAGCCTGAACGACCTCGAGACAGGCATCGCCACGGAGAACGCCGAGAGGAAGAAGATGTATGACCAGCTCAACGAGAAAGTCGCAGAGGGCTTCGCCCGCATCTCCGCTGCCATCGAGAAGGAGACGATCGCCCGCGAGAGGGCCGTCAGCGCCGCCACGACAGAGGCCCTCACAAACACGAA

>Sweh208

AGGTCCGCCGCGTCGACGACGACACGCGTGTGAAGATGATCAAGGACGCCATCGCGCACCTCGACAGACTCATCCAGACGGAGTCGAGGAAGCGCCAGGCCTCGTTCGAGGACATCCGCGAGGAAGTCAAGAAGTCTGCCGACAACATGTACCTGACGATCAAGGAGGAGATCGACACCATGGCCGCAAACTTCCGCAAGTCTCTTGCTGAGATGGGCGACACGCTCAACAACGTCGAGACGAACCTCCAGAACCAGATCGCCATCCACAACGACGCCATCGCAGCCCTCAGGAAGGAGGCCCTCAAGAGCCTGAACGACCTCGAGACAGGCATCGCCACGGAGAACGCCGAGAGGAAGAAGATGTATGACCAGCTCAACGAGAAAGTCGCAGAGGGCTTCGCCCGCATCTCCGCCGCCATCGAGAAGGAGACGATCGCCCGCGAGAGGGCCGTCAGCGCTGCCACGACAGAGGCCCTCACAAACACGAA

>Sweh212

AGGTCCGCCGCGTCGACGACGACACGCGTGTGAAGATGATCAAGGACGCCATCGCGCACCTTGACAGACTCATCCAGACAGAGTCGAGGAAGCGCCAGGCCTCGTTCGAGGACATCCGCGAGGAAGTCAAGAAGTCTGCCGACAACATGTACCTGACGATCAAGGAGGAGATCGACACCATGGCCGCAAACTTCCGCAAGTCTCTTGCTGAGATGGGCGAYACGCTCAACAACGTCGAGACGAACCTCCAGAACCAGATCGCCATCCACAACGACGCCATCGCAGCCCTTAGGAAGGAGGCCCTCAAGAGCCTGAACGACCTCGAGACAGGCATCGCCACGGAGAACGCCGAGAGGAAGAAGATGTAYGACCAGCTCAACGAGAAAGTCGCAGAGGGCTTCGCCCGCATCTCCGCTGCCATCGAGAAGGAGACGATCGCCCGCGAGAGGGCCGTCAGCGCCGCCACGACAGAGGCCCTCACAAACACGAA

>Sweh213

AGGTCCGCCGCGTCGACGACGACACGCGTGTGAAGATGATCAAGGACGCCATCGCACACCTTGACAGACTCATCCAGACAGAGTCGAGGAAGCGCCAGGCCTCGTTCGAGGACATCCGCGAGGAAGTCAAGAAGTCTGCCGACAACATGTACCTGACGATCAAGGAGGAGATCGACACCATGGCCGCAAACTTCCGCAAGTCTCTTGCTGAGATGGGCGATACGCTCAACAACGTCGAGACGAACCTCCAGAACCAGATCGCCATCCACAACGACGCCATCGCAGCCCTTAGGAAGGAGGCCCTCAAGAGCCTGAACGACCTCGAGACAGGCATCGCCACGGAGAACGCCGAGAGGAAGAAGATGTACGACCAGCTCAACGAGAAAGTCGCAGAGGGCTTCGCCCGCATCTCCGCTGCCATCGAGAAGGAGACGATCGCCCGCGAGAGGGCCGTCAGCGCCGCCACGACAGAGGCCCTCACAAACACGAA

>Sweh217

AGGTCCGCCGCGTCGACGACGACACGCGTGTGAAGATGATCAAGGACGCCATCGCACACCTTGACAGACTCATCCAGACAGAGTCGAGGAAGCGCCAGGCCTCGTTCGAGGACATCCGCGAGGAAGTCAAGAAGTCTGCCGACAACATGTACCTGACGATCAAGGAGGAGATCGACACCATGGCCGCAAACTTCCGCAAGTCTCTTGCTGAGATGGGCGATACGCTCAACAACGTCGAGACGAACCTCCAGAACCAGATCGCCATCCACAACGACGCCATCGCAGCCCTTAGGAAGGAGGCCCTCAAGAGCCTGAACGACCTCGAGACAGGCATCGCCACGGAGAACGCCGAGAGGAAGAAGATGTACGACCAGCTCAACGAGAAAGTCGCAGAGGGCTTCGCCCGCATCTCCGCTGCCATCGAGAAGGAGACGATCGCCCGCGAGAGGGCCGTCAGCGCCGCCACGACAGAGGCCCTCACAAACACGAA
