# Supplementary material for: Multilocus Genotyping of Human Giardia Isolates Suggests Limited Zoonotic Transmission and Association between Assemblage B and Flatulence in Children
Source: PLoS Negl Trop Dis. 2011 Aug 2;5(8):e1262. doi: 10.1371/journal.pntd.0001262 (PMC3149019; doi:10.1371/journal.pntd.0001262)
Supplement: File S2 — Gdh sequences from 120 isolates. (DOC) [file pntd.0001262.s003.doc]

Supplementary file S2 . Gdh sequences from 120 isolates

>Sweh001

GCTCTCGGGCCCTACAAGGGTGGTCTCCGCTTCCACCCCTCTGTCAACCTCTCGATCCTTAAGTTCCTCGGCTTTGAGCAGATCCTGAAGAACTCCCTTACCACGCTTCCGATGGGCGGTGGTAAGGGCGGCTCCGACTTCGATCCTAAGGGCAAGTCGGACAACGAGGTCATGCGCTTCTGCCAGTCCTTTATGACCGAGCTCCAGAGGCACGTCGGGGCTGACACCGACGTTCCTGCTGGCGATATTGGCGTCGGCGGTCGCGAGATCGGTTATCTGTTTGGACAGTATAAGCGCCTCAGGAACGAGTTTACGGGCGTCCTCACGGGCAAGAACATCAAGTGGGGCGGGTCTCTCATCAGACCAGAGGCCACAGGGTATGGAGCTGTCTACTTCCTGGAGGAGATGT

>Sweh005

GCTCTCGGGCCCTACAAGGGTGGTCTCCGCTTCCACCCCTCTGTCAACCTCTCGATCCTYAAGTTCCTCGGCTTTGAGCAGATCCTGAAGAACTCCCTTACCACGCTYCCGATGGGCGGTGGTAAGGGCGGCTCCGACTTCGATCCTAAGGGCAAGTCGGACAACGAGGTCATGCGCTTCTGCCAGTCCTTTATGACCGAGCTCCAGAGGCACGTCGGRGCTGACACCGACGTTCCTGCTGGCGATATTGGCGTCGGYGGTCGCGAGATYGGTTATCTGTTTGGACAGTAYAAGCGCCTCAGGAACGAGTTYACGGGCGTCCTCACGGGCAAGAACATCAAGTGGGGCGGGTCTCTYATCAGRCCAGAGGCCACAGGRTATGGAGCTGTCTACTTCCTGGAGGAGA---

>Sweh006

GCTCTCGGGCCCTACAAGGGTGGTCTCCGCTTCCACCCCTCTGTCAAYCTCTCGATCCTYAAGTTCCTCGGCTTTGAGCAGATCCTGAAGAACTCCCTTACCACGCTYCCRATGGGCGGTGGTAAGGGCGGCTCCGACTTCGATCCYAAGGGCAAGTCGGACAAYGAGGTCATGCGCTTYTGCCAGTCCTTTATGACYGAGCTCCAGAGGCACGTCGGGGCTGACACCGACGTTCCTGCTGGCGATATTGGCGTCGGCGGTCGCGAGATYGGTTATYTGTTTGGACAGTAYAAGCGYCTCAGGAACGAGTTCACGGGCGTCCTCACGGGCAAGAACATCAAGTGGGGCGGGTCTCTCATCAGRCCAGAGGCCACAGGGTATGGAGCTGTCTACTTCCTGGAGGAGATG-

>Sweh007

GCTCTCGGGCCCTACAAGGGTGGTCTCCGCTTCCACCCCTCTGTCAACCTCTCGATCCTTAAGTTCCTCGGCTTTGAGCAGATCCTGAAGAACTCCCTTACCACGCTCCCRATGGGCGGTGGTAAGGGCGGCTCCGACTTCGATCCTAAGGGCAAGTCGGACAACGAGGTCATGCGCTTTTGCCAGTCCTTTATGACTGAGCTCCAGAGGCACGTCGGGGCTGACACCGACGTTCCTGCTGGCGATATTGGCGTCGGCGGTCGCGAGATYGGTTATCTGTTTGGACAGTAYAAGCGYCTCAGGAACGAGTTCACGGGCGTCCTCACGGGCAAGAACATCAAGTGGGGCGGGTCTCTCATCAGGCCAGAGGCCACAGGGTATGGAGCTGTCTACTTCCTGGAGGAGATGT

>Sweh008

GCTCTCGGGCCCTACAAGGGTGGTCTCCGCTTCCACCCCTCTGTCAACCTCTCGATCCTYAAGTTCCTCGGCTTTGAGCAGATCCTGAAGAACTCCCTTACCACGCTCCCRATGGGCGGTGGTAAGGGCGGCTCCGACTTCGATCCTAAGGGCAAGTCGGACAACGAGGTCATGCGCTTYTGCCAGTCCTTTATGACYGAGCTCCAGAGGCACGTCGGGGCTGACACCGACGTTCCTGCTGGCGATATTGGCGTCGGCGGTCGCGAGATYGGTTATCTGTTTGGACAGTAYAAGCGYCTCAGGAACGAGTTCACGGGCGTYCTCACGGGCAAGAACATCAAGTGGGGCGGGTCTCTCATCAGRCCAGAGGCCACAGGGTATGGAGCTGTCTACTTCCTGGAGGAGATGT

>Sweh009

GCTCTCGGGCCCTACAAGGGTGGTCTCCGCTTCCACCCCTCTGTCAAYCTCTCGATCCTYAAGTTCCTCGGCTTTGAGCAGATCCTGAAGAACTCCCTTACCACGCTYCCRATGGGCGGTGGTAARGGCGGCTCCGACTTCGATCCTAAGGGCAARTCGGACAACGAGGTCATGCGCTTYTGYCAGTCCTTTATGACCGAGCTCCAGAGGCACGTCGGGGCTGACACCGACGTTCCTGCTGGCGATATTGGCGTCGGCGGTCGCGAGATCGGTTATCTGTTTGGACAGTAYAAGCGCCTCAGGAACGAGTTYACGGGCGTYCTCACGGGCAAGAACATCAAGTGGGGYGGGTCTCTCATCAGRCCAGAGGCCACAGGGTATGGAGCTGTCTACTTCCTGGAGGAGATGT

>Sweh010

GCTCTCGGGCCCTACAAGGGTGGTCTCCGCTTCCACCCCTCTGTCAACCTCTCGATCCTYAAGTTCCTCGGCTTTGAGCAGATCCTGAAGAACTCCCTTACCACGCTYCCGATGGGCGGTGGTAAGGGCGGCTCCGACTTCGATCCTAAGGGCAAGTCGGACAACGAGGTCATGCGCTTCTGCCAGTCCTTYATGACCGAGCTCCAGAGGCACGTCGGGGCTGACACCGACGTTCCTGCTGGCGATATTGGCGTCGGCGGTCGCGAGATYGGTTATCTGTTTGGACAGTAYAAGCGYCTCAGGAACGAGTTYACGGGCGTCCTCACGGGCAAGAAYATCAAGTGGGGYGGGTCTCTYATCAGRCCAGAGGCCACAGGGTATGGAGCTGTCTACTTCCTGGAGGAGAT--

>Sweh011

-CTCTCGGGCCCTACAAGGGTGGTCTCCGCTTCCACCCCTCTGTCAACCTCTCGATCCTYAAGTTCCTCGGCTTTGAGCAGATCCTGAAGAACTCCCTTACCACGCTYCCGATGGGCGGTGGTAAGGGCGGCTCCGACTTCGATCCTAAGGGCAAGTCGGACAACGAGGTCATGCGCTTCTGCCAGTCCTTYATGACCGAGCTCCAGAGGCACGTCGGGGCTGACACCGACGTTCCTGCTGGCGATATTGGCGTCGGCGGTCGCGAGATYGGTTATCTGTTTGGACAGTAYAAGCGYCTCAGGAACGAGTTYACGGGCGTCCTCACGGGCAAGAACATCAAGTGGGGYGGGTCTCTCATCAGRCCAGAGGCCACAGGGTATGGAGCTGTCTACTTCCTGGAGGAGA---

>Sweh013

---CTCGGGCCCTACAAGGGTGGTCTCCGCTTCCACCCCTCTGTCAACCTCTCGATCCTTAAGTTCCTCGGCTTTGAGCAGATCCTGAAGAACTCCCTTACCACGCTYCCGATGGGCGGTGGTAAGGGCGGCTCCGACTTCGATCCYAAGGGCAAGTCGGACAACGAGGTCATGCGCTTCTGCCAGTCCTTTATGACCGAGCTCCAGAGGCACGTCGGGGCTGACACCGACGTTCCTGCTGGCGATATTGGCGTCGGCGGTCGCGAGATYGGTTATCTGTTTGGACAGTATAAGCGCCTCAGGAACGAGTTCACGGGCGTCCTCACGGGCAAGAACATCAARTGGGGCGGGTCTCTCATCAGRCCAGAGGCCACAGGGTATGGAGCTGTCTACTTCCTGGAGGAGA---

>Sweh014

-~~CTCGGGCCCTACAAGGGTGGTCTCCGCTTCCACCCCTCTGTCAAYCTCTCGATCCTYAAGTTCCTCGGCTTTGAGCAGATCCTGAAGAACTCCCTTACCACGCTYCCGATGGGCGGTGGTAAGGGCGGCTCCGACTTCGATCCTAAGGGCAAGTCGGACAACGAGGTCATGCGCTTTTGCCAGTCCTTTATGACYGAGCTCCAGAGGCACGTCGGGGCTGACACCGACGTTCCTGCTGGCGATATTGGCGTCGGCGGTCGCGAGATYGGTTATCTGTTTGGACAGTAYAAGCGYCTCAGGAACGAGTTCACGGGCGTCCTCACGGGCAAGAACATCAAGTGGGGYGGGTCTCTYATCAGRCCAGAGGCCACAGGGTATGGAGCTGTCTACTTCCTGGAGGAGATGT

>Sweh015

~CTCTCGGGCCCTACAAGGGTGGTCTCCGCTTCCACCCCTCTGTCAAYCTCTCGATCCTYAAGTTCCTCGGCTTTGAGCAGATCCTGAAGAACTCCCTTACCACGCTYCCRATGGGCGGTGGTAAGGGCGGCTCCGACTTCGATCCTAAGGGCAAGTCGGACAACGAGGTCATGCGCTTTTGCCAGTCCTTTATGACYGAGCTCCAGAGGCACGTCGGGGCTGACACCGACGTTCCTGCTGGCGATATTGGCGTCGGCGGTCGCGAGATCGGTTATCTGTTTGGACAGTAYAAGCGYCTCAGGAACGAGTTYACGGGCGTCCTCACGGGCAAGAACATCAAGTGGGGCGGGTCTCTCATCAGRCCAGAGGCCACAGGGTATGGAGCTGTCTAYTTCCTGGAGGAGATGT

>Sweh019

GCTCTCGGGCCCTACAAGGGTGGTCTCCGCTTCCACCCCTCTGTCAACCTCTCGATCCTTAAGTTCCTCGGCTTTGAGCAGATCCTGAAGAACTCCCTTACCACGCTCCCGATGGGCGGTGGTAAGGGCGGCTCCGACTTCGATCCTAAGGGCAAGTCGGACAACGAGGTCATGCGCTTTTGCCAGTCCTTTATGACTGAGCTCCAGAGGCACGTCGGGGCTGACACCGACGTTCCTGCTGGCGATATTGGCGTCGGCGGTCGCGAGATTGGTTATCTGTTTGGACAGTACAAGCGCCTCAGGAACGAGTTCACGGGCGTCCTCACGGGCAAGAACATCAAGTGGGGCGGGTCTCTCATCAGGCCAGAGGCCACAGGGTATGGAGCTGTCTACTTCCTGGAGGAGATGT

>Sweh021

GCTCTCGGGCCCTACAAGGGTGGTCTCCGCTTCCACCCCTCTGTCAACCTCTCGATCCTTAAGTTCCTCGGCTTTGAGCAGATCCTGAAGAACTCCCTTACCACGCTTCCGATGGGCGGTGGTAAGGGCGGCTCCGACTTCGATCCTAAGGGCAAGTCGGACAACGAGGTCATGCGCTTCTGCCAGTCCTTTATGACCGAGCTCCAGAGGCACGTCGGGGCTGACACCGACGTTCCTGCTGGCGATATTGGCGTCGGCGGTCGCGAGATCGGTTATCTGTTTGGACAGTATAAGCGCCTCAGGAACGAGTTTACGGGCGTCCTCACGGGCAAGAACATCAAGTGGGGCGGGTCTCTCATCAGACCAGAGGCCACAGGGTATGGAGCTGTCTACTTCCTGGAGGAGATGT

>Sweh022

GCTCTCGGGCCCTACAAGGGTGGTCTCCGCTTCCACCCCTCTGTCAACCTCTCGATCCTTAAGTTCCTCGGCTTTGAGCAGATCCTGAAGAACTCCCTTACCACGCTTCCGATGGGCGGTGGTAAGGGCGGCTCCGACTTCGATCCTAAGGGCAAGTCGGACAACGAGGTCATGCGCTTCTGCCAGTCCTTTATGACCGAGCTCCAGAGGCACGTCGGGGCTGACACCGACGTTCCTGCTGGCGATATTGGCGTCGGCGGTCGCGAGATCGGTTATCTGTTTGGACAGTATAAGCGCCTCAGGAACGAGTTTACGGGCGTCCTCACGGGCAAGAACATCAAGTGGGGCGGGTCTCTCATCAGACCAGAGGCCACAGGGTATGGAGCTGTCTACTTCCTGGAGGAGATGT

>Sweh023

GCTCTCGGGCCCTACAAGGGTGGTCTCCGCTTCCACCCCTCTGTCAACCTCTCGATCCTTAAGTTCCTCGGCTTTGAGCAGATCCTGAAGAACTCCCTTACCACGCTYCCGATGGGCGGTGGTAAGGGCGGCTCCGACTTCGATCCTAAGGGCAAGTCGGACAACGAGGTCATGCGCTTCTGCCAGTCCTTTATGACCGAGCTCCAGAGGCACGTCGGGGCTGACACCGACGTTCCTGCTGGCGATATTGGCGTCGGCGGTCGCGAGATCGGTTATCTGTTTGGACAGTATAAGCGCCTCAGGAACGAGTTYACGGGCGTYCTCACGGGCAAGAACATCAAGTGGGGYGGGTCTCTCATCAGACCAGAGGCCACAGGGTATGGAGCTGTCTACTTCCTGGAGGAGATGT

>Sweh025

GCTCTCGGGCCCTACAAGGGTGGTCTCCGCTTCCACCCCTCTGTCAAYCTCTCGATCCTYAAGTTCCTCGGCTTTGAGCAGATCCTGAAGAACTCCCTTACCACGCTYCCGATGGGCGGTGGTAAGGGCGGCTCCGACTTYGATCCTAAGGGCAAGTCGGACAACGAGGTCATGCGCTTCTGCCAGTCCTTTATGACCGAGCTCCAGAGGCACGTCGGGGCTGACACCGACGTTCCTGCTGGCGATATTGGCGTCGGCGGTCGCGAGATCGGTTATCTGTTTGGACAGTATAAGCGCCTCAGGAACGAGTTCACGGGCGTCCTCACGGGCAAGAACATCAAGTGGGGYGGGTCTCTYATCAGACCAGAGGCCACAGGGTATGGAGCTGTCTACTTCCTGGAGGAGATGT

>Sweh027

GCTCTCGGGCCCTACAARGGTGGTCTCCGCTTCCACCCCTCTGTCAAYCTCTCGATCCTCAAGTTCCTCGGCTTTGAGCAGATCCTGAAGAACTCCCTTACCACGCTTCCGATGGGCGGTGGTAAGGGCGGCTCCGACTTCGATCCTAAGGGCAAGTCGGACAAYGAGGTCATGCGCTTTTGCCAGTCCTTTATGACTGAGCTCCAGAGGCACGTCGGGGCTGACACCGACGTTCCTGCTGGCGATATTGGCGTCGGCGGTCGCGAGATYGGTTATCTGTTTGGACAGTAYAAGCGYCTCAGGAACGAGTTYAYGGGCGTCCTCACGGGCAARAACATCAAGTGGGGCGGGTCTCTCATCAGRCCAGAGGCCACAGGGTATGGAGCTGTCTACTTCCTGGAGGAGATG-

>Sweh028

GCTCTCGGGCCCTACAAGGGTGGTCTCCGCTTCCACCCCTCTGTCAACCTCTCGATCCTTAAGTTCCTCGGCTTTGAGCAGATCCTGAAGAACTCCCTTACCACGCTYCCRATGGGCGGTGGTAAGGGCGGCTCCGACTTCGATCCTAAGGGCAAGTCGGACAACGAGGTCATGCGCTTTTGCCAGTCCTTTATGACYGAGCTCCAGAGGCACGTCGGGGCTGACACCGACGTTCCTGCTGGCGATATTGGCGTCGGCGGTCGCGAGATTGGTTATCTGTTTGGACAGTACAAGCGYCTCAGGAACGAGTTCACGGGYGTCCTCACGGGCAAGAACATCAAGTGGGGCGGGTCTCTCATCAGGCCAGAGGCCACAGGGTATGGAGCTGTCTACTTCCTGGAGGAGATG-

>Sweh033

GCTCTCGGGCCCTACAAGGGTGGTCTCCGCTTCCACCCCTCTGTCAACCTCTCGATCCTTAAGTTCCTCGGCTTTGAGCAGATCCTGAAGAACTCCCTTACCACGCTCCCGATGGGCGGTGGTAAGGGCGGCTCCGACTTCGATCCTAAGGGCAAGTCGGACAACGAGGTCATGCGCTTCTGCCAGTCCTTTATGACCGAGCTCCAGAGGCACGTCGGGGCTGACACCGACGTTCCTGCTGGCGATATTGGCGTCGGCGGTCGCGAGATCGGTTATCTGTTTGGACAGTATAAGCGCCTCAGGAACGAGTTTACGGGCGTCCTCACGGGCAAGAACATCAAGTGGGGCGGGTCTCTCATCAGACCAGAGGCCACAGGGTATGGAGCTGTCTACTTCCTGGAGGAGATGT

>Sweh034

GCTCTCGGGCCCTACAAGGGTGGTCTCCGCTTCCACCCCTCTGTCAACCTCTCGATCCTTAAGTTCCTCGGCTTTGAGCAGATCCTGAAGAACTCCCTTACCACGCTCCCGATGGGCGGTGGTAAGGGCGGCTCCGACTTCGATCCTAAGGGCAAGTCGGACAACGAGGTCATGCGCTTCTGCCAGTCCTTTATGACCGAGCTCCAGAGGCACGTCGGGGCTGACACCGACGTTCCTGCTGGCGATATTGGCGTCGGCGGTCGCGAGATCGGTTATCTGTTTGGACAGTATAAGCGCCTCAGGAACGAGTTTACGGGCGTCCTCACGGGCAAGAACATCAAGTGGGGCGGGTCTCTCATCAGACCAGAGGCCACAGGGTATGGAGCTGTCTACTTCCTGG---------

>Sweh035

GCTCTCGGGCCCTACAAGGGTGGTCTCCGCTTCCACCCCTCTGTCAACCTCTCGATCCTTAAGTTCCTCGGCTTTGAGCAGATCCTGAAGAACTCCCTTACCACGCTCCCGATGGGCGGTGGTAAGGGCGGCTCCGACTTCGATCCTAAGGGCAAGTCGGACAACGAGGTCATGCGCTTCTGCCAGTCCTTTATGACCGAGCTCCAGAGGCACGTCGGGGCTGACACCGACGTTCCTGCTGGCGATATTGGCGTCGGCGGTCGCGAGATCGGTTATCTGTTTGGACAGTATAAGCGCCTCAGGAACGAGTTCACGGGCGTCCTCACGGGCAAGAACATCAAGTGGGGCGGGTCTCTCATCAGACCAGAGGCCACAGGGTATGGAGCTGTCTACTTCCTGGAGGAGATGT

>Sweh039

GCTCTCGGGCCCTACAAGGGTGGTCTCCGCTTCCACCCCTCTGTCAACCTCTCGATCCTYAAGTTCCTCGGCTTTGAGCAGATCCTGAAGAACTCCCTTACCACGCTYCCRATGGGCGGTGGTAAGGGCGGCTCCGACTTCGATCCTAAGGGCAAGTCGGACAACGAGGTCATGCGCTTTTGCCAGTCCTTTATGACTGAGCTCCAGAGGCACGTCGGGGCTGACACCGACGTTCCCGCTGGCGATATTGGCGTCGGCGGTCGCGAGATTGGTTATCTGTTTGGACAGTACAAGCGTCTCAGGAACGAGTTCACGGGCGTCCTCACGGGCAAGAACATCAAGTGGGGCGGGTCTCTCATCAGGCCAGAGGCCACAGGGTATGGAGCTGTCTACTTCCTGGAGGAGATGT

>Sweh041

GCTCTCGGGCCCTACAAGGGTGGTCTCCGCTTCCACCCCTCTGTCAACCTCTCGATCCTTAAGTTCCTCGGCTTTGAGCAGATCCTGAAGAACTCCCTTACCACGCTTCCGATGGGCGGTGGTAAGGGCGGCTCCGACTTCGATCCTAAGGGCAAGTCGGACAACGAGGTCATGCGCTTCTGCCAGTCCTTTATGACCGAGCTCCAGAGGCACGTCGGGGCTGACACCGACGTTCCTGCTGGCGATATTGGCGTCGGCGGTCGCGAGATCGGTTATCTGTTTGGACAGTATAAGCGCCTCAGGAACGAGTTTACGGGCGTCCTCACGGGCAAGAACATCAAGTGGGGCGGGTCTCTCATCAGACCAGAGGCCACAGGGTATGGAGCTGTCTACTTCCTGGAGGAGATG-

>Sweh042

GCTCTCGGGCCCTACAAGGGTGGTCTCCGCTTCCACCCCTCTGTCAACCTCTCGATCCTTAAGTTCCTCGGCTTTGAGCAGATCCTGAAGAACTCCCTTACCACGCTCCCAATGGGCGGTGGTAAGGGCGGCTCCGACTTCGATCCTAAGGGCAAGTCGGACAACGAGGTCATGCGCTTTTGCCAGTCCTTTATGACTGAGCTCCAGAGGCACGTCGGGGCTGACACCGACGTTCCYGCTGGCGATATTGGCGTCGGCGGTCGCGAGATTGGTTATCTGTTTGGACAGTACAAGCGTCTCAGGAACGAGTTCACGGGCGTCCTCACGGGCAAGAACATCAAGTGGGGCGGGTCTCTCATCAGGCCAGAGGCCACAGGGTATGGAGCTGTCTACTTCCTGGAGGAGATGT

>Sweh043

GCTCTCGGGCCCTACAAGGGTGGTCTCCGCTTCCACCCCTCTGTCAACCTCTCGATCCTTAAGTTCCTCGGCTTTGAGCAGATCCTGAAGAACTCCCTTACCACGCTCCCGATGGGCGGTGGTAAGGGCGGCTCCGACTTCGATCCYAAGGGCAAGTCGGACAACGAGGTCATGCGCTTCTGYCAGTCCTTTATGACCGAGCTCCAGAGGCACGTCGGGGCTGACACCGACGTTCCTGCTGGCGATATTGGCGTCGGCGGTCGCGAGATCGGTTATCTGTTTGGACAGTATAAGCGCCTCAGGAACGAGTTCACGGGCGTYCTCACGGGCAAGAACATCAAGTGGGGCGGGTCTCTCATCAGACCAGAGGCCACAGGGTATGGAGCTGTCTACTTCCTGGAGGAGATGT

>Sweh044

GCTCTCGGGCCCTACAAGGGTGGTCTCCGCTTCCACCCCTCTGTCAAYCTCTCGATCCTYAAGTTCCTCGGCTTTGAGCAGATCCTGAAGAACTCCCTTACCACGCTYCCGATGGGCGGTGGTAAGGGCGGCTCCGACTTCGATCCTAAGGGYAAGTCGGACAACGAGGTCATGCGCTTYTGCCAGTCCTTTATGACYGAGCTCCAGAGGCACGTCGGGGCTGACACCGACGTTCCTGCTGGCGATATTGGCGTCGGCGGTCGCGAGATYGGTTATCTGTTTGGACAGTAYAAGCGYCTCAGGAACGAGTTCACGGGCGTCCTCACGGGCAAGAACATCAAGTGGGGCGGGTCTCTCATCAGRCCAGAGGCCACAGGGTATGGAGCTGTCTACTTCCTGGAGGAGATGT

>Sweh045

-CTCTCGGGCCCTACAAGGGTGGTCTCCGCTTCCACCCCTCTGTCAACCTCTCGATCCTTAAGTTCCTCGGCTTTGAGCAGATCCTGAAGAACTCCCTTACCACGCTCCCAATGGGCGGTGGTAAGGGCGGCTCCGACTTCGATCCTAAGGGCAAGTCGGACAACGAGGTCATGCGCTTTTGCCAGTCCTTTATGACTGAGCTCCAGAGGCACGTCGGGGCTGACACCGACGTTCCTGCTGGCGATATTGGCGTCGGCGGTCGCGAGATTGGTTATCTGTTTGGACAGTACAAGCGTCTCAGGAACGAGTTCACGGGCGTCCTCACGGGCAAGAACATCAAGTGGGGCGGGTCTCTCATCAGGCCAGAGGCCACAGGGTATGGAGCTGTCTACTTCCTGGAGGAGATGT

>Sweh047

GCTCTCGGGCCCTACAAGGGTGGTCTCCGCTTCCACCCCTCTGTCAACCTCTCGATCCTTAAGTTCCTCGGCTTTGAGCAGATCCTGAAGAACTCCCTTACCACGCTCCCGATGGGCGGTGGTAAGGGCGGCTCCGACTTCGATCCTAAGGGCAAGTCGGACAACGAGGTCATGCGCTTCTGCCAGTCCTTTATGACCGAGCTCCAGAGGCACGTCGGGGCTGACACCGACGTTCCTGCTGGCGATATTGGCGTCGGCGGTCGCGAGATCGGTTATCTGTTTGGACAGTATAAGCGCCTCAGGAACGAGTTCACGGGCGTTCTCACGGGCAAGAACATCAAGTGGGGTGGGTCTCTCATCAGACCAGAGGCCACAGGGTATGGAGCTGTCTACTTCCTGGAGGAGATGT

>Sweh048

GCTCTCGGGCCCTACAAGGGTGGTCTCCGCTTCCACCCCTCTGTCAACCTCTCGATCCTTAAGTTCCTCGGCTTTGAGCAGATCCTGAAGAACTCCCTTACCACGCTCCCGATGGGCGGTGGTAAGGGCGGCTCCGACTTCGATCCTAAGGGCAAGTCGGACAACGAGGTCATGCGCTTCTGCCAGTCCTTTATGACCGAGCTCCAGAGGCACGTCGGGGCTGACACCGACGTTCCTGCTGGCGATATTGGCGTCGGCGGTCGCGAGATCGGTTATCTGTTTGGACAGTATAAGCGCCTCAGGAACGAGTTCACGGGCGTTCTCACGGGCAAGAACATCAAGTGGGGTGGGTCTCTCATCAGACCAGAGGCCACAGGGTATGGAGCTGTCTACTTCCTGGAGGAGATGT

>Sweh049

GCTCTCGGGCCCTACAAGGGTGGTCTCCGCTTCCACCCCTCTGTCAACCTCTCGATCCTTAAGTTCCTCGGCTTTGAGCAGATCCTGAAGAACTCCCTTACCACGCTCCCGATGGGCGGTGGTAAGGGCGGCTCCGACTTCGATCCTAAGGGCAAGTCGGACAACGAGGTCATGCGCTTCTGCCAGTCCTTTATGACCGAGCTCCAGAGGCACGTCGGGGCTGACACCGACGTTCCTGCTGGCGATATTGGCGTCGGCGGTCGCGAGATCGGTTATCTGTTTGGACAGTATAAGCGCCTCAGGAACGAGTTCACGGGCGTTCTCACGGGCAAGAACATCAAGTGGGGTGGGTCTCTCATCAGACCAGAGGCCACAGGGTATGGAGCTGTCTACTTCCTGGAGGAGATGT

>Sweh051

GCTCTCGGGCCCTACAAGGGTGGTCTCCGCTTCCACCCCTCTGTCAACCTCTCGATCCTTAAGTTCCTCGGCTTTGAGCAGATCCTGAAGAACTCCCTTACCACGCTTCCGATGGGCGGTGGTAAGGGCGGCTCCGACTTCGATCCTAAGGGCAAGTCGGACAACGAGGTCATGCGCTTCTGCCAGTCCTTTATGACCGAGCTCCAGAGGCACGTCGGGGCTGACACCGACGTTCCTGCTGGCGATATTGGCGTCGGCGGTCGCGAGATCGGTTATCTGTTTGGACAGTATAAGCGCCTCAGGAACGAGTTTACGGGCGTCCTCACGGGCAAGAACATCAAGTGGGGCGGGTCTCTCATCAGACCAGAGGCCACAGGGTATGGAGCTGTCTACTTCCTGGAGGAGATGT

>Sweh056

GCTCTCGGGCCCTACAAGGGTGGTCTCCGCTTCCACCCCTCTGTCAACCTCTCGATCCTTAAGTTCCTCGGCTTTGAGCAGATCCTGAAGAACTCCCTTACCACGCTTCCGATGGGCGGTGGTAAGGGCGGCTCCGACTTCGATCCTAAGGGCAAGTCGGACAACGAGGTCATGCGCTTCTGCCAGTCCTTTATGACCGAGCTCCAGAGGCACGTCGGGGCTGACACCGACGTTCCTGCTGGCGATATTGGCGTCGGCGGTCGCGAGATCGGTTATCTGTTTGGACAGTATAAGCGCCTCAGGAACGAGTTTACGGGCGTCCTCACGGGCAAGAACATCAAGTGGGGCGGGTCTCTCATCAGACCAGAGGCCACAGGGTATGGAGCTGTCTACTTCCTGGAGGAGATGT

>Sweh057

GCTCTCGGGCCCTACAAGGGTGGTCTCCGCTTCCACCCCTCTGTCAACCTCTCGATCCTTAAGTTCCTCGGCTTTGAGCAGATCCTGAAGAACTCCCTTACCACGCTTCCGATGGGCGGTGGTAAGGGCGGCTCCGACTTCGATCCTAAGGGCAAGTCGGACAACGAGGTCATGCGCTTCTGCCAGTCCTTTATGACCGAGCTCCAGAGGCACGTCGGGGCTGACACCGACGTTCCTGCTGGCGATATTGGCGTCGGCGGTCGCGAGATCGGTTATCTGTTTGGACAGTATAAGCGCCTCAGGAACGAGTTTACGGGCGTCCTCACGGGCAAGAACATCAAGTGGGGCGGGTCTCTCATCAGACCAGAGGCCACAGGGTATGGAGCTGTCTACTTCCTGGAGGAGATGT

>Sweh058

GCTCTCGGGCCCTACAAGGGTGGTCTCCGCTTCCACCCCTCTGTCAACCTCTCGATCCTTAAGTTCCTCGGCTTTGAGCAGATCCTGAAGAACTCCCTTACCACGCTTCCGATGGGCGGTGGTAAGGGCGGCTCCGACTTCGATCCTAAGGGCAAGTCGGACAACGAGGTCATGCGCTTCTGCCAGTCCTTTATGACCGAGCTCCAGAGGCACGTCGGGGCTGACACCGACGTTCCTGCTGGCGATATTGGCGTCGGCGGTCGCGAGATCGGTTATCTGTTTGGACAGTATAAGCGCCTCAGGAACGAGTTTACGGGCGTCCTCACGGGCAAGAACATCAAGTGGGGCGGGTCTCTCATCAGACCAGAGGCCACAGGGTATGGAGCTGTCTACTTCCTGGAGGAGATGT

>Sweh059

GCTCTCGGGCCCTACAAGGGTGGTCTCCGCTTCCACCCCTCTGTCAACCTCTCGATCCTTAAGTTCCTCGGCTTTGAGCAGATCCTGAAGAACTCCCTTACCACGCTCCCGATGGGCGGTGGTAAGGGCGGCTCCGACTTCGATCCTAAGGGCAAGTCGGACAACGAGGTCATGCGCTTCTGCCAGTCCTTTATGACCGAGCTCCAGAGGCACGTCGGGGCTGACACCGACGTTCCTGCTGGCGATATTGGCGTCGGCGGTCGCGAGATCGGTTATCTGTTTGGACAGTATAAGCGCCTCAGGAACGAGTTCACGGGCGTTCTCACGGGCAAGAACATCAAGTGGGGTGGGTCTCTCATCAGACCAGAGGCCACAGGGTATGGAGCTGTCTACTTCCTGGAGGAGATGT

>Sweh060

GCTCTCGGGCCCTACAAGGGTGGTCTCCGCTTCCACCCCTCTGTCAACCTCTCGATCCTTAAGTTCCTCGGCTTTGAGCAGATCCTGAAGAACTCCCTTACCACGCTCCCAATGGGCGGTGGTAAGGGCGGCTCCGACTTCGATCCTAAGGGCAAGTCGGACAACGAGGTCATGCGCTTTTGCCAGTCCTTTATGACTGAGCTCCAGAGGCACGTCGGGGCTGACACCGACGTTCCTGCTGGCGATATTGGCGTCGGCGGTCGCGAGATTGGTTATCTGTTTGGACAGTACAAGCGTCTCAGGAACGAGTTCACGGGCGTCCTCACGGGCAAGAACATCAAGTGGGGCGGGTCTCTCATCAGGCCAGAGGCCACAGGGTATGGAGCTGTCTACTTCCTGGAGGAGATGT

>Sweh062

GCTCTCGGGCCCTACAAGGGTGGTCTCCGCTTCCACCCCTCTGTCAACCTCTCGATYCTYAAGTTCCTCGGCTTTGAGCAGATCCTGAAGAACTCCCTTACCACGCTYCCGATGGGCGGTGGTAAGGGCGGCTCCGACTTCGATCCTAAGGGCAAGTCGGACAACGAGGTCATGCGCTTYTGCCAGTCCTTTATGACCGAGCTCCAGAGGCACGTCGGGGCTGACACCGACGTTCCTGCTGGCGATATTGGCGTCGGCGGTCGCGAGATYGGTTATCTGTTTGGACAGTAYAAGCGYCTCAGGAACGAGTTCACGGGCGTCCTCACGGGCAAGAACATCAAGTGGGGCGGGTCTCTCATCAGRCCAGAGGCCACAGGGTATGGAGCTGTCTAYTTCCTGGAGGAGATGT

>Sweh064

GCTCTCGGGCCCTACAAGGGTGGTCTCCGCTTCCACCCCTCTGTCAACCTCTCGATCCTTAAGTTCCTCGGCTTTGAGCAGATCCTGAAGAACTCCCTTACCACGCTYCCRATGGGCGGTGGTAAGGGCGGCTCCGACTTCGATCCTAAGGGCAAGTCGGACAACGAGGTCATGCGCTTCTGCCAGTCCTTTATGAYCGAGCTCCAGAGGCACGTCGGGGCTGACACCGACGTTCCTGCTGGCGATATTGGCGTCGGCGGTCGCGAGATCGGTTATCTGTTTGGACAGTAYAAGCGCCTCAGGAACGAGTTCACGGGCGTCCTCACGGGCAAGAACATCAARTGGGGYGGGTCTCTYATCAGACCAGAGGCCACAGGGTATGGAGCTGTCTACTTCCTGGAGGAGATGT

>Sweh066

GCTCTCGGGCCCTACAAGGGTGGTCTCCGCTTCCACCCCTCTGTCAACCTCTCGATYCTCAAGTTCCTCGGCTTTGAGCAGATCCTGAAGAACTCCCTTACCACGCTYCCGATGGGCGGTGGTAAGGGCGGCTCCGACTTCGATCCTAAGGGCAAGTCGGACAACGAGGTCATGCGCTTYTGYCAGTCCTTTATGACYGAGCTCCAGAGGCACGTCGGGGCTGACACCGACGTTCCTGCTGGCGATATTGGCGTCGGCGGTCGCGAGATYGGTTATCTGTTTGGACAGTAYAAGCGCCTCAGGAACGAGTTCACGGGCGTCCTCACGGGCAAGAACATCAAGTGGGGCGGGTCTCTCATCAGRCCAGAGGCCACAGGGTATGGAGCTGTCTACTTCCTGGAGGAGATGT

>Sweh067

GCTCTCGGGCCCTACAAGGGTGGTCTCCGCTTCCACCCCTCTGTCAACCTCTCGATCCTTAAGTTCCTCGGCTTTGAGCAGATCCTGAAGAACTCCCTTACCACGCTYCCGATGGGCGGTGGTAAGGGCGGCTCCGACTTCGATCCTAAGGGCAAGTCGGACAACGAGGTCATGCGCTTCTGCCAGTCCTTTATGACCGAGCTCCAGAGGCACGTCGGGGCTGACACCGACGTTCCTGCTGGCGATATTGGCGTCGGCGGTCGCGAGATCGGTTATCTGTTTGGACAGTATAAGCGCCTCAGGAACGAGTTYACGGGCGTYCTCACGGGCAAGAACATCAAGTGGGGYGGGTCTCTCATCAGACCAGAGGCCACAGGGTATGGAGCTGTCTACTTCCTGGAGGAGATGT

>Sweh068

GCTCTCGGGCCCTACAAGGGTGGTCTCCGCTTCCACCCCTCTGTCAAYCTCTCGATCCTYAAGTTCCTCGGCTTTGAGCAGATCCTGAAGAACTCCCTTACCACGCTYCCGATGGGCGGTGGTAAGGGCGGCTCCGACTTCGATCCTAAGGGYAAGTCGGACAACGAGGTCATGCGCTTYTGCCAGTCCTTTATGACYGAGCTCCAGAGGCACGTCGGGGCTGACACCGACGTTCCTGCTGGCGATATTGGCGTCGGCGGTCGCGAGATCGGTTATCTGTTTGGACAGTAYAAGCGCCTCAGGAACGAGTTYACGGGCGTYCTCACGGGCAAGAACATCAAGTGGGGYGGGTCTCTCATCAGRCCAGAGGCCACAGGGTATGGAGCTGTCTACTTCCTGGAGGAGATGT

>Sweh069

GCTCTCGGGCCCTACAARGGTGGTCTCCGCTTCCACCCCTCTGTCAACCTCTCGATCCTCAAGTTCCTCGGCTTTGAGCAGATCCTGAAGAACTCCCTTACCACGCTTCCGATGGGCGGTGGTAAGGGCGGCTCCGACTTCGATCCTAAGGGCAAGTCGGACAACGAGGTCATGCGCTTTTGCCAGTCCTTTATGACTGAGCTCCAGAGGCACGTCGGGGCTGACACCGACGTTCCTGCTGGCGATATTGGCGTCGGCGGTCGCGAGATYGGTTATCTGTTTGGACAGTACAAGCGCCTCAGGAACGAGTTCACRGGCGTCCTCACGGGCAAGAACATCAAGTGGGGCGGGTCTCTCATCAGGCCAGAGGCCACAGGGTATGGAGCTGTCTACTTCCTGGAGGAGATGT

>Sweh074

GCTCTCGGGCCCTACAAGGGTGGTCTCCGCTTCCACCCCTCTGTCAACCTCTCGATCCTTAAGTTCCTCGGCTTTGAGCAGATCCTGAAGAACTCCCTTACCACGCTTCCGATGGGCGGTGGTAAGGGCGGCTCCGACTTCGATCCTAAGGGCAAGTCGGACAACGAGGTCATGCGCTTCTGCCAGTCCTTTATGACCGAGCTCCAGAGGCACGTCGGGGCTGACACCGACGTTCCTGCTGGCGATATTGGCGTCGGCGGTCGCGAGATCGGTTATCTGTTTGGACAGTATAAGCGCCTCAGGAACGAGTTTACGGGCGTCCTCACGGGCAAGAACATCAAGTGGGGCGGGTCTCTCATCAGACCAGAGGCCACAGGGTATGGAGCTGTCTACTTCCTGGAGGAGATGT

>Sweh075

GCTCTCGGGCCCTACAAGGGTGGTCTCCGCTTCCACCCCTCTGTCAACCTCTCGATCCTYAAGTTCCTCGGCTTTGAGCAGATCCTGAAGAACTCCCTTACCACGCTYCCGATGGGCGGTGGTAAGGGCGGCTCCGACTTYGATCCTAAGGGCAARTCGGACAACGARGTCATGCGCTTCTGCCAGTCCTTTATGACYGAGCTCCAGAGGCACGTCGGGGCTGACACCGACGTTCCTGCTGGCGATATTGGCGTCGGCGGTCGCGAGATCGGTTATCTGTTTGGRCAGTAYAAGCGCCTCAGGAACGAGTTCACGGGCRTYCTCACGGGCAAGAACATCAAGTGGGGCGGGTCTCTYATCAGACCAGAGGCCACAGGGTATGGAGCYGTCTACTTCCTGGAGGAGATGT

>Sweh076

GCTCTCGGGCCCTACAAGGGTGGTCTCCGCTTCCACCCCTCTGTCAAYCTCTCGATCCTCAAGTTCCTCGGCTTTGAGCAGATCCTGAAGAACTCCCTTACCACGCTYCCRATGGGCGGTGGTAAGGGCGGCTCCGACTTCGATCCTAAGGGCAAGTCGGACAACGAGGTCATGCGCTTTTGCCAGTCCTTTATGACTGAGCTCCAGAGGCACGTYGGGGCTGACACCGACGTTCCTGCTGGCGATATTGGCGTCGGCGGTCGCGAGATYGGTTATCTGTTTGGACARTACAAGCGYCTCAGGAACGAGTTCACGGGCGTCCTCACGGGCAAGAACATCAAGTGGGGCGGGTCTCTCATCAGGCCAGAGGCCACAGGGTATGGAGCTGTCTACTTCCTGGAGGAGATGT

>Sweh079

GCTCTCGGGCCCTACAAGGGTGGTCTCCGCTTCCACCCCTCTGTCAACCTCTCGATCCTYAAGTTCCTCGGCTTTGAGCAGATCCTGAAGAACTCCCTTACCACGCTYCCGATGGGCGGTGGTAAGGGCGGCTCCGACTTCGATCCTAAGGGCAAGTCGGACAACGAGGTCATGCGCTTYTGCCAGTCCTTTATGACYGAGCTCCAGAGGCACGTCGGGGCTGACACCGACGTTCCYGCTGGCGATATTGGCGTCGGCGGTCGCGAGATYGGTTATCTGTTTGGACAGTAYAAGCGYCTCAGGAACGAGTTCACGGGCGTYCTCACGGGCAAGAACATCAAGTGGGGCGGGTCTCTCATCAGRCCAGAGGCCACAGGGTATGGAGCYGTCTACTTCCTGGAGGAGATGT

>Sweh081

GCTCTCGGGCCCTACAAGGGTGGTCTCCGCTTCCACCCCTCTGTCAACCTCTCGATCCTYAAGTTCCTCGGCTTTGAGCAGATCCTGAAGAACTCCCTTACCACGCTCCCAATGGGCGGTGGTAAGGGCGGCTCCGACTTCGATCCTAAGGGCAAGTCGGACAACGAGGTCATGCGCTTTTGCCAGTCCTTTATGACYGAGCTCCAGAGGCACGTCGGGGCTGACACCGACGTTCCTGCTGGCGATATTGGCGTCGGCGGTCGCGAGATYGGTTATCTGTTTGGACAGTAYAAGCGYCTCAGGAACGAGTTCACGGGCGTYCTCACGGGMAAGAACATCAAGTGGGGCGGGTCTCTCATCAGRCCAGAGGCCACAGGGTATGGAGCTGTCTACTTCCTGGAGGAGATGT

>Sweh082

GCTCTCGGGCCCTACAAGGGTGGTCTCCGCTTCCACCCCTCTGTCAACCTCTCGATCCTTAAGTTCCTCGGCTTTGAGCAGATCCTGAAGAACTCCCTTACCACGCTCCCGATGGGCGGTGGTAAGGGCGGCTCCGACTTCGATCCTAAGGGCAAGTCGGACAACGAGGTCATGCGCTTCTGCCAGTCCTTTATGACCGAGCTCCARAGGCACGTCGGGGCTGACACCGACGTTCCTGCTGGCGATATTGGCGTCGGCGGTCGCGAGATYGGTTATCTGTTTGGACAGTAYAAGCGCCTCAGGAACGAGTTCACGGGCGTCCTCACGGGCAAGAACATCAAGTGGGGCGGGTCTCTCATCAGRCCAGAGGCCACAGGGTATGGAGCTGTCTACTTCCTGGAGGAGATGT

>Sweh083

GCTCTCGGGCCCTACAAGGGTGGTCTCCGCTTCCACCCCTCTGTCAACCTCTCGATCCTTAAGTTCCTCGGCTTTGAGCAGATCCTGAAGAACTCCCTTACCACGCTCCCGATGGGCGGTGGTAAGGGCGGCTCCGACTTCGATCCTAAGGGCAAGTCGGACAACGAGGTCATGCGCTTCTGCCAGTCCTTTATGACCGAGCTCCAGAGGCACGTCGGGGCTGACACCGACGTTCCTGCTGGCGATATTGGCGTCGGCGGTCGCGAGATCGGTTATCTGTTTGGACAGTATAAGCGCCTCAGGAACGAGTTCACGGGCGTTCTCACGGGCAAGAACATCAAGTGGGGTGGGTCTCTCATCAGACCAGAGGCCACAGGGTATGGAGCTGTCTACTTCCTGGAGGAGATGT

>Sweh084

GCTCTCGGGCCCTACAAGGGTGGTCTCCGCTTCCACCCCTCTGTCAACCTCTCGATCCTTAAGTTCCTCGGCTTTGAGCAGATCCTGAAGAACTCCCTTACCACGCTYCCGATGGGCGGTGGTAAGGGCGGCTCCGACTTCGATCCTAAGGGCAAGTCGGACAACGAGGTCATGCGCTTCTGCCAGTCCTTTATGACCGAGCTCCAGAGGCACGTCGGGGCTGACACCGACGTTCCTGCTGGCGATATTGGCGTCGGCGGTCGCGAGATCGGTTATCTGTTTGGACAGTATAAGCGCCTCAGGAACGAGTTYACGGGCGTCCTCACGGGCAAGAACATCAAGTGGGGCGGGTCTCTCATCAGACCAGAGGCCACAGGGTATGGAGCTGTCTACTTCCTGGAGGAGATGT

>Sweh086

GCTCTCGGGCCCTACAAGGGTGGTCTCCGCTTCCACCCCTCTGTCAACCTCTCGATCCTYAAGTTCCTCGGCTTTGAGCAGATCCTGAAGAACTCCCTTACCACGCTYCCGATGGGCGGTGGTAAGGGCGGCTCYGACTTCGATCCTAAGGGCAAGTCGGACAACGAGGTCATGCGCTTYTGCCAGTCCTTTATGACYGAGCTCCAGAGGCACGTCGGGGCTGACACCGACGTTCCTGCTGGCGATATTGGCGTCGGCGGTCGCGAGATYGGTTATCTGTTTGGACAGTAYAAGCGCCTCAGGAACGAGTTYACGGGCGTCCTCACGGGCAAGAACATCAAGTGGGGCGGGTCTCTCATCAGRCCAGAGGCCACAGGGTATGGAGCYGTCTACTTCCTGGAGGAGATGT

>Sweh088

GCTCTCGGGCCCTACAAGGGTGGTCTCCGCTTCCACCCCTCTGTCAACCTCTCGATCCTCAAGTTCCTCGGCTTTGAGCAGATCCTGAAGAACTCCCTTACCACGCTTCCGATGGGCGGTGGTAAGGGCGGCTCCGACTTCGATCCTAAGGGCAAGTCGGACAACGAGGTCATGCGCTTTTGCCAGTCCTTTATGACTGAGCTCCAGAGGCACGTCGGGGCTGACACCGACGTTCCTGCTGGCGATATTGGCGTCGGCGGTCGCGAGATCGGTTATCTGTTTGGACAGTATAAGCGCCTCAGGAACGAGTTTACGGGCGTCCTCACGGGCAAGAACATCAAGTGGGGCGGGTCTCTCATCAGACCAGAGGCCACAGGGTATGGAGCTGTCTACTTCCTGGAGGAGATGT

>Sweh089

GCTCTCGGGCCCTACAAGGGTGGTCTCCGCTTCCACCCCTCTGTCAACCTCTCGATCCTCAAGTTCCTCGGCTTTGAGCAGATCCTGAAGAACTCCCTTACCACGCTYCCRATGGGCGGTGGTAAGGGCGGCTCCGACTTCGATCCTAAGGGCAAGTCGGACAACGAGGTCATGCGCTTYTGCCAGTCCTTTATGACYGAGCTCCAGAGGCACGTCGGGGCTGACACCGACGTTCCTGCTGGCGATATTGGCGTCGGCGGTCGCGAGATYGGTTATCTGTTTGGACAGTAYAAGCGCCTCAGGAACGAGTTYACGGGCGTYCTCACGGGCAAGAACATCAAGTGGGGCGGGTCTCTCATCAGRCCAGAGGCCACAGGGTATGGAGCTGTCTACTTCCTGGAGGAGATGT

>Sweh090

GCTCTCGGGCCCTACAAGGGTGGTCTCCGCTTCCACCCCTCTGTCAAYCTCTCGATCCTYAAGTTCCTCGGCTTTGAGCAGATCCTGAAGAACTCCCTTACCACGCTYCCRATGGGCGGTGGTAAGGGCGGCTCCGACTTCGATCCTAAGGGCAAGTCGGACAACGAGGTCATGCGCTTYTGCCAGTCCTTTATGACYGAGCTCCAGAGGCACGTCGGGGCTGACACCGACGTTCCTGCTGGCGATATTGGCGTCGGCGGTCGCGAGATYGGTTATCTGTTTGGACAGTAYAAGCGYCTCAGGAACGAGTTYACGGGCGTCCTCACGGGCAAGAACATCAAGTGGGGCGGGTCTCTCATCAGGCCAGAGGCCACAGGGTATGGAGCTGTCTACTTCCTGGAGGAGATG-

>Sweh091

GCTCTCGGGCCCTACAAGGGTGGTCTCCGCTTCCACCCCTCTGTCAACCTCTCGATCCTTAAGTTCCTCGGCTTTGAGCAGATCCTGAAGAACTCCCTTACCACGCTCCCGATGGGCGGTGGTAAGGGCGGCTCCGACTTCGATCCTAAGGGCAAGTCGGACAACGAGGTCATGCGCTTCTGCCAGTCCTTTATGACYGAGCTCCAGAGGCACGTCGGGGCTGACACCGACGTTCCTGCTGGCGATATTGGCGTCGGCGGTCGCGAGATYGGTTATCTGTTTGGACAGTAYAAGCGYCTCAGGAACGAGTTYACGGGCGTCCTCACGGGCAAGAACATCAAGTGGGGCGGGTCTCTCATCAGRCCAGAGGCCACAGGGTATGGAGCTGTCTACTTCCTGGAGGAGATGT

>Sweh092

GCTCTCGGGCCCTACAAGGGTGGTCTCCGCTTCCACCCCTCTGTCAACCTCTCGATCCTCAAGTTCCTCGGCTTTGAGCAGATCCTGAAGAACTCCCTTACCACGCTTCCRATGGGCGGTGGTAAGGGCGGCTCYGACTTCGATCCTAAGGGCAAGTCRGACAACGAGGTCATGCGCTTTTGCCAGTCCTTTATGACTGAGCTCCAGAGGCACGTCGGGGCTGACACCGACGTTCCTGCTGGCGATATTGGCGTCGGCGGTCGCGAGATYGGTTATCTGTTTGGACAGTAYAAGCGCCTCAGGAACGAGTTYACGGGCGTCCTCACGGGCAAGAACATCAAGTGGGGCGGGTCTCTCATCAGRCCAGAGGCCACAGGGTATGGAGCYGTCTACTTCCTGGAGGAGATGT

>Sweh093

GCTCTCGGGCCCTACAAGGGTGGTCTCCGCTTCCACCCCTCTGTCAAYCTCTCGATCCTYAAGTTCCTCGGCTTTGAGCAGATCCTGAAGAACTCCCTTACCACGCTYCCGATGGGCGGTGGTAAGGGCGGCTCCGACTTCGATCCTAAGGGCAAGTCGGACAACGAGGTCATGCGCTTYTGCCAGTCCTTTATGACYGAGCTCCAGAGGCACGTCGGGGCTGACACCGACGTTCCTGCTGGCGATATTGGCGTCGGCGGTCGCGAGATCGGTTATCTGTTTGGACAGTAYAAGCGCCTCAGGAACGAGTTCACGGGCGTCCTCACGGGCAAGAACATCAAGTGGGGCGGGTCTCTCATCAGRCCAGAGGCCACAGGGTATGGAGCTGTCTACTTCCTGGAGGAGATGT

>Sweh094

-CTCTCGGGCCCTACAAGGGTGGTCTCCGCTTCCACCCCTCTGTCAACCTCTCGATCCTYAAGTTCCTCGGCTTTGAGCAGATCCTGAAGAACTCCCTTACCACGCTYCCRATGGGCGGTGGTAAGGGCGGCTCCGACTTCGATCCTAAGGGCAAGTCGGACAACGAGGTCATGCGCTTTTGCCAGTCCTTTATGACTGAGCTCCAGAGGCACGTCGGGGCTGACACCGACGTTCCYGCTGGCGATATTGGCGTCGGCGGTCGCGAGATTGGTTATCTGTTTGGACAGTACAAGCGYCTCAGGAACGAGTTCACGGGCGTCCTCACGGGCAAGAACATCAAGTGGGGCGGGTCTCTCATCAGGCCAGAGGCCACAGGGTATGGAGCTGTCTACTTCCTGGAGGAGATGT

>Sweh095

GCTCTCGGGCCCTACAAGGGTGGTCTCCGCTTCCACCCCTCTGTCAAYCTCTCGATCCTYAAGTTCCTCGGCTTTGAGCAGATCCTGAAGAACTCCCTTACCACGCTYCCGATGGGCGGTGGTAAGGGCGGCTCCGACTTCGATCCTAAGGGCAAGTCGGACAAYGAGGTCATGCGCTTYTGCCAGTCCTTTATGACYGAGCTCCAGAGGCACGTCGGGGCTGACACCGACGTTCCTGCTGGCGATATTGGCGTCGGCGGTCGCGAGATYGGTTATCTGTTTGGACAGTAYAAGCGCCTCAGGAACGAGTTCACGGGCGTCCTCACGGGCAAGAACATCAAGTGGGGYGGGTCTCTCATCAGRCCAGAGGCCACRGGGTATGGAGCTGTCTACTTCCTGGAGGAGATG-

>Sweh102

GCTCTCGGGCCCTACAAGGGTGGTCTCCGCTTCCACCCCTCTGTCAACCTCTCGATCCTTAAGTTCCTCGGCTTTGAGCAGATCCTGAAGAACTCCCTTACCACGCTCCCGATGGGCGGTGGTAAGGGCGGCTCCGACTTCGATCCTAAGGGCAAGTCGGACAACGAGGTCATGCGCTTCTGCCAGTCCTTTATGACCGAGCTCCAGAGGCACGTCGGGGCTGACACCGACGTTCCTGCTGGCGATATTGGCGTCGGCGGTCGCGAGATCGGTTATCTGTTTGGACAGTATAAGCGCCTCAGGAACGAGTTCACGGGCGTTCTCACGGGCAAGAACATCAAGTGGGGTGGGTCTCTCATCAGACCAGAGGCCACAGGGTATGGAGCTGTCTACTTCCTGGAGGAGATG

>Sweh103

GCTCTCGGGCCCTACAAGGGTGGTCTCCGCTTCCACCCCTCTGTCAACCTCTCGATCCTCAAGTTCCTCGGCTTTGAGCAGATCCTGAAGAAYTCCCTTACCACGCTTCCGATGGGCGGTGGTAAGGGCGGCTCCGACTTCGATCCTAAGGGCAAGTCGGACAAYGAGGTCATGCGCTTTTGCCAGTCCTTTATGACTGAGCTCCAGAGGCACGTCGGGGCTGACACCGACGTTCCTGCTGGCGATATTGGCGTCGGCGGTCGCGAGATTGGTTATCTGTTTGGACAGTACAAGCGCCTCAGGAACGAGTTCACRGGCGTCCTCACGGGCAAGAACATCAAGTGGGGCGGGTCTCTCATCAGGCCAGAGGCCACAGGGTATGGAGCTGTCTACTTCCTGGAGGAGAT--

>Sweh105

GCTCTCGGGCCCTACAAGGGTGGTCTCCGCTTCCACCCCTCTGTCAACCTCTCGATCCTTAAGTTCCTCGGCTTTGAGCAGATCCTGAAGAACTCCCTTACCACGCTCCCGATGGGCGGTGGTAAGGGCGGCTCCGACTTCGATCCTAAGGGCAAGTCGGACAACGAGGTCATGCGCTTCTGCCAGTCCTTTATGACCGAGCTCCAGAGGCACGTCGGGGCTGACACCGACGTTCCTGCTGGCGATATTGGCGTCGGCGGTCGCGAGATCGGTTATCTGTTTGGACAGTATAAGCGCCTCAGGAACGAGTTTACGGGCGTCCTCACGGGCAAGAACATCAAGTGGGGCGGGTCTCTCATCAGACCAGAGGCCACAGGGTATGGAGCTGTCTACTTCCTGGAGGAGATGT

>Sweh106

-CTCTCGGGCCCTACAAGGGTGGTCTCCGCTTCCACCCCTCTGTCAACCTCTCGATCCTCAAGTTCCTCGGCTTTGAGCAGATCCTGAAGAACTCCCTTACCACGCTCCCGATGGGCGGTGGTAAGGGCGGCTCCGACTTCGATCCTAAGGGCAAGTCGGACAACGAGGTCATGCGCTTCTGCCAGTCCTTTATGACCGAGCTCCAGAGGCACGTCGGRGCTGACACCGACGTTCCTGCTGGCGATATTGGCGTCGGCGGTCGCGAGATCGGTTATCTGTTTGGACAGTATAAGCGCCTCAGGAACGAGTTCACGGGCGTCCTCACGGGCAAGAACATCAAGTGGGGCGGGTCTCTCATCAGRCCAGAGGCCACAGGGTATGGAGCTGTCTACTTCCTGGAGGAGATG-

>Sweh107

GCTCTCGGGCCCTACAAGGGTGGTCTCCGCTTCCACCCCTCTGTCAACCTCTCGATCCTCAAGTTCCTCGGCTTTGAGCAGATCCTGAAGAACTCCCTTACCACGCTTCCGATGGGCGGTGGTAAGGGCGGCTCCGACTTCGATCCTAAGGGCAAGTCGGACAACGAGGTCATGCGCTTTTGCCAGTCCTTTATGACTGAGCTCCAGAGGCACGTCGGGGCTGACACCGACGTTCCTGCTGGCGATATTGGCGTCGGCGGTCGCGAGATCGGTTATCTGTTTGGACAGTACAAGCGCCTCAGGAACGAGTTCACGGGCGTCCTCACGGGCAAGAACATCAAGTGGGGCGGGTCTCTCATCAGGCCAGAGGCCACAGGGTATGGAGCTGTCTACTTCCTGGAGGAGATGT

>Sweh111

GCTCTCGGGCCCTACAAGGGTGGTCTCCGCTTCCACCCCTCTGTCAACCTCTCGATCCTTAAGTTCCTCGGCTTTGAGCAGATCCTGAAGAACTCCCTTACCACGCTCCCRATGGGCGGTGGTAAGGGCGGCTCCGACTTCGATCCTAAGGGCAAGTCGGACAACGAGGTCATGCGCTTYTGCCAGTCCTTTATGACTGAGCTCCAGAGGCACGTCGGGGCTGACACCGACGTTCCTGCTGGCGATATTGGCGTCGGCRGTCGCGAGATYGGTTATCTGTTTGGACAGTAYAAGCGYCTCAGGAACGAGTTCACGGGCGTCCTCACGGGCAAGAACATCAAGTGGGGCGGGTCTCTCATCAGRCCAGAGGCCACAGGGTATGGAGCYGTCTACTTCCTGGAGGAGATGT

>Sweh112

-CTCTCGGGCCCTACAAGGGTGGTCTCCGCTTCCACCCCTCTGTCAACCTCTCGATCCTCAAGTTCCTCGGCTTTGAGCAGATCCTGAAGAACTCCCTTACCACGCTTCCGATGGGCGGTGGTAAGGGCGGCTCCGACTTCGATCCTAAGGGCAAGTCGGACAACGAGGTCATGCGCTTTTGCCAGTCCTTTATGACTGAGCTCCAGAGGCACGTCGGGGCTGACACCGACGTTCCTGCTGGCGATATTGGCGTCGGCGGTCGCGAGATTGGTTATCTGTTTGGACAGTACAAGCGTCTCAGGAACGAGTTCACGGGCGTCCTCACGGGCAAGAACATCAAGTGGGGCGGGTCTCTCATCAGACCAGAGGCCACAGGGTATGGAGCTGTCTACTTCCTGGAGGAGATGT

>Sweh113

GCTCTCGGGCCCTACAAGGGTGGTCTCCGCTTCCACCCCTCTGTCAACCTCTCGATCCTYAAGTTCCTCGGCTTTGAGCAGATCCTGAAGAACTCCCTTACCACGCTYCCGATGGGCGGTGGTAAGGGCGGCTCCGACTTCGATCCTAAGGGCAAGTCGGACAACGAGGTCATGCGCTTTTGCCAGTCCTTTATGACYGAGCTCCAGAGGCACGTCGGGGCTGACACCGACGTTCCTGCTGGCGATATTGGYGTCGGCGGTCGCGAGATYGGTTATCTGTTTGGACAGTAYAAGCGCCTCAGGAACGAGTTCACGGGCGTCCTCACGGGCAAGAACATCAAGTGGGGCGGGTCTCTCATCAGRCCAGAGGCCACAGGGTATGGAGCTGTCTACTTCCTGGAGGAGATGT

>Sweh115

GCTCTCGGGCCCTACAAGGGTGGTCTCCGCTTCCACCCCTCTGTCAACCTCTCGATCCTYAAGTTCCTCGGCTTTGAGCAGATCCTGAAGAACTCCCTTACCACGCTYCCRATGGGCGGTGGTAAGGGCGGCTCCGACTTCGATCCTAAGGGCAAGTCGGACAACGAGGTCATGCGCTTTTGCCAGTCCTTTATGACYGAGCTCCAGAGGCACGTCGGGGCTGACACCGACGTTCCTGCTGGCGATATTGGCGTCGGCGGTCGCGAGATYGGTTATCTGTTTGGACAGTAYAAGCGYCTCAGGAACGAGTTCACGGGCGTYCTCACGGGMAAGAACATCAAGTGGGGCGGGTCTCTCATCAGGCCAGAGGCCACAGGGTATGGAGCTGTCTACTTCCTGGAGGAGATGT

>Sweh116

GCTCTCGGRCCCTACAAGGGTGGTCTCCGCTTCCACCCCTCTGTCAAYCTCTCGATCCTYAAGTTCCTCGGCTTTGAGCAGATCCTGAAGAACTCCCTTACCACGCTYCCGATGGGCGGTGGTAAGGGCGGCTCCGACTTCGATCCTAAGGGCAAGTCGGACAACGAGGTCATGCGCTTYTGCCAGTCCTTTATGACYGAGCTCCAGAGGCACGTCGGGGCTGACACCGACGTTCCTGCTGGCGATATTGGCGTCGGCGGTCGCGAGATYGGTTATCTGTTTGGACAGTACAAGCGCCTCAGGAACGAGTTCACGGGCGTCCTCACGGGCAAGAACATCAAGTGGGGCGGGTCTCTCATCAGGCCAGAGGCCACAGGGTATGGAGCTGTCTACTTCCTGGAGGAGATGT

>Sweh117

GCTCTCGGGCCCTACAAGGGTGGTCTCCGCTTCCACCCCTCTGTCAACCTCTCGATCCTTAAGTTCCTCGGCTTTGAGCAGATCCTGAAGAACTCCCTTACCACGCTCCCRATGGGCGGTGGTAAGGGCGGCTCCGACTTCGATCCTAAGGGCAAGTCGGACAACGAGGTCATGCGCTTYTGCCAGTCCTTTATGACTGAGCTCCAGAGGCACGTCGGGGCTGACACCGACGTTCCTGCTGGCGATATTGGCGTCGGCRGTCGCGAGATYGGTTATCTGTTTGGACAGTAYAAGCGYCTCAGGAACGAGTTCACGGGCGTCCTCACGGGCAAGAACATCAAGTGGGGCGGGTCTCTCATCAGRCCAGAGGCCACAGGGTATGGAGCYGTCTACTTCCTGGAGGAGATGT

>Sweh118

GCTCTCGGGCCCTACAAGGGTGGTCTCCGYTTCCACCCCTCTGTCAACCTCTCGATCCTYAAGTTCCTCGGCTTTGAGCAGATCCTGAAGAACTCCCTTACCACGCTYCCGATGGGCGGTGGTAAGGGCGGCTCCGACTTCGATCCTAAGGGCAAGTCGGACAACGAGGTCATGCGCTTTTGCCAGTCCTTTATGACYGAGCTCCAGAGGCACGTCGGGGCTGACACCGACGTTCCTGCTGGCGATATTGGCGTCGGCGGTCGCGAGATYGGTTATCTGTTTGGACAGTAYAAGCGCCTCAGGAACGAGTTCACGGGCGTCCTCACGGGCAAGAACATCAAGTGGGGCGGGTCTCTCATCAGRCCAGAGGCCACAGGGTATGGAGCYGTCTACTTCCTGGAGGAGATGT

>Sweh119

GCTCTCGGGCCCTACAAGGGTGGTCTCCGCTTCCACCCCTCTGTCAAYCTCTCGATCCTYAAGTTCCTCGGCTTTGAGCAGATCCTGAAGAACTCCCTTACCACGCTYCCRATGGGCGGTGGTAAGGGCGGCTCCGACTTCGATCCTAAGGGCAAGTCGGACAACGAGGTCATGCGCTTYTGCCAGTCCTTTATGACTGAGCTCCAGAGGCACGTCGGRGCTGACACCGACGTTCCTGCTGGCGATATTGGCGTCGGCGGTCGCGAGATCGGTTATCTGTTTGGACAGTAYAAGCGCCTCAGGAACGAGTTCACGGGCGTCCTCACGGGCAAGAACATCAAGTGGGGCGGGTCTCTCATCAGRCCAGAGGCCACAGGGTATGGAGCTGTCTACTTCCTGGAGGAGATGT

>Sweh121

--TCTCGGGCCCTACAAGGGTGGTCTCCGCTTCCACCCCTCTGTCAACCTCTCGATCCTYAAGTTCCTCGGCTTTGAGCAGATCCTGAAGAACTCCCTTACCACGCTYCCGATGGGCGGTGGTAAGGGCGGCTCCGACTTYGATCCCAAGGGCAAGTCGGACAACGAGGTCATGCGCTTYTGCCAGTCCTTTATGACYGAGCTCCAGAGGCACGTCGGGGCTGACACCGACGTTCCTGCTGGCGATATTGGCGTCGGCGGTCGCGAGATYGGTTATCTGTTTGGACAGTAYAAGCGYCTCAGGAACGAGTTCACGGGCGTYCTCACGGGCAAGAACATCAAGTGGGGCGGGTCTCTYATCAGRCCAGAGGCCACAGGGTATGGAGCTGTCTACTTCCTGGAGGAGATGT

>Sweh123

GCTCTCGGGCCCTACAAGGGTGGTCTCCGCTTCCACCCCTCTGTCAACCTCTCGATCCTYAAGTTCCTCGGCTTTGAGCAGATCCTGAAGAACTCCCTTACCACGCTTCCGATGGGCGGTGGTAAGGGCGGCTCCGACTTCGATCCTAAGGGCAAGTCGGACAACGAGGTCATGCGCTTYTGCCAGTCCTTTATGACYGAGCTCCAGAGGCACGTCGGGGCTGACACCGACGTTCCTGCTGGCGATATTGGCGTCGGCGGTCGCGAGATCGGTTATCTGTTTGGACAGTAYAAGCGCCTCAGGAACGAGTTCACGGGCGTYCTCACGGGCAAGAACATCAAGTGGGGCGGGTCTCTCATCAGRCCAGAGGCCACAGGGTATGGAGCTGTCTACTTCCTGGAGGAGATGC

>Sweh124

-CTCTCGGGCCCTACAAGGGTGGTCTCCGCTTCCACCCCTCTGTCAAYCTCTCGATCCTCAAGTTCCTCGGCTTTGAGCAGATCCTGAAGAACTCCCTTACCACGCTCCCRATGGGCGGTGGTAAGGGCGGCTCCGACTTCGATCCTAAGGGCAAGTCGGACAACGAGGTCATGCGCTTYTGCCAGTCCTTTATGACYGAGCTCCAGAGGCACGTCGGGGCTGACACCGACGTTCCTGCTGGCGATATTGGCGTCGGCGGTCGCGAGATCGGTTATCTGTTTGGACAGTACAAGCGCCTCAGGAACGAGTTCACGGGCGTCCTCACGGGCAAGAACATCAAGTGGGGCGGGTCTCTCATCAGGCCAGAGGCCACAGGGTATGGAGCTGTCTACTTCCTGGAGGAGATGT

>Sweh126

GCTCTCGGGCCCTACAARGGTGGTCTCCGCTTCCACCCCTCTGTCAAYCTCTCGATCCTYAAGTTCCTCGGCTTTGAGCAGATCCTGAAGAACTCCCTTACCACGCTCCCRATGGGCGGTGGTAAGGGCGGCTCCGACTTCGATCCTAAGGGCAAGTCGGACAACGAGGTCATGCGCTTYTGCCAGTCCTTTATGACYGAGCTCCAGAGGCACGTCGGGGCTGACACCGACGTTCCTGCTGGCGATATTGGCGTCGGCGGTCGCGAGATYGGTTATCTGTTTGGACAGTAYAAGCGYCTCAGGAACGAGTTYACGGGCGTCCTCACGGGCAAGAACATCAAGTGGGGCGGGTCTCTCATCAGACCAGAGGCCACAGGGTATGGAGCTGTCTACTTCCTGGAGGAGATGT

>Sweh127

GCTCTCGGGCCCTACAAGGGTGGTCTCCGCTTCCACCCCTCTGTCAACCTCTCGATCCTYAAGTTCCTCGGCTTTGAGCAGATCCTGAARAACTCCCTTACCACGCTCCCRATGGGCGGTGGTAAGGGCGGCTCCGACTTCGATCCTAAGGGCAAGTCGGACAACGAGGTCATGCGCTTYTGCCAGTCCTTTATGACYGAGCTCCAGAGGCACGTCGGGGCTGACACCGACGTTCCTGCTGGCGATATTGGCGTCGGCGGTCGCGAGATCGGTTATCTGTTTGGACAGTAYAAGCGYCTCAGGAACGAGTTCACGGGCGTYCTCACGGGCAAGAACATCAAGTGGGGCGGGTCTCTCATCAGRCCAGAGGCCACAGGGTATGGAGCTGTCTACTTCCTGGAGGAGAT--

>Sweh128

GCTCTCGGGCCCTACAAGGGTGGTCTCCGCTTCCACCCCTCTGTCAAYCTCTCGATCCTYAAGTTCCTCGGCTTTGAGCAGATCCTGAAGAACTCYCTTACCACGCTYCCRATGGGCGGTGGTAAGGGCGGCTCCGACTTCGATCCTAAGGGCAAGTCGGACAACGAGGTCATGCGCTTYTGCCAGTCCTTTATGACYGAGCTCCAGAGGCACGTCGGRGCTGACACCGACGTTCCTGCTGGCGATATTGGCGTCGGCGGTCGCGAGATYGGTTATCTGTTTGGACAGTAYAAGCGCCTCAGGAACGAGTTYACGGGCGTCCTCACGGGCAAGAACATCAAGTGGGGCGGGTCTCTYATCAGRCCAGAGGCCACAGGRTATGGAGCTGTCTACTTCCTGGAGGAGATGT

>Sweh136

GCTCTCGGGCCCTACAAGGGTGGTCTCCGCTTCCACCCCTCTGTCAACCTCTCGATCCTTAAGTTCCTCGGCTTTGAGCAGATCCTGAAGAACTCCCTTACCACGCTTCCGATGGGCGGTGGTAAGGGCGGCTCCGACTTCGATCCTAAGGGCAAGTCGGACAACGAGGTCATGCGCTTTTGCCAGTCCTTTATGACTGAGCTCCAGAGGCACGTCGGGGCTGACACCGACGTTCCTGCTGGCGATATTGGCGTCGGCGGTCGCGAGATTGGTTATCTGTTTGGACAGTACAAGCGTCTCAGGAACGAGTTCACGGGCGTCCTCACGGGCAAGAACATCAAGTGGGGCGGGTCTCTCATCAGGCCAGAGGCCACAGGGTATGGAGCTGTCTACTTCCTGGAGGAGATGT

>Sweh137

GCTCTCGGGCCCTACAAGGGTGGTCTCCGCTTCCACCCCTCTGTCAACCTCTCGATCCTYAAGTTCCTCGGCTTTGAGCAGATCCTGAAGAACTCCCTTACCACGCTYCCGATGGGCGGTGGTAARGGCGGCTCCGACTTCGATCCTAAGGGCAAGTCGGACAACGAGGTCATGCGCTTYTGCCAGTCCTTTATGACYGAGCTCCAGAGGCACGTCGGGGCTGACACCGACGTTCCTGCTGGCGATATTGGCGTCGGCGGTCGCGAGATYGGTTATCTGTTTGGACAGTAYAAGCGCCTCAGGAACGAGTTYACRGGCGTCCTCACGGGCAAGAACATCAAGTGGGGCGGGTCTCTCATCAGRCCAGAGGCCACAGGGTATGGAGCTGTCTAYTTCCTGGAGGAGATGT

>Sweh141

GCTCTCGGGCCCTACAAGGGTGGTCTCCGCTTCCACCCCTCTGTCAACCTCTCGATCCTYAAGTTCCTCGGCTTTGAGCAGATCCTGAAGAACTCCCTTACCACGCTYCCGATGGGCGGTGGTAAGGGCGGCTCCGACTTCGATCCTAAGGGCAAGTCGGACAACGAGGTCATGCGCTTYTGCCAGTCCTTTATGACTGAGCTCCAGAGGCACGTCGGGGCTGACACCGACGTTCCTGCTGGCGATATTGGCGTCGGCGGTCGCGAGATYGGTTATCTGTTTGGACAGTAYAAGCGCCTCAGGAACGAGTTCACRGGCGTCCTCACGGGCAAGAACATCAAGTGGGGCGGGTCTCTCATCAGRCCAGAGGCCACAGGGTATGGAGCTGTCTACTTCCTGGAGGAGATGT

>Sweh142

GCTCTCGGGCCCTACAAGGGTGGTCTCCGCTTCCACCCCTCTGTCAACCTCTCGATCCTYAAGTTCCTCGGCTTTGAGCAGATCCTGAAGAACTCYCTTACCACGCTCCCRATGGGCGGTGGTAAGGGCGGCTCCGACTTCGATCCTAAGGGCAAGTCGGACAACGAGGTCATGCGCTTYTGCCAGTCCTTTATGACYGAGCTCCAGAGGCACGTCGGGGCTGACACCGACGTTCCTGCTGGCGATATTGGCGTCGGCGGTCGCGAGATYGGTTATCTGTTTGGACAGTAYAAGCGYCTCAGGAACGAGTTCACGGGCGTYCTCACGGGCAAGAACATCAAGTGGGGCGGGTCTCTCATCAGRCCAGAGGCCACAGGGTATGGAGCTGTCTACTTCCTGGAGGAGATGT

>Sweh143

GCTCTCGGGCCCTACAAGGGTGGTCTCCGCTTCCACCCCTCTGTCAAYCTCTCGATCCTCAAGTTCCTCGGCTTTGAGCAGATCCTGAAGAACTCCCTTACCACGCTCCCAATGGGCGGTGGTAAGGGCGGCTCCGACTTCGATCCTAAGGGCAAGTCGGACAAYGAGGTCATGCGCTTTTGCCAGTCCTTTATGACTGAGCTCCAGARGCACGTCGGGGCTGACACCGACGTTCCTGCTGGCGATATTGGCGTCGGCGGTCGCGAGATYGGTTATCTGTTTGGACAGTAYAAGCGCCTCAGGAACGAGTTCACGGGCGTCCTCACGGGCAAGAACATCAAGTGGGGCGGGTCTCTCATCAGRCCAGAGGCCACAGGGTATGGAGCTGTCTACTTCCTGGAGGAGATGT

>Sweh144

GCTCTCGGGCCCTACAAGGGTGGTCTCCGCTTCCACCCCTCTGTCAACCTCTCGATCCTTAAGTTCCTCGGCTTTGAGCAGATCCTGAAGAACTCCCTTACCACGCTCCCGATGGGCGGTGGTAAGGGCGGCTCCGACTTCGATCCTAAGGGCAAGTCGGACAACGAGGTCATGCGCTTCTGCCAGTCCTTTATGACCGAGCTCCAGAGGCACGTCGGGGCTGACACCGACGTTCCTGCTGGCGATATTGGCGTCGGCGGTCGCGAGATCGGTTATCTGTTTGGACAGTATAAGCGCCTCAGGAACGAGTTCACGGGCGTTCTCACGGGCAAGAACATCAAGTGGGGTGGGTCTCTCATCAGACCAGAGGCCACAGGGTATGGAGCTGTCTACTTCCTGGAGGAGATGT

>Sweh148

GCTCTCGGGCCCTACAAGGGTGGTCTCCGCTTCCACCCCTCTGTCAAYCTCTCGATCCTYAAGTTCCTCGGCTTTGAGCAGATCCTGAAGAACTCCCTTACCACGCTYCCGATGGGCGGTGGTAAGGGCGGCTCCGACTTCGATCCTAAGGGCAAGTCGGACAACGAGGTCATGCGCTTYTGCCAGTCCTTTATGACYGAGCTCCAGAGGCACGTCGGGGCTGACACCGACGTTCCTGCTGGCGATATTGGCGTCGGCGGTCGCGAGATYGGTTATCTGTTTGGACAGTACAAGCGCCTCAGGAACGAGTTCACRGGCGTCCTCACGGGCAAGAACATCAAGTGGGGCGGRTCTCTCATCAGRCCAGAGGCCACAGGGTATGGAGCYGTCTACTTCCTGGAGGAGATGT

>Sweh149

GCTCTCGGGCCCTACAAGGGTGGTCTCCGCTTCCACCCCTCTGTCAAYCTCTCGATCCTYAAGTTCCTCGGCTTTGAGCAGATCCTGAAGAACTCCCTTACCACGCTTCCGATGGGCGGTGGTAAGGGCGGCTCCGACTTCGATCCTAAGGGCAAGTCGGACAACGAGGTCATGCGCTTYTGYCAGTCCTTTATGACYGAGCTCCAGAGGCACGTCGGGGCTGACACCGACGTTCCTGCTGGCGATATTGGCGTCGGCGGTCGCGAGATYGGTTATCTGTTTGGACAGTAYAAGCGYCTCAGGAACGAGTTCACGGGCGTYCTCACGGGCAARAACATCAAGTGGGGCGGGTCTCTCATCAGRCCAGAGGCCACAGGGTATGGAGCTGTCTACTTCCTGGAGGAGATGT

>Sweh151

GCTCTCGGGCCCTACAAGGGTGGTCTCCGCTTCCACCCCTCTGTCAAYCTCTCRATCCTCAAGTTCCTCGGCTTTGAGCAGATCCTGAAGAACTCCCTTACCACGCTYCCRATGGGCGGTGGTAAGGGCGGCTCCGACTTCGATCCTAAGGGCAAGTCGGACAACGAGGTCATGCGCTTYTGCCAGTCCTTTATGACYGAGCTCCAGAGGCACGTCGGGGCTGACACCGACGTTCCTGCTGGCGATATTGGCGTCGGCGGTCGCGAGATYGGTTATCTGTTTGGRCAGTAYAAGCGCCTCAGGAACGAGTTCACGGGCGTCCTCACGGGCAAGAACATCAAGTGGGGCGGGTCTCTCATCAGGCCAGAGGCCACAGGGTATGGAGCTGTCTACTTCCTGGAGGAGATGT

>Sweh154

GCTCTCGGGCCCTACAAGGGTGGTCTCCGCTTCCACCCCTCTGTCAACCTCTCGATCCTTAAGTTCCTCGGCTTTGAGCAGATCCTGAAGAACTCCCTTACCACGCTCCCGATGGGCGGTGGTAAGGGCGGCTCCGACTTCGATCCTAAGGGCAAGTCGGACAACGAGGTCATGCGCTTCTGCCAGTCCTTTATGACCGAGCTCCAGAGGCACGTCGGGGCTGACACCGACGTTCCTGCTGGCGATATTGGCGTCGGCGGTCGCGAGATCGGTTATCTGTTTGGACAGTATAAGCGCCTCAGGAACGAGTTCACGGGCGTTCTCACGGGCAAGAACATCAAGTGGGGTGGGTCTCTCATCAGACCAGAGGCCACAGGGTATGGAGCTGTCTACTTCCTGGAGGAGATGT

>Sweh156

GCTCTCGGGCCCTACAAGGGTGGTCTCCGCTTCCACCCCTCTGTCAACCTCTCGATCCTTAAGTTCCTCGGCTTTGAGCAGATCCTGAAGAACTCCCTTACCACGCTTCCGATGGGCGGTGGTAAGGGCGGCTCCGACTTCGATCCTAAGGGCAAGTCGGACAACGAGGTCATGCGCTTCTGCCAGTCCTTTATGACCGAGCTCCAGAGGCACGTCGGGGCTGACACCGACGTTCCTGCTGGCGATATTGGCGTCGGCGGTCGCGAGATCGGTTATCTGTTTGGACAGTATAAGCGCCTCAGGAACGAGTTTACGGGCGTCCTCACGGGCAAGAACATCAAGTGGGGCGGGTCTCTCATCAGACCAGAGGCCACAGGGTATGGAGCTGTCTACTTCCTGGAGGAGATGT

>Sweh158

GCTCTCGGGCCCTACAAGGGTGGTCTCCGCTTCCACCCCTCTGTCAACCTCTCGATCCTTAAGTTCCTCGGCTTTGAGCAGATCCTGAAGAACTCCCTTACCACGCTTCCGATGGGCGGTGGTAAGGGCGGCTCCGACTTCGATCCTAAGGGCAAGTCGGACAACGAGGTCATGCGCTTCTGCCAGTCCTTTATGACCGAGCTCCAGAGGCACGTCGGGGCTGACACCGACGTTCCTGCTGGCGATATTGGCGTCGGCGGTCGCGAGATCGGTTATCTGTTTGGACAGTATAAGCGCCTCAGGAACGAGTTTACGGGCGTCCTCACGGGCAAGAACATCAAGTGGGGCGGGTCTCTCATCAGACCAGAGGCCACAGGGTATGGAGCTGTCTACTTCCTGGAGGAGATGT

>Sweh159

GCTCTCGGGCCCTACAAGGGTGGTCTCCGCTTCCACCCCTCTGTCAACCTCTCGATCCTTAAGTTCCTCGGCTTTGAGCAGATCCTGAAGAACTCCCTTACCACGCTTCCGATGGGCGGTGGTAAGGGCGGCTCCGACTTCGATCCTAAGGGCAAGTCGGACAACGAGGTCATGCGCTTCTGCCAGTCCTTTATGACCGAGCTCCAGAGGCACGTCGGGGCTGACACCGACGTTCCTGCTGGCGATATTGGCGTCGGCGGTCGCGAGATCGGTTATCTGTTTGGACAGTATAAGCGCCTCAGGAACGAGTTTACGGGCGTCCTCACGGGCAAGAACATCAAGTGGGGCGGGTCTCTCATCAGACCAGAGGCCACAGGGTATGGAGCTGTCTACTTCCTGGAGGAGATG-

>Sweh160

GCTCTCGGGCCCTACAAGGGTGGTCTCCGCTTCCACCCCTCTGTCAACCTCTCGATCCTTAAGTTCCTCGGCTTTGAGCAGATCCTGAAGAACTCCCTTACCACGCTTCCGATGGGCGGTGGTAAGGGCGGCTCCGACTTCGATCCTAAGGGCAAGTCGGACAACGAGGTCATGCGCTTCTGCCAGTCCTTTATGACCGAGCTCCAGAGGCACGTCGGGGCTGACACCGACGTTCCTGCTGGCGATATTGGCGTCGGCGGTCGCGAGATCGGTTATCTGTTTGGACAGTATAAGCGCCTCAGGAACGAGTTTACGGGCGTCCTCACGGGCAAGAACATCAAGTGGGGCGGGTCTCTCATCAGACCAGAGGCCACAGGGTATGGAGCTGTCTACTTCCTGGAGGAGATGT

>Sweh161

GCTCTCGGGCCCTACAAGGGTGGTCTCCGCTTCCACCCCTCTGTCAAYCTCTCGATCCTYAAGTTCCTCGGCTTTGAGCAGATCCTGAAGAACTCCCTTACCACGCTCCCRATGGGCGGTGGTAAGGGCGGCTCCGACTTCGATCCTAAGGGSAAGTCGGACAACGAGGTCATGCGCTTYTGCCAGTCCTTTATGACYGAGCTCCAGAGGCACGTCGGGGCTGACACCGACGTTCCTGCTGGCGATATTGGCGTCGGCGGTCGCGAGATYGGTTATCTGTTTGGACAGTAYAAGCGYCTCAGGAACGAGTTCACGGGCGTCCTCACGGGCAAGAACATCAAGTGGGGCGGGTCTCTCATCAGRCCAGAGGCCACAGGGTATGGAGCTGTCTACTTCCTGGAGGAGATGT

>Sweh163

-CTCTCGGGCCCTACAAGGGTGGTCTCCGCTTCCACCCCTCTGTCAACCTCTCGATCCTYAAGTTCCTCGGCTTTGAGCAGATCCTGAAGAACTCCCTTACCACGCTYCCGATGGGCGGTGGTAAGGGCGGCTCCGACTTCGATCCTAAGGGCAAGTCGGACAACGAGGTCATGCGCTTTTGCCAGTCCTTTATGACYGAGCTCCAGAGGCACGTCGGGGCTGACACCGACGTTCCTGCTGGCGATATTGGCGTCGGCGGTCGCGAGATYGGTTATCTGTTTGGACAGTAYAAGCGYCTCAGGAACGAGTTCACGGGCGTCCTCACGGGCAAGAACATCAAGTGGGGCGGGTCTCTCATCAGRCCAGAGGCCACAGGGTATGGAGCYGTCTACTTCCTGGAGGAGATGT

>Sweh167

GCTCTCGGGCCCTACAAGGGTGGTCTCCGCTTCCACCCCTCTGTCAACCTCTCGATCCTTAAGTTCCTCGGCTTTGAGCAGATCCTGAAGAACTCCCTTACCACGCTCCCGATGGGCGGTGGTAAGGGCGGCTCCGACTTCGATCCTAAGGGCAAGTCGGACAACGAGGTCATGCGCTTCTGCCAGTCCTTTATGACCGAGCTCCAGAGGCACGTCGGGGCTGACACCGACGTTCCTGCTGGCGATATTGGCGTCGGCGGTCGCGAGATCGGTTATCTGTTTGGACAGTATAAGCGCCTCAGGAACGAGTTCACGGGCGTTCTCACGGGCAAGAACATCAAGTGGGGTGGGTCTCTCATCAGACCAGAGGCCACAGGGTATGGAGCTGTCTACTTCCTGGAGGAGATGT

>Sweh168

GCTCTCGGGCCCTACAAGGGTGGTCTCCGCTTCCACCCCTCTGTCAACCTCTCGATCCTTAAGTTCCTCGGCTTTGAGCAGATCCTGAAGAACTCCCTTACCACGCTCCCGATGGGCGGTGGTAAGGGCGGCTCCGACTTCGATCCTAAGGGCAAGTCGGACAACGAGGTCATGCGCTTCTGCCAGTCCTTTATGACCGAGCTCCAGAGGCACGTCGGGGCTGACACCGACGTTCCTGCTGGCGATATTGGCGTCGGCGGTCGCGAGATCGGTTATCTGTTTGGACAGTATAAGCGCCTCAGGAACGAGTTCACGGGCGTTCTCACGGGCAAGAACATCAAGTGGGGTGGGTCTCTCATCAGACCAGAGGCCACAGGGTATGGAGCTGTCTACTTCCTGGAGGAGATGT

>Sweh169

GCTCTCGGGCCCTACAAGGGTGGTCTCCGCTTCCACCCCTCTGTCAAYCTCTCGATCCTYAAGTTCCTCGGCTTTGAGCAGATCCTGAAGAACTCCCTTACCACGCTYCCRATGGGCGGTGGTAAGGGCGGCTCCGACTTCGATCCTAAGGGCAAGTCGGACAACGAGGTCATGCGCTTYTGCCAGTCCTTTATGACYGAGCTCCAGAGGCACGTCGGGGCTGACACCGACGTTCCTGCTGGCGATATTGGCGTCGGCGGTCGCGAGATYGGTTATCTGTTTGGACAGTAYAAGCGCCTCAGGAACGAGTTYACGGGCGTCCTCACGGGCAAGAACATCAAGTGGGGCGGGTCTCTCATCAGACCAGAGGCCACAGGGTATGGAGCTGTCTACTTCCTGGAGGAGATGT

>Sweh170

GCTCTCGGGCCCTACAAGGGTGGTCTCCGCTTCCACCCCTCTGTCAACCTCTCGATCCTYAAGTTCCTCGGCTTTGAGCAGATCCTGAAGAACTCCCTTACCACGCTYCCRATGGGCGGTGGTAAGGGCGGCTCCGACTTCGATCCTAAGGGCAAGTCGGACAACGAGGTCATGCGCTTYTGCCAGTCCTTTATGACYGAGCTCCAGAGGCACGTCGGGGCTGACACCGACGTTCCTGCTGGCGATATTGGCGTCGGCGGTCGCGAGATYGGTTATCTGTTTGGACAGTAYAAGCGCCTCAGGAACGAGTTYACGGGCGTCCTCACGGGYAAGAACATYAAGTGGGGYGGGTCTCTCATCAGRCCAGAGGCCACAGGGTATGGAGCYGTCTACTTCCTGGAGGAGATGT

>Sweh171

-CTCTCGGGCCCTACAAGGGTGGTCTCCGCTTCCACCCCTCTGTCAACCTCTCGATCCTYAAGTTCCTCGGCTTTGAGCAGATCCTGAAGAACTCCCTTACCACGCTTCCGATGGGCGGTGGTAAGGGCGGCTCCGACTTCGATCCYAAGGGCAAGTCGGACAACGAGGTCATGCGCTTYTGCCAGTCCTTTATGACYGAGCTCCAGAGGCACGTCGGGGCTGACACCGACGTTCCTGCTGGCGATATTGGCGTCGGCGGTCGCGAGATCGGTTATCTGTTTGGACAGTAYAAGCGCCTCAGGAACGAGTTCACGGGCGTYCTCACGGGCAAGAACATCAAGTGGGGCGGGTCTCTCATCAGGCCAGAGGCCACAGGGTATGGAGCTGTCTACTTCCTGGAGGAGATGT

>Sweh179

GCTCTCGGGCCCTACAAGGGTGGTCTCCGCTTCCACCCCTCTGTCAACCTCTCGATCCTTAAGTTCCTCGGCTTTGAGCAGATCCTGAAGAACTCCCTTACCACGCTCCCGATGGGCGGTGGTAAGGGCGGCTCCGACTTCGATCCTAAGGGCAAGTCGGACAACGAGGTCATGCGCTTCTGCCAGTCCTTTATGACCGAGCTCCAGAGGCACGTCGGGGCTGACACCGACGTTCCTGCTGGCGATATTGGCGTCGGCGGTCGCGAGATCGGTTATCTGTTTGGACAGTATAAGCGCCTCAGGAACGAGTTTACGGGCGTCCTCACGGGCAAGAACATCAAGTGGGGCGGGTCTCTCATCAGACCAGAGGCCACAGGGTATGGAGCTGTCTACTTCCTGGAGGAGATGT

>Sweh184

GCTCTCGGGCCCTACAAGGGTGGTCTCCGCTTCCACCCCTCTGTCAAYCTCTCGATCCTYAAGTTCCTCGGCTTTGAGCAGATCCTGAAGAACTCCCTTACCACGCTCCCRATGGGCGGTGGTAAGGGCGGCTCCGACTTCGATCCTAAGGGCAAGTCGGACAACGAGGTCATGCGCTTYTGCCAGTCCTTTATGACYGAGCTCCAGAGGCACGTCGGGGCTGACACCGACGTTCCTGCTGGCGATATTGGCGTCGGCGGTCGCGAGATYGGTTATCTGTTTGGACAGTAYAAGCGYCTCAGGAACGAGTTCACGGGCGTCCTCACGGGCAAGAACATCAAGTGGGGCGGGTCTCTCATCAGRCCAGAGGCCACAGGGTATGGAGCTGTCTACTTCCTGGAGGAGATGT

>Sweh186

GCTCTCGGGCCCTACAAGGGTGGTCTCCGCTTCCACCCCTCTGTCAACCTCTCGATCCTTAAGTTCCTCGGCTTTGAGCARATCCTGAAGAACTCCCTTACCACGCTYCCGATGGGCGGTGGTAAGGGCGGCTCCGACTTCGATCCTAAGGGCAAGTCGGACAACGAGGTCATGCGCTTCTGCCAGTCCTTTATGACYGAGCTCCAGAGGCACGTCGGRGCTGACACCGACGTTCCTGCTGGCGATATTGGCGTCGGCGGTCGCGAGATYGGTTATCTGTTTGGACAGTAYAAGCGCCTCAGGAACGAGTTYACGGGCGTCCTCACGGGCAAGAACATCAAGTGGGGYGGGTCTCTYATCAGACCAGAGGCCACAGGRTATGGAGCYGTCTACTTCCTGGAGGAGATGT

>Sweh188

GCTCTCGGRCCCTACAAGGGTGGTCTCCGCTTCCACCCCTCTGTCAACCTCTCGATCCTTAAGTTCCTCGGCTTTGAGCARATCCTGAAGAACTCCCTTACCACGCTCCCGATGGGCGGTGGTAAGGGCGGCTCCGACTTCGATCCTAAGGGCAAGTCGGACAACGAGGTCATGCGCTTCTGCCAGTCCTTTATGACCGAGCTCCAGAGGCACGTCGGRGCTGACACCGACGTTCCTGCTGGCGATATTGGCGTCGGCGGTCGCGAGATYGGTTATYTGTTTGGACAGTATAAGCGCCTCAGGAACGAGTTCACGGGCGTCCTCACGGGCAARAACATCAAGTGGGGCGGGTCTCTCATCAGRCCAGAGGCCACAGGRTATGGAGCTGTCTACTTCCTGGAGGAGATGT

>Sweh189

GCTCTCGGGCCCTACAAGGGTGGTCTCCGCTTCCACCCCTCTGTCAACCTCTCGATCCTYAAGTTCCTCGGCTTTGAGCAGATCCTGAAGAACTCCCTTACCACGCTYCCRATGGGCGGTGGTAAGGGCGGCTCCGACTTCGATCCTAAGGGCAAGTCGGACAACGAGGTCATGCGCTTTTGCCAGTCCTTTATGACTGAGCTCCAGAGGCACGTCGGGGCTGACACCGACGTTCCYGCTGGCGATATTGGCGTCGGCGGTCGCGAGATYGGTTATCTGTTTGGACAGTACAAGCGYCTCAGGAACGAGTTCACGGGCGTCCTCACGGGCAAGAACATCAAGTGGGGYGGGTCTCTCATCAGRCCAGAGGCCACAGGGTATGGAGCTGTCTACTTCCTGGAGGAGATGT

>Sweh191

GCTCTCGGGCCCTACAAGGGTGGTCTCCGCTTCCACCCCTCTGTCAACCTCTCGATCCTTAAGTTCCTCGGCTTTGAGCAGATCCTGAAGAACTCCCTTACCACGCTYCCGATGGGCGGTGGTAAGGGCGGCTCCGACTTCGATCCTAAGGGCAAGTCGGACAACGAGGTCATGCGCTTCTGCCAGTCCTTTATGACCGAGCTCCAGAGGCACGTCGGGGCTGACACCGACGTTCCTGCTGGCGATATTGGCGTCGGCGGTCGCGAGATCGGTTATCTGTTTGGACAGTATAAGCGCCTCAGGAACGAGTTYACGGGCGTYCTCACGGGCAAGAACATCAAGTGGGGYGGGTCTCTCATCAGACCAGAGGCCACAGGGTATGGAGCTGTCTACTTCCTGGAGGAGATGT

>Sweh192

-CTCTCGGGCCCTACAAGGGTGGTCTCCGCTTCCACCCCTCTGTCAACCTCTCGATCCTTAAGTTCCTCGGCTTTGAGCAGATCCTGAAGAACTCCCTTACCACGCTCCCGATGGGCGGTGGTAAGGGCGGCTCCGACTTCGATCCTAAGGGCAAGTCGGACAACGAGGTCATGCGCTTCTGCCAGTCCTTTATGACCGAGCTCCAGAGGCACGTCGGGGCTGACACCGACGTTCCTGCTGGCGATATTGGCGTCGGCGGTCGCGAGATCGGTTATCTGTTTGGACAGTATAAGCGCCTCAGGAACGAGTTCACGGGCGTTCTCACGGGCAAGAACATCAAGTGGGGTGGGTCTCTCATCAGACCAGAGGCCACAGGGTATGGAGCTGTCTACTTCCTGGAGGAGATGT

>Sweh193

GCTCTCGGGCCCTACAAGGGTGGTCTCCGCTTCCACCCCTCTGTCAACCTCTCGATCCTYAAGTTCCTCGGCTTTGAGCAGATCCTGAAGAACTCCCTTACCACGCTYCCGATGGGCGGTGGTAAGGGCGGCTCCGACTTCGATCCTAAGGGCAAGTCGGACAACGAGGTCATGCGCTTYTGCCAGTCCTTTATGACYGAGCTCCAGAGGCACGTCGGGGCTGACACCGACGTTCCTGCTGGCGATATTGGCGTCGGCGGTCGCGAGATYGGTTATCTGTTTGGACAGTAYAAGCGCCTCAGGAACGAGTTCACGGGCGTCCTCACGGGCAAGAACATCAAGTGGGGCGGGTCTCTCATCAGRCCAGAGGCCACAGGGTATGGAGCTGTCTACTTCCTGGAGGAGAT--

>Sweh195

GCTCTCGGGCCCTACAAGGGTGGTCTCCGCTTCCACCCCTCTGTCAACCTCTCGATCCTCAAGTTCCTCGGCTTTGAGCAGATCCTGAAGAACTCCCTTACCACGCTYCCRATGGGCGGTGGTAAGGGCGGCTCCGACTTCGATCCTAAGGGCAAGTCGGACAACGAGGTCATGCGCTTYTGCCAGTCCTTTATGACTGAGCTCCAGAGGCACGTCGGGGCTGACACCGACGTTCCTGCTGGCGATATTGGCGTCGGCGGTCGCGAGATYGGTTATCTGTTTGGACAGTACAAGCGCCTCAGGAACGAGTTCACGGGCGTCCTCACGGGCAAGAACATCAAGTGGGGCGGGTCTCTCATCAGGCCAGAGGCCACAGGGTATGGAGCTGTCTACTTCCTGGAGGAGATGT

>Sweh196

GCTCTCGGGCCCTACAAGGGTGGTCTCCGYTTCCACCCCTCTGTCAACCTCTCGATCCTCAAGTTCCTCGGCTTTGAGCAGATCCTGAAGAACTCCCTTACCACGCTTCCRATGGGCGGTGGTAAGGGCGGCTCCGACTTCGATCCTAAGGGCAAGTCGGACAACGAGGTCATGCGCTTYTGCCAGTCCTTTATGACYGAGCTCCAGAGGCACGTCGGGGCTGACACCGACGTTCCTGCTGGCGATATTGGCGTCGGCGGTCGCGAGATCGGTTATCTGTTTGGRCAGTAYAAGCGCCTCAGGAACGAGTTCACGGGCGTCCTCACGGGCAAGAACATCAAGTGGGGCGGGTCTCTCATCAGRCCAGAGGCCACAGGGTATGGAGCTGTCTACTTCCTGGAGGAGATGT

>Sweh197

GCTCTCGGGCCCTACAAGGGTGGTCTCCGCTTCCACCCCTCTGTCAACCTCTCGATCCTYAAGTTCCTCGGCTTTGAGCAGATCCTGAAGAACTCCCTTACCACGCTYCCRATGGGCGGTGGTAAGGGCGGCTCCGACTTCGATCCTAAGGGCAAGTCGGACAACGAGGTCATGCGCTTYTGCCAGTCCTTTATGACYGAGCTCCAGAGGCACGTCGGGGCTGACACCGACGTTCCTGCTGGCGATATTGGCGTCGGCGGTCGCGAGATCGGTTATCTGTTTGGACAGTAYAAGCGYCTCAGGAACGAGTTCACGGGCGTCCTCACGGGCAAGAACATCAAGTGGGGCGGGTCTCTCATCAGGCCAGAGGCCACAGGGTATGGAGCTGTCTACTTCCTGGAGGAGATGT

>Sweh198

GCTCTCGGGCCCTACAAGGGTGGTCTCCGCTTCCACCCCTCTGTCAACCTCTCGATCCTTAAGTTCCTCGGCTTTGAGCAGATCCTGAAGAACTCCCTTACCACGCTCCCAATGGGCGGTGGTAAGGGCGGCTCCGACTTCGATCCTAAGGGCAAGTCGGACAACGAGGTCATGCGCTTTTGCCAGTCCTTTATGACTGAGCTCCAGAGGCACGTCGGGGCTGACACCGACGTTCCYGCTGGCGATATTGGCGTCGGCGGTCGCGAGATYGGTTATCTGTTTGGACAGTAYAAGCGYCTCAGGAACGAGTTCACGGGCGTCCTCACGGGCAAGAACATCAAGTGGGGYGGGTCTCTCATCAGRCCAGAGGCCACAGGGTATGGAGCTGTCTACTTCCTGGAGGAGATGT

>Sweh199

GCTCTCGGGCCCTACAAGGGTGGTCTCCGCTTCCACCCCTCTGTCAACCTCTCGATCCTTAAGTTCCTCGGCTTTGAGCAGATCCTGAAGAACTCCCTTACCACGCTTCCGATGGGCGGTGGTAAGGGCGGCTCCGACTTCGATCCTAAGGGCAAGTCGGACAACGAGGTCATGCGCTTCTGCCAGTCCTTTATGACCGAGCTCCAGAGGCACGTCGGGGCTGACACCGACGTTCCTGCTGGCGATATTGGCGTCGGCGGTCGCGAGATCGGTTATCTGTTTGGACAGTATAAGCGCCTCAGGAACGAGTTTACGGGCGTCCTCACGGGCAAGAACATCAAGTGGGGCGGGTCTCTCATCAGACCAGAGGCCACAGGGTATGGAGCTGTCTACTTCCTGGAGGAGATGT

>Sweh200

GCTCTCGGGCCCTACAAGGGTGGTCTCCGCTTCCACCCCTCTGTCAACCTCTCGATCCTTAAGTTCCTCGGCTTTGAGCAGATCCTGAAGAACTCCCTTACCACGCTTCCGATGGGCGGTGGTAAGGGCGGCTCCGACTTCGATCCTAAGGGCAAGTCGGACAACGAGGTCATGCGCTTCTGCCAGTCCTTTATGACCGAGCTCCAGAGGCACGTCGGGGCTGACACCGACGTTCCTGCTGGCGATATTGGCGTCGGCGGTCGCGAGATCGGTTATCTGTTTGGACAGTATAAGCGCCTCAGGAACGAGTTTACGGGCGTCCTCACGGGCAAGAACATCAAGTGGGGCGGGTCTCTCATCAGACCAGAGGCCACAGGGTATGGAGCTGTCTACTTCCTGGAGGAGATGT

>Sweh202

GCTCTCGGGCCCTACAAGGGTGGTCTCCGCTTCCACCCCTCTGTCAACCTCTCGATCCTTAAGTTCCTCGGCTTTGAGCAGATCCTGAAGAACTCCCTTACCACGCTTCCGATGGGCGGTGGTAAGGGCGGCTCCGACTTCGATCCTAAGGGCAAGTCGGACAACGAGGTCATGCGCTTCTGCCAGTCCTTTATGACCGAGCTCCAGAGGCACGTCGGGGCTGACACCGACGTTCCTGCTGGCGATATTGGCGTCGGCGGTCGCGAGATCGGTTATCTGTTTGGACAGTATAAGCGCCTCAGGAACGAGTTTACGGGCGTCCTCACGGGCAAGAACATCAAGTGGGGCGGGTCTCTCATCAGACCAGAGGCCACAGGGTATGGAGCTGTCTACTTCCTGGAGGAGATGT

>Sweh203

----TCGGGCCCTACAAGGGTGGTCTCCGCTTCCACCCCTCTGTCAACCTCTCGATCCTYAAGTTCCTCGGCTTTGAGCAGATCCTGAAGAACTCCCTTACCACGCTYCCGATGGGCGGTGGTAAGGGCGGCTCCGACTTYGATCCTAAGGGCAAGTCGGACAACGAGGTCATGCGCTTCTGCCAGTCCTTTATGACCGAGCTCCAGAGGCACGTCGGGGCTGACACCGACGTTCCTGCTGGCGATATTGGCGTCGGCGGTCGCGAGATCGGTTATCTGTTTGGACAGTATAAGCGCCTCAGGAACGAGTTCACGGGCGTYCTCACGGGCAAGAACATCAAGTGGGGCGGGTCTCTTATCAGACCAGAGGCCACAGGGTATGGAGCTGTCTACTTCCTGGAGGAGATGT

>Sweh206

GCTCTCGGGCCCTACAAGGGTGGTCTCCGCTTCCACCCCTCTGTCAAYCTCTCGATCCTYAAGTTCCTCGGCTTTGAGCAGATCCTGAAGAACTCCCTTACCACGCTCCCRATGGGCGGTGGTAAGGGCGGCTCCGACTTCGATCCTAAGGGCAAGTCGGACAACGAGGTCATGCGCTTYTGCCAGTCCTTTATGACYGAGCTCCAGAGGCACGTCGGGGCTGACACCGACGTTCCTGCTGGCGATATTGGCGTCGGCGGTCGCGAGATYGGTTATCTGTTTGGACAGTAYAAGCGYCTCAGGAACGAGTTCACGGGCGTCCTCACGGGCAAGAACATCAAGTGGGGCGGGTCTCTCATCAGRCCAGAGGCCACAGGGTATGGAGCTGTCTACTTCCTGGAGGAGATGT

>Sweh208

GCTCTCGGGCCCTACAAGGGTGGTCTCCGCTTCCACCCCTCTGTCAACCTCTCGATCCTTAAGTTCCTCGGCTTTGAGCAGATCCTGAAGAACTCCCTTACCACGCTCCCAATGGGCGGTGGTAAGGGCGGCTCCGACTTCGATCCTAAGGGCAAGTCGGACAACGAGGTCATGCGCTTCTGCCAGTCCTTTATGACCGAGCTCCAGAGGCACGTCGGGGCTGACACCGACGTTCCTGCTGGCGATATTGGCGTCGGCGGTCGCGAGATCGGTTATCTGTTTGGACAGTATAAGCGCCTCAGGAACGAGTTTACGGGCGTCCTCACGGGCAAGAACATCAAGTGGGGCGGGTCTCTCATCAGACCAGAGGCCACAGGGTATGGAGCTGTCTACTTCCTGGAGGAGATGT

>Sweh212

GCTCTCGGGCCCTACAAGGGTGGTCTCCGCTTCCACCCCTCTGTCAACCTCTCGATCCTTAAGTTCCTCGGCTTTGAGCAGATCCTGAAGAACTCCCTTACCACGCTCCCGATGGGCGGTGGTAAGGGCGGCTCCGACTTCGATCCTAAGGGCAAGTCGGACAACGAGGTCATGCGCTTCTGCCAGTCCTTTATGACCGAGCTCCAGAGGCACGTCGGGGCTGACACCGACGTTCCTGCTGGCGATATTGGCGTCGGCGGTCGCGAGATCGGTTATCTGTTTGGACAGTATAAGCGCCTCAGGAACGAGTTCACGGGCGTTCTCACGGGCAAGAACATCAAGTGGGGTGGGTCTCTCATCAGACCAGAGGCCACAGGGTATGGAGCTGTCTACTTCCTGGAGGAGATGT

>Sweh213

GCTCTCGGGCCCTACAAGGGTGGTCTCCGCTTCCACCCCTCTGTCAACCTCTCGATCCTTAAGTTCCTCGGCTTTGAGCAGATCCTGAAGAACTCCCTTACCACGCTTCCGATGGGCGGTGGTAAGGGCGGCTCCGACTTCGATCCTAAGGGCAAGTCGGACAACGAGGTCATGCGCTTCTGCCAGTCCTTTATGACCGAGCTCCAGAGGCACGTCGGGGCTGACACCGACGTTCCTGCTGGCGATATTGGCGTCGGCGGTCGCGAGATCGGTTATCTGTTTGGACAGTATAAGCGCCTCAGGAACGAGTTTACGGGCGTCCTCACGGGCAAGAACATCAAGTGGGGCGGGTCTCTCATCAGACCAGAGGCCACAGGGTATGGAGCTGTCTACTTCCTGGAGGAGATGT

>Sweh217

GCTCTCGGGCCCTACAAGGGTGGTCTCCGCTTCCACCCCTCTGTCAACCTCTCGATCCTTAAGTTCCTCGGCTTTGAGCAGATCCTGAAGAACTCCCTTACCACGCTTCCGATGGGCGGTGGTAAGGGCGGCTCCGACTTCGATCCTAAGGGCAAGTCGGACAACGAGGTCATGCGCTTCTGCCAGTCCTTTATGACCGAGCTCCAGAGGCACGTCGGGGCTGACACCGACGTTCCTGCTGGCGATATTGGCGTCGGCGGTCGCGAGATCGGTTATCTGTTTGGACAGTATAAGCGCCTCAGGAACGAGTTTACGGGCGTCCTCACGGGCAAGAACATCAAGTGGGGCGGGTCTCTCATCAGACCAGAGGCCACAGGGTATGGAGCTGTCTACTTCCTGGAGGAGATGT
