# Supplementary material for: Multilocus Genotyping of Human Giardia Isolates Suggests Limited Zoonotic Transmission and Association between Assemblage B and Flatulence in Children
Source: PLoS Negl Trop Dis. 2011 Aug 2;5(8):e1262. doi: 10.1371/journal.pntd.0001262 (PMC3149019; doi:10.1371/journal.pntd.0001262)
Supplement: File S3 — Tpi sequences from 120 isolates. (DOC) [file pntd.0001262.s004.doc]

Supplementary file S3. Tpi sequences from 120 isolates

>Sweh001

~~GGTAACTTCAAATGCAATGGATCGCTCGACTTCATTAAGAGCCACGTAGCGTCCATCGCCTCCTATAAGATCCCCGAGTCCGTGGACGTTGTTGTTGCTCCCTCCTTTGTGCACCTTTCTACAGCTATTGCGGCGAATACTTCGAAGTGTCTGAAAATAGCAGCACAGAACGTGTATCTGGAAGGGAACGGTGCATGGACCGGCGAGACAAGCGTCGAGATGCTGCTGGACATGGGGCTGAGCCATGTAATAATAGGACACTCTGAAAGACGTAGAATCATGGGCGAGACCAATGAGCAGAGTGCTAAGAAGGCGAAGCGTGCTCTGGACAAAGGTATGACTGTTATCTTCTGCACCGGAGAGACCCTGGATGAACGCAAGGCCAATAACACTATGGAGGTAAATATTGCTCAGCTCGAGGCTCTTAAGAAGGAGATTGGAGAATCAAAGAAGTTATGGGAGAACGTTGTAATTGCCTATGAGCCGGTGTGGTCTATCGGCACGGGC

>Sweh005

-GGGTAACTTCAARTGCAAYGGATCGCTCGACTTCATTAAGAGCCACGTAGCGTCCATCGCCTCCTATAAGATCCCCGAGTCCGTGGACGTTGTTGTTGCTCCCTCCTTTGTGCACCTTTCTACAGCTATTGCGGCGAAYACTTCGAAGTGTCTGAAAATAGCAGCACAGAACGTGTATCTGGARGGGAACGGTGCATGGACCGGCGAGACAAGCGTCGAGATGCTGCTGGACATGGGGCTGAGCYATGTAATARTAGGACACTCTGAAAGRCGTAGAATCATGGGCGAGACCAATGAGCAGAGTGCTAAGAAGGCGAAGCGTGCTCTGGACAAAGGTATGACTGTTATCTTCTGCACCGGAGAGACCYTGGATGARCGCAAGGCCAATAACACTATGGAGGTGAATATTGCTCAGCTCGAGGCTCTTAAGAAGGAGATTGGAGAATCAAAGAAGTTATGGGAGAACGTTGTAATTGCCTATGARCCGGTGTGGTCTATCGGCACGGG-

>Sweh006

-GGGTAACTTCAARTGCAATGGATCGCTCGACTTCATTAAGAGCCACGTAGCGTCCATCGCCTCCYATAAGATCCCCGAGTCCGTGGACGTTGTTGTTGCTCCCTCCTTTGTGCACCTTTCTACAGCTATTGCGGCGAAYACYTCGAAGTGTCTGAAAATAGCAGCACARAACGTGTATCTGGAGGGGAACGGTGCATGGACCGGCGAGACAAGCGTCGAGATGCTGCTRGACATGGGGCTGAGCCATGTAATAATAGGACACTCTGAAAGACGTAGAATCATGGGCGAGACCAATGAGCAGAGTGCTAAGAAGGCGAAGCGTGCTCTRGAYAAAGGTATGACTGTTATCTTCTGCACCGGAGAGACCCTGGATGAACGCAAGGCCAATAACACTATGGAGGTGAATATTGCTCAGCTCGAGGCTCTTAAGAAGGAGATTGGAGAATCAAAGAAGTTATGGGAGAACGTTGTAATTGCCTATGAGCCGGTGTGGTCTATCGGCACGGG-

>Sweh007

GgGGTAACTTCAAATGCAATGGATCGCTCGACTTCATTARGAGCCACGTAGCGTCCATCGCCTCCCATAAGATCCCCGAGTCCGTGGACGTTGTTGTTGCTCCCTCCTTTGTGCACCTTTCTACAGCTATTGCGGCGAACACCTCGAAGTGTCTGAAAATAGCAGCACAGAACGTGTATCTGGAGGGGAACGGTGCATGGACCGGCGAGACAAGCGTCGAGATGCTGCTRGACATGGGRCTGAGCCATGTAATAATAGGACACTCTGAAAGACGTAGAATCATGGGCGAGACCAAYGAGCAGAGTGCTAAGAAGGCGAAGCGTGCTCTGGACAAAGGTATGACTGTTATCTTCTGCACCGGAGAGACCCTGGATGAACGCAAGGCCAATAACACTATGGAGGTGAATATTGCTCAGCTCGAGGCTCTTAAGAAGGAGATTGGAGAATCAAAGAAGTTATGGGAGAACGTTGTAATTGCCTATGAGCCGGTGTGGTCTATCGGCACGGG

>Sweh008

-GGGTAACTTCAAGTGCAATGGATCGCTCGACTTCATTAAGAGCCACGTAGCGTCCATCGCCTCCYATAAGATCCCCGAGTCCGTGGACGTTGTTGTTGCTCCCTCCTTTGTGCACCTTTCTACAGCTATTGCGGCGAATACCTCGAAGTGTCTGAAAATAGCAGCACAGAAYGTGTATCTGGAGGGGAACGGTGCATGGACCGGCGAGACAAGCGTCGARATGCTGCTGGAYATGGGGCTGAGCCATGTAATAATAGGACACTCTGAAAGACGTAGAATCATGGGCGAGACCAATGAGCAGAGTGCTAAGAAGGCGAAGCGTGCTCTGGACAAAGGTATGACTGTTATCTTCTGCACCGGAGAGACCCTGGATGAACGCAAGGCCAATAACACTATGGAGGTGAATATTGCTCAGCTCGAGGCTCTTAAGAAGGAGATTGGAGAATCAAAGAAGTTATGGGAGAACGTTGTAATTGCCTATGAGCCGGTGTGGTCTATCGGCACGG--

>Sweh009

GGGGTAACTTCAAGTGCAACGGATCGCTCGACTTCATTAAGAGCCACGTAGCGTCCATCGCCTCCCATAAGATCCCCGAGTCCGTGGACGTTGTTGTTGCTCCCTCCTTTGTGCACCTTTCTACAGCTATTGCGGCRAACACCTCGAAGTGTCTGAAAATAGCAGCACAGAACGTGTATCTGGAGGGGAACGGTGCATGGACCGGCGAGACAAGCGTCGAGATGCTGCTGGACATGGGGCTGAGCCATGTAATAATAGGACACTCTGAAAGACGTAGAATCATGGGCGAGACCAATGAGCAGAGTGCTAAGAAGGCGAAGCGTGCTCTGGACAAAGGTATGACTGTTATCTTCTGCACCGGAGAGACCCTGGATGAACGCAAGGCCAATAACACTATGGAGGTGAATATTGCTCAGCTCGAGGCTCTTAAGAAGGAGATTGGAGAATCAAAGAAGTTATGGGAGAACGTTGTAATTGCCTATGAGCCGGTGTGGTCTATCGGCACGGGC

>Sweh010

~~GGTAACTTCAARTGCAATGGATCGCTCGACTTCATTAAGAGCCACGTAGCGTCCATCGCCTCCCATAAGATCCCCGAGTCCGTGGACGTTGTTGTTGCTCCCTCCTTTGTGCACCTTTCTACAGCTATTGCGGCGAACACCTCGAAGTGTCTGAAAATAGCAGCACAGAACGTGTATCTGGAGGGGAACGGTGCATGGACCGGCGAGACAAGCGTCGAGATGCTGCTGGACATGGGGCTGAGCCATGTAATAATAGGACACTCTGAAAGACGTAGAATCATGGGCGAGACCAATGAGCAGAGTGCTAAGAAGGCGAAGCGTGCTCTGGACAAAGGTATGACTGTTATCTTCTGCACCGGAGAGACCCTGGATGAACGCAAGGCCAATAACACTATGGAGGTGAATATTGCTCAGCTCGAGGCTCTTAAGAAGGAGATTGGAGAATCAAAGAAGyTATGGGAGAAYGTTGTAATTGCCTATGAGCCGGTGTGGTCTATCGGCACGGGC

>Sweh011

~GGGTAACTTCAAATGCAATGGATCGCTCGACTTCATTaAGAGCCACGTAGCGTCCATCGCCTCCYATAAGATCCCCGAGTCCGTGGACGTTGTTGTTGCTCCCTCCTTTGTGCAYCTTTCTACAGCTATTGCGGCRAACACCTCGAAGTGTCTGAAAATAGCAGCACAGAACGTGTATCTGGAGGGGAACGGTGCATGGACCGGCGAGACAAGCGTCGAGATGCTGCTGGACATGGGGCTGAGCCATGTAATAATAGGACACTCTGAAAGACGTAGAATCATGGGCGAGACCAATGAGCAGAGTGCTAAGAAGGCGAAGCGTGCTCTGGACAAAGGTATGACTGTTATCTTCTGCACCGGAGAGACCCTGGATGAACGCAAGGCCAATAACACTATGGAGGTGAATATTGCTCAGCTCGAGGCTCTTAAGAAGGAGATTGGAGAATCAAAGAAGTTATGGGAGAACGTTGTAATTGCCTATGAGCCGGTGTGGTCTATCGGCACGGGC

>Sweh013

GGGGTAACTTCAAGTGCAATGGATCGCTCGACTTCATTAAGAGCCACGTAGCRTCCATCGCCTCCCATAAGATCCCCGAGTCCGTGGACGTTGTTGTTGCTCCCTCCTTTGTGCACCTTTCTACAGCTATTGCGGCGAACACCTCGAAGTGTCTGAAAATAGCAGCACAGAACGTGTATCTGGAGGGGAACGGTGCATGGACCGGRGAGACAAGCGTCGAGATGCTGCTGGACATGGRGCTGAGCCATGTAATAATAGGACACTCTGAAAGACGTAGAATCATGGGCGAGACCAATGAGCAGAGTGCTAAGAAGGCGAAGCGTGCTCTGGACAAAGGTATGACTGTTATCTTCTGCACCGGAGAGACCCTGGATGAACGCAAGGCCAATAACACTATGGAGGTGAATATTGCTCAGCTCGAGGCTCTTAAGAAGGAGATTGGAGAATCAAAGAAGTTATGGGAGAACGTTGTAATTGCCTATGAGCCGGTGTGGTCTATC---------

>Sweh014

-GGGTAACTTCAAGTGCAATGGATCGCTCGACTTCATTAAGAGCCACGTAGCGTCCATCGCCTCCYATAAGATCCCCGAGTCCGTGGACGTTGTTGTTGCTCCCTCCTTTGTGCACCTTTCTACAGCTATTGCGGCRAAYACYTCGAAGTGTCTGAAAATAGCAGCACAGAACGTGTATCTGGAGGGGAACGGTGCATGGACCGGCGAGACAAGCGTCGAGATGCTGCTGGACATGGGGCTGAGCCATGTAATAATAGGACACTCTGAAAGACGTAGAATCATGGGCGAGACCAATGAGCAGAGTGCTAAGAAGGCGAAGCGTGCTCTGGACAAAGGTATGACTGTTATCTTCTGCACCGGAGAGACCCTGGATGAACGCAAGGCCAATAACACTATGGAGGTGAATATTGCTCAGCTCGAGGCTCTTAAGAAGGAGATTGGAGAATCAAAGAAGTTATGGGAGAACGTTGTAATTGCCTATGAGCCGGTGTGGTCTATCGGCACGGG-

>Sweh015

-GGGTAACTTCAAATGCAATGGATCGCTCGAYTTCATTAAGAGCCACGTAGCGTCCATCGCCTCCYATAAGATCCCCGAGTCCGTGGACGTTGTTGTTGCTCCCTCCTTTGTGCACCTTTCTACAGCTATTGCGGCGAAYACYTCGAAGTGTCTGAAAATAGCAGCACAGAACGTGTATCTGGAGGGGAACGGTGCATGGACCGGCGAGACAAGCGTCGAGATGCTGCTGGACATGGGGCTGAGCCATGTAATAATAGGACACTCTGAAAGACGTAGAATCATGGGCGAGACCAATGAGCAGAGTGCTAAGAAGGCGAAGCGTGCTCTGGACAAAGGTATGACTGTTATCTTCTGCACCGGAGAGACCCTGGATGAACGCAAGGCCAATAACACTATGGAGGTGAATATTGCTCAGCTCGAGGCTCTTAAGAAGGAGATTGGAGAATCAAAGAAGTTATGGGAGAACGTTGTAATTGCYTATGAGCCGGTGTGGTCTATCGGC------

>Sweh019

-GGGTAACTTCAAGTGCAATGGATCGCTCGACTTCATTAAGAGCCACGTAGCGTCCATCGCCTCCTATAAGATCCCCGAGTCCGTGGACGTTGTTGTTGCTCCCTCCTTTGTGCACCTTTCTACAGCTATTGCGGCGAACACCTCGAAGTGTCTGAAAATAGCAGCACAGAATGTGTATTTGGAGGGGAACGGTGCATGGACCGGCGAGACAAGCGTCGAGATGCTGCTGGACATGGGGCTGAGCCATGTAATAATAGGACACTCTGAAAGACGTAGAATCATGGGCGAGACCAATGAGCAGAGTGCTAAGAAGGCGAAGCGTGCTCTGGACAAAGGTATGACTGTTATCTTCTGCACCGGAGAGACCCTGGATGAACGCAAGGCCAATAACACTATGGAGGTGAATATTGCTCAGCTCGAGGCTCTTAAGAAGGAGATTGGAGAATCAAAGAAGTTATGGGAGAACGTTGTAATTGCCTATGAGCCGGTGTGGTCTATCGGCACGG--

>Sweh021

~GGGTAACTTCAAATGCAATGGATCGCTCGACTTCATTAAGAGCCACGTAGCGTCCATCGCCTCCTATAAGATCCCCGAGTCCGTGGACGTTGTTGTTGCTCCCTCCTTTGTGCACCTTTCTACAGCTATTGCGGCGAATACTTCGAAGTGTCTGAAAATAGCAGCACAGAACGTGTATCTGGAAGGGAACGGTGCATGGACCGGCGAGACAAGCGTCGAGATGCTGCTGGACATGGGGCTGAGCCATGTAATAATAGGACACTCTGAAAGACGTAGAATCATGGGCGAGACCAATGAGCAGAGTGCTAAGAAGGCGAAGCGTGCTCTGGACAAAGGTATGACTGTTATCTTCTGCACCGGAGAGACCCTGGATGAACGCAAGGCCAATAACACTATGGAGGTAAATATTGCTCAGCTCGAGGCTCTTAAGAAGGAGATTGGAGAATCAAAGAAGTTATGGGAGAACGTTGTAATTGCCTATGAGCCGGTGTGGTCTATCGGCACGGGC

>Sweh022

~GGGTAACTTCAAATGCAATGGATCGCTCGACTTCATTAAGAGCCACGTAGCGTCCATCGCCTCCTATAAGATCCCCGAGTCCGTGGACGTTGTTGTTGCTCCCTCCTTTGTGCACCTTTCTACAGCTATTGCGGCGAATACTTCGAAGTGTCTGAAAATAGCAGCACAGAACGTGTATCTGGAAGGGAACGGTGCATGGACCGGCGAGACAAGCGTCGAGATGCTGCTGGACATGGGGCTGAGCCATGTAATAATAGGACACTCTGAAAGACGTAGAATCATGGGCGAGACCAATGAGCAGAGTGCTAAGAAGGCGAAGCGTGCTCTGGACAAAGGTATGACTGTTATCTTCTGCACCGGAGAGACCCTGGATGAACGCAAGGCCAATAACACTATGGAGGTAAATATTGCTCAGCTCGAGGCTCTTAAGAAGGAGATTGGAGAATCAAAGAAGTTATGGGAGAACGTTGTAATTGCCTATGAGCCGGTGTGGTCTATCGGCACGGGC

>Sweh023

GGGGTAACTTCAAATGCAATGGATCGCTCGACTTCATTAAGAGCCACGTAGCGTCCATCGCCTCCTATAAGATCCCCGAGTCCGTGGACGTTGTTGTTGCTCCCTCCTTTGTGCACCTTTCTACAGCTATTGCGGCGAATACTTCGAAGTGTCTGAAAATAGCAGCACAGAACGTGTATCTGGAAGGGAACGGTGCATGGACCGGCGAGACAAGCGTCGAGATGCTGCTGGACATGGGGCTGAGCCATGTAATAATAGGACACTCTGAAAGACGTAGAATCATGGGCGAGACCAATGAGCAGAGTGCTAAGAAGGCGAAGCGTGCTCTGGACAAAGGTATGACTGTTATCTTCTGCACCGGAGAGACCCTGGATGAACGCAAGGCCAATAACACTATGGAGGTAAATATTGCTCAGCTCGAGGCTCTTAAGAAGGAGATTGGAGAATCAAAGAAGTTATGGGAGAACGTTGTAATTGCCTATGAGCCGGTGTGGTCTATCGGCACGGGC

>Sweh025

-GGGTAACTTCAARTGCAATGGATCGCTCGACTTCATTAAGAGCCACGTAGCGTCCATCGCCTCCYATAAGATCCCCGAGTCCGTGGACGTTGTTGTTGCTCCCTCCTTTGTGCACCTTTCTACAGCTATTGCGGCGAAYACYTCGAAGTGTCTGAAAATAGCAGCACAGAACGTGTATCTGGARGGGAACGGTGCATGGACCGGCGAGACAAGCGTCGAGATGCTGCTGGACATGGGGCTGAGCCATGTAATARTAGGACACTCTGAAAGACGTAGAATCATGGGCGAGACCAATGAGCAGAGTGCTAAGAAGGCGAAGCGTGCTCTGGACAAAGGTATGACTGTTATCTTCTGCACCGGAGAGACYCTGGATGARCGCAAGGCCAATAACACTATGGAGGTGAATATTGCTCAGCTCGAGGCTCTTAAGAAGGAGATTGGAGAMTCAAAGAAGTTRTGGGAGAACGTTGTRATTGCCTATGAGCCGGTGTGGTCTATCGGCACGG--

>Sweh027

-GGGTAACTTCAAGTGCAATGGATCGCTCGACTTCATTAAGAGCCACGTAGCGTCCATCGCCTCCCATAAGATCCCCGAGTCCGTGGACGTTGTTGTTGCTCCCTCCTTTGTGCACCTTTCTACAGCTATTGCGGCRAAYACCTCGAAGTGTCTGAAAATAGCAGCACAGAACGTGTATCTGGAGGGGAACGGTGCATGGACCGGCGAGACAAGCGTCGAGATGCTGCTGGACATGGGGCTGAGCCATGTAATAATAGGACACTCTGAAAGACGTAGAATCATGGGCGAGACCAATGAGCAGAGTGCTAAGAAGGCGAAGCGTGCTCTGGACAAAGGTATGACTGTTATCTTCTGCACCGGAGAGACCCTGGATGAACGCAAGGCCAATAACACTATGGAGGTGAATATTGCTCAGCTCGAGGCTCTTAAGAAGGAGATTGGAGAATCAAAGAAGTTATGGGAGRACGTTGTAATTGCCTATGAGCCGGTGTGGTCTATCGGCA-----

>Sweh028

-GGGTAACTTCAAGTGCAATGGATCGCTCGACTTCATTAAGAGCCACGTAGCGTCCATCGCCTCCTATAAGATCCCCGAGTCCGTGGACGTTGTTGTTGCTCCCTCCTTTGTGCATCTTTCTACAGCTATTGCGGTAAACACCTCGAAGTGTCTGAAAATAGCAGCACAGAACGTGTATCTGGAGGGGAACGGTGCATGGACCGGCGAGACAAGCGTCGAGATGCTGCTGGACATGGGGCTGAGCCATGTAATAATAGGACACTCTGAAAGACGTAGAATCATGGGCGAGACCAATGAGCAGAGTGCTAAGAAGGCGAAGCGTGCTCTGGACAAAGGTATGACTGTTATCTTCTGCACCGGAGAGACCCTGGATGAACGCAAGGCCAATAACACTATGGAGGTGAATATTGCTCAGCTCGAGGCTCTTAAGAAGGAGATTGGAGAATCAAAGAAGTTATGGGAGAACGTTGTAATTGCCTATGAGCCAGTGTGGTCTATCGGCACG---

>Sweh033

GGGGTAACTTCAAATGCAATGGATCGCTCGACTTCATTAAGAGCCACGTAGCGTCCATCGCCTCCTATAAGATCCCCGAGTCCGTGGACGTTGTTGTTGCTCCCTCCTTTGTGCACCTTTCTACAGCTATTGCGGCGAATACTTCGAAGTGTCTGAAAATAGCAGCACAGAACGTGTATCTGGAAGGGAATGGTGCATGGACCGGCGAGACAAGCGTCGAGATGCTGCTGGACATGGGGCTGAGCCATGTAATAATAGGACACTCTGAAAGACGTAGAATCATGGGCGAGACCAATGAGCAGAGTGCTAAGAAGGCGAAGCGTGCTCTGGACAAAGGTATGACTGTTATCTTCTGCACCGGAGAGACCCTGGATGAACGCAAGGCCAATAACACTATGGAGGTGAATATTGCTCAGCTCGAGGCTCTTAAGAAGGAGATTGGAGAATCAAAGAAGTTATGGGAGAACGTTGTAATTGCCTATGAGCCGGTGTGGTCTATCGGCACGGGC

>Sweh034

-GGGTAACTTCAAATGCAACGGATCGCTCGACTTCATTAAGAGCCACGTAGCGTCCATCGCCTCCTATAAGATCCCCGAGTCCGTGGACGTTGTTGTTGCTCCCTCCTTTGTGCACCTTTCTACAGCTATTGCGGCGAATACTTCGAAGTGTCTGAAAATAGCAGCACAGAACGTGTATCTGGAGGGGAACGGTGCATGGACCGGCGAGACAAGCGTCGAGATGCTGCTGGACATGGGGCTGAGCTATGTAATAGTAGGACACTCTGAAAGGCGTAGAATCATGGGCGAGACCAATGAGCAGAGTGCTAAGAAGGCGAAGCGTGCTCTGGACAAAGGTATGACTGTTATCTTCTGCACCGGAGAGACCCTGGATGAGCGCAAGGCCAATAACACTATGGAGGTGAATATTGCTCAGCTCGAGGCTCTTAAGAAGGAGATTGGAGAATCAAAGAAGTTATGGGAGAACGTTGTAATTGCCTATGAGCCGGTGTGGTCTATCGGCACGG--

>Sweh035

~GGGTAACTTCAARTGCAATGGATCGCTCGACTTCATTAAGAGCCACGTAGCGTCCATCGCCTCCCATAAGATCCCCGAGTCCGTGGACGTTGTTGTTGCTCCCTCCTTTGTGCAYCTTTCTACAGCTATTGCGGCGAACACCTCGAAGTGTCTGAAAATAGCAGCACAGAACGTGTATCTGGAGGGGAACGGTGCATGGACCGGCGAGAYAAGCGTCGAGATGCTGCTGGACATGGGGCTGAGCCATGTAATAATAGGACACTCTGAAAGACGTAGAATCATGGGCGAGACCAATGAGCAGAGTGCTAAGAAGGYGAAGCGTGCTCTGGACAAAGGTATGACTGTTATCTTCTGYACCGGAGAGACCCTGGATGAACGCAAGGCCAATAACACTATGGAGGTGAATATTGCTCAGCTCGAGGCTCTTAAGAAGGAGATTGGAGAATCAAAGAAGTTATGGGAGAACGTTGTAATTGCYTATGAGCCGGTGTGGTCTATCGGCACGGGC

>Sweh039

GGGGTAACTTCAAGTGCAATGGATCGCTCGACTTCATTAAGAGCCACGTAGCGTCCATCGCCTCCCATAAGATCCCCGAGTCCGTGGACGTTGTTGTTGCTCCCTCCTTTGTGCACCTTTCTACAGCTATTGCGGCGAACACCTCGAAGTGTCTGAAAATAGCAGCACAGAACGTGTATCTGGAGGGGAACGGTGCATGGACCGGCGAGACAAGCGTCGAGATGCTGCTGGACATGGGGCTGAGCCATGTAATAATAGGACACTCTGAAAGACGTAGAATCATGGGCGAGACCAATGAGCAGAGTGCTAAGAAGGCGAAGCGTGCTCTGGACAAAGGTATGACTGTTATCTTCTGCACCGGAGAGACCCTGGATGAACGCAAGGCCAATAACACTATGGAGGTGAATATTGCTCAGCTCGAGGCTCTTAAGAAGGAGATTGGAGAATCAAAGAAGTTATGGGAGAACGTTGTAATTGCCTATGAGCCGGTGTGGTCTATCGGCACGGGC

>Sweh041

GGGGTAACTTCAAATGCAATGGATCGCTCGACTTCATTAAGAGCCACGTAGCGTCCATCGCCTCCTATAAGATCCCCGAGTCCGTGGACGTTGTTGTTGCTCCCTCCTTTGTGCACCTTTCTACAGCTATTGCGGCGAATACTTCGAAGTGTCTGAAAATAGCAGCACAGAACGTGTATCTGGAAGGGAACGGTGCATGGACCGGCGAGACAAGCGTCGAGATGCTGCTGGACATGGGGCTGAGCCATGTAATAATAGGACACTCTGAAAGACGTAGAATCATGGGCGAGACCAATGAGCAGAGTGCTAAGAAGGCGAAGCGTGCTCTGGACAAAGGTATGACTGTTATCTTCTGCACCGGAGAGACCCTGGATGAACGCAAGGCCAATAACACTATGGAGGTAAATATTGCTCAGCTCGAGGCTCTTAAGAAGGAGATTGGAGAATCAAAGAAGTTATGGGAGAACGTTGTAATTGCCTATGAGCCGGTGTGGTCTATCGGCACGGGC

>Sweh042

~~~GTAACTTCAAATGCAATGGATCGCTCGACTTCATTAAGAGCCACGTAGCGTCCATCGCCTCCTATAAGATCCCCGAGTCCGTGGACGTTGTTGTTGCTCCCTCCTTTGTGCACCTTTCTACAGCTATTGCGGCGAATACTTCGAAGTGTCTGAAAATAGCAGCACAGAACGTGTATCTGGAAGGGAACGGTGCATGGACCGGCGAGACAAGCGTCGAGATGCTGCTGGACATGGGGCTGAGCCATGTAATAATAGGACACTCTGAAAGACGTAGAATCATGGGCGAGACCAATGAGCAGAGTGCTAAGAAGGCGAAGCGTGCTCTGGACAAAGGTATGACTGTTATCTTCTGCACCGGAGAGACCCTGGATGAACGCAAGGCCAATAACACTATGGAGGTAAATATTGCTCAGCTCGAGGCTCTTAAGAAGGAGATTGGAGAATCAAAGAAGTTATGGGAGAACGTTGTAATTGCCTATGAGCCGGTGTGGTCTATCGGCACGGGC

>Sweh043

~GGGTAACTTCAARTGCAATGGATCGCTCGACTTCATTAAGAGCCACGTAGCGTCCATCGCCTCCCATAAGATCCCCGAGTCCGTGGACGTTGTTGTTGCTCCCTCCTTTGTGCACCTTTCTACAGCTATTGCGGCGAAYACYTCGAAGTGTCTGAAAATAGCAGCACAGAACGTGTATCTGGAGGGGAACGGTGCATGGACCGGCGAGACAAGCGTCGAGATGCTGCTGGACATGGGGCTGAGYCATGTAATAATAGGACACTCTGAAAGACGTAGAATCATGGGCGAGACCAATGAGCAGAGTGCTAAGAAGGCGAAGCGTGCTCTGGACAAAGGTATGACTGTTATCTTCTGCACCGGAGAGACCCTGGATGAACGCAAGGCCAATAACACTATGGAGGTGAATATTGCTCAGCTCGAGGCTCTTAAGAAGGAGATTGGAGAATCAAAGAAGTTATGGGAGAACRTTGTAATTGCYTATGAGCCGGTGTGGTCTATCGGCACGGGC

>Sweh044

~GGGTAACTTCAARTGCAATGGATCGCTCGACTTCATTAAGAGCCACGTAGCGTCCATCGCCTCCYATAAGATCCCCGAGTCCGTGGACGTTGTTGTTGCTCCCTCCTTTGTGCACCTTTCTACAGCTATTGCGGCGAAYACYTCGAAGTGTCTGAAAATAGCAGCACAGAACGTGTATCTGGAGGGGAACGGTGCATGGACCGGCGAGACAAGCGTCGAGATGCTGCTGGACATGGGGCTGAGCCATGTAATAATAGGACACTCTGAAAGACGTAGAATCATGGGCGAGACCAATGAGCAGAGTGCTAAGAAGGCGAAGCGTGCTCTGGACAAAGGTATGACTGTTATCTTCTGCACCGGAGAGACCCTGGATGAACGCAAGGCCAATAACACTATGGAGGTRAATATTGCTCAGCTCGAGGCTCTTAAGAAGGAGATTGGAGAATCAAAGAAGTTATGGGAGAACGTTGTAATTGCCTATGAGCCGGTGTGGTCTATCGGCACGGGC

>Sweh045

GGGGTAACTTCAAATGCAATGGATCGCTCGACTTCATTAAGAGCCACGTAGCGTCCATCGCCTCCYATAAGATCCCCGAGTCCGTGGACGTTGTTGTTGCTCCCTCCTTTGTGCACCTTTCTACAGCTATTGCGGCGAAYACyTCGAAGTGTCTGAAAATAGCAGCACAGAACGTGTATCTGGARGGGAACGGTGCATGGACCGGCGAGACAAGCGTCGAGATGCTGCTGGACATGGGGCTGAGCCATGTAATAATAGGACACTCTGAAAGACGTAGAATCATGGGCGAGACCAATGAGCAGAGTGCTAAGAAGGCGAAGCGTGCTCTGGACAAAGGTATGACTGTTATCTTCTGCACCGGAGAGACCCTGGATGAACGCAAGGCCAATAACACTATGGAGGTGAATATTGCTCAGCTCGAGGYTCTTAAGAAGGAGATTGGAGAATCAAAGAAGTTATGGGAGAACGTTGTAATTGCCTATGAGCCGGTGTGGTCTATCGGCACGGG

>Sweh047

-GGGTAACTTCAAATGCAATGGATCGCTCGACTTCATTAAGAGCCACGTAGCGTCCATCGCCTCCTATAAGATCCCCGAGTCCGTGGACGTTGTTGTTGCTCCCTCCTTTGTGCACCTTTCTACAGCTATTGCGGCGAATACTTCGAAGTGTCTGAAAATAGCAGCACAGAACGTGTATCTGGAAGGGAACGGTGCATGGACCGGCGAGACAAGCGTCGAGATGCTGCTGGACATGGGGCTGAGCCATGTAATAATAGGACACTCTGAAAGACGTAGAATCATGGGCGAGACCAATGAGCAGAGTGCTAAGAAGGCGAAGCGTGCTCTGGACAAAGGTATGACTGTTATCTTCTGCACCGGAGAGACCCTGGATGAACGCAAGGCCAATAACACTATGGAGGTGAATATTGCTCAGCTCGAGGCTCTTAAGAAGGAGATTGGAGAATCAAAGAAGTTATGGGAGAACGTTGTAATTGCCTATGAGCCGGTGTGGTCTATCGGCACGGGC

>Sweh048

GGGGTAACTTCAAATGCAATGGATCGCTCGACTTCATTAAGAGCCACGTAGCGTCCATCGCCTCCTATAAGATCCCCGAGTCCGTGGACGTTGTTGTTGCTCCCTCCTTTGTGCACCTTTCTACAGCTATTGCGGCGAATACTTCGAAGTGTCTGAAAATAGCAGCACAGAACGTGTATCTGGAAGGGAACGGTGCATGGACCGGCGAGACAAGCGTCGAGATGCTGCTGGACATGGGGCTGAGCCATGTAATAATAGGACACTCTGAAAGACGTAGAATCATGGGCGAGACCAATGAGCAGAGTGCTAAGAAGGCGAAGCGTGCTCTGGACAAAGGTATGACTGTTATCTTCTGCACCGGAGAGACCCTGGATGAACGCAAGGCCAATAACACTATGGAGGTGAATATTGCTCAGCTCGAGGCTCTTAAGAAGGAGATTGGAGAATCAAAGAAGTTATGGGAGAACGTTGTAATTGCCTATGAGCCGGTGTGGTCTATCGGCACGGGC

>Sweh049

~~~~~AACTTCAAATGCAATGGATCGCTCGACTTCATTAAGAGCCACGTAGCGTCCATCGCCTCCTATAAGATCCCCGAGTCCGTGGACGTTGTTGTTGCTCCCTCCTTTGTGCACCTTTCTACAGCTATTGCGGCGAATACTTCGAAGTGTCTGAAAATAGCAGCACAGAACGTGTATCTGGAAGGGAACGGTGCATGGACCGGCGAGACAAGCGTCGAGATGCTGCTGGACATGGGGCTGAGCCATGTAATAATAGGACACTCTGAAAGACGTAGAATCATGGGCGAGACCAATGAGCAGAGTGCTAAGAAGGCGAAGCGTGCTCTGGACAAAGGTATGACTGTTATCTTCTGCACCGGAGAGACCCTGGATGAACGCAAGGCCAATAACACTATGGAGGTGAATATTGCTCAGCTCGAGGCTCTTAAGAAGGAGATTGGAGAATCAAAGAAGTTATGGGAGAACGTTGTAATTGCCTATGAGCCGGTGTGGTCTATCGGCACGGGC

>Sweh051

GGGGTAACTTCAAATGCAATGGATCGCTCGACTTCATTAAGAGCCACGTAGCGTCCATCGCCTCCTATAAGATCCCCGAGTCCGTGGACGTTGTTGTTGCTCCCTCCTTTGTGCACCTTTCTACAGCTATTGCGGCGAATACTTCGAAGTGTCTGAAAATAGCAGCACAGAACGTGTATCTGGAAGGGAACGGTGCATGGACCGGCGAGACAAGCGTCGAGATGCTGCTGGACATGGGGCTGAGCCATGTAATAATAGGACACTCTGAAAGACGTAGAATCATGGGCGAGACCAATGAGCAGAGTGCTAAGAAGGCGAAGCGTGCTCTGGACAAAGGTATGACTGTTATCTTCTGCACCGGAGAGACCCTGGATGAACGCAAGGCCAATAACACTATGGAGGTAAATATTGCTCAGCTCGAGGCTCTTAAGAAGGAGATTGGAGAATCAAAGAAGTTATGGGAGAACGTTGTAATTGCCTATGAGCCGGTGTGGTCTATCGGCACGGGC

>Sweh056

GGGGTAACTTCAAATGCAATGGATCGCTCGACTTCATtaAGAGCCACGTAGCGTCCATCGCCTCCTATAAGATCCCCGAGTCCGTGGACGTTGTTGTTGCTCCCTCCTTTGTGCACCTTTCTACAGCTATTGCGGCGAATACTTCGAAGTGTCTGAAAATAGCAGCACAGAACGTGTATCTGGAAGGGAACGGTGCATGGACCGGCGAGACAAGCGTCGAGATGCTGCTGGACATGGGGCTGAGCCATGTAATAATAGGACACTCTGAAAGACGTAGAATCATGGGCGAGACCAATGAGCAGAGTGCTAAGAAGGCGAAGCGTGCTCTGGACAAAGGTATGACTGTTATCTTCTGCACCGGAGAGACCCTGGATGAACGCAAGGCCAATAACACTATGGAGGTAAATATTGCTCAGCTCGAGGCTCTTAAGAAGGAGATTGGAGAATCAAAGAAGTTATGGGAGAACGTTGTAATTGCCTATGAGCCGGTGTGGTCTATCGGCACGGGC

>Sweh057

GGGGTAACTTCAAATGCAATGGATCGCTCGACTTCATTAAGAGCCACGTAGCGTCCATCGCCTCCTATAAGATCCCCGAGTCCGTGGACGTTGTTGTTGCTCCCTCCTTTGTGCACCTTTCTACAGCTATTGCGGCGAATACTTCGAAGTGTCTGAAAATAGCAGCACAGAACGTGTATCTGGAAGGGAACGGTGCATGGACCGGCGAGACAAGCGTCGAGATGCTGCTGGACATGGGGCTGAGCCATGTAATAATAGGACACTCTGAAAGACGTAGAATCATGGGCGAGACCAATGAGCAGAGTGCTAAGAAGGCGAAGCGTGCTCTGGACAAAGGTATGACTGTTATCTTCTGCACCGGAGAGACCCTGGATGAACGCAAGGCCAATAACACTATGGAGGTAAATATTGCTCAGCTCGAGGCTCTTAAGAAGGAGATTGGAGAATCAAAGAAGTTATGGGAGAACGTTGTAATTGCCTATGAGCCGGTGTGGTCTATCGGCACGGGC

>Sweh058

GGGGTAACTTCAAATGCAATGGATCGCTCGACTTCATTAAGAGCCACGTAGCGTCCATCGCCTCCTATAAGATCCCCGAGTCCGTGGACGTTGTTGTTGCTCCCTCCTTTGTGCACCTTTCTACAGCTATTGCGGCGAATACTTCGAAGTGTCTGAAAATAGCAGCACAGAACGTGTATCTGGAAGGGAACGGTGCATGGACCGGCGAGACAAGCGTCGAGATGCTGCTGGACATGGGGCTGAGCCATGTAATAATAGGACACTCTGAAAGACGTAGAATCATGGGCGAGACCAATGAGCAGAGTGCTAAGAAGGCGAAGCGTGCTCTGGACAAAGGTATGACTGTTATCTTCTGCACCGGAGAGACCCTGGATGAACGCAAGGCCAATAACACTATGGAGGTAAATATTGCTCAGCTCGAGGCTCTTAAGAAGGAGATTGGAGAATCAAAGAAGTTATGGGAGAACGTTGTAATTGCCTATGAGCCGGTGTGGTCTATCGGCACGGGC

>Sweh059

GGGGTAACTTCAAATGCAATGGATCGCTCGACTTCATTAAGAGCCACGTAGCGTCCATCGCCTCCTATAAGATCCCCGAGTCCGTGGACGTTGTTGTTGCTCCCTCCTTTGTGCACCTTTCTACAGCTATTGCGGCGAATACTTCGAAGTGTCTGAAAATAGCAGCACAGAACGTGTATCTGGAAGGGAACGGTGCATGGACCGGCGAGACAAGCGTCGAGATGCTGCTGGACATGGGGCTGAGCCATGTAATAATAGGACACTCTGAAAGACGTAGAATCATGGGCGAGACCAATGAGCAGAGTGCTAAGAAGGCGAAGCGTGCTCTGGACAAAGGTATGACTGTTATCTTCTGCACCGGAGAGACCCTGGATGAACGCAAGGCCAATAACACTATGGAGGTAAATATTGCTCAGCTCGAGGCTCTTAAGAAGGAGATTGGAGAATCAAAGAAGTTATGGGAGAACGTTGTAATTGCCTATGAGCCGGTGTGGTCTATCGGCACGGGC

>Sweh060

GGGGTAACTTCAAGTGCAATGGATCGCTCGACTTCATTAAGAGCCACGTAGCGTCCATCGCCTCCCATAAGATCCCCGAGTCCGTGGACGTTGTTGTTGCTCCCTCCTTTGTGCACCTTTCTACAGCTATTGCGGCGAACACCTCGAAGTGTCTGAAAATAGCAGCACAGAACGTGTATCTGGAGGGGAACGGTGCATGGACCGGCGAGACAAGCGTCGAGATGCTGCTGGACATGGGGCTGAGCCATGTAATAATAGGACACTCTGAAAGACGTAGAGTCATGGGCGAGACCAATGAGCAGAGTGCTAAGAAGGCGAAGCGTGCTCTGGACAAAGGTATGACTGTTATCTTCTGCACCGGAGAGACTCTGGATGAACGCAAGGCCAATAACACTATGGAGGTGAATATTGCTCAGCTCGAGGCTCTTAAGAAGGAGATTGGAGAATCAAAGAAGTTATGGGAGAACGTTGTAATTGCCTATGAGCCGGTGTGGTCTATCGGCACGGGC

>Sweh062

GGGGTAACTTCAAATGCAAYGGATCGCTCGACTTCATTAAGAGCCACGTAGCGTCCATCGCCTCCYATAAGATCCCCGAGTCCGTRGACGTTGTTGTTGCTCCCTCCTTTGTGCACCTTTCTACAGCTATTGCGGCGAAYACCTCGAAGTGTCTGAAAATAGCAGCACAGAACGTGTATCTGGAGGGGAACGGTGCATGGACCGGCGAGACAAGCGTCGAGATGCTGCTGGACATGGGGCTGAGCYATGTAATAATAGGACACTCTGAAAGACGTAGAATCATGGGCGAGACCAATGAGCAGAGTGCTAAGAAGGCGAAGCGTGCTCTGGACAAAGGTATGACTGTTATCTTCTGCACYGGAGAGACCCTGGATGAACGCAAGGCCAATAACACTATGGAGGTGAATATTGCTCAGCTCGAGGCTCTTAAGAAGGAGATTGGAGAATCAAAGAAGTTRTGGGAGAACGTTGTAATTGCCTATGAGCCGGTGTGGTCTATCGGCACGGGC

>Sweh064

-GGGTAACTTCAARTGCAATGGATCGCTCGACTTCATTAAGAGCCACGTAGCGTCCATCGCCTCCYATAAGATCCCCGAGTCCGTGGACGTTGTTGTTGCTCCCTCYTTTGTGCACCTTTCTACAGCTATTGCGGCGAAYACYTCGAAGTGTCTGAAAATAGCAGCACAGAACGTGTATCTGGAGGGGAACGGTGCATGGACCGGCGAGACAAGCGTCGAGATGCTGCTGGACATGGGGCTGAGCCATGTAATAATAGGACACTCTGAAAGACGTAGAATCATGGGCGAGACCAATGAGCAGAGTGCTAAGAAGGCGAAGCGTGCTCTGGACAAAGGTATGACTGTTATCTTCTGCACCGGAGAGACCCTGGATGAACGCAAGGCCAATAACACTATGGAGGTGAATATTGCTCAGCTCGAGRCTCTTAAGAAGGAGATTGGAGAATCAAAGAAGTTATGGGAGAACGTTGTAATTGCYTATGAGCCGGTGTGGTCTATCGGCACGG--

>Sweh066

~GGGTAACTTCAARTGCAAYGGATCGCTCGACTTCATTAAGAGCCACGTAGCGTCCATCGCCTCCyATAAGATCCCCGAGTCCGTGGACGTTGTTGTTGCTCCCTCCTTTGTGCACCTTTCTACAGCTATTGCGGCGAACACCTCGAAGTGTCTGAAAATAGCAGCACAGAACGTGTATCTGGAGGGGAACGGTGCATGGACCGGCGAGACAAGCGTCGAGATGCTGCTGGACATGGGGCTGAGCYATGTAATAATAGGACACTCTGAAAGACGTAGAATCATGGGCGAGACCAATGAGCAGAGTGCTAAGAAGGCGAAGCGTGCTCTGGACAAAGGTATGACTGTTATCTTCTGCACyGGAGAGACCCTGGATGAACGCAAGGCCAATAACACTATGGAGGTGAATATTGCTCAGCTCGAGGCTCTTAAGAAGGAGATTGGAGAMTCAAAGAAGTTrTGGGAGAACGTtgTAATTGCCTATGAgCCGGTGTGGTCTATCGGCACGGGC

>Sweh067

GGGGTAACTTCAAATGCAATGGATCGCTCGACTTCATTAAGAGCCACGTAGCGTCCATCGCCTCCTATAAGATCCCCGAGTCCGTGGACGTTGTTGTTGCTCCCTCCTTTGTGCACCTTTCTACAGCTATTGCGGCGAATACTTCGAAGTGTCTGAAAATAGCAGCACAGAACGTGTATCTGGAAGGGAACGGTGCATGGACCGGCGAGACAAGCGTCGAGATGCTGCTGGACATGGGGCTGAGCCATGTAATAATAGGACACTCTGAAAGACGTAGAATCATGGGCGAGACCAATGAGCAGAGTGCTAAGAAGGCGAAGCGTGCTCTGGACAAAGGTATGACTGTTATCTTCTGCACCGGAGAGACCCTGGATGAACGCAAGGCCAATAACACTATGGAGGTAAATATTGCTCAGCTCGAGGCTCTTAAGAAGGAGATTGGAGAATCAAAGAAGTTATGGGAGAACGTTGTAATTGCCTATGAGCCGGTGTGGTCTATCGGCACGGGC

>Sweh068

GGGGTAACTTCAARTGCAAYGGATCGCTCGACTTCATTAAGAGCCACGTAGCGTCCATCGCCTCCCATAAGATCCCCGAGTCCGTGGACGTTGTTGTTGCTCCCTCCTTTGTGCACCTTTCTACAGCTATTGCGGCGAAYACYTCGAAGTGTCTGAAAATAGYAGCACAGAACGTGTATCTGGAGGGGAACGGTGCATGGACCGGCGAGACAAGCGTCGAGATGCTGCTGGACATGGGGCTGAGCYATGTAATARTAGGACACTCTGAAAGRCGTAGAATCATGGGCGAGACCAATGAGCAGAGTGCTAAGAAGGCGAAGCGTGCTCTGGACAAAGGTATGACTGTTATCTTCTGCACCGGAGAGACCCTGGATGARCGCAAGGCCAATAACACTATGGAGGTGAATATTGCTCAGCTCGAGGCTCTTAAGAAGGAGATTGGAGAMTCAAAGAAGTTRTGGGAGAACGTTGTAATTGCYTATGARCCGGTGTGGTCTATCGGCACGGGC

>Sweh069

GGGGTAACTTCAARTGCAATGGATCGCTCGACTTCATTAAGAGCCACGTAGCGTCCATCGCCTCCCATAAGATCYCCGAGTCCGTGGACGTTGTTGTTGCTCCCTCCTTTGTGCACCTTTCTACAGCTATTGCGGCGAACACCTCGAAGTGTCTGAAAATAGCAGCACAGAACGTGTATCTGGAGGGGAACGGTGCATGGACCGGCGAGACAAGCGTCGAGATGCTGCTGGACATGGGGCTGAGCCATGTAATAATAGGACACTCTGAAAGACGTAGAATCATGGGCGAGACCAATGAGCAGAGTGCTAAGAAGGCGAAGCGTGCTCTGGACAAAGGTATGACTGTTATCTTCTGCACCGGAGAGACCCTGGATGAACGCAAGGCCAATAACACTATGGAGGTGAATATTGCTCAGCTCGAGGCTCTTAAGAAGGAGATTGGAGAATCAAAGAAGTTATGGGAGAACGTTGTAATTGCCTATGAGCCGGTGTGGTCTATCGGCAC

>Sweh074

GGGGTAACTTCAAATGCAATGGATCGCTCGACTTCATTAAGAGCCACGTAGCGTCCATCGCCTCCTATAAGATCCCCGAGTCCGTGGACGTTGTTGTTGCTCCCTCCTTTGTGCACCTTTCTACAGCTATTGCGGCGAATACTTCGAAGTGTCTGAAAATAGCAGCACAGAACGTGTATCTGGAAGGGAACGGTGCATGGACCGGCGAGACAAGCGTCGAGATGCTGCTGGACATGGGGCTGAGCCATGTAATAATAGGACACTCTGAAAGACGTAGAATCATGGGCGAGACCAATGAGCAGAGTGCTAAGAAGGCGAAGCGTGCTCTGGACAAAGGTATGACTGTTATCTTCTGCACCGGAGAGACCCTGGATGAACGCAAGGCCAATAACACTATGGAGGTAAATATTGCTCAGCTCGAGGCTCTTAAGAAGGAGATTGGAGAATCAAAGAAGTTATGGGAGAACGTTGTAATTGCCTATGAGCCGGTGTGGTCTATCGGCACGGGC

>Sweh075

GGGGTAACTTCAAGTGCAATGGATCGCTCGACTTCATTAAGAGCCACRTAGCGTCCATCGCCTCCYATAAGATCCCCGAGTCCGTGGACGTTGTTGTTGCTCCCTCCTTTGTGCACCTTTCTACAGCTATTGCGGCGAAYACCTCGAAGTGTCTGAAAATAGCAGCACAGAACGTGTATCTGGAGGGGAACGGTGCATGGACCGGCGAGACAAGCGTCGAGATGCTGCTGGACATGGGGCTGAGCCATGTAATAATAGGACACTCTGAAAGACGTAGAATCATGGGCGAGACCAATGAGCAGAGTGCTAAGAAGGCGAAGCGTGCTCTGGACAAAGGTATGACTGTTATCTTCTGCACCGGAGAGACCCTGGATGAACGCAAGGCCAATAACACTATGGAGGTGAATATTGCTCAGCTCGAGGCTCTTAAGAAGGAGATTGGAGAATCAAAGAAGTTATGGGAGAACGTTGTAATTGCCTATGAGCCGGTGTGGTCTATCGGCACGGGC

>Sweh076

~GGGTAACTTCAAGTGCAATGGATCGCTCGACTTCATTAAGAGCCACGTAGCGTCCATCGCCTCCCATAAGATCCCCGAGTCCGTGGACGTTGTTGTTGCTCCCTCCTTTGTGCACCTTTCTACAGCTATTGCGGCGAACACCTCGAAGTGTCTGAAAATAGCAGCACAGAACGTGTATCTGGAGGGGAACGGTGCATGGACCGGCGAGAYAAGCGTCGAGATGCTGCTGGACATGGGGCTGAGCCATGTAATAATAGGACACTCTGAAAGACGTAGAATCATGGGCGAGACCAATGARCAGAGTGCTAAGAAGGCGAAGCGTGCTCTGGACAAAGGTATGACTGTTATCTTCTGCACCGGAGAGACCCTGGATGAACGCAAGGCCAATAACACTATGGAGGTGAATATTGCTCAGCTCGAGGCTCTTAAGAAGGAGATTGGAGAATCAAAGAAGTTATGGGAGAACGTTGTAATTGCCTATGAGCCGGTGTGGTCTATCGGCACGGG

>Sweh079

-GGGTAACTTCAAGTGCAATGGATCGCTCGACTTCATTAAGAGCCACGTAGCGTCCATCGCCTCCYATAAGATCCCCGAGTCCGTGGACGTTGTTGTTGCTCCCTCCTTTGTGCACCTTTCTACAGCTATTGCGGCGAACACCTCGAAGTGTCTGAAAATAGCAGCACAGAACGTGTATCTGGAGGGGAACGGTGCATGGACCGGCGAGACAAGCGTCGAGATGCTGCTGGACATGGGGCTGAGCCATGTAATAATAGGACACTCTGAAAGACGTAGAATCATGGGCGAGACCAATGAGCAGAGTGCTAAGAAGGCGAAGCGTGCTCTGGACAAAGGTATGACTGTTATCTTCTGCACCGGAGAGACCCTGGATGAACGCAAGGCCAATAACACTATGGAGGTGAATATTGCTCAGCTCGAGGCTCTTAAGAAGGAGATTGGAGAATCAAAGAAGTTATGGGAGAACGTTGTAATTGCCTATGAGCCGGTGTGGTCTATCGGCACGGG-

>Sweh081

GGGGTAACTTCAAGTGCAATGGATCGCTCGACTTCATTAAGAGCCACRTAGCGTCCATCGCCTCCCATAAGATCYCCGAGTCCGTGGACGTTGTTGTTGCTCCCTCCTTTGTGCACCTTTCTACAGCTATTGCGGCRAAYACCTCGAAGTGTCTGAAAATAGCAGCACAGAACGTGTATCTGGAGGGGAACGGTGCATGGACCGGCGAGACAAGCGTCGAGATGCTGCTGGACATGGGGCTGAGCCATGTAATAATAGGACACTCTGAAAGACGTAGAATCATGGGCGAGACCAATGAGCAGAGTGCTAAGAAGGCGAAGCGTGCTCTGGACAAAGGTATGACTGTTATCTTCTGCACCGGAGAGACCCTGGATGAACGCAAGGCCAATAACACTATGGAGGTGAATATTGCTCAGCTCGAGGCTCTTAAGAAGGAGATTGGAGAATCAAAGAAGTTATGGgAGAACGTTGTAATTGCCTATGAGCCGGTGTGGTCTATCGGCACGGGC

>Sweh082

GGGGTAACTTCAAATGYAATGGATCRCTCGACTTCATTAAGAGCCACGTAGCGTCCATCGCCTCCYATAAGATCCCCGAGTCCGTGGACGTTGTTGTTGCTCCCTCCTTTGTGCACCTTTCTACAGCTATTGCGGCRAACACCTCGAAGTGTCTGAAAATAGCAGCACAGAACGTGTATCTGGAGGGGAACGGTGCATGGACCGGCGAGACAAGCGTCGAGATGCTGCTRGACATGGGGYTGAGCCATGTAATAATAGGACACTCTGAAAGACGTAGAATCATGGGCGARACCAATGAGCAGAGTGCTAAGAAGGCGAAGCGTGCTCTGGACAAAGGTATGACTGTTATCTTCTGCACCGGAGAGACCCTGGATGAACGCAAGGCCAATAACACTATGGAGGTGAATATTGCTCAGCTCGAGGCTCTTAAGAAGGAGATTGGAGAATCAAAGAAGTTATGGGAGAACGTtgtaATTGCYTATGAGCCGGTGTGGTCTATCGGCACGGGC

>Sweh083

GGGGTAACTTCAAATGCAATGGATCGCTCGACTTCATTAAGAGCCACGTAGCGTCCATCGCCTCCTATAAGATCCCCGAGTCCGTGGACGTTGTTGTTGCTCCCTCCTTTGTGCACCTTTCTACAGCTATTGCGGCGAATACTTCGAAGTGTCTGAAAATAGCAGCACAGAACGTGTATCTGGAAGGGAACGGTGCATGGACCGGCGAGACAAGCGTCGAGATGCTGCTGGACATGRGGCTGAGCCATGTAATAATAGGACACTCTGAAAGACGTAGAATCATGGGCGAGACCAATGAGCAGAGTGCTAAGAAGGCGAAGCGTGCTCTGGACAAAGGTATGACTGTTATCTTCTGCACCGGAGAGACCCTGGATGAACGCAAGGCCAATAACACTATGGAGGTGAATATTGCTCAGCTCGAGGCTCTTAAGAAGGAGATTGGAGAATCAAAGAAGTTATGGGAGAACGTTGTAATTGCCTATGAGCCGGTGTGGTCTATCGGCACGGGC

>Sweh084

GGGGTAACTTCAAATGCAATGGATCGCTCGACTTCATTAAGAGCCACGTAGCGTCCATCGCCTCCTATAAGATCCCCGAGTCCGTGGACGTTGTTGTTGCTCCCTCCTTTGTGCACCTTTCTACAGCTATTGCGGCGAATACTTCGAAGTGTCTGAAAATAGCAGCACAGAACGTGTATCTGGAAGGGAACGGTGCATGGACCGGCGAGACAAGCGTCGAGATGCTGCTGGACATGGGGCTGAGCCATGTAATAATAGGACACTCTGAAAGACGTAGAATCATGGGCGAGACCAATGAGCAGAGTGCTAAGAAGGCGAAGCGTGCTCTGGACAAAGGTATGACTGTTATCTTCTGCACCGGAGAGACCCTGGATGAACGCAAGGCCAATAACACTATGGAGGTGAATATTGCTCAGCTCGAGGCTCTTAAGAAGGAGATTGGAGAATCAAAGAAGTTATGGGAGAACGTTGTAATTGCCTATGAGCCGGTGTGGTCTATCGGCACGGG

>Sweh086

GGGGTAACTTCAAGTGCAAYGGATCGCTCGACTTCATTAAGAGCCACGTAGCGTCCATCGCCTCCCATAAGATCCCCGAGTCCGTGGACGTTGTTGTTGCTCCCTCCTTTGTGCACCTTTCTACAGCTATTGCGGCGAAYACYTCGAAGTGTCTGAAAATAGCAGCACAGAACGTGTATCTGGAGGGGAACGGTGCATGGACCGGCGAGACAAGCGTCGAGATGCTGCTGGACATGGGGCTGAGCCATGTAATARTAGGACACTCTGAAAGRCGTAGAATCATGGGCGAGACCAATGAGCAGAGTGCYAAGAAGGCGAAGCGTGCTCTGGACAAAGGTATGACTGTTATCTTCTGCACCGGAGAGACCCTGGATGAACGCAAGGCCAATAACACTATGGAGGTGAATATTGCTCAGCTCGAGGCTCTTAAGAAGGAGATTGGAGAATCAAAGAAGTTATGGGAGAACGTTGTAATTGCCTATGAGCCGGTGTGGTCTATCGGCACGGG

>Sweh088

GGGGTAACTTCAAGTGCAATGGATCGCTCGACTTCATTAAGAGCCACGTAGCGTCCATCGCCTCCCATAAGATCCCCGAGTCCGTGGAYGTTGTTGTTGCTCCCTCCTTTGTGCACCTTTCTACAGCTATTGCGGCGAATACCTCGAAGTGTCTGAAAATAGCAGCACAGAACGTGTATYTGGARGGGAACGGTGCATGGACCGGCGAGACAAGCGTCGAGATGCTGCTGGACATGGGGCTGAGCCATGTAATAATAGGACACTCTGAAAGACGTAGAATCATGGGCGAGACCAATGAGCAGAGTGCTAAGAAGGCGAAGCGTGCTCTGGACAAAGGTATGACTGTTATCTTCTGCACCGGAGAGACCCTGGATGAACGCAAGGCCAATAACACTATGGAGGTGAATATTGCTCAGCTCGAGGCTCTTAAGAAGGAGATTGGAGAATCAAAGAAGTTATGGGAGAACGTTGTAATTGCCTATGAGCCGGTGTGGTCTATCGGCACGGGC

>Sweh089

GGGGTAACTTCAARTGCAATGGATCGCTCGACTTCATTAAGAGCCACGTAGCGTCCATCGCCTCCCATAAGATCCCCGAGTCCGTGGACGTTGTTGTTGCTCCCTCCTTTGTGCACCTTTCTACAGCTATTGCGGCGAAyACCTCGAAGTGTCTGAAAATAGCAGCACAGAACGTGTATCTGGAGGGGAACGGTGCATGGACCGGCGAGACAAGCGTCGAGATGCTGCTGGACATGGGGCTGAGCCATGTAATAATAGGACACTCTGAAAGACGTAGAATCATGGGCGAGACCAATGAGcAGAGTGCTAAGAAGGCGAAGCGTGCTCTGGACAAAGGTATGACTGTTATCTTCTGCACCGGAGAGACCCTGGATGAACGCAAGGCCAATAACACTATGGAGGTGAATATTGCTCAGCTCGAGGCTCTTAAGAAGGAGATTGGAGAATCAAAGAAGTTATGGGAGAACGTTGTAATTGCCTATGAGCCGGTGTGGTCTATCGGCACGGGC

>Sweh090

-GGGTAACTTCAARTGCAATGGATCRCTCGACTTCATTAAGAGCCACGTAGCGTCCATCGCCTCCTATAAGATCCCCGAGTCCGTGGACGTTGTTGTTGCTCCCTCCTTTGTGCACCTTTCTACAGCTATTGCGGCRAAYACYTCGAAGTGTCTGAAAATAGCAGCACAGAACGTGTATCTGGAGGGGAACGGTGCATGGACCGGCGAGACAAGCGTCGAGATGCTGCTGGACATGGGGCTGAGCCATGTAATAATAGGACACTCTGAAAGACGTAGAATCATGGGCGAGACCAATGAGCAGAGTGCTAAGAAGGCGAAGCGTGCTCTGGACAAAGGTATGACTGTTATCTTCTGCACCGGAGAGACCCTGGATGAACGCAAGGCCAATAACACTATGGAGGTGAATATTGCTCAGCTCGAGGCTCTTAAGAAGGAGATTGGAGAATCAAAGAAGTTATGGGAGAACGTTGTAATTGCYTATGAGCCGGTGTGGTCTATCGGCACGGG-

>Sweh091

~~~~~~~~TTCAARTGCAAYGGATCGCTCGACTTCATTAAGAGCCACGTAGCGTCCATSGCCTCCYATAAGATCCCCGAGTCCGTGGACGTTGTTGTTGCTCCCTCCTTTGTGCACCTTTCTACAGCTATTGCGGCGAAYACYTCGAAGTGTCTGAAAATAGCAGCACAGAACGTGTATCTGGARGGGAACGGTGCATGGACCGGCGAGACAAGCGTCGAGATGCTGCTGGACATGGGGCTGAGYYATGTAATAATAGGACACTCTGAAAGACGTAGAATCATGGGCGAGACCAATGAGCAGAGTGCYAAGAAGGCGAAGCGTGCTCTGGAYAAAGGTATGACTGTTATCTTCTGCACCGGAGAGACCCTGGATGAACGCAAGGCCAATAACACTATGGAGGTGAATATTGCTCAGCTCGAGGCTCTTAAGAAGGAGATTGGAGAATCAAAGAAGTTATGGGAGAACGTTGTAATTGCCTATGAGYCGGTGTGGTCTATCGGCACGGG

>Sweh092

-GGGTAACTTCAAGTGCAATGGATCGCTCGACTTCATTAAGAGCCACGTAGCGTCCATCGCCTCCYATAAGATCCCCGAGTCCGTGGACGTTGTTGTTGCTCCCTCCTTTGTGCACCTTTCTACAGCTATTGCGGCGAAYACYTCGAAGTGTCTGAAAATAGCAGCACAGAACGTGTATCTGGAGGGGAACGGTGCATGGACCGGCGAGACAAGCGTCGAGATGCTGCTGGACATGGGGCTGAGCCATGTAATAATAGGACACTCTGAAAGACGTAGAATCATGGGCGAGACCAATGAGCAGAGTGCTAAGAAGGCGAAGCGTGCTCTGGACAAAGGTATGACTGTTATCTTCTGCACCGGAGAGACCCTGGATGAACGCAAGGCCAATAACACTATGGAGGTGAATATTGCTCAGCTCGAGGCTCTTAAGAAGGAGATTGGAGAATCAAAGAAGTTRTGGGAGAACGTTGTAATTGCCTATGAGCCGGTGTGGTCTATCGGCACGG--

>Sweh093

GGGGTAACTTCAAGTGCAAYGGATCGCTCGACTTCATTAAGAGCCACGTAGCGTCCATCGCCTCCCATAAGATCCCCGAGTCCGTGGACGTTGTTGTTGCTCCCTCCTTTGTGCACCTTTCTACAGCTATTGCGGCGAACACCTCGAAGTGTCTGAAAATAGCAGCACAGAACGTGTATCTGGARGGGAACGGTGCATGGACCGGCGAGACAAGCGTCGAGATGCTGCTGGACATGGGGCTGAGCCATGTAATAATAGGACACTCTGAAAGACGTAGAATCATGGGCGAGACCAATGAGCAGAGTGCTAAGAAGGCGAAGCGTGCTCTGGACAAAGGTATGACTGTTATCTTCTGCACCGGAGAGACCCTGGATGAACGCAAGGCCAATAACACTATGGAGGTGAATATTGCTCAGCTCGAGGCTCTTAAGAAGGAGATTGRAGAATCAAAGAAGTTATGGGAGAACGTTGTAATTGCCTATGAGCCGGTGTGGTCTATCGGCACGGGC

>Sweh094

GGGGTAACTTCAAGTGCAATGGATCGCTCGACTTCATTAAGAGCCACGTAGCGTCCATCGCCTCCCATAAGATCCCCGAGTCCGTGGACGTTGTTGTTGCTCCCTCCTTTGTGCACCTTTCTACAGCTATTGCGGCGAACACCTCGAAGTGTCTGAAAATAGCAGCACAGAACGTGTATCTGGAGGGGAACGGTGCATGGACCGGCGAGACAAGCGTCGAGATGCTGCTGGACATGGGGCTGAGCCATGTAATAATAGGACACTCTGAAAGACGTAGAATCATGGGCGAGACCAATGAGCAGAGTGCTAAGAAGGCGAAGCGTGCTCTGGACAAAGGTATGACTGTTATCTTCTGCACCGGAGAGACCCTGGATGAACGCAAGGCCAATAACACTATGGAGGTGAATATTGCTCAGCTCGAGGCTCTTAAGAAGGAGATTGGAGAATCAAAGAAGTTATGGGAGAACGTTGTAATTGCCTATGAGCCGGTGTGGTCTATCGGCACGGGC

>Sweh095

~~~GTAACTTCAAGTGCAAYGGATCGCTCGACTTCATTaAGAGCCACGTAGCGTCCATCGCCTCCYATAAGATCCCCGAGTCCGTGGACGTTGTTGTTGCTCCCTCCTTTGTGCACCTTTCTACAGCTATTGCGGCGAAYACYTCGAAGTGTCTGAAAATAGCAGCACAGAACGTGTATCTGGARGGGAAYGGTGCATGGACCGGCGAGACAAGCGTCGAGATGCTGCTGGACATGGGGCTGARCYATGTAATAATAGGACACTCTGAAAGACGTAGAATCATGGGCGAGACCAATGAGCAGAGTGCTAAGAAGGCGAAGCGTGCTCTGGACAAAGGTATGACTGTTATCTTCTGCACCGGAGAGACCCTGGATGAACGCAAGGCCAATAACACTATGGAGGTRAATATTGCTCAGCTCGAGGCTCTTAAGAAGGAGATTGGAGAATCAAAGAAGTTATGGGAGAACGTTGTAATTGCYTATGAGCCGGTGTGGTCTATCGGCACGG

>Sweh102

-GGGTAACTTCAAGTGCAATGGATCGCTCGACTTCATTAAGAGCCACGTAGCGTCCATCGCCTCCCATAAGATCCCCGAGTCCGTGGACGTTGTTGTTGCTCCCTCCTTTGTGCACCTTTCTACAGCTATTGCGGCGAACACCTCGAAGTGTCTGAAAATAGCAGCACAGAACGTGTATCTGGAGGGGAACGGTGCATGGACCGGCGAGACAAGCGTCGAGATGCTGCTGGACATGGGGCTGAGCCATGTAATAATAGGACACTCTGAAAGACGTAGAATCATGGGCGAGACCAATGAGCAGAGTGCTAAGAAGGCGAAGCGTGCTCTGGACAAAGGTATGACTGTTATCTTCTGCACCGGAGAGACCCTGGATGAACGCAAGGCCAATAACACTATGGAGGTGAATATTGCTCAGCTCGAGGCTCTTAAGAAGGAGATTGGAGAATCAAAGAAGTTATGGGAGAACGTTGTAATTGCCTATGAGCCGGTGTGGTCTATCGGCACG---

>Sweh103

~~GGTAACTTCAAGTGCAATGGATCGCTCGACTTCATTAAGAGCCACGTAGCGTCCATCGCCTCCCATAAGATCCCCGAGTCCGTGGACGTTGTTGTTGCTCCCTCCTTTGTGCACCTTTCTACAGCTATTGCGGCGAACACCTCGAAGTGTCTGAAAATAGCAGCACAGAACGTGTATCTGGAGGGGAACGGTGCATGGACCGGCGAGACAAGCGTCGAGATGCTGCTGGACATGGGGCTGAGCCATGTAATAATAGGACACTCTGAAAGACGTAGAATCATGGGCGAGACCAATGAGCAGAGTGCTAAGAAGGCGAAGYGTGCTCTGGACAAAGGTATGACTGTTATCTTCTGCACCGGAGAGACCCTGGATGAACGCAAGGCCAATAACACTATGGAGGTGAATATTGCTCAGCTCGAGGCTCTTAAGAAGGAGATTGGAGAATCAAAGAAGTTATGGGAGRACGTTGTAATTGCYTATGAGCCGGTGTGGTCTATCGGCACGGGC

>Sweh105

-GGGTAACTTCAAATGCAATGGATCGCTCGACTTCATTAAGAGCCACGTAGCGTCCATCGCCTCCTATAAGATCCCCGAGTCCGTGGACGTTGTTGTTGCTCCCTCCTTTGTGCACCTTTCTACAGCTATTGCGGCGAATACTTCGAAGTGTCTGAAAATAGCAGCACAGAACGTGTATCTGGAGGGGAACGGTGCATGGACCGGCGAGACAAGCGTCGAGATGCTGCTGGACATGGGGCTGAGCCATGTAATAATAGGACACTCTGAAAGACGTAGAATCATGGGCGAGACCAATGAGCAGAGTGCTAAGAAGGCGAAGCGTGCTCTGGACAAAGGTATGACTGTTATCTTCTGCACCGGAGAGACCCTGGATGAACGCAAGGCCAATAACACTATGGAGGTGAATATTGCTCAGCTCGAGGCTCTTAAGAAGGAGATTGGAGAATCAAAGAAGTTATGGGAGAAYGTTGTAATTGCCTATGAGCCGGTRTGGTCTATCGGCACGGG-

>Sweh106

GGGGTAACTTCAAGTGCAATGGATCGCTCGACTTCATTAAGAGCCACGTAGCGTCCATCGCCTCCCATAAGATCCCCGAGTCCGTGGACGTTGTTGTTGCTCCCTCCTTTGTGCACCTTTCTACAGCTATTGCGGCRAACACCTCGAAGTGTCTGAAAATAGCAGCACAGAACGTGTATCTGGAGGGGAACGGTGCATGGACCGGCGAGACAAGCGTCGAGATGCTGCTGGACATGGGGCTGAGCCATGTAATAATAGGACACTCTGAAAGACGTAGAATCATGGGCGAGACCAATGAGCAGAGTGCTAAGAAGGCGAAGCGTGCTCTGGACAAAGGTATGACTGTTATCTTCTGCACCGGAGAGACCCTGGATGAACGCAAGGCCAATAACACTATGGAGGTGAATATTGCTCAGCTCGAGGCTCTTAAGAAGGAGATTGGAGAATCAAAGAAGTTATGGGAGAACGTTGTAATTGCCTATGAGCCGGTGTGGTCTATCGGCACGGG

>Sweh107

~GGGTAACTTCAAATGCAATGGATCGCTCGACTTCATTAAGAGCCACGTAGCGTCCATCGCCTCCCATAAGATCCCCGAGTCCGTGGACGTTGTTGTTGCTCCCTCCTTTGTGCACCTTTCTACAGCTATTGCGGCAAACACCTCGAAGTGTCTGAAAATAGCAGCACAGAACGTGTATCTGGAGGGGAACGGTGCATGGACCGGCGAGACAAGCGTCGAGATGCTGCTGGACATGGGGCTGAGCCATGTAATAATAGGACACTCTGAAAGGCGTAGAATCATGGGCGAGACCAATGAGCAGAGTGCTAAGAAGGCGAAGCGTGCTCTGGACAAAGGTATGACTGTTATCTTCTGCACTGGAGAGACCCTGGATGAACGCAAGGCCAATAACACTATGGAGGTGAATATTGCTCAGCTCGAGGCTCTTAAGAAGGAGATTGGAGAATCAAAGAAGTTATGGGAGAACGTTGTAATTGCCTATGAGCCGGTGTGGTCTATCGGCACGGGC

>Sweh111

GGGGTAACTTCAAATGCAATGGATCGCTCGACTTCATTAMGAGCCACGTAGCGTYCATCGCCTCCCATAAGATCCCCGAGTCCGTGGACGTTGTTGTTGCTCCCTCCTTTGTGCACCTTTCTACAGCTATTGCGGCGAACACCTCGAAGTGTCTGAAAATAGCAGCACAGAACGTGTATCTGGAGGGGAACGGTGCATGGACCGGCGAGACAAGCGTCGAGATGCTGCTGGACATGGGGCTGAGCCATGTAATAATAGGACACTCTGAAAGACGTAGAATCATGGGCGAGACCAATGAGCAGAGTGCTAAGAAGGCGAAGCGTGCTCTGGACAAAGGTATGACTGTTATCTTCTGCACCGGAGAGACCCTGGATGAACGCAAGGCCAATAACACTATGGAGGTGAATATTGCTCAGCTCGAGGCTCTTAAGAAGGAGATTGGAGAATCAAAGAAGTTATGGGAGAACGTTGTAATTGCCTATGAGCCGGTGTGGTCTATCGGCACGG

>Sweh112

GGGGTAACTTCAAGTGCAATGGATCGCTCGACTTCATTAAGAGCCACGTAGCGTCCATCGCCTCCCATAAGATCCCCGAGTCCGTGGACGTTGTTGTTGCTCCCTCCTTTGTGCACCTTTCTACAGCCATTGCGGCGAACACCTCGAAGTGTCTGAAAATAGCAGCACAGAACGTGTATCTGGAGGGGAACGGTGCATGGACCGGCGAGACAAGCGTCGAGATGCTGCTGGACATGGGGCTGAGCCATGTAATAATAGGACACTCTGAAAGACGTAGAATCATGGGCGAGACCAATGAGCAGAGTGCTAAGAAGGCGAAGCGTGCTCTGGACAAAGGTATGACTGTTATCTTCTGCACCGGAGAGACCCTGGATGAACGCAAGGCCAATAACACTATGGAGGTGAATATTGCTCAGCTCGAGGCTCTTAAGAAGGAGATTGGAGAATCAAAGAAGTTATGGGAGAACGTTGTAATTGCCTATGAGCCGGTGTGGTCTATCGGCACGGGC

>Sweh113

-GGGTAACTTCAARTGCAATGGATCGCTCGACTTCATTAAGAGCCACGTAGCGTCCATCGCCTCCYATAAGATCCCCGAGTCCGTGGACGTTGTTGTTGCTCCCTCCTTTGTGCACCTTTCTACAGCTATTGCGGCGAACACCTCGAAGTGTCTGAAAATAGCAGCACAGAACGTGTATCTGGAGGGGAACGGTGCATGGACCGGCGAGACAAGCGTCGAGATGCTGCTGGACATGGGGCTGAGCCATGTAATAATAGGACACTCTGAAAGACGTAGAATCATGGGCGAGACCAATGAGCAGAGTGCTAAGAAGGCGAAGCGTGCTCTGGACAAAGGTATGACTGTTATCTTCTGCACCGGAGAGACCCTGGATGAACGCAAGGCCAATAACACTATGGAGGTGAATATTGCTCAGCTCGAGGCTCTTAAGAAGGAGATTGGAGAATCAAAGAAGTTATGGGAGAACGTTGTAATTGCCTATGAGCCGGTGTGGTCTATCGGCACGGG-

>Sweh115

GGGGTAACTTCAAGTGCAATGGATCGCTCGACTTCATTAAGAGCCACGTAGCGTCCATCGCCTCCCATAAGATCYCCGAGTCCGTGGACGTTGTTGTTGCTCCCTCCTTTGTGCACCTTTCTACAGCTATTGCGGCRAACACCTCGAAGTGTCTGAAAATAGCAGCACAGAACGTGTATCTGGAGGGGAACGGTGCATGGACCGGCGAGACAAGCGTCGAGATGCTGCTGGACATGGGGCTGAGCCATGTAATAATAGGACACTCTGAAAGACGTAGAATCATGGGCGAGACCAATGAGCAGAGTGCTAAGAAGGCGAAGCGTGCTCTGGACAAAGGTATGACTGTTATCTTCTGCACCGGAGAGACCCTGGATGAACGCAAGGCCAATAACACTATGGAGGTGAATATTGCTCAGCTCGAGGCTCTTAAGAAGGAGATTGGAGAATCAAAGAAGTTATGGGAGAACGTTGTAATTGCCTATGAGCCGGTGTGGTCTATCGGCACGGGC

>Sweh116

GGGGTAACTTCAAGTGCAATGGATCGCTCGACTTCATTAAGAGCCACGTAGCGTCCATCGCCTCCCATAAGATCCCCGAGTCCGTGGACGTTGTTGTTGCTCCCTCCTTTGTGCACCTTTCTACAGCTATTGCGGCRAACACCTCGAAGTGTCTGAAAATAGCAGCACAGAACGTGTATCTGGAGGGGAACGGTGCATGGACCGGCGAGACAAGCGTCGAGATGCTGCTGGACATGGGGCTGAGCCATGTAATAATAGGACACTCTGAAAGACGTAGAATCATGGGCGAGACCAATGAGCAGAGTGCTAAGAAGGCGAAGCGTGCTCTGGACAAAGGTATGACTRTTATCTTCTGCACCGGAGAGACCCTGGATGAACGCAAGGCCAATAACACTATGGAGGTGAATATTGCTCAGCTCGAGGCTCTTAAGAAGGAGATTGGAGAATCAAAGAAGTTATGGGAGAACGTTGTAATTGCYTATGAGCCGGTGTGGTCTATCGGCACGGGC

>Sweh117

GGGGTAACTTCAAATGCAATGGATCGCTCGACTTCATTAMGAGCCACGTAGCGTCCATCGCCTCCCATAAGATCCCCGAGTCCGTGGACGTTGTTGTTGCTCCCTCCTTTGTGCACCTTTCTACAGCTATTGCGGCRAACACCTCGAAGTGTCTGAAAATAGCAGCACAGAACGTGTATCTGGAGGGGAACGGTGCATGGACCGGCGAGACAAGCGTCGAGATGCTGCTGGACATGGGGCTGAGCCATGTAATAATAGGACACTCTGAAAGACGTAGAATCATGGGCGARACCAATGAGCAGAGTGCTAAGAAGGCGAAGCGTGCTCTGGACAAAGGTATGACTGTTATCTTCTGCACCGGAGAGACCCTGGATGAACGCAAGGCCAATAACACTATRGAGGTGAATATTGCTCAGCTCGAGGCTCTTAAGAAGGAGATTGGAGAATCAAAGAAGTTATGGGAGAACGTTGTAATTGCCTATGAGCCGGTGTGGTCTATCGGCACGGGC

>Sweh118

GGGGTAACTTCAARTGCAATGGATCGCTCGACTTCATTAAGAGCCACGTAGCGTCCATCGCCTCCYATAAGATCCCCGAGTCCGTGGACGTTGTTGTTGCTCCCTCCTTTGTGCACCTTTCTACAGCTATTGCGGCGAAYACYTCGAAGTGTCTGAAAATAGCAGCACAGAACGTGTATCTGGAAGGGAACGGTGCATGGACCGGCGAGACAAGCGTCGAGATGCTGCTGGACATGGGGCTGAGCCATGTAATAATAGGACACTCTGAAAGACGTAGAATCATGGGYGAGACCAATGAGCAGAGTGCTAARAAGGCGAAGCGTGCTCTGGACAAAGGTATGACTGTTATCTTCTGCACCGGAGAGACCCTGGATGAACGCAAGGCCAATAACACTATGGAGGTGAATATTGCTCAGCTCGAGGCTCTTAAGAAGGAGATTGGAGAATCAAAGAAGTTATGRGAGAACGTTGTAATTGCCTATGAGCCGGTGTGGTCTATCGGCACGGGC

>Sweh119

-GGGTAACTTCAAATGCAATGGATCGCTCGACTTCATTAAGAGCCACGTAGCGTCCATCGCCTCCTATAAGATCCCCGAGTCCGTGGACGTTGTTGTTGCTCCCTCCTTTGTGCACCTTTCTACAGCTATTGCGGCGAATACTTTGAAGTGTCTGAAAATAGCAGCACAGAACGTGTATCTGGAGGGGAACGGTGCATGGACCGGCGAGACAAGCGTCGAGATGCTGCTGGACATGGGGCTGAGCCATGTAATAATAGGACACTCTGAAAGACGTAGAATCATGGGCGAGACCAATGAGCAGAGTGCTAAGAAGGCGAAGCGTGCTCTGGACAAAGGTATGACTGTTATCTTCTGCACCGGAGAGACCCTGGATGAACGCAAGGCCAATAACACTATGGAGGTGAATATTGCTCAGCTCGAGGCTCTTAAGAAGGAGATTGGAGAATCAAAGAAGTTATGGGAGAACGTTGTAATTGCCTATGAGCCGGTGTGGTCTATCGGCACGGG-

>Sweh121

GGGGTAACTTCAARTGCAATGGATCGCTCGACTTCATTAAGAGCCACGTAGCGTCCATCGCCTCCYATAAGATCCCCGAGTCCGTGGACGTTGTTGTTGCTCCCTCCTTTGTGCACCTTTCTACAGCTATTGCGGCRAAYACCTCGAAGTGTCTGAAAATAGCAGCACAGAACGTGTATCTGGAGGGGAACGGTGCATGGACCGGCGAGACAAGCGTCGAGATGCTGCTGGACATGGRGCTGAGCCATGTAATAATAGGACACTCTGAAAGACGTAGAATCATGGGCGAGACCAATGAGCAGAGTGCTAAGAAGGCGAAGCGTGCTCTGGACAAAGGTATGACTGTTATCTTCTGCACCGGAGAGACCCTGGATGARCGCAAGGCCAATAACACTATGGAGGTGAATATTGCYCAGCTCGAGGCTCTTAAGAAGGAGATTGGAGAATCAAAGAAGTTATGGGAGAACGTTRTAATTGCYTATGAGCCGGTGTGGTCTATCGGCACGGGC

>Sweh123

~~GGTAACTTCAAGTGCAATGGATCGCTCGACTTCATTAAGAGCCACGTAGCGTCCATCGCCTCCYATAAGATCCCCGAGTCCGTGGACGTTGTTGTTGCTCCCTCCTTTGTGCACCTTTCTACAGCTATTGCGGCRAAYACYTCGAAGTGTCTGAAAATAGCAGCACAGAACGTGTATCTGGARGGGAACGGTGCATGGACCGGCGAGACAAGCGTCGAGATGCTGCTGGACATGGGGCTGAGCCATGTAATAATAGGACACTCTGAAAGACGTAGAATCATGGGCGAGACCAATGAGCAGAGTGCTAAGAAGGCGAAGCGTGCTCTGGACAAAGGTATGACTGTTATCTTCTGCACCGGAGAGACCCTGGATGAACGCAAGGCCAATAACACTATGGAGGTGAATATTGCTCAGCTCGAGGCTCTTAAGAAGGAGATTGGAGAATCAAAGAAGTTATGGGAGAACGTTGTAATTGCYTATGAGCCGGTGTGGTCTATCGGCACGGG

>Sweh124

GGGGTAACTTCAARTGCAATGGATCRCTCGACTTCATTAAGAGCCACGTAGCGTCCATCGCCTCCYATAAGATCCCCGAGTCCGTGGACGTTGTTGTTGCTCCCTCCTTTGTGCACCTTTCTACAGCTATTGCGGCRAACACCTCGAAGTGTCTGAAAATAGCAGCACAGAACGTGTATCTGGAGGGGAACGGTGCATGGACCGGCGAGACAAGCGTCGAGATGCTGCTGGACATGGGGCTGAGCYATGTAATARTAGGACACTCTGAAAGACGTAGAATCATGGGCGAGACCAATGAGCAGGGTGCTAARAAGGCGAAGCGTGCTCTGGACAAAGGTATGACTGTTATCTTYTGCACCGGAGAGACCCTGGATGARCGCAAGGCCAATAACACTATGGAGGTGAATATTGCTCAGCTCGAGGCTCTTAAGAAGGAGATTGGAGAMTCAAAGAAGTTRTGGGAGAACGTTGTAATTGCYTATGARCCGGTGTGGTCTATCGGCACGGGC

>Sweh126

GGGGTAACTTCAAATGCAATGGATCGCTCGACTTCATTARGAGCCACGTAGCGTCCATCGCCTCCYATAAGATCCCCGAGTCCGTGGACGTTGTTGTTGCTCCCTCCTTTGTGCACCTTTCTACAGCTATTGCGGCGAAYACYTCGAAGTGTCTGAAAATAGCAGCACAGAACGTGTATCTGGARGGGAACGGTGCATGGACCGGCGAGACAAGCGTCGAGATGCTGCTGGACATGGGGCTGAGCCATGTAATAATAGGACACTCTGAAAGACGTAGAATCATGGGCGAGACCAATGAGCAGAGTGCTAAGAAGGCGAAGCGTGCTCTGGACAAAGGTATGACTGTTATCTTCTGCACCGGAGAGACCCTGGATGAACGCAAGGCCAATAACACTATGGAGGTGAATATTGCTCAGCTCGAGGCTCTTAAGAAGGAGATTGGAGAATCAAAGAAGTTATGGGAGAACGTTGTAATTGCCTATGAGCCGGTGTGGTCTATCGGCACGGGC

>Sweh127

GGGGTAACTTCAARTGCAATGGATCGCTCGACTTCATTAAGAGCCACGTAGCGTCCATCGCCTCCCATAAGATCCCCGAGTCCGTGGACGTTGTTGTTGCTCCCTCCTTTGTGCACCTTTCTACAGCTATTGCGGCGAACACCTCGAAGTGTCTGAAAATAGCAGCACAGAACGTGTATCTGGAGGGGAACGGTGCATGGACCGGCGAGACAAGCGTCGAGATGCTGCTGGACATGGGGCTGAGCCATGTAATAATAGGACACTCTGAAAGACGTAGAATCATGGGCGAGACCAATGAGCAGAGTGCTAAGAAGGCGAAGCGTGCTCTGGACAAAGGTATGACTGTTATCTTCTGCACCGGAGAGACCCTGGATGAACGCAAGGCCAATAACACTATGGAGGTGAATATTGCTCAGCTCGAGGCTCTTAAGAAGGAGATTGGAGAATCAAAGAAGTTATGGGAGAACGTtGTAATTGCCTATGAGCCGGTGTGGTCTATCGGCACGGG

>Sweh136

GGGGTAACTTCAAGTGCAATGGATCGCTCGACTTCATTAAGAGCCACGTAGCGTCCATCGCCTCCCATAAGATCCCCGAGTCCGTGGACGTTGTTGTTGCTCCCTCCTTTGTGCACCTTTCTACAGCTATTGCGGCGAACACCTCGAAGTGTCTGAAAATAGCAGCACAGAACGTGTATCTGGAGGGGAACGGTGCATGGACCGGCGAGACAAGCGTCGAGATGCTGCTGGACATGGGGCTGAGCCATGTAATAATAGGACACTCTGAAAGACGTAGAATCATGGGCGAGACCAATGAGCAGAGTGCTAAGAAGGCGAAGCGTGCTCTGGACAAAGGTATGACTGTTATCTTCTGCACCGGAGAGACCCTGGATGAACGCAAGGCCAATAACACTATGGAGGTGAATATTGCTCAGCTCGAGGCTCTTAAGAAGGAGATTGGAGAATCAAAGAAGTTATGGGAGAACGTTGTAATTGCCTATGAGCCGGTGTGGTCTATCGGCACGGGC

>Sweh137

GGGGTAACTTCAAGTGCAATGGATCGCTCGACTTCATTARGAGCCACGTAGCGTCCATCGCCTCCCATAAGATCCCCGAGTCCGTGGACGTTGTTGTTGCTCCCTCCTTTGTGCACCTTTCTACAGCTATTGCGGCGAAYACCTCGAAGTGTCTGAAAATAGCAGCACAGAACGTGTATCTGGAGGGGAACGGTGCATGGACCGGCGAGACAAGCGTCGAGATGCTGCTGGAYATGGGGCTGAGCCATGTAATAATAGGACACTCTGAAAGACGTAGAATCATGGGCGAGACCAATGAGCAGAGTGCTAAGAAGGCGAAGCGTGCTCTGGACAAAGGTATGACTGTTATCTTCTGCACCGGAGAGACCCTGGATGAACGCAAGGCCAATAACACTATGGAGGTGAATATTGCTCAGCTCGAGGCTCTTAAGAAGGAGATTGGAGAATCAAAGAAGTTATGGGAGAACGTTGTAATTGCCTATGAGCCGGTGTGGTCTATCGGCACGGGC

>Sweh141

-GGGTAACTTCAAATGCAATGGATCGCTCGACTTCATTAAGAGCCACGTAGCGTCCATCGCCTCCYATAAGATCCCCGAGTCCGTGGACGTTGTTGTTGCTCCCTCCTTTGTGCACCTTTCTACAGCTATTGCGGCRAACACCTCGAAGTGTCTGAAAATAGCAGCACAGAACGTGTATCTGGAGGGGAACGGTGCATGGACCGGCGAGACAAGCGTCGAGATGCTGCTGGACATGGGGCTGAGCCATGTAATAATAGGACACTCTGAAAGACGTAGAATCATGGGCGAGACCAATGAGCAGAGTGCTAAGAAGGCGAAGCGTGCTCTGGACAAAGGTATGACTGTTATCTTCTGCACCGGAGAGACCCTGGATGAACGCAAGGCCAATAACACTATGGAGGTGAATATTGCTCAGCTCGAGGCTCTTAAGAAGGAGATTGGAGAATCAAAGAAGTTATGGGAGAACGTTGTAATTGCCTATGAGCCGGTGTGGTCTATCGGCACGG--

>Sweh142

-GGGTAACTTCAAGTGCAATGGATCGCTCGACTTCATTAAGAGCCACGTAGCGTCCATCGCCTCCCATAAGATCCCCGAGTCCGTGGACGTTGTTGTTGCTCCCTCCTTTGTGCAYCTTTCTACAGCTATTGCGGCAAACACCTCGAAGTGTCTGAAAATAGCAGCACAGAACGTGTATCTGGAGGGGAACGGTGCATGGACCGGCGAGAYAAGCGTCGAGATGCTGYTGGACATGGGGCTGAGCCATGTAATAATAGGACACTCTGAAAGACGTAGAATCATRGGCGAGACCAATGAGCAGAGTGCTAAGAAGGCGAAGCGTGCTCTGGACAAAGGTATGACTGTTATCTTCTGCACCGGAGAGACCCTGGATGAACGCAAGGCCAATAACACTATGGAGGTGAATATTGCTCAGCTCGAGGCTCTTAAGAAGGAGATTGGAGAATCAAAGAAGTTATGGGAGAACGTTGTAATTGCCTATGAGCCGGTGTGGTCTATCGGCACGGGC

>Sweh143

GGGGTAACTTCAAGTGCAATGGATCGCTCGACTTCATTAAGAGCCACGTAGCGTCCATCGCCTCCCATAAGATCCCCGAGTYCGTGGACGTTGTTGTTGCTCCCTCCTTTGTGCACCTTTCTACAGCTATTGCGGCGAAYACYTCGAAGTGTCTGAAAATAGCAGCACAGAACGTGTATCTGGAGGGGAACGGTGCATGGACCGGCGAGACAAGCGTCGAGATGCTGCTGGACATGGGGCTGAGCYATGTAATAATAGGACACTCTGAAAGACGTAGAATCATGGGCGAGACCAATGAGCAGAGTGCTAAGAAGGCGAAGCGTGCTCTGGACAAAGGTATGACTGTTATCTTCTGCACCGGAGAGACCCTGGATGAACGCAAGGCCAATAACACTATGGAGGTGAATATTGCTCAGCTCGAGGCTCTTAAGAAGGAGATTGGAGAATCAAAGAAGTTATGGGAGAACGTTGTAATTGCCTATGAGCCGGTGTGGTCTATCGGCACGGGC

>Sweh144

GGGGTAACTTCAAATGCAATGGATCGCTCGACTTCATTAAGAGCCACGTAGCGTCCATCGCCTCCTATAAGATCCCCGAGTCCGTGGACGTTGTTGTTGCTCCCTCCTTTGTGCACCTTTCTACAGCTATTGCGGCGAATACTTCGAAGTGTCTGAAAATAGCAGCACAGAACGTGTATCTGGAAGGGAACGGTGCATGGACCGGCGAGACAAGCGTCGAGATGCTGCTGGACATGGGGCTGAGCCATGTAATAATAGGACACTCTGAAAGACGTAGAATCATGGGCGAGACCAATGAGCAGAGTGCTAAGAAGGCGAAGCGTGCTCTGGACAAAGGTATGACTGTTATCTTCTGCACCGGAGAGACCCTGGATGAACGCAAGGCCAATAACACTATGGAGGTGAATATTGCTCAGCTCGAGGCTCTTAAGAAGGAGATTGGAGAATCAAAGAAGTTATGGGAGAACGTTGTAATTGCCTATGAGCCGGTGTGGTCTATCGGCACGGGC

>Sweh148

-GGGTAACTTCAAGTGCAATGGATCGCTCGACTTCATTAAGAGYCACGTAGCGTCCATCGCCTCCCATAAGATCCCCGAGTCCGTGGACGTTGTTGTTGCTCCCTCCTTTGTGCACCTTTCTACAGCTATTGCGGCGAACACCTCGAAGTGTCTGAAAATAGCAGCACAGAACGTGTATCTGGAGGGGAACGGTGCATGGACCGGCGAGACAAGCGTCGAGATGCTGCTGGACATGGGGCTGAGCCATGTAATAATAGGACACTCTGAAAGACGTAGAATCATGGGCGAGACCAATGAGCAGAGTGCTAAGAAGGCGAAGCGTGCTCTGGACAAAGGTATGACTGTTATCTTCTGCACCGGAGAGACCCTGGATGAACGCAAGGCCAATAACACTATGGAGGTGAATATTGCTCAGCTCGAGGCTCTTAAGAAGGAGATTGGAGAATCAAAGAAGTTATGGGAGAACGTTGTAATTGCYTATGAGCCGGTGTGGTCTATCGGCACGGG-

>Sweh149

-GGGTAACTTCAARTGCAATGGATCRCTCGACTTCATTAAGAGCCACGTAGCGTCCATCGCCTCCTATAAGATCCCCGAGTCCGTGGACGTTGTTGTTGCTCCCTCCTTTGTGCACCTTTCTACAGCTATTGCGGCRAAYACYTCGAAGTGTCTGAAAATAGCAGCACAGAACGTGTATCTGGAGGGGAACGGTGCATGGACCGGCGAGACAAGCGTCGAGATGCTGCTGGACATGGGGCTGAGCCATGTAATAATAGGACACTCTGAAAGACGTAGAATCATGGGCGAGACCAATGAGCAGAGTGCTAAGAAGGCGAAGCGTGCTCTGGACAAAGGTATGACTGTTATCTTCTGCACCGGAGAGACCCTGGATGAACGCAAGGCCAATAACACTATGGAGGTGAATATTGCTCAGCTCGAGGCTCTTAAGAAGGAGATTGGAGAATCAAAGAAGTTATGGGAGAACGTTGTAATTGCCTATGAGCCGGTGTGGTCTATCGGCACGGG-

>Sweh151

-GGGTAACTTCAARTGCAATGGATCGCTCGACTTCATTAAGAGCCACGTAGCGTCCATCGCCTCCYATAAGATCCCCGAGTCCGTGGACGTTGTTGTTGCTCCCTCCTTTGTGCACCTTTCTACAGCTATTGCGGCGAAYACYTCGAAGTGTCTGAAAATAGCAGCACARAACGTGTATCTGGAGGGGAACGGTGCATGGACCGGCGAGACAAGCGTCGAGATGCTGCTRGACATGGGGCTGAGCCATGTAATAATAGGACACTCTGAAAGACGTAGAATCATGGGCGAGACCAATGAGCAGAGTGCTAAGAAGGCGAAGCGTGCTCTRGAYAAAGGTATGACTGTTATCTTCTGCACCGGAGAGACCCTGGATGAACGCAAGGCCAATAACACTATGGAGGTGAATATTGCTCAGCTCGAGGCTCTTAAGAAGGAGATTGGAGAATCAAAGAAGTTATGGGAGAACGTTGTAATTGCCTATGAGCCGGTGTGGTCTATCGGCACGGG-

>Sweh151

-GGGTAACTTCAAATGCAATGGATCGCTCGACTTCATTAAGAGCCACGTAGCGTCCATCGCCTCCTATAAGATCCCCGAGTCCGTGGACGTTGTTGTTGCTCCCTCCTTTGTGCACCTTTCTACAGCTATTGCGGCGAAYACYTCGAAGTGTCTGAAAATAGCAGCACAGAACGTGTATCTGGAGGGGAACGGTGCATGGACCGGCGAGACAAGCGTCGAGATGCTGCTGGACATGGGGCTGAGCCATGTAATAATAGGACACTCTGAAAGACGTAGAATCATGGGCGAGACCAATGAGCAGAGTGCTAAGAAGGCGAAGCGTGCTCTGGACAAAGGTATGACTGTTATCTTCTGCACCGGAGAGACCCTGGATGAACGCAAGGCCAATAACACTATGGAGGTGAATATTGCTCAGMTCGAGGCTCTTAAGAAGGAGATTGGAGAATCAAAGAAGTTATGGGAGAACGTTGTAATTGCCTATGAGCCGGTGTGGTCTATCGGCACGGGC

>Sweh154

GGGGTAACTTCAAATGCAATGGATCGCTCGACTTCATTAAGAGCCACGTAGCGTCCATCGCCTCCTATAAGATCCCCGAGTCCGTGGACGTTGTTGTTGCTCCCTCCTTTGTGCACCTTTCTACAGCTATTGCGGCGAATACTTCGAAGTGTCTGAAAATAGCAGCACAGAACGTGTATCTGGAAGGGAACGGTGCATGGACCGGCGAGACAAGCGTCGAGATGCTGCTGGACATGGGGCTGAGCCATGTAATAATAGGACACTCTGAAAGACGTAGAATCATGGGCGAGACCAATGAGCAGAGTGCTAAAAAGGCGAAGCGTGCTCTGGACAAAGGTATGACTGTTATCTTCTGCACCGGAGAGACCCTGGATGAACGCAAGGCCAATAACACTATGGAGGTAAATATTGCTCAGCTCGAGGCTCTTAAGAAGGAGATTGGAGAATCAAAGAAGTTATGGGAGAACGTTGTAATTGCCTATGAGCCGGTGTGGTCTATCGGCACGGGC

>Sweh156_

GGGGTAACTTCAAATGCAATGGATCGCTCGACTTCATTAAGAGCCACGTAGCGTCCATCGCCTCCTATAAGATCCCCGAGTCCGTGGACGTTGTTGTTGCTCCCTCCTTTGTGCACCTTTCTACAGCTATTGCGGCGAATACTTCGAAGTGTCTGAAAATAGCAGCACAGAACGTGTATCTGGAAGGGAACGGTGCATGGACCGGCGAGACAAGCGTCGAGATGCTGCTGGACATGGGGCTGAGCCATGTAATAATAGGACACTCTGAAAGACGTAGAATCATGGGCGAGACCAATGAGCAGAGTGCTAAGAAGGCGAAGCGTGCTCTGGACAAAGGTATGACTGTTATCTTCTGCACCGGAGAGACCCTGGATGAACGCAAGGCCAATAACACTATGGAGGTAAATATTGCTCAGCTCGAGGCTCTTAAGAAGGAGATTGGAGAATCAAAGAAGTTATGGGAGAACGTTGTAATTGCCTATGAGCCGGTGTGGTCTATCGGCACGGGC

>Sweh158

GGGGTAACTTCAAATGCAATGGATCGCTCGACTTCATTAAGAGCCACGTAGCGTCCATCGCCTCCTATAAGATCCCCGAGTCCGTGGACGTTGTTGTTGCTCCCTCCTTTGTGCACCTTTCTACAGCTATTGCGGCGAATACTTCGAAGTGTCTGAAAATAGCAGCACAGAACGTGTATCTGGAAGGGAACGGTGCATGGACCGGCGAGACAAGCGTTGAGATGCTGCTGGACATGGGGCTGAGCCATGTAATAATAGGACACTCTGAAAGACGTAGAATCATGGGCGAGACCAATGAGCAGAGTGCTAAGAAGGCGAAGCGTGCTCTGGACAAAGGTATGACTGTTATCTTCTGCACCGGAGAGACTCTGGATGAACGCAAGGCCAATAACACTATGGAGGTGAATATTGCTCAGCTCGAGGCTCTTAAGAAGGAGATTGGAGAATCAAAGAAGTTATGGGAGAACGTTGTAATTGCCTATGAGCCGGTGTGGTCTATCGGCACGGGC

>Sweh159

GGGGTAACTTCAAATGCAATGGATCGCTCGACTTCATTAAGAGCCACGTAGCGTCCATCGCCTCCTATAAGATCCCCGAGTCCGTGGACGTTGTTGTTGCTCCCTCCTTTGTGCACCTTTCTACAGCTATTGCGGCGAATACTTCGAAGTGTCTGAAAATAGCAGCACAGAACGTGTATCTGGAAGGGAACGGTGCATGGACCGGCGAGACAAGCGTCGAGATGCTGCTGGACATGGGGCTGAGCCATGTAATAATAGGACACTCTGAAAGACGTAGAATCATGGGCGAGACCAATGAGCAGAGTGCTAAGAAGGCGAAGCGTGCTCTGGACAAAGGTATGACTGTTATCTTCTGCACCGGAGAGACCCTGGATGAACGCAAGGCCAATAACACTATGGAGGTAAATATTGCTCAGCTCGAGGCTCTTAAGAAGGAGATTGGAGAATCAAAGAAGTTATGGGAGAACGTTGTAATTGCCTATGAGCCGGTGTGGTCTATCGGCACGGGC

>Sweh160

GGGGTAACTTCAAATGCAATGGATCGCTCGACTTCATTAAGAGCCACGTAGCGTCCATCGCCTCCTATAAGATCCCCGAGTCCGTGGACGTTGTTGTTGCTCCCTCCTTTGTGCACCTTTCTACAGCTATTGCGGCGAATACTTCGAAGTGTCTGAAAATAGCAGCACAGAACGTGTATCTGGAAGGGAACGGTGCATGGACCGGCGAGACAAGCGTCGAGATGCTGCTGGACATGGGGCTGAGCCATGTAATAATAGGACACTCTGAAAGACGTAGAATCATGGGCGAGACCAATGAGCAGAGTGCTAAGAAGGCGAAGCGTGCTCTGGACAAAGGTATGACTGTTATCTTCTGCACCGGAGAGACCCTGGATGAACGCAAGGCCAATAACACTATGGAGGTAAATATTGCTCAGCTCGAGGCTCTTAAGAAGGAGATTGGAGAATCAAAGAAGTTATGGGAGAACGTTGTAATTGCCTATGAGCCGGTGTGGTCTATCGGCACGGGC

>Sweh161

GGGGTAACTTCAARTGCAATGGATCGCTCGACTTCATTAAGAGCCACGTAGCGTCCATCGCCTCCYATAAGATCCCCGAGTCCGTGGACGTTGTTRTTGCTCCCTCCTTTGTGCACCTTTCTACAGCTATTGCGGCrAACACYTCGAAGTGTCTGAAAATAGCAGCACAGAACGTGTATCTGGARGGGAACGGTGCATGGACCGGCGAGACAAGCGTCGAGATGCTGCTGGACATGGGGCTGAGCCATGTAATAATAGGACACTCTGAAAGACGTAGAATCATGGGCGAGACCAATGAGCAGAGTGCTAAGAAGGCGAAGCGTGCTCTGGACAAAGGTATGACTGTTATCTTCTGCACCGGAGAGACCCTGGATGAACGCAAGGCCAATAACACTATGGAGGTGAATATTGYTCAGCTCGAGGCTCTTAAGAAGGAGATTGGAGAATCAAAGAAGTTATGGGAGAACGTTGTAATTGCCTATGAGCCGGTGTGGTCTATCGGCACGGG

>Sweh163

-GGGTAACTTCAARTGCAATGGATCGCTCGACTTCATTAAGAGCCACGTAGCGTCCATCGCCTCCYATAAGATCCCCGAGTCCGTGGACGTTGTTGTTGCTCCCTCCTTTGTGCACCTTTCTACAGCTATTGCGGCGAAYACYTCGAAGTGTCTGAAAATAGCAGCACAGAAYGTGTATCTGGAAGGGAACGGTGCATGGACCGGCGAGACAAGCGTCGAGATGCTGYTGGACATGGGGCTGAGCCATGTAATAATAGGACACTCTGAAAGACGTAGAATCATGGGCGAGACCAATGAGCAGAGTGCTAAGAAGGCGAAGCGTGCTCTRGACAAAGGTATGACTGTTATCTTCTGCACCGGAGAGACCCTGGATGAACGCAAGGCCAATAACACTATGGAGGTGAATATTGCTCAGCTCGAGGCTCTTAAGAAGGAGATTGGAGAMTCAAAGAAGTTRTGGGAGRACRTTGTAATTGCCTATGAGCCGGTGTGGTCTATCGGCACGG--

>Sweh167

GGGGTAACTTCAAATGCAATGGATCGCTCGACTTCATTAAGAGCCACGTAGCGTCCATCGCCTCCTATAAGATCCCCGAGTCCGTGGACGTTGTTGTTGCTCCCTCCTTTGTGCACCTTTCTACAGCTATTGCGGCGAATACTTCGAAGTGTCTGAAAATAGCAGCACAGAACGTGTATCTGGAAGGGAACGGTGCATGGACCGGCGAGACAAGCGTCGAGATGCTGCTGGACATGGGGCTGAGCCATGTAATAATAGGACACTCTGAAAGACGTAGAATCATGGGCGAGACCAATGAGCAGAGTGCTAAGAAGGCGAAGCGTGCTCTGGACAAAGGTATGACTGTTATCTTCTGCACCGGAGAGACCCTGGATGAACGCAAGGCCAATAACACTATGGAGGTAAATATTGCTCAGCTCGAGGCTCTTAAGAAGGAGATTGGAGAATCAAAGAAGTTATGGGAGAACGTTGTAATTGCCTATGAGCCGGTGTGGTCTATCGGCACGGGC

>Sweh168

---------TCAAATGCAATGGATCGCTCGACTTCATTAAGAGCCACGTAGCGTCCATCGCCTCCTATAAGATCCCCGAGTCCGTGGACGTTGTTGTTGCTCCCTCCTTTGTGCACCTTTCTACAGCTATTGCGGCGAATACTTCGAAGTGTCTGAAAATAGCAGCACAGAACGTGTATCTGGAAGGGAACGGTGCATGGACCGGCGAGACAAGCGTCGAGATGCTGCTGGACATGGGGCTGAGCCATGTAATAATAGGACACTCTGAAAGACGTAGAATCATGGGCGAGACCAATGAGCAGAGTGCTAAGAAGGCGAAGCGTGCTCTGGACAAAGGTATGACTGTTATCTTCTGCACCGGAGAGACCCTGGATGAACGCAAGGCCAATAACACTATGGAGGTAAATATTGCTCAGCTCGAGGCTCTTAAGAAGGAGATTGGAGAATCAAAGAAGTTATGGGAGAACGTTGTAATTGCCTATGAGCCGGTGTGGTCTATCGGCACG---

>Sweh169

~~GGTAACTTCAAATGCAATGGATCGCTCGACTTCATTAAGAGCCACGTAGCGTCCATCGCCTCCTATAAGATCYCCGAGTCCGTGGACGTTGTTGTTGCTCCCTCCTTTGTGCACCTTTCTACAGCTATTGCGGCGAATACYTCGAAGTGTCTGAAAATAGCAGCACAGAACGTGTATCTGGAGGGGAACGGTGCATGGACCGGCGAGACAAGCGTCGAGATGCTGCTGGACATGGGGCTGAGCCATGTAATAATAGGACACTCTGAAAGACGTAGAATCATGGGCGAGACCAATGAGCAGAGTGCTAAGAAGGCGAAGCGTGCTCTGGACAAAGGTATGACTGTTATCTTCTGCACCGGAGAGACCYTGGATGAACGCAAGGCCAATAACACTATGGAGGTGAATATTGCTCAGCTCGAGGCTCTTAAGAAGGAGATTGGAGAATCAAAGAAGTTATGGGAGAACGTTGTAATTGCCTATGAGCCGGTGTGGTCTATCGGCACGGG

>Sweh170

--------TTCAAATGCAATGGATCGCTCGACTTCATTAAGAGCCACGTAGCGTCCATCGCCTCCTATAAGATCCCCGAGTCCGTGGACGTTGTTGTTGCTCCCTCCTTTGTGCACCTTTCTACAGCTATTGCGGCGAAYACYTCGAAGTGTCTGAAAATAGCAGCACAGAAYGTGTATCTGGAGGGGAACGGTGCATGGACCGGCGAGACAAGCGTCGARATGCTGCTGGACATGGGGCTGAGCCATGTAATAATAGGACACTCTGAAAGACGTAGAATCATGGGCGAGACCAATGAGCAGAGTGCTAAGAAGGCGAAGCRTGCTCTGGACAAAGGTATGACTGTTATCTTCTGCACCGGAGAGACCCTGGATGAACGCAAGGCCAATAACACTATGGAGGTGAATATTGCTCAGCTCGAGGCTCTTAAGAAGGAGATTGGAGAMTCAAAGAAGTTATGGGAGAACGTTGTAATTGCCTATGAGCCGGTGTGGTCTATCGGCACGG--

>Sweh171

-GGGTAACTTCAAGTGCAATGGATCGCTCGACTTCATTAAGAGCCACGTAGCGTCCATCGCCTCCCATAAGATCCCCGAGTCCGTGGACGTTGTTGTTGCTCCCTCCTTTGTGCACCTTTCTACAGCTATTGCGGCGAATACCTCGAAGTGTCTGAAAATAGCAGCACAGAATGTGTATCTGGAGGGGAACGGTGCATGGACCGGCGAGACAAGCGTCGAGATGCTGCTGGACATGGGGCTGAGCCATGTAATAATAGGACACTCTGAAAGACGTAGAATCATGGGCGAGACCAATGAGCAGAGTGCTAAGAAGGCGAAGCGTGCTCTGGACAAAGGTATGACTGTTATCTTCTGCACCGGAGAGACCCTGGATGAACGCAAGGCCAATAACACTATGGAGGTGAATATTGCTCAGCTCGAGGCTCTTAAGAAGGAGATTGGAGAATCAAAGAAGTTATGGGAGAACGTTGTAATTGCCTATGAGCCGGTGTGGTCTATCGGCACGGGC

>Sweh179

GGGGTAACTTCAAATGCAATGGATCGCTCGACTTCATTAAGAGCCACGTAGCGTCCATCGCCTCCTATAAGATCCCCGAGTCCGTGGACGTTGTTGTTGCTCCCTCCTTTGTGCACCTTTCTACAGCTATTGCGGCGAATACTTCGAAGTGTCTGAAAATAGCAGCACAGAACGTGTATCTGGAAGGGAACGGTGCATGGACCGGCGAGACAAGCGTCGAGATGCTGCTGGACATGGGGCTGAGCCATGTAATAATAGGACACTCTGAAAGACGTAGAATCATGGGCGAGACCAATGAGCAGAGTGCTAAGAAGGCGAAGCGTGCTCTGGACAAAGGTATGACTGTTATCTTCTGCACCGGAGAGACCCTGGATGAACGCAAGGCCAATAACACTATGGAGGTGAATATTGCTCAGCTCGAGGCTCTTAAGAAGGAGATTGGAGAATCAAAGAAGTTATGGGAGAACGTTGTAATTGCCTATGAGCCGGTGTGGTCTATCGGCACGGGC

>Sweh184

----TAACTTCAAATGCAATGGATCRCTCGACTTCATTAAGAGCCACGTAGCGTCCATCGCCTCCYATAAGATCCCCGAGTCCGTGGACGTTGTTGTTGCTCCCTCCTTTGTGCACCTTTCTACAGCTATTGCGGCRAAYACYTCGAAGTGTCTGAAAATAGCAGCACAGAAYGTGTATCTGGARGGGAACGGTGCATGGACCGGCGAGACAAGCGTCGARATGCTGCTGGACATGGGGCTGAGCCATGTAATAATAGGACACTCTGAAAGACGTAGAATCATGGGCGAGACCAATGAGCAGAGTGCTAAGAAGGCGAAGCGTGCTCTGGACAAAGGTATGACTGTTATCTTCTGCACCGGAGAGACCCTGGATGAACGCAAGGCCAATAACACTATGGAGGTGAATATTGCTCAGCTCGAGGCTCTTAAGAAGGAGATTGGAGAATCAAAGAAGTTATGGGAGAACGTTGTAATTGCCTATGAGCCGGTGTGGTCTATCGGCA-----

>Sweh186

-GGGTAACTTCAARTGCAATGGATCGCTCRACTTCATTAAGAGCCACGTAGCGTCCATCGCCTCCYATAAGATCCCCGAGTCCGTGGACGTTGTTGTTGCTCCCTCCTTTGTGCACCTTTCTACAGCTATTGCGGCRAAYACCTCGAAGTGTCTGAAAATAGCAGCACAGAAYGTGTATCTGGARGGGAACGGTGCATGGACCGGCGAGACAAGCGTCGAGATGCTGCTGGACATGGGGCTGAGCCATGTAATARTAGGACACTCTGAAAGACGTAGAATCATGGGCGAGACCAATGAGCAGAGTGCYAAGAAGGCGAAGCGTGCTCTGGACAAAGGTATGACTGTTATCTTCTGCACCGGAGAGACCCTGGATGAACGCAAGGCCAATAACACTATGGAGGTGAATATTGCTCAGCTCGAGGCTCTTAAGAAGGAGATTGGAGAATCAAAGAAGTTATGGGAGAACGTTGTAATTGCCTATGAGCCGGTGTGGTCTATCGGCACG---

>Sweh188

GGGGTAACTTCAAATGCAATGGATCGCTCRACTTCATTAAGRGCCACGTAGCGTCCATCGCCTCCYATAAGATCCCCGAGTCCGTGGACGTTGTTGTTGCTCCCTCCTTTGTGCACCTTTCTACAGCTATTGCGGCGAAYACCTCGAAGTGTCTGAAAATAGCAGCACAGAAYGTGTATCTGGAGGGGAACGGTGCATGGACCGGCGAGACAAGCGTCGAGATGCTGCTGGACATGGGGCTGAGCCATGTAATARTAGGACACTCTGAAAGACGTAGAATCATGGGCGAGACCAATGAGCAGAGTGCTAAGAAGGCGAAGCGTGCTCTGGACAAAGGTATGACTGTTATCTTCTGCACCGGAGAGACCCTGGATGAACGCAAGGCCAATAACACTATGGAGGTGAATATTGCTCAGCTCGAGGCTCTTAAGAAGGAGATTGGAGAATCAAAGAAGTTATGGGAGAACRTTGTAATTGCCTATGAGCCGGTGTGGTCTATCGGCACGGG

>Sweh189

GGGGTAACTTCAAGTGCAATGGATCGCTCGACTTCATTAAGAGCCACGTAGCGTCCATCGCCTCCCATAAGATCCCCGAGTCCGTGGACGTTGTTGTTGCTCCCTCCTTTGTGCACCTTTCTACAGCTATTGCGGCGAACACCTCGAAGTGTCTGAAAATAGCAGCACAGAACGTGTATCTGGAGGGGAACGGTGCATGGACCGGCGAGACAAGCGTCGAGATGCTGCTGGACATGGGGCTGAGCCATGTAATAATAGGACACTCTGAAAGACGTAGAATCATGGGCGAGACCAATGAGCAGAGTGCTAAGAAGGCGAAGCGTGCTCTGGACAAAGGTATGACTGTTATCTTCTGCACCGGAGAGACCCTGGATGAACGCAAGGCCAATAACACTATGGAGGTGAATATTGCTCAGCTCGAGGCTCTTAAGAAGGAGATTGGAGAATCAAAGAAGTTATGGGAGAACGTTGTAATTGCCTATGAGCCGGTGTGGTCTATCGGCACGGGC

>Sweh191

GGGGTAACTTCAARTGCAATGGATCGCTCGACTTCATTAAGAGCCACGTAGCGTCCATCGCCTCCYATAAGATCCCCGAGTCCGTGGACGTTGTTGTTGCTCCCTCCTTTGTGCACCTTTCTACAGCTATTGCGGCGAAYACYTCGAAGTGTCTGAAAATAGCAGCACAGAACGTGTATCTGGARGGGAACGGTGCATGGACCGGCGAGACAAGCGTCGAGATGCTGCTGGACATGGGGCTGAGCCATGTAATAATAGGACACTCTGAAAGACGTAGAATCATGGGYGAGACCAATGAGCAGAGTGCTAAGAAGGCGAAGCGTGCTCTGGACAAAGGTATGACTGTTATCTTCTGCACCGGAGAGACCCTGGATGAACGCAAGGCCAATAACACTATGGAGGTRAATATTGCTCAGCTCGAGGCTCTTAAGAAGGAGATTGGAGAATCAAAGAAGTTATGGGAGAACGTTGTAATTGCCTATGAGCCGGTGTGGTCTATCGGCACGGGC

>Sweh192

GGGGTAACTTCAAATGCAATGGATCGCTCGACTTCATTAAGAGCCACGTAGCGTCCATCGCCTCCTATAAGATCCCCGAGTCCGTGGACGTTGTTGTTGCTCCCTCCTTTGTGCACCTTTCTACAGCTATTGCGGCGAATACTTCGAAGTGTCTGAAAATAGCAGCACAGAACGTGTATCTGGAAGGGAACGGTGCATGGACCGGCGAGACAAGCGTCGAGATGCTGCTGGACATGGGGCTGAGCCATGTAATAATAGGACACTCTGAAAGACGTAGAATCATGGGCGAGACCAATGAGCAGAGTGCTAAGAAGGCGAAGCGTGCTCTGGACAAAGGTATGACTGTTATCTTCTGCACCGGAGAGACCCTGGATGAACGCAAGGCCAATAACACTATGGAGGTAAATATTGCTCAGCTCGAGGCTCTTAAGAAGGAGATTGGAGAATCAAAGAAGTTATGGGAGAACGTtGTAATtGCC

>Sweh193

GGGGTAACTTCAARTGCAATGGATCGCTCGACTTCATTAAGAGCCACGTAGCGTCCATCGCCTCCCATAAGATCYCCGAGTCCGTGGACGTTGTTGTTGCTCCCTCCTTTGTGCACCTTTCTACAGCTATTGCGGCRAACACCTCGAAGTGTCTGAAAATAGCAGCACAGAACGTGTATCTGGAGGGGAACGGTGCATGGACCGGCGAGACAAGCGTCGAGATGCTGCTGGACATGGGGCTGAGCCATGTAATAATAGGACACTCTGAAAGACGTAGAATCATGGGCGAGACCAATGAGCAGAGTGCTAAGAAGGCGAAGCGTGCTCTGGACAAAGGTATGACTGTTATCTTCTGCACCGGAGAGACCCTGGATGARCGCAAGGCCAATAACACTATGGAGGTGAATATTGCYCAGCTCGAGGCTCTTAAGAAGGAGATTGGAGAATCAAAGAAGTTRTGGGAGAACGTTGTAATTGCCTATGAGCCGGTGTGGTCTATCGGCACGGGC

>Sweh195

GGGGTAACTTCAARTGCAATGGATCGCTCGACTTCATTARGAGCCACGTAGCGTCCATCGYCTCCCATAAGATCCCCGAGTCCGTGGACGTTGTTGTTGCTCCCTCCTTTGTGCACCTTTCTACAGCTATTGCGGCGAAYACCTCGAAGTGTCTGAAAATAGCAGCACAGAACGTGTATCTGGAGGGGAACGGTGCATGGACCGGCGAGACAAGCGTCGAGATGCTGCTGGACATGGGGCTGAGCCATGTAATAATAGGACACTCTGAAAGACGTAGAATCATGGGCGAGACCAATGAGCAGAGTGCTAAGAAGGCGAAGCGTGCTCTGGACAAAGGTATGACTGTTATCTTCTGCACCGGAGAGACCCTGGATGAACGCAAGGCCAATAACACYATGGAGGTGAATATTGCTCAGCTCGAGGCTCTTAAGAAGGAGATTGGAGAATCAAAGAAGTTATGGGAGAACGTTGTAATTGCCTATGAGCCGGTGTGGTCTATCGGCACGGGC

>Sweh196

GGGGTAACTTCAAGTGCAATGGATCGCTCGACTTCATTAAGAGCCACGTAGCGTCCATCGCCTCCCATAAGATCCCCGAGTCCGTGGACGTTGTTGTTGCTCCCTCCTTTGTGCACCTTTCTACAGCTATTGCGGCGAAYACTTCGAAGTGTCTGAAAATAGCAGCACAGAACGTGTATCTGGAAGGGAACGGTGCATGGACCGGCGAGACAAGCGTCGAGATGCTGCTGGACATGGGGCTGAGCCATGTAATAATAGGACACTCTGAAAGACGTAGAATCATGGGCGAGACCAATGAGCAGAGTGCTAAGAAGGCGAAGCGTGCTCTGGACAAAGGTATGACTGTTATCTTCTGCACCGGAGAGACCCTGGATGAACGCAAGGCCAATAACACTATGGAGGTAAATATTGCTCAGCTCGAGGCTCTTAAGAAGGAGATTGGAGAATCAAAGAAGTTATGGGAGAACGTTGTAATTGCCTATGAGCCGGTGTGGTCTATCGGCA-----

>Sweh197

GGGGTAACTTCAARTGCAATGGATCGCTCGACTTCATTAAGAGCCACGTAGCGTCCATCGCCTCCCATAAGATCCCCGAGTCCGTGGAYGTTGTTGTTGCTCCCTCCTTTGTGCACCTTTCTACAGCTATTGCGGCGAAYACCTCGAAGTGTCTGAAAATAGCAGCACAGAACGTGTATCTGGAGGGGAACGGTGCATGGACCGGCGAGACAAGCGTCGAGATGCTGCTGGACATGGGGCTGAGCCATGTAATARTAGGACACTCTGAAAGACGTAGAATCATGGGCGAGACCAATGAGCAGAGTGCTAAGAAGGCGAAGCGTGCTCTGGACAAAGGTATGACTGTTATCTTCTGCACCGGAGAGACCCTGGATGAACGCAAGGCCAATAACACTATGGAGGTGAATATTGCTCAGCTCGAGGCTCTTAAGAAGGAGATTGGAGAATCAAAGAAGTTATGGGAGAACGTTGTAATTGCCTATGAGCCGGTGTGGTCTATCGGCACGGGC

>Sweh198

GGGGTAACTTCAAGTGCAATGGATCGCTCGACTTCATTAAGAGCCACGTAGCGTCCATCGCCTCCCATAAGATCCCCGAGTCCGTGGACGTTGTTGTTGCTCCCTCCTTTGTGCACCTTTCTACAGCTATTGCGGCGAACACCTCGAAGTGTCTGAAAATAGCAGCACAGAACGTGTATCTGGAGGGGAACGGTGCATGGACCGGCGAGACAAGCGTCGAGATGCTGCTGGACATGGGGCTGAGCCATGTAATAATAGGACACTCTGAAAGACGTAGAATCATGGGCGAGACCAATGAGCAGAGTGCTAAGAAGGCGAAGCGTGCTCTGGACAAAGGTATGACTGTTATCTTCTGCACCGGAGAGACCCTGGATGAACGCAAGGCCAATAACACTATGGAGGTGAATATTGCTCAGCTCGAGGCTCTTAAGAAGGAGATTGGAGAATCAAAGAAGTTATGGGAGAACGTTGTAATTGCCTATGAGCCGGTGTGGTCTATCGGCACGGGC

>Sweh199

GGGGTAACTTCAAATGCAATGGATCGCTCGACTTCATTAAGAGCCACGTAGCGTCCATCGCCTCCTATAAGATCCCCGAGTCCGTGGACGTTGTTGTTGCTCCCTCCTTTGTGCACCTTTCTACAGCTATTGCGGCGAATACTTCGAAGTGTCTGAAAATAGCAGCACAGAACGTGTATCTGGAAGGGAACGGTGCATGGACCGGCGAGACAAGCGTTGAGATGCTGCTGGACATGGGGCTGAGCCATGTAATAATAGGACACTCTGAAAGACGTAGAATCATGGGCGAGACCAATGAGCAGAGTGCTAAGAAGGCGAAGCGTGCTCTGGACAAAGGTATGACTGTTATCTTCTGCACCGGAGAGACTCTGGATGAACGCAAGGCCAATAACACTATGGAGGTGAATATTGCTCAGCTCGAGGCTCTTAAGAAGGAGATTGGAGAATCAAAGAAGTTATGGGAGAACGTTGTAATTGCCTATGAGCCGGTGTGGTCTATCGGCACGGGC

>Sweh200

GGGGTAACTTCAAATGCAATGGATCGCTCGACTTCATTAAGAGCCACGTAGCGTCCATCGCCTCCTATAAGATCCCCGAGTCCGTGGACGTTGTTGTTGCTCCCTCCTTTGTGCACCTTTCTACAGCTATTGCGGCGAATACTTCGAAGTGTCTGAAAATAGCAGCACAGAACGTGTATCTGGAAGGGAACGGTGCATGGACCGGCGAGACAAGCGTTGAGATGCTGCTGGACATGGGGCTGAGCCATGTAATAATAGGACACTCTGAAAGACGTAGAATCATGGGCGAGACCAATGAGCAGAGTGCTAAGAAGGCGAAGCGTGCTCTGGACAAAGGTATGACTGTTATCTTCTGCACCGGAGAGACTCTGGATGAACGCAAGGCCAATAACACTATGGAGGTGAATATTGCTCAGCTCGAGGCTCTTAAGAAGGAGATTGGAGAATCAAAGAAGTTATGGGAGAACGTTGTAATTGCCTATGAGCCGGTGTGGTCTATCGGCACGGGC

>Sweh202

GGGGTAACTTCAAATGCAATGGATCGCTCGACTTCaTTAAGAGCCACGTAGCGTCCATCGCCTCCTATAAGATCCCCGAGTCCGTGGACGTTGTTGTTGCTCCCTCCTTTGTGCACCTTTCTACAGCTATTGCGGCGAATACTTCGAAGTGTCTGAAAATAGCAGCACAGAACGTGTATCTGGAAGGGAACGGTGCATGGACCGGCGAGACAAGCGTTGAGATGCTGCTGGACATGGGGCTGAGCCATGTAATAATAGGACACTCTGAAAGACGTAGAATCATGGGCGAGACCAATGAGCAGAGTGCTAAGAAGGCGAAGCGTGCTCTGGACAAAGGTATGACTGTTATCTTCTGCACCGGAGAGACTCTGGATGAACGCAAGGCCAATAACACTATGGAGGTGAATATTGCTCAGCTCGAGGCTCTTAAGAAGGAGATTGGAGAATCAAAGAAGTTATGGGAGAACGTTGTAATTGCCTATGAGCCGGTGTGGTCTATCGGCACGGGC

>Sweh203

------ACTTCAARTGCAATGGATCGCTCGACTTCATTAAGRGCCACGTAGCGTCCATCGCCTCCCATAAGATCCCCGAGTCCGTGGACGTTGTTGTTGCTCCCTCCTTTGTGCAYCTTTCTACAGCTATTGCGGCGAAYACYTCGAAGTGTCTGAAAATAGCAGCACAGAACGTGTATCTGGAGGGGAACGGTGCATGGACCGGCGAGACAAGCGTCGAGATGCTGCTRGACATGGGGCTGAGCCATGTAATAATAGGACACTCTGAAAGACGTAGAATCATGGGCGAGACCAATGAGCAGAGTGCTAAGAAGGCGAAGCGTGCTCTGGACAAAGGTATGACTGTTATCTTCTGCACCGGAGAGACCCTGGATGAACGCAAGGCCAATAACACTATGGAGGTGAATATTGCTCAGCTCGAGGCTCTTAAGAAGGAGATTGGAGAATCAAAGAAGTTATGGGAGAACGTTGTAATTGCYTATGAGCCGGTGTGGTCTATCGGCACGGGC

>Sweh206

GGGGTAACTTCAARTGCAATGGATCGCTCGACTTCATTAAGAGCCACGTAGCGTCCATCGCCTCCYATAAGATCCCCGAGTCCGTGGACGTTGTTRTTGCTCCCTCCTTTGTGCACCTTTCTACAGCTATTGCGGCrAAYACYTCGAAGTGTCTGAAAATAGCAGCACAGAACGTGTATCTGGARGGGAACGGTGCATGGACCGGCGAGACAAGCGTCGAGATGCTGCTGGACATGGGGCTGAGCCATGTAATAATAGGACACTCTGAAAGACGTAGAATCATGGGCGAGACCAATGAGCAGAGTGCTAAGAAGGCGAAGCGTGCTCTGGACAAAGGTATGACTGTTATCTTCTGCACCGGAGAGACCCTGGATGAACGCAAGGCCAATAACACTATGGAGGTGAATATTGYTCAGCTCGAGGCTCTTAAGAAGGAGATTGGAGAATCAAAGAAGTTATGGGAGAACGTTGTRATTGCCTATGAGCCGGTGTGGTCTATCGGCACGGGC

>Sweh208

GGGGTAACTTCAAGTGCAACGGATCGCTCGACTTCATTAAGAGCCACGTAGCGTCCATCGCCTCCYATRAGATCCCCGAGTCCGTGGACGTTGTTGTTGCTCCCTCCTTTGTGCACCTTTCTACAGCTATTGCGGCGAACACCTCGAAGTGTCTGAAAATAGCAGCACAGAACGTGTATCTGGAGGGGAACGGTGCATGGACCGGCGAGACAAGCGTCGAGATGCTGCTGGACATGGGGCTGAGCCATGTAATAATAGGACACTCTGAAAGACGTAGAATCATGGGCGAGACCAATGAGCAGAGTGCTAAGAAGGCGAAGCGTGCTCTGGACAAAGGTATGACTGTTATCTTCTGCACCGGAGAGACCCTGGATGAACGCAAGGCCAATAACACTATGGAGGTGAATATTGCTCAGCTCGAGGCTCTTAAGAAGGAGATTGGAGAATCAAAGAAGTTATGGGAGAACGTTGTAATTGCCTATGAGCCGGTGTGGTCTATCGGCACGGGC

>Sweh212

~~~~TAACTTCAAATGCAATGGATCGCTCGACTTCATTAAGAGCCACGTAGCGTCCATCGCCTCCTATAAGATCCCCGAGTCCGTGGACGTTGTTGTTGCTCCCTCCTTTGTGCACCTTTCTACAGCTATTGCGGCGAATACTTCGAAGTGTCTGAAAATAGCAGCACAGAACGTGTATCTGGAAGGGAACGGTGCATGGACCGGCGAGACAAGCGTTGAGATGCTGCTGGACATGGGGCTGAGCCATGTAATAATAGGACACTCTGAAAGACGTAGAATCATGGGCGAGACCAATGAGCAGAGTGCTAAGAAGGCGAAGCGTGCTCTGGACAAAGGTATGACTGTTATCTTCTGCACCGGAGAGACTCTGGATGAACGCAAGGCCAATAACACTATGGAGGTGAATATTGCTCAGCTCGAGGCTCTTAAGAAGGAGATTGGAGAATCAAAGAAGTTATGGGAGAACGTtGTAATTGCCTATGAGCCGGTGTGGTCTATCGGCACGGGC

>Sweh213

GGGGTAACTTCAAATGCAATGGATCGCTCGACTTCATTAAGAGCCACGTAGCGTCCATCGCCTCCTATAAGATCCCCGAGTCCGTGGACGTTGTTGTTGCTCCCTCCTTTGTGCACCTTTCTACAGCTATTGCGGCGAATACTTCGAAGTGTCTGAAAATAGCAGCACAGAACGTGTATCTGGAAGGGAACGGTGCATGGACCGGCGAGACAAGCGTCGAGATGCTGCTGGACATGGGGCTGAGCCATGTAATAATAGGACACTCTGAAAGACGTAGAATCATGGGCGAGACCAATGAGCAGAGTGCTAAGAAGGCGAAGCGTGCTCTGGACAAAGGTATGACTGTTATCTTCTGCACCGGAGAGACCCTGGATGAACGCAAGGCCAATAACACTATGGAGGTAAATATTGCTCAGCTCGAGGCTCTTAAGAAGGAGATTGGAGAATCAAAGAAGTTATGGGAGAACGTTGTAATTGCCTATGAGCCGGTGTGGTCTATCGGCACGGGC

>Sweh217

GGGGTAACTTCAAATGCAATGGATCGCTCGACTTCATTAAGAGCCACGTAGCGTCCATCGCCTCCTATAAGATCCCCGAGTCCGTGGACGTTGTTGTTGCTCCCTCCTTTGTGCACCTTTCTACAGCTATTGCGGCGAATACTTCGAAGTGTCTGAAAATAGCAGCACAGAACGTGTATCTGGAAGGGAACGGTGCATGGACCGGCGAGACAAGCGTCGAGATGCTGCTGGACATGGGGCTGAGCCATGTAATAATAGGACACTCTGAAAGACGTAGAATCATGGGCGAGACCAATGAGCAGAGTGCTAAGAAGGCGAAGCGTGCTCTGGACAAAGGTATGACTGTTATCTTCTGCACCGGAGAGACCCTGGATGAACGCAAGGCCAATAACACTATGGAGGTAAATATTGCTCAGCTCGAGGCTCTTAAGAAGGAGATTGGAGAATCAAAGAAGTTATGGGAGAACGTTGTAATTGCCTATGAGCCGGTGTGGTCTATCGGCACGGGC
