# Supplementary material for: Multilocus Genotyping of Human Giardia Isolates Suggests Limited Zoonotic Transmission and Association between Assemblage B and Flatulence in Children
Source: PLoS Negl Trop Dis. 2011 Aug 2;5(8):e1262. doi: 10.1371/journal.pntd.0001262 (PMC3149019; doi:10.1371/journal.pntd.0001262)
Supplement: Table S1 — (DOC) [file pntd.0001262.s005.doc]

**Supplementary Table S1. GenBank accession numbers of reference sequences. Isolate names in brackets.**

| **Sub-assemblage** | **β-giardin** | ***gdh*** | ***tpi*** |
| --- | --- | --- | --- |
| AI | EU014394 (WB) | AY178735 (Ad-1) | L02120 (WB) |
| AII | Subtype A2: AY072723 (KC8)  Subtype A3: AY072724 (ISSGF7) | EF685688 (JH) | U57897 (JH) |
| AIII | EU216429 (JT001) | DQ100288 (NLR118) | DQ650648 (isolate name not stated) |
| BIII | AY072726 (LD18)  AY072727 (BAH8) | AF069059 (BAH-12)  AB195224 (GH135) | AF069561 (BAH12)  AB516352 (GH135) |
| BIV | AY072725 (Nij5) | L40508 (Ad-7)  AY178750 (Vanc89/UBC/059) | L02116 (GS/M)  AF069560 (AD-19) |
|  | Human isolates | | |
| A | GQ329671 (Sweh166) | GQ329674 (Sweh166) | GQ329677 (Sweh166) |
| A | GQ329672 (Sweh173) | GQ329675 (Sweh173) | GQ329678 (Sweh173) |
|  | Animal isolates | | |
| A | JF773749 (Swefd165) | JF773754 (Swefd165) | JF773758 (Swefd165) |
| A | JF773747 (Swesheep015) | JF773750 (Swesheep015) | JF773756 (Swesheep015) |
| A | JF773748 (Swesheep016) | JF773751 (Swesheep016) | JF773757 (Swesheep016) |
| A | EU769204 (Swecat035) | JF773752 (Swecat035) | EU781000 (Swecat035) |
| A | EU769205 (Swecat078) | JF773753 (Swecat078) | EU781001 (Swecat078) |
| AIII | EU769206 (Swecat171) | EU769223 (Swecat171) | EU781002 (Swecat171) |
|  |  |  |  |
| B | EU769211 (Swemon200) | EU769226 (Swemon200) | EU781015 (Swemon200) |
| B | EU769209 (Swegp138) | EU769225 (Swegp138) | EU781014 (Swegp138) |
| B | EU769210 (Swerab176) | JF773755 (Swerab176) | JF773759 (Swerab176) |
