# Supplementary material for: Multilocus Genotyping of Human Giardia Isolates Suggests Limited Zoonotic Transmission and Association between Assemblage B and Flatulence in Children
Source: PLoS Negl Trop Dis. 2011 Aug 2;5(8):e1262. doi: 10.1371/journal.pntd.0001262 (PMC3149019; doi:10.1371/journal.pntd.0001262)
Supplement: Table S2 — (DOC) [file pntd.0001262.s006.doc]

**Supplementary table S2A**. Intra-genotypic nucleotide substitutions at the β-giardin locus for 120 assemblage B isolates from the present study*.

| Sub-assemblage | Isolate | GenBank acc. no. | Nucleotide position from start of gene | | | | | | | | | | | | |
| --- | --- | --- | --- | --- | --- | --- | --- | --- | --- | --- | --- | --- | --- | --- | --- |
|  |  |  | 204 | 210 | 228 | 273 | 285 | **354** | 369 | 438 | 477 | 516 | 564 | 588 | 609 |
| BIII | BAH8 | AY072727 | G | C | A | A | T | **C** | C | C | A | T | T | C | C |
| BIII | LD18 | AY072726 | G | C | A | G | T | **C** | C | C | A | T | T | C | C |
| BIV | Nij5 | AY072725 | G | T | A | A | T | **T** | C | T | A | T | T | C | C |
| BIV | ISSG4 | AY072728 | G | C | G | A | T | **T** | C | C | A | T | C | C | T |
|  | | |  |  |  |  |  |  |  |  |  |  |  |  |  |
| Nucleotides | | |  |  |  |  |  |  |  |  |  |  |  |  |  |
| C |  |  |  | 53 |  |  | 1 | **29** | 96 | 87 |  | 22 | 6 | 115 | 106 |
| T |  |  |  | 42 |  |  | 112 | **55** | 22 | 33 |  | 97 | 101 | 1 | 6 |
| Y (C/T) |  |  |  | 25 |  |  | 7 | **36** | 2 | 0 |  | 1 | 13 | 4 | 8 |
| A |  |  | 17 |  | 91 | 110 |  | **0** |  |  | 115 |  |  |  |  |
| G |  |  | 102 |  | 14 |  |  | **0** |  |  |  |  |  |  |  |
| R (A/G) |  |  | 1 |  | 15 | 10 |  | **0** |  |  | 5 |  |  |  |  |

*The figures given indicate the number of isolates with C, T, Y, A, G, or R at a particular nucleotide position, including only those positions where five or more isolates had nucleotide substitutions. Nucleotide positions highlighted in bold are the substitution patterns proposed as markers for different B sub-assemblages in Table 4 (Wielinga and Thompson, 2007).

**Supplementary table S2B.** Intra-genotypic nucleotide substitutions at the tpi locus for 120 assemblage B isolates from the present study*.

| Sub-assemblages | Isolate | GenBank acc. no. | Nucleotide position from start of gene | | | | | | | | | | | | | | | | | | | | | |
| --- | --- | --- | --- | --- | --- | --- | --- | --- | --- | --- | --- | --- | --- | --- | --- | --- | --- | --- | --- | --- | --- | --- | --- | --- |
|  |  |  | **39** | 45 | 51 | 65 | **91** | 100 | 141 | 162 | **165** | **168** | 198 | **210** | 243 | 271 | 280 | 297 | 393 | 402 | 429 | 471 | 483 | 504 |
| BIII | 2434 | AY368165 | **G** | T |  | A | **C** |  |  | G | **C** | **C** |  | **G** | C | C | A | A | T | A | G | A | A | C |
| BIII | BAH-12 | AF069561 | **G** | T |  | A | **C** |  |  | G | **C** | **C** |  | **G** | C | C | A | A | C | A | G | A | A | C |
| BIII-like | 3920 | AY368166 | **G** | T |  | A | **C** |  |  | G | **T** | **C** |  | **G** | C | C | A | A | C | G | G | A | G | C |
| BIV-like | 7237 | AY368168 | **A** | T |  | A | **T** |  |  | G | **T** | **T** |  | **G** | C | C | A | A | C | A | G | A | A | C |
| BIV | AD-19 | AF069560 | **A** | T |  | A | **T** |  |  | G | **T** | **T** |  | **A** | C | C | A | A | C | A | A | A | A | C |
|  | | |  |  |  |  |  |  |  |  |  |  |  |  |  |  |  |  |  |  |  |  |  |  |
| Nucleotides | | |  |  |  |  |  |  |  |  |  |  |  |  |  |  |  |  |  |  |  |  |  |  |
| C |  |  |  | 3 |  |  | **41** | 115 | 115 |  | **38** | **51** | 112 |  | 115 | 111 |  |  | 113 |  |  |  |  | 112 |
| T |  |  |  | 109 |  |  | **46** |  |  |  | **42** | **40** | 2 |  | 5 | 1 |  |  | 6 |  |  |  |  | 0 |
| Y (C/T) |  |  |  | 8 |  |  | **33** | 5 | 5 |  | **40** | **29** | 6 |  | 0 | 8 |  |  | 1 |  |  |  |  | 8 |
| A |  |  | **54** |  |  | 114 |  |  |  | 3 |  |  |  | **38** |  |  | 111 | 115 |  | 113 | 23 | 114 | 112 |  |
| G |  |  | **37** |  | 115 | 0 |  |  |  | 95 |  |  |  | **67** |  |  | 1 | 2 |  | 1 | 94 | 0 | 0 |  |
| R (A/G) |  |  | **29** |  | 5 | 4 |  |  |  | 22 |  |  |  | **1515 15** |  |  | 8 | 3 |  | 6 | 3 | 0 | 8 |  |
| M (A/C) |  |  |  |  |  | 2 |  |  |  |  |  |  |  |  |  |  |  |  |  |  |  | 6 |  |  |

*The digits indicate the number of isolates with C, T, Y, A, G, R or M at a particular nucleotide position. Only those positions where 5 or more isolates had nucleotide substitutions are included. Nucleotide positions highlighted in bold are the substitution patterns proposed as markers for different B sub-assemblages in Table 3 (Wielinga and Thompson, 2007).

**Supplementary table S2C.** Intra genotypic nucleotide substitutions at the gdh locus for 120 assemblage B isolates from the present study*.

| Sub-assemblage | Isolate | GenBank acc. no. | Nucleotide position from start of gene | | | | | | | | | | | | | | | | | |
| --- | --- | --- | --- | --- | --- | --- | --- | --- | --- | --- | --- | --- | --- | --- | --- | --- | --- | --- | --- | --- |
|  |  |  | 297 | **309** | 357 | 360 | 414 | **429** | **447** | 468 | 486 | 519 | **540** | 546 | **561** | 570 | 597 | 606 | **612** | 636 |
| BIII | BAH12 | AF069059 | C | **C** | T | G | C | **T** | **T** | G | T | C | **C** | C | **C** | C | C | C | **G** | T |
| BIII-like | gd-ber1 | DQ090532 | C | **C** | T | G | C | **T** | **T** | G | T | C | **T** | C | **C** | C | C | C | **G** | T |
| B-central | NLH25 | AY826193 | T | **C** | T | G | C | **C** | **C** | G | T | C | **T** | C | **C** | C | C | C | **G** | T |
| B-IV-like | NLH35 | AY826197 | C | **T** | C | G | C | **C** | **C** | G | T | C | **T** | C | **C** | T | T | C | **A** | T |
| BIV | Ad-7 | L40508 | C | **T** | T | G | C | **C** | **C** | G | T | C | **T** | C | **T** | C | C | C | **A** | T |
| BIV | Van/89/UBC | AY178750 | C | **T** | C | G | C | **C** | **C** | G | T | C | **T** | C | **T** | C | C | C | **A** | T |
|  | | |  |  |  |  |  |  |  |  |  |  |  |  |  |  |  |  |  |  |
| Nucleotides | | |  |  |  |  |  |  |  |  |  |  |  |  |  |  |  |  |  |  |
| C |  |  | 95 | **17** | 42 |  | 115 | **54** | **53** |  | 1 | 59 | **18** | 82 | **74** | 91 | 93 | 110 |  | 0 |
| T |  |  | 0 | **57** | 30 |  | 0 | **28** | **24** |  | 114 | 10 | **47** | 6 | **24** | 12 | 12 | 1 |  | 109 |
| Y (C/T) |  |  | 25 | **46** | 48 |  | 5 | **38** | **43** |  | 5 | 51 | **55** | 32 | **22** | 17 | 15 | 9 |  | 11 |
| A |  |  |  |  |  | 7 |  |  |  | 0 |  |  |  |  |  |  |  |  | **50** |  |
| G |  |  |  |  |  | 81 |  |  |  | 114 |  |  |  |  |  |  |  |  | **21** |  |
| R (A/G) |  |  |  |  |  | 32 |  |  |  | 6 |  |  |  |  |  |  |  |  | **49** |  |

*The digits indicate the number of isolates with C, T, Y, A, G or R at a particular nucleotide position. Only those positions where 5 or more isolates had nucleotide substitutions are included. Nucleotide positions highlighted in bold are the substitution patterns proposed as markers for different B sub-assemblages in Table 2B (Wielinga and Thompson, 2007).
